# Molecular Changes during Germination of Cocoa Beans, Part 2

**DOI:** 10.1021/acs.jafc.4c03524

**Published:** 2024-07-29

**Authors:** Konrad Brückel, Timo D. Stark, Corinna Dawid, Thomas Hofmann

**Affiliations:** ^†^Food Chemistry and Molecular Sensory Science, TUM School of Life Sciences ^‡^Professorship for Functional Phytometabolomics, TUM School of Life Sciences, Technical University of Munich, Lise-Meitner-Straße 34, 85354 Freising, Germany

**Keywords:** fermentation, UHPLC, MS/MS, metabolites, HMG glucosides, HOJA sulfate, HOJA, THOA

## Abstract

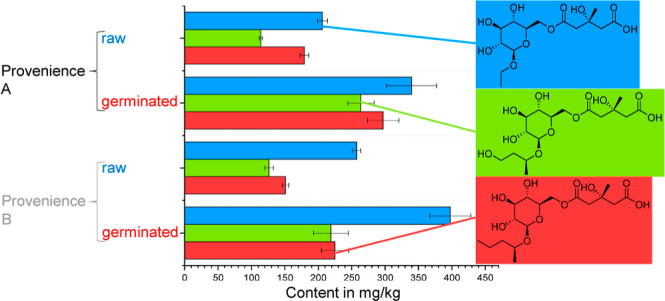

A recently published untargeted metabolomics approach toward marker compounds of cocoa germination revealed and identified 12-hydroxyjasmonic acid sulfate, (+)-catechin, and (−)-epicatechin as the most downregulated compounds and two hydroxymethylglutaryl glucosides (HMG gluc) A and B, among others, as the decisive upregulated compounds in the germinated material. These findings were quantitatively evaluated using ultrahigh-performance liquid chromatography–tandem mass spectrometry not only in previously examined sample material but also in a vastly expanded array of cocoa samples of different provenience and process and in cocoa products such as cocoa liquor and chocolate. Hereby, yields of newly identified HMG gluc derivatives could be determined in raw, fermented, germinated, and alternatively processed cocoa, and isomers of HMG gluc A and B could be established as key process indicators. Based on unsupervised clustering and supervised classification, models could identify germinated samples in testing sets consisting of raw, fermented, and germinated samples.

## Introduction

Traditionally harvested cocoa beans undergo a wild fermentation process before the drying and roasting steps, followed by further refining processes. However, this process relies on the presence of certain microorganisms and defined environmental conditions (moisture, temperature, and processing time) to achieve a successful outcome.^1−3^ Previous studies have reported that, during this first step, not only yeast and bacteria but also the onset of germination may affect the metabolome of the cocoa seed.^3,4^ While the role of fermentation has been vastly studied, the contribution of the latter remains partly unknown.^5,6^ Previous work has revealed the effects of germination with regard to the proteome and peptidome of cocoa.^7,8^ Moreover, enzyme activity had been detected even in dried beans.^9^ A curing treatment, which comprised the application of moisture to raw cocoa beans, resulted in an altered profile of odor-active compounds.^10−12^ This novel treatment yielded a final chocolate product with a decreased perception of the taste attributes of astringency and bitterness and a reduced content of acetic acid as compared to chocolate produced from fermented (F) and unfermented beans from the same origin.^13^ In this context, it would be relevant to understand if chocolates obtained from nonfermented beans, in which germination was favored against fermentation, could be distinguished from the products of F beans.

Theobromine,^14^ 2,5-diketopiperazines,^15,16^ flavan-3-ols, and polyphenols had been highlighted as the major contributors to the astringent and bitter taste of raw and F beans as well as roasted nibs.^17−21^ However, it remained in the dark if further unknown taste active compounds could be affected by curing and which metabolic processes might explain the described flavor alterations in cured cocoa. Thus, in a recent paper, several compounds, which might be characteristic markers of cocoa germination, had been identified using a metabolomics approach.^22^ Besides (+)-catechin, (−)-epicatechin, and 12-hydroxyjasmonic acid (HOJA) sulfate, which appeared to be downregulated in the germinated (G) samples, the presence of two 3-hydroxy-3-methylglutaryl glucosides (HMG gluc) and two trihydroxy octadecenoic acid isomers could be confirmed in cocoa, which seemed to be upregulated in the G samples. Therefore, a targeted and quantitative method needs to be developed to verify these findings obtained by a nontargeted approach, and the scope of samples needs to be enlarged to evaluate the influence not only of the process but also of the provenience.

HMG gluc were first identified in plants by Wald et al.,^23^ but only HMG gluc A had been previously described in cocoa.^24,25^ Moreover, for the first time, additional HMG gluc were found in cocoa samples, which could be confirmed via coelution with standard compounds.^22^ Among these, only HMG gluc D (Licoagroside B),^26−28^ HMG gluc E,^29,30^ HMG gluc G,^29,31^ HMG gluc J,^32^ HMG gluc M,^33^ and HMG gluc O had been previously described in various plant materials.^34^ These compounds might also be included in the quantification to evaluate the role of HMG gluc within the cocoa process.

In this study, a quantification method was established, validated, and used to determine the proposed marker candidates and further HMG gluc in a variety of cocoa beans, liquor (i.e., ground roasted cocoa beans), and chocolate samples of different proveniences and treatments. Key process indicators (KPIs) were defined to enable the characterization of the underlying cocoa process and the origin of cocoa products.

## Materials and Methods (Including Safety Information)

### Chemicals

9,10,13-(*S*,*S*,*S*)-Trihydroxy-(11*E*)-octadecenoic acid (=9,10,13-(11*E*)-THOA) and 9,12,13-(*S*,*S*,*S*)-trihydroxy-(10*E*)-octadecenoic acid (=9,12,13-(10*E*)-THOA) were delivered as ethanolic solutions from Larodan Inc. (Malmö, Sweden). 3-Hydroxy-3-methylglutaryl glucoside (=HMG gluc) A was provided as a synthesis product from Mars, Inc. (Hackettstown, NJ, USA). HMG gluc C, D, E, F, G, H, I, J, K, L, M, N, and O were commercially received from AnalytiCon Discovery GmbH (Potsdam, Germany) as isolates from different plant species. Sodium hexyl sulfate, (+)-catechin and (−)-epicatechin were purchased from Merck. HOJA and its sulfate were synthesized according to an adapted protocol by Jimenez-Aleman et al., as described previously.^22,35^ Stock solutions of the standard compounds were prepared in MeOH-*d*_4_ and DMSO-*d*_6_. Quantitative ^1^H nuclear magnetic resonance spectroscopy (NMR) was used to determine the concentrations of the standard stock solutions.

Acetonitrile (ACN) and methanol (MeOH) used as chromatography solvents and for mass spectrometry (MS) were purchased from CLN (Niederhummel, Germany) in LC-MS purity. Water as a solvent was prepared by filtration with an AQUA-Lab—B30—Integrity system (AQUA-Lab, Ransbach-Baumbach, Germany), and aqueous solvents used for chromatography were renewed after 1 week. Formic acid was received from Merck (Darmstadt, Germany) in >98% purity for use as a modifier for chromatography. All samples, as presented in Table S1 of the Supporting Information, were obtained from the food industry and stored at 5 °C in the absence of light. Sample solutions and ground samples were stored at −18 °C until further preparation and measurement.

### Preparation of Samples and Standard Solutions

#### Homogenization

Bean samples were prepared as follows: about 10 g (which equals about ten cocoa beans) was peeled, frozen in liquid nitrogen, and instantly ground with a material beater at 25 krpm for 10 s in an IKA A10 basic mill (IKA-Werke GmbH & Co., KG, Staufen, Germany). The resulting powder was transferred to glass bottles under an argon atmosphere until use and stored at −18 °C. Aliquots of about 100–200 mg of sample material (cocoa bean powder/cocoa liquor) were accurately weighed into sample tubes (Precellys lysing kit CK Mix; consisting of zirconium oxide mix beads of 1.4 and 2.8 mm in 2 mL standard tubes) provided by Bertin Corp. (Rockville, USA) in triplicate and stored at −18 °C until extraction.

#### Extraction

The tubes containing the sample material were brought to room temperature. According to the method development (described in the Supporting Information), approximately 1.0 mL of extraction solvent mix [7:3 MeOH/water (v/v)] and of internal standard mix (35 μL) were added, the tubes were tightly closed, and incubation was performed in a lab shaker (30 °C, 1200 rpm, 60 min). The samples were then cooled to −18 °C for 30 min, extracted in a Precellys (Bertin Corp., Rockville, USA) grinding device (3 × 30 s, 6000 rpm, 20 s break), and immediately centrifuged afterward in a Mini Spin (Eppendorf SE, Hamburg, Germany) device (2 × 13.4 krpm, 5 min). The supernatant was then decanted into 2.0 mL tubes (Eppendorf SE, Hamburg, Germany).

#### Defatting

Approximately 1.0 mL of distilled *n*-pentane was added to the supernatant after extraction to remove apolar compounds, which might affect chromatographic performances. Extraction was performed in a lab shaker (300 rpm at room temperature for 30 min). The pentane phase was removed with an Eppendorf pipet and disposed, and the lower phase was extracted with *n*-pentane again (1 mL, 300 rpm, at room temperature for 30 min).

#### Membrane Filtration

A 0.1–1 mL Injekt-F Luer Solo syringe (Braun Melsungen AG, Melsungen, Germany) equipped with a hypodermic needle (0.80 × 50 mm BL/LB, 21G x 2″, Sterican Braun Melsungen AG, Melsungen, Germany) was used to retrieve the bottom phase after extraction. This defatted extract was passed through a membrane filter (Minisart RC, hydrophilic, nonsterile, 15 mm, 0.45 μm pore width, Sartorius Stedim Biotech GmbH, Goettingen, Germany) into HPLC vials (N9, flat, label, screw neck, 1.5 mL/11.6 × 32 mm amber, Macherey-Nagel GmbH & Co., KG, Düren, Germany). The filtered extract was then diluted in the extraction solvent for the ultrahigh-performance liquid chromatography-MS (UHPLC/MS) measurement [1:10 (v/v) for marker and HMG gluc determination and 1:100 (v/v) for (−)-epicatechin/(+)-catechin determination].

#### Internal Standard Mix

Sodium hexyl sulfate (6.41 mg) was dissolved in MeOH/water 1:1 (1 mL, v/v). HMG gluc I was dissolved in DMSO-*d*_6_ (2.78 mg, 600 μL) and diluted 1:100 (v/v) in a MeOH/water mixture of 7:3 (1.0 mL, v/v). Aliquots of these stock solutions (30 μL of hexyl sulfate stock solution and 500 μL of HMG gluc I stock solution) were poured into a 1 mL volumetric flask and filled up until marked with MeOH/water 7:3 (v/v). Approximately 20 μL of this solution was added to each sample before the workup. For the calibration of marker compounds in the Waters Xevo TQ-S system, a 1:5 (v/v) dilution was prepared, and 20 μL was added to each calibration solution.

#### Calibration Solutions

Details on the preparation of calibration solutions are available in the Supporting Information (Tables S2 and S3).

#### Spiking Experiments

Spiking experiments were designed to evaluate the method’s performance and determine the influence of matrix effects on quantification. Screening of the test sample set could not provide a blank sample, indicating a matrix sample free of analytes. Therefore, beans with low contents for most of the analytes were used as the matrix for spiking.

To evaluate the recovery at several concentration steps, the natural content was spiked with 0.5, 1.0, 2.0, and 4.0 equiv for all standards available, each in triplicate. HMG gluc other than HMG gluc A, B, and C were spiked in one assay, and the remaining standards were spiked in a separate assay. HOJA was spiked in a third separate assay to avoid carryover from synthesis traces in the HOJA sulfate standard. These spiking solutions were directly produced from quantitative NMR solutions, which were mixed in volumetric flasks to obtain their respective concentrations of four equivalents. These solutions were diluted 1:2, 1:4, and 1:8 using DMSO and ACN/water 7:3 (v/v) in such ratios to provide the same solvent mixtures for each step. Moreover, these solvent mixtures were also used for the solvent blank samples in each assay.

### Ultrahigh-Performance Liquid Chromatography–Tandem Mass Spectrometry

#### Quantification of Marker Compounds

Electrospray ionization (ESI) mass spectra and product ion spectra were acquired by using a Waters Xevo TQ-S mass spectrometer. The MS/MS system was operated in the MRM mode, detecting negative ions at the following ion source parameters: capillary voltage at −2.00 kV, source offset at 50.0 V, source temperature at 150 °C, desolvation temperature at 600 °C, cone gas flow at 150 L/h, desolvation gas flow at 800 L/h, collision gas flow at 0.15 mL/min, and nebulizer gas flow at 7.00 bar. The dwell time was adjusted to 9 ms for each measured transition. The column oven temperature was adjusted to 50 °C. For analysis of the metabolites, the MS/MS parameters were tuned to achieve fragmentation of the [M–H]^−^ molecular ions into specific product ions, with the optimized parameters illustrated in Table S4 (Supporting Information). HMG gluc I was chosen as an internal standard for the other HMG gluc due to its structural similarity and their absence in the previously analyzed cocoa samples. For similar reasons, hexyl sulfate was used as an internal standard for HOJA sulfate. During the measurement of several sample sets, the detected intensities of (−)-epicatechin exceeded the linearity range of the method, requiring further dilution of the already 1:10 (v/v) diluted extracts. Consequently, the internal standard would have been diluted in the same way. Thus, an additional workup for the quantification of (−)-epicatechin using a higher internal standard dosage would have been necessary, which was not possible given the restricted amount of internal standard available. Therefore, an external calibration for the quantification of (−)-epicatechin was utilized.

For tuning, ACN/water solutions of each analyte and internal standard were introduced by means of flow injection, using a syringe pump. Analytical separation using aliquots of 2 μL of sample solution was performed on an Acquity UHPLC I-Class System (Waters, Milford, MA, USA) composed of a binary solvent manager, sample manager, and column oven fitted with ACQUITY UPLC 2.1 mm × 150 mm, 130 Å, 1.7 μm, BEH C18 column (Waters, Manchester, United Kingdom), coupled to a Waters Xevo TQ-S mass spectrometer (Waters, Milford, MA, USA). The system was run with MassLynx 4.1 Software (Waters), and data processing and analysis were executed with TargetLynx (Waters). Operated with a constant flow rate of 400 μL/min, the mobile phase was mixed from solvent A (0.1% formic acid in water) and solvent B (0.1% formic acid in ACN) using the following gradient: starting with 5%, solvent B was increased to 30% in 10 min and furthermore increased to 99% within 2 min. After 2 min at 99%, solvent B was reduced to 5% within 1 min, and equilibration at these starting conditions was performed for 1 min.

#### Quantification of Further HMG Glucosides

All HMG gluc were tuned with an AB Sciex 6500 mass spectrometer (Darmstadt, Germany), which was used to obtain ESI mass spectra as well as product ion spectra. For tuning, the ACN/water solutions of each analyte and internal standard were introduced by means of flow injection using a syringe pump. Table S5 in the Supporting Information summarizes the optimized parameters after tuning.

The samples were separated by an ExionLC UHPLC with a Kinetex 2.1 × 100 mm, 100 Å, 1.7 μm, C18 column (Phenomenex, Aschaffenburg, Germany). The gradients and solvents used were identical to those used for the quantification of the marker compounds. Data acquisition and instrumental control were performed with Analyst version 1.6.3 software (Sciex, Darmstadt, Germany). Data evaluation/integration was performed by MultiQuant software (Sciex).

#### Method Validation

A detailed description of the method validation can be found in the Supporting Information: results for limit of detection/limit of quantification (LOD/LOQ) and linearity range are presented in Table S6, while results for recovery and robustness are depicted in Table S7. Each of the triplicates of spiked solutions was measured twice (thus resulting in six intraday replicates), and the average values and standard deviations were calculated. The recovery *R* was calculated as the ratio of the concentration measured in the spiked sample to the sum of the endogenous concentration and the spiked concentration.

### Data Evaluation

Calculations of regressions, analyte concentrations, and analyte ratios were performed with Microsoft Excel. Mean values and standard deviations of analyte concentrations and ratios were calculated and plotted using an Origin Lab. Data for heatmaps were preprocessed in Python (version 3.11.5) and plotted by Seaborn data visualization library (version 0.13.2), using *z*-scores (standardized to normal distribution) of mean values obtained from six technical replicates (three biological replicates, double measurement).

Data for box plots were preprocessed in Python and visualized in TIBCO Spotfire 7.14.0. All further data processing and evaluation were done using Python packages. From these boxplots, the most-promising features were selected according to the following criteria, which could discriminate G samples from the relict non-G samples. First, only those features were selected for which the intraclass range in the target class had no or only a minimum overlap with the intraclass range in the other classes of process types. Second, in order to avoid noise in the dataset, only those features were selected, which had minimum intraclass variance. Third, in order to verify the quality of this preset of features, those were plotted against each other in a pair plot (“scatter plot matrix”), thereby visually evaluating the linear separability of the two classes (G and non-G). Next, hierarchical density-based spatial clustering of applications with noise (HDBSCAN, Python HDBSCAN package version 0.8.37) was applied, using a minimum cluster size of 2 and the cluster selection method excess of mass (EOM), to cluster the data points in order to verify that the G samples can be isolated using the four selected features (with standard scaling applied to them). HDBSCAN was chosen for hierarchical clustering because it was obvious that the data contained noise. As there were some interfeature correlations observed, two-dimensional principal component analysis (PCA) (Scikit-learn version 1.5.1) was used to examine, if the dimensionality of the classification problem could be reduced without loss of vital information derived from variance. Therefore, the results of the selected features were normalized by standard scaling before being reduced to two dimensions by PCA. Fourth, HDBSCAN was also performed after two-dimensional PCA in order to evaluate if clustering was still feasible after a reduction of dimensions to two. The results of the HDBSCAN processes were visualized by scatter plots and confusion matrices (Seaborn data visualization library).

To confirm the results, supervised classification by linear regression (Scikit-learn version 1.5.1) was chosen to determine, if an algorithm based on the PCA dimensions could be trained with a set of G and non-G samples, which was subsequently validated with a test set also consisting of G and non-G samples. Three different classification problems were examined by using a separate model for each (classifier), for which the dimensions of the standard-scaled features were reduced to two dimensions through PCA. Each of these classifiers was cross-validated against at least three differently sampled partitions, each splitting the whole dataset into a training and a test dataset. In the first classification model, G and FG (fermented and germinated) samples were combined as the G class (G + FG), and classification was based on the PCA dimensions of all four features.

In the second model, only G samples were allocated in the target class (G), and FG samples were combined with all other processing types in the second class (non-G + FG). For this model, HOJA was dropped as a feature, and the remaining features were scaled to two PCA dimensions, as HOJA was initially selected to classify FG samples. In every partition of this model, the raw counterpart (same origin) of the G sample was present in their respective test or training set. This was to ascertain whether the G sample could be identified against another non-G sample of the same origin so that any differences in analyte concentration due to influences of region, plant, environment, etc., could be ruled out.

In the third model, again only G samples were marked as target class (G), and in every partition, like model 2, the raw counterpart of the G sample was included in the testing or training sets respective to the G sample, and HOJA was excluded as a feature before PCA. Also, in addition to that, all FG samples and their fermented counterparts were included only in the testing sets of each cross-validated model (the training set had no FG samples or their counterparts), which proved that G samples could be classified against all fermented samples, whether or not G.

## Results/Discussion

### Quantification Results

The previously established and validated LS-MS/MS methods were consequently used for the quantification of these compounds in sample sets of different origins and process parameters. Hereby, the identified marker candidates were measured at the Waters system, while HMG gluc were quantified at the Sciex system to cover the disparity of endogenous concentrations.

#### Comparison of Marker Contents in Profiling Samples

In a first sample batch, samples 8–15 from Batch 1 (Table S1, Supporting Information) composed of raw, fermented, and G cocoa liquor were measured, in which the nontargeted profiling had been based on.^22^ Furthermore, markers and further HMG compounds were quantified in the corresponding bean samples 16–23 from Batch 2 (Table S1, Supporting Information). Figure S2A–E (Supporting Information) highlights the resulting contents (received as the mean value of six replicates per sample). The top eight samples comprise cocoa liquor (Liq) samples, while the bottom eight samples comprise the corresponding dried cocoa bean material. For sample origins of Southeast Asia (SEA) type 1a and 1b, each of the raw materials (raw) and the corresponding G materials is depicted in pairs. SEA type 1 samples include F material and material that is produced in a mixed process combining FG. Moreover, two additional samples of Latin American (LA) origin are included, with samples of type 1a having undergone only a short time fermentation and samples of type 1b having a higher degree of fermentation.

As depicted in Figure S2A (Supporting Information), HOJA sulfate and (+)-catechin contents in the G samples are lower than in the corresponding non-G samples of the same provenience. Especially, these differences can be seen for the proveniences of SEA types 1 and 1a. On the one hand, the content of (−)-epicatechin appears to be higher in the nonfermented material (i.e., G and raw material) than in the F counterparts. In the samples of LA origin, the content is reduced in the high-F material compared with the low-F corresponding samples. In conclusion, fermentation reduced the content of (−)-epicatechin and (+)-catechin, which had been shown by Payne et al. for the traditional fermentation process.^18^ On the other hand, no noticeable differences were observed in the (−)-epicatechin content between G samples and their corresponding raw materials. HOJA sulfate, which was described in cocoa by Patras and Milev, was also reduced in the F samples and in the G samples compared to the corresponding raw samples.^36,37^ This confirms the findings by Hurst, suggesting a reduction of the HOJA sulfate content due to fermentation.^38,39^ Miersch reported that 12-hydroxyjasmonate sulfate and 12-hydroxyjasmonate, among other jasmonate derivatives, play an important role in plant growth and germination, which might explain the observed reduction of HOJA sulfate content in the G samples.^40^

As depicted in Figure S2B (Supporting Information), a noteworthy upregulation of HMG gluc A and B in the G samples confirms the results of the nontargeted approach, where both compounds had been assigned as marker candidates due to their high absolute *p*1 values in the *S*-plot.^22^ The results of the further, even lower concentrated marker compounds are discussed in the Supporting Information. Nevertheless, the absolute differences in concentrations determined for these assumed marker compounds appear relatively low, and the maximum fold change ranges between two and three for the highest potent candidates. To obtain a more dependable criterion characterizing the cocoa process, ratios from upregulated (e.g., HMG gluc A) and downregulated compounds by germination (e.g., HOJA sulfate) were calculated. This way, higher fold changes between differently processed samples should be achieved, and variances due to other factors that influence absolute concentrations should be limited as far as possible. These ratios should be evaluated with regard to their ability to characterize the underlying process.

While, in some of the samples, ratios with HOJA sulfate increased the variance due to germination, this was not successful with (+)-catechin and (−)-epicatechin ratios (Supporting Information). Thus, it was decided to investigate the promising marker compounds along with their respective HOJA sulfate ratios in larger sample sets.

#### Comparison of Marker Contents within All Samples

As depicted in the heatmap (Figure S6, Supporting Information), this upscaled quantification confirmed the results of the sample set of the profiling. Indeed, the three HMG gluc B isomers, HMG gluc A isomer 1, HMG gluc C isomer 2, and HMG gluc H, impart the highest contents only in the G samples (red; samples 8, 9, 21, and 23), whereas their contents are distinguishably lower in the remaining samples. Apart from these standardized quantification data, a look at the absolute concentrations of these newly discovered HMG gluc highlights the high variance within the dataset not only between different samples but also analytes. Given the results of the quantification (Table S8, Supporting Information), the concentration of HMG gluc A isomer 1 varies between 25 and 378 mg/kg, while isomer 2 ranges from a lower LOQ to 67 mg/kg. Although HMG gluc A isomers have been described and patented in cocoa, among other HMG gluc derivatives, no quantitative data regarding their natural occurrence in cocoa has been published yet.^24,25^ HMG gluc B, which had also been previously identified in cocoa,^22^ was found in concentrations ranging from 0.02 to 1.3 mg/kg (for isomer 1), 1.0 to 264 mg/kg (for isomer 2), and 0.8 to 161 mg/kg (for isomer 3). HMG gluc H was found in an even higher concentration range from 5.8 to 419 mg/kg, while the remaining HMG gluc were found in concentrations lower than 28 mg/kg (HMG gluc E). In summary, 22 newly identified analytes (including isomers), which had been identified in a previous study,^22^ could be determined in 82 cocoa samples of various proveniences and processing types for the first time (depicted in Table S8, Supporting Information).

According to the results described in the previous section, marker ratios of ω_analyte_/ω_HOJA sulfate_ appeared to be promising as additional characteristic criteria. When these ratios were calculated for the concentration measured in the overall sample set upon aggregation by processing type, this picture turned out to be inconclusive, as depicted in Figure S7 (Supporting Information). In order to better understand the distribution of values of analytes within the processing type classes, both the concentrations and the HOJA sulfate ratios have been visualized in box plots (Figures S8–S19, Supporting Information). It can be ascertained that HMG gluc B isomers 2 and 3 are highly upregulated in the G class, while HMG gluc A isomer 1 is upregulated in both the FG and G samples. HOJA, on the other hand, appears to be elevated in FG samples only. Even though HOJA sulfate had a narrow intraclass range for FG and G samples, it varied heavily and had outliers for raw and F samples. As some of the samples had concentrations similar to FG and G, HOJA sulfate and its ratios (see Supporting Information) were dropped for further consideration as a feature for classification. In order to verify this assumption, an unsupervised HDBSCAN approach was used, coupled with a logistic regression approach.

### Data Analysis

#### Selection of Features

**Figure 1:**
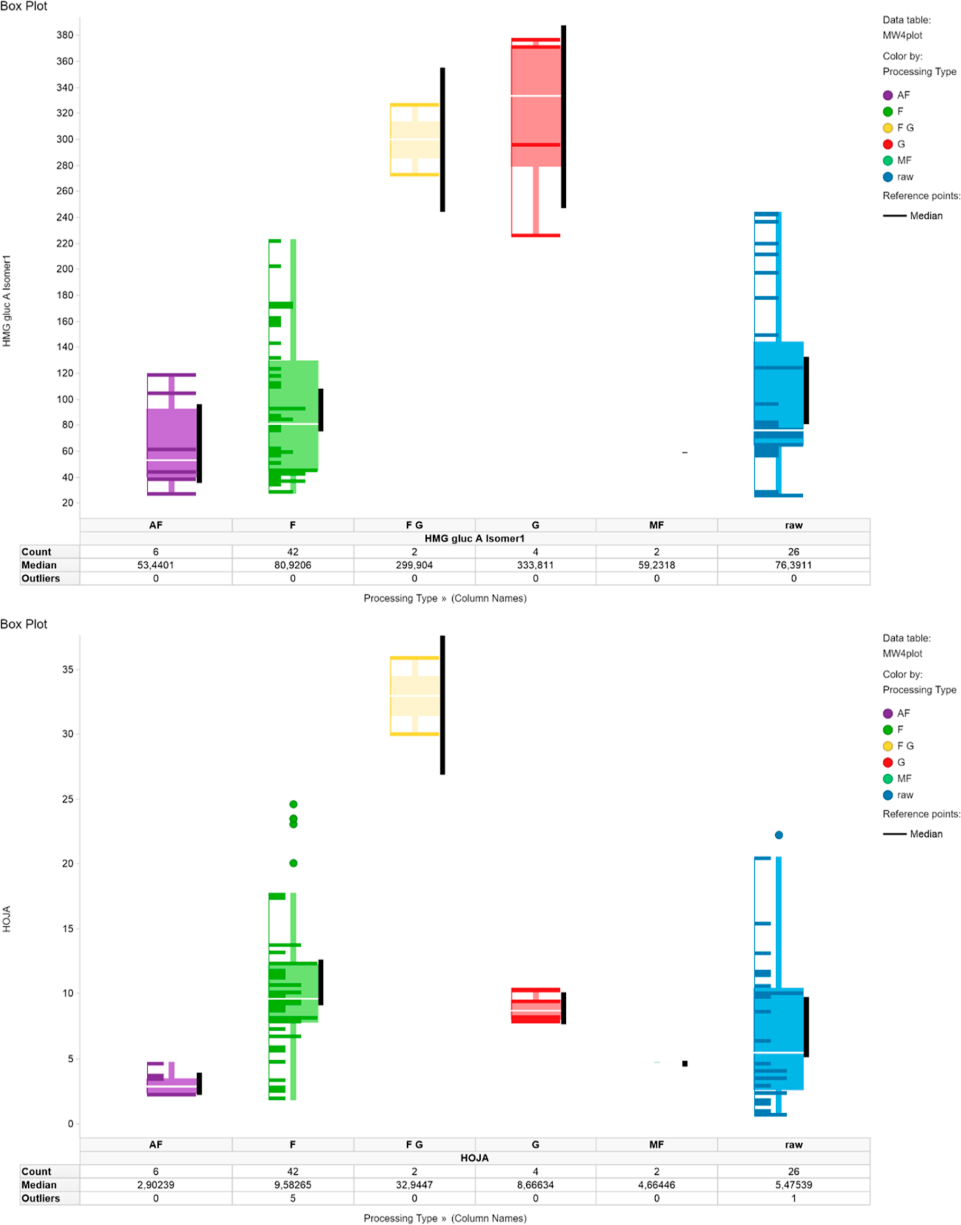
Boxplots of mean values of ω_HMG gluc A isomer 1_ (top) and ω_HOJA_ (bottom) highlight the distribution of samples grouped by process type: fermented (F; in light green), raw (blue), germinated (G; red), alternatively fermented (AF; purple), combination of fermentation and germination (FG; orange), and microfermented (MF; dark green). Black bar marks a confidence interval on a level of 95%.

**Figure 2:**
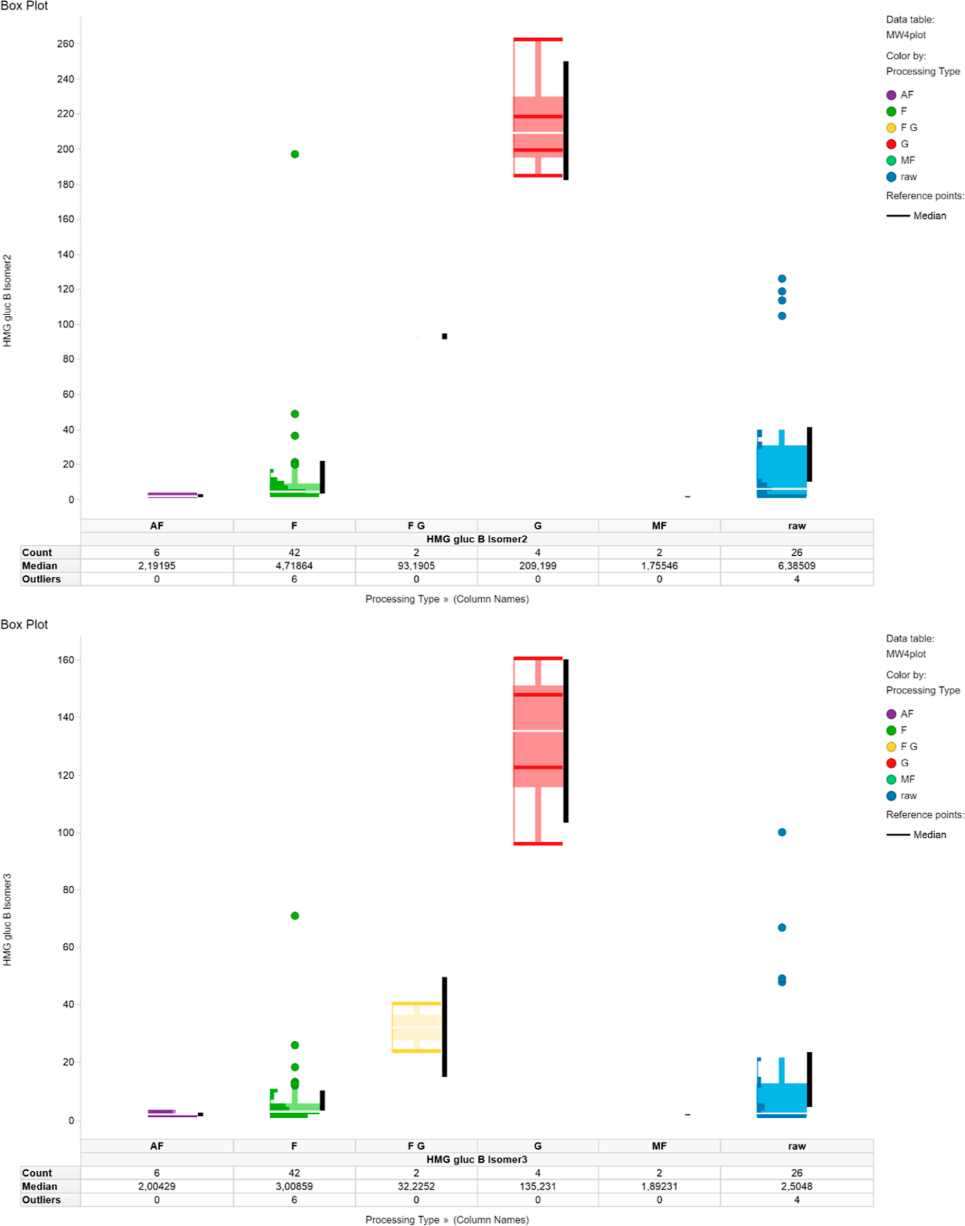
Boxplots of mean values of ω_HMG gluc B isomer 2_ (top) and ω_HMG gluc B isomer 3_ (bottom) highlight the distribution of samples grouped by process type: F (light green), raw (blue), G (red), AF (purple), combination of FG (orange), and MF (dark green). Black bar marks a confidence interval on a level of 95%.

The concentrations of HMG gluc A isomer 1, HMG gluc B isomer 2, and HMG gluc B isomer 3 (Figures 1 and 2) were upregulated only in the G samples, while the HOJA concentration appeared to be only elevated in the FG samples, thus could identify FG from the other classes. These four concentrations could be selected as features for further data analysis.

#### Unsupervised Clustering

**Figure 3:**
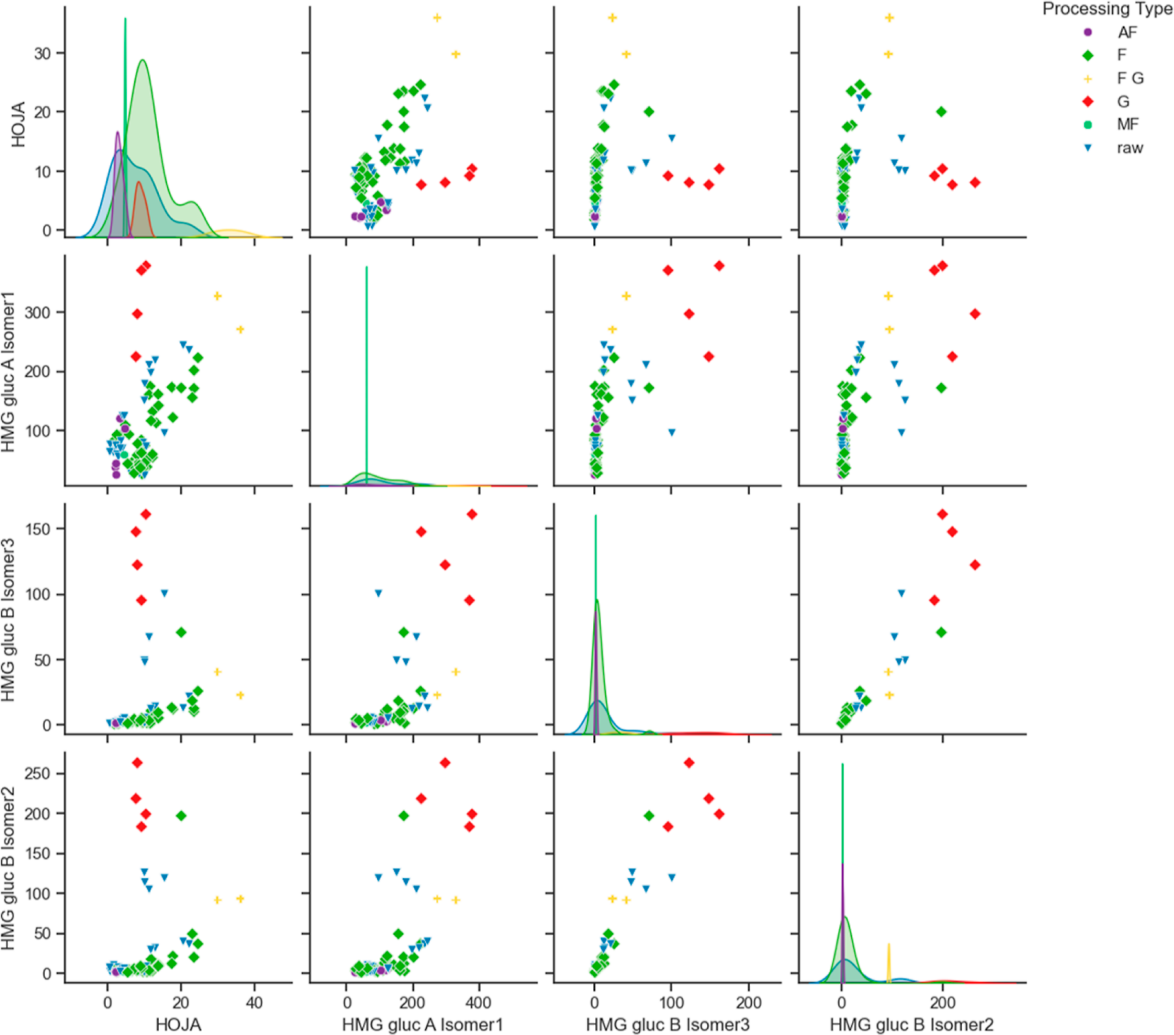
Pair plot (scatter plot matrix) of four selected features, highlighting the distribution of samples by processing type: F (light green), raw (blue), G (red), AF (purple), combination of FG (orange), and MF (dark green). Most plots displayed a linear separability of the G samples and plots with HOJA and HMG gluc A isomer 1 also displayed the separability of FG samples.

The investigation of the influence of germination resulted in the three classes of process types: class G (4 samples G type), class FG (2 samples FG type), and class non-G (76 samples of raw, F, AF, and MF type). In an unsupervised clustering approach, it was examined if the selected features would be sufficient to group the samples in clusters that could be aligned with these three target classes. In the pair plot (Figure 3), the coordinates of data points of G and FG type are linearly separable from those of another processing type on most graphs.

Following this visual analysis, HDBSCAN was performed for unsupervised clustering on four dimensions. The results in Table 1 demonstrate that all four G samples are mathematically separable from the other samples, but clustering was not possible for the FG samples, which were excluded as outliers. Even though the algorithm is unsupervised and only clusters data points based on EOM method, it was able to cluster all the G only samples into one cluster.

**Table 1:** Clusters and Processing Type Counts Based on Four Selected Features: Here, without Supervision, the Algorithm Was Able to Put All the G Samples into One Cluster (0), while, Surprisingly, Both the FG Samples Were Excluded as Outliers (−1)

cluster	processing type	support
–1	F	3
	FG	2
	raw	2
0	G	4
1	raw	4
2	F	4
	raw	2
3	F	7
4	AF	6
	F	28
	MF	2
	raw	18

**Figure 4:**
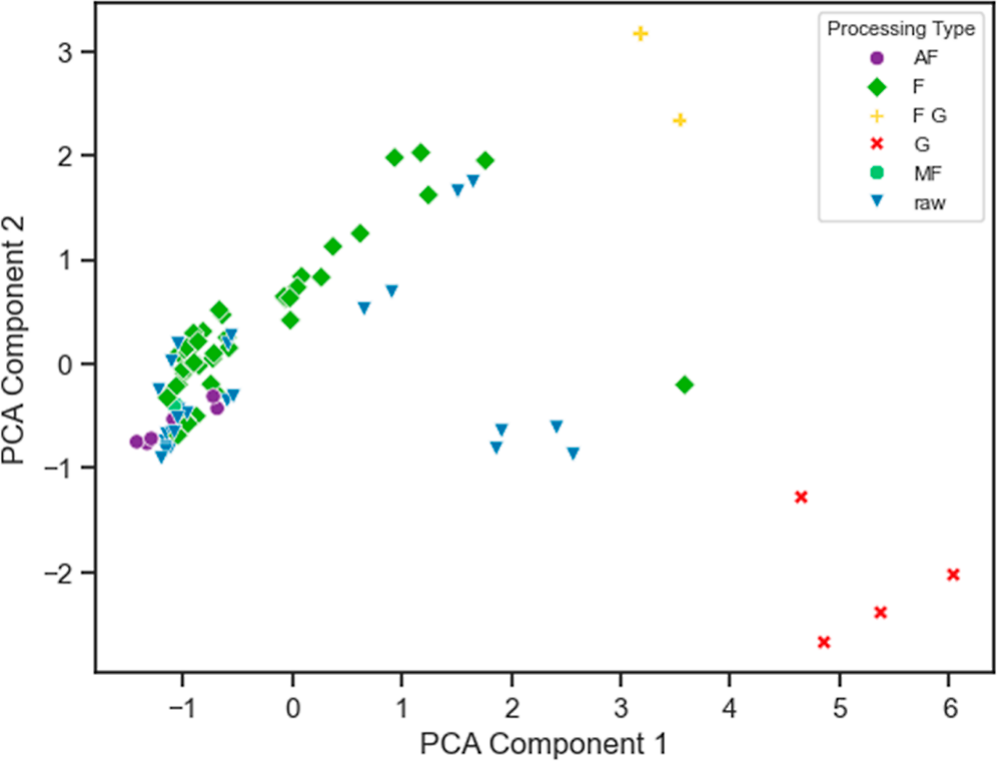
Scatter plot visualizing the results of two-dimensional PCA after standard scaling the four features, highlighting the distribution of samples by processing type: F (light green), raw (blue), G (red), AF (purple), combination of FG (orange), and MF (dark green). G as well as FG samples have linear separability.

After this, dimensionality was reduced by PCA. The first two resulting dimensions, which have a cumulative explained variance of 92.8% (i.e., 7.2% of information loss), include sufficient information to reduce clustering input data dimensionality from four to two PCA dimensions. At a glance, it seems like the cluster for G samples forms near the bottom-right of the two-dimensional plane (Figure 4).

Following, HDBSCAN was applied on the dimensions of the PCA, which revealed again that all four G samples are in one cluster, which is depicted in Table S10 (Supporting Information). Moreover, all the other non-G samples were contained in 11 further clusters, while both FG samples were found outliers along with nine other samples. Which in summary indicates that the selected features contain sufficient information, which could be used for a supervised classification after PCA with G samples as the target class. As the clustering failed to detect the FG samples as a separate cluster, the classification of FG samples appears to be less feasible.

Now, with the decreased dimensions and still linear separability being identified in the scatterplot after PCA (Figure S22, Supporting Information), it appears more probable that a supervised classification would work even better than unsupervised clustering. Supervised algorithms can bypass the limitations of unsupervised clustering and the uncertainty of how many clusters would be formed.

#### Supervised Classification

With the results of the unsupervised clustering given, a supervised approach was chosen based on logistic regression. As the aim was to verify if the selected features were able to serve as criteria to determine whether samples had undergone germination, an algorithm was established by using part of the samples for training and the other part for validating the algorithm. Due to the reasons already outlined, all supervised algorithms used PCA dimensions based on the selected features.

In the first approach of a supervised classification, the target class was defined as G and FG samples combined (G + FG), whereas all other samples were put in the class non-G. The first partition of this classification model could classify the three G samples and the FG sample in the training set as well as each G and FG sample in the test set correctly as G + FG (see Tables S12 and S13, Supporting Information). The second partition could also classify the two G samples and the two FG samples in the training set and the two G samples in the test set correctly (see Tables S14 and S15, Supporting Information). In the third partition of this model, all three G samples in the training set as well as both the G and FG samples in the test set were identified as G + FG class. However, the FG sample in the training set was falsely classified as non-G (see Tables S16 and S17, Supporting Information).

**Figure 5:**
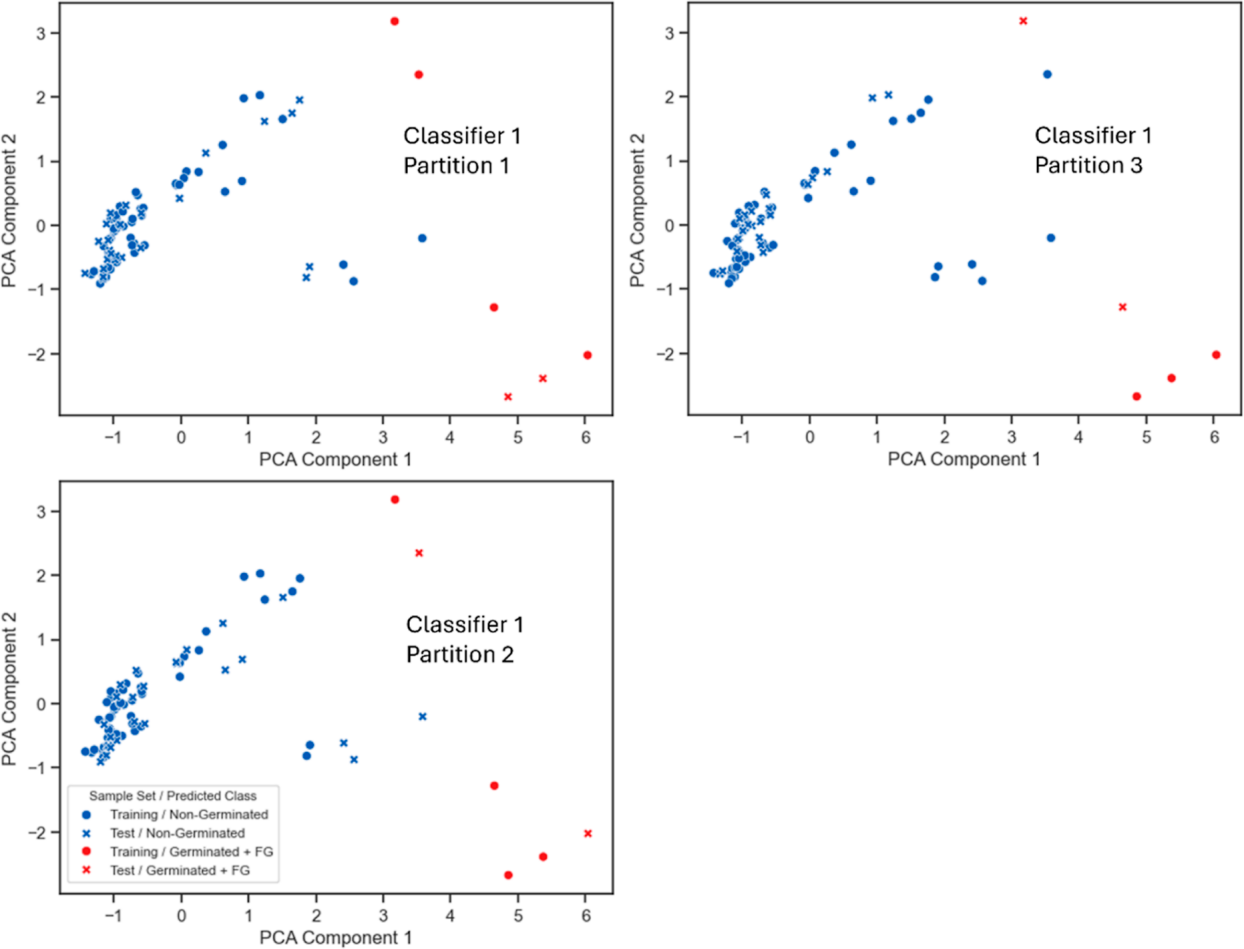
Scatter plots visualizing the results of classification after two-dimensional PCA, highlighting the prediction of the training set (•) and the test set (×) of partitions 1–3 of the 1st classifier, with the G + FG class (red) and the non-G class (blue).

Although this outcome might indicate that all six G samples (including FG samples) could be classified correctly (see Figures 5 and S24, Supporting Information), the unsupervised clustering did not support this conclusion (Table S10, Supporting Information). As the FG samples were not clustered together but were excluded as outliers in both unsupervised models, the supervised model could have been overfitted by including FG samples in the target class, which means that data for new FG samples might not be classified correctly. In order to exclude these effects, a further classifier was established, which should only identify G samples as target class (G) and FG samples as non-G (along with the remaining samples). For this purpose, “HOJA” was dropped as a variable from the set of features because that is a good differentiator of FG samples. Moreover, it appeared to add noise to the dataset if the algorithm should be taught only how to identify G samples apart from non-G + FG (FG type and other samples).

**Figure 6:**
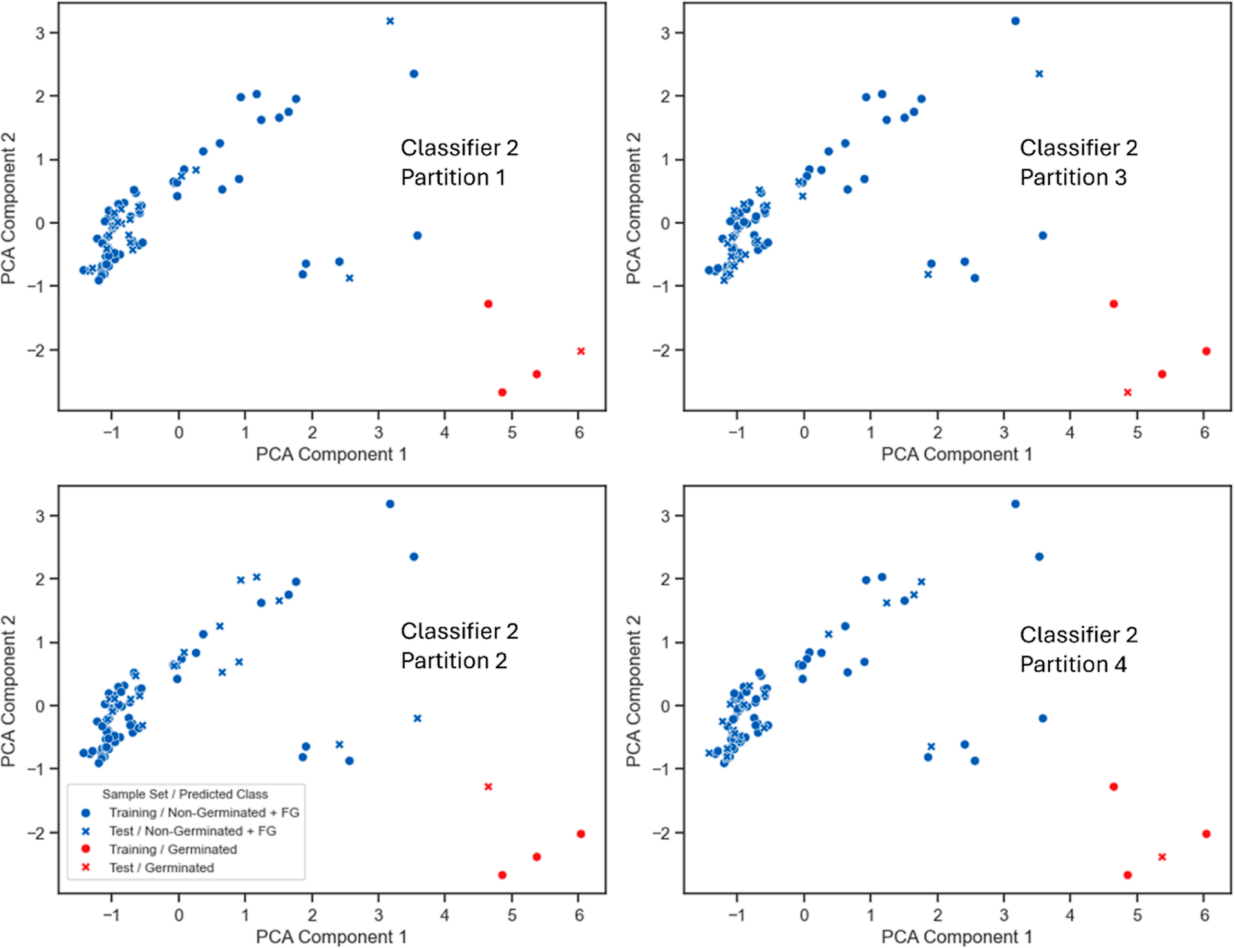
Scatter plots visualizing the results of classification after two-dimensional PCA, highlighting the prediction of the training set (•) and the test set (×) of partitions 1–4 of the 2nd classifier, with the G class (red) and the non-G + FG class (blue).

There were four different partitions of training and test subsets on this second classifier. Each run of the training and testing instance in all four partitions yielded 100% accuracy, precision, recall, and F1 score, hence making it quite clear that G samples can be identified from the rest. It can be concluded with high confidence that the model is not overfitted because it could be successfully trained and tested in differently sampled test sets with high performance. The results of this classifier are exemplarily given in Tables S18 and S19 (Supporting Information) for the first partition of four, as all four cross-validated algorithms yielded the same results for training and testing, respectively (Figures 6 and S25 and S26, Supporting Information). Additionally, this demonstrates that G samples can be identified from non-G samples from the same environmental conditions as well.

Next, a different model was chosen to determine whether the G samples could also be identified in a test set containing FG samples and their F counterpart of the same origin. This could prove the robustness of identifying G samples even if the model was not trained against FG samples.

**Figure 7:**
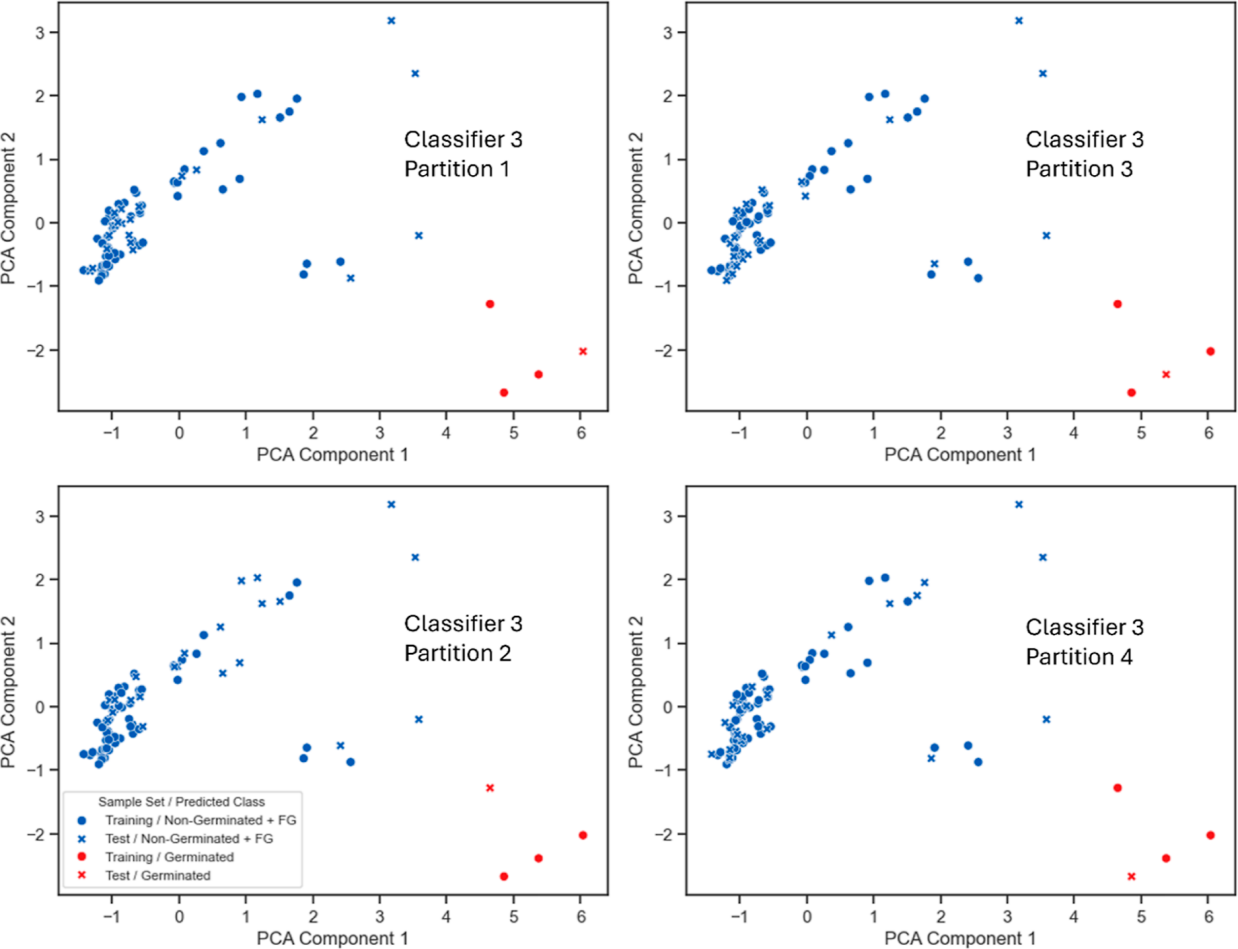
Scatter plots visualizing the results of classification after two-dimensional PCA, highlighting the prediction of the training set (•) and the test set (×) of partitions 1–4 of the 3rd classifier, with the G class (red) and the non-G + FG class (blue).

The outcome of the third logistic approach was similar to the second classification model: again, with 100% accuracy, recall, precision, F1 score, accuracy, etc., in all four different partitions of training and test sets, the G samples could be classified correctly. This ensured identification of the G samples out of all other samples, even with the noise that FG and F data produce in the test sets, without any of them helping train the model. The outcome is depicted exemplarily for partition 1 in Tables S20 and S21 (Supporting Information) and Figures S27 and S28 (Supporting Information), while the combined scatter plot for all four partitions is visualized in Figure 7.

In summary, all four selected features might be able to classify samples with a known history of germination (G and FG). These results have to be taken with a grain of salt, as they would need to be ascertained with further data collection, including FG samples.

However, when classifiers were based on the two-dimensional PCA results of only HMG gluc A isomer 1, HMG gluc B isomers 1 and 2, G only samples could be unequivocally identified even in the presence of F samples and raw samples of the same provenience. In future quantification experiments, it is recommended to increase the class balance in favor of G and FG samples in order to confirm the results of this study.

### Outlook

In this work, a new UHPLC-MS/MS method could be developed and established, enabling the measurement of a total of 22 newly identified analytes (including isomers) in 82 cocoa samples of various proveniences and processing. Hereby, unique upregulation (HMG gluc A/B isomers, THOA) or downregulation [HOJA sulfate, (−)-epicatechin, and (+)-catechin], as found earlier by means of untargeted HR-LC-ToF-MS (see part 1),^22^ could be confirmed in G samples upon comparison with non-G samples of the same origin. Widening the scope of samples highlighted the observed deviations between samples of different proveniences. Although the expected up/downregulation of the postulated marker compounds, which was predicted by the nontargeted approach, based on a small sample set, could not be confirmed for all marker candidates, it was possible to determine HMG gluc B isomers 2 and 3 and HMG gluc A isomer 1 as the most promising KPIs. On the one hand, these findings can help improve attempts to replace traditional cocoa fermentation by more reliable and controlled processes. On the other hand, these results can help to retrieve deeper insights into the physiological changes during the germination of seeds. While jasmonic acid derivatives have already been discussed as phytohormones induced by stress, the role of HMG gluc within the plant has not yet been revealed. Further research is needed to fully understand these compounds and their influence on plant germination.

## Supporting Information Available

Overview of sample materials and preparation of calibration solutions; tuning parameters and mass transitions of all analytes; results of quantification of all analytes including plots of calculated analyte ratios for selected samples; results of data analysis including unsupervised clustering and logistic regression, visualization by scatter plots, and confusion matrices; processed MRM-chromatograms of selected calibration and sample runs, measured at the Waters and the Sciex systems; and high-resolution mass spectra of standard compounds used for quantification: MS^e^ and QToF-MS (MS^2^) (PDF)

## Abbreviations

ACN: acetonitrile
AF: alternatively fermented
Cat: (+)-catechin
*c*: mass concentration
DB: dried cocoa beans
DMSO: dimethyl sulfoxide
EC: (−)-epicatechin
ESI: electrospray ionization
F: fermented
FA: formic acid
FG: fermented and germinated combined
G: germinated
GC: gas chromatography
Gluc: glucoside
GPC: gel permeation chromatography
HDBSCAN: hierarchical density-based spatial cluster of applications with noise
HILIC: hydrophilic interaction chromatography
HMG: 3,3-hydroxymethyl glutaryl/glutarate
HOJA: 12-hydroxyjasmonic acid
HPLC: high-performance liquid chromatography
IS: internal standard
KPI: key process indicator
krpm: kilo rotations per minute
LA: Latin American
Liq: cocoa liquor
LOD: limit of detection
LOQ: limit of quantification
MeOH: methanol
MF: microfermented
MRM: multiple reaction monitoring (mode)
MS: mass spectrometry
MS^e^: pseudo MS^2^ scan using high collision energy
MS/MS: tandem mass spectrometry
NMR: nuclear magnetic resonance spectroscopy
OPLS-DA: orthogonal partial least-squares discriminant analysis
PCA: principal component analysis
qNMR: quantitative NMR
R: recovery
SEA: Southeast Asia
SPE: solid-phase extraction
THOA: trihydroxy octadecenoic acid
ToF-MS: time-of-flight mass spectrometry
UHPLC: ultrahigh-performance liquid chromatography
v/v: volume fraction
μm: micrometer
μL: microliter
ω: mass fraction
