# Supplementary material for: Molecular Changes during Germination of Cocoa Beans, Part 2
Source: J Agric Food Chem. 2024 Jul 29;72(31):17524–35. doi: 10.1021/acs.jafc.4c03524 (PMC11311213; doi:10.1021/acs.jafc.4c03524)
Supplement: Supplementary file 1 — jf4c03524_si_001.pdf [file jf4c03524_si_001.pdf]

Supporting Information:

Molecular changes during germination of cocoa beans, Part 2

Konrad Brückel[†], Timo D. Stark[*†], Corinna Dawid[†] and Thomas Hofmann[†]

[†] Food Chemistry and Molecular Sensory Science, TUM School of Life Sciences, Technical University of Munich

Lise-Meitner-Straße 34
85354 Freising,

Germany

[*]Corresponding author

Materials and methods:

Table S 1: Samples used for Profiling (Batch 1) and additional samples (Batch 2-13) with their respective identifiers (Abbreviation in plots, Sample No.) and description, as provided by food industry.

Batch	Abbreviation in Plots	Sample No.(#)	Sample Description
1	raw Liq, SEA Type 1a	10	Cocoa Liquor from unfermented non-germinated South East Asian Type 1a beans
	G Liq, SEA Type 1a	8	Cocoa Liquor from unfermented germinated South East Asian Type 1a beans
	raw Liq, SEA Type 1b	11	Cocoa Liquor from unfermented non-germinated South East Asian Type 1b beans
	G Liq, SEA Type 1b	9	Cocoa Liquor from unfermented germinated South East Asian Type 1b beans
	F Liq, SEA Type 1	13	Cocoa Liquor from fermented non-germinated South East Asian Type 1 beans
	F G Liq, SEA Type 1	12	Cocoa Liquor from fermented germinated South East Asian Type 1 beans
	F Liq, LA Type 1a	15	Cocoa Liquor from low-fermented Latin American Type 1 beans
	F Liq, LA Type 1b	14	Cocoa Liquor from high-fermented Latin American Type 1 beans
2	raw DB, SEA Type 1a	22	Cocoa Beans from unfermented non-germinated South East Asian Type 1a beans
	G DB, SEA Type 1a	23	Cocoa Beans from unfermented germinated South East Asian Type 1a beans
	raw DB, SEA Type 1b	20	Cocoa Beans from unfermented non-germinated South East Asian Type 1b beans
	G DB, SEA Type 1b	21	Cocoa Beans from unfermented germinated South East Asian Type 1b beans
	F DB, SEA Type 1	16	Cocoa Beans from fermented non-germinated South East Asian Type 1 beans
	F G DB, SEA Type 1	17	Cocoa Beans from fermented germinated South East Asian Type 1 beans
	F DB, LA Type 1a	18	Cocoa Beans from low-fermented Latin American Type 1 beans
	F DB, LA Type 1b	19	Cocoa Beans from high-fermented Latin American Type 1 beans
3	F Liq, LA Type 1a	40	Cocoa liquor from fermented Latin Amercian Type 1 beans
	F Liq, LA Type 1b	41	Cocoa liquor from fermented Latin Amercian Type 1 beans
	F Liq, LA Type 1c	42	Cocoa liquor from fermented Latin Amercian Type 1 beans
	F Liq, WA Type 2a	43	Cocoa liquor from fermented West African Type 2 beans
	F Liq, WA Type 2b	44	Cocoa liquor from fermented West African Type 2 beans
	F Liq, WA Type 2c	45	Cocoa liquor from fermented West African Type 2 beans
4	F Nibs U, LA Type 1a	28	Fermented unroasted nibs from Latin American Type 1 beans
	F Nibs U, LA Type 1b	29	Fermented unroasted nibs from Latin American Type 1 beans
	F Nibs U, LA Type 1c	30	Fermented unroasted nibs from Latin American Type 1 beans
	F Nibs U, WA Type 2a	31	Fermented unroasted nibs from West African Type 2 beans
	F Nibs U, WA Type 2b	32	Fermented unroasted nibs from West African Type 2 beans
	F Nibs U, WA Type 2c	33	Fermented unroasted nibs from West African Type 2 beans
5	F Liq, WA Type 2	50	Cocoa Liquor from fermented West African Type 2 beans
	F Liq, LA Type 1	60	Cocoa liquor from fermented Latin American Type 1 beans
	F Liq, LA Type 1	62	Cocoa Liquor from fermented Latin American Type 1 beans
	raw Liq, LA Type 1	64	Cocoa Liquor from raw Latin American Type 1 beans
	F Liq, SEA Type 1	72	Cocoa Liquor from fermented South East Asian Type 1 beans
	F Liq, SEA Type 1	74	Cocoa Liquor from fermented South East Asian Type 1 beans
	raw Liq, SEA Type 1	76	Cocoa Liquor from raw South East Asian Type 1 beans
	raw Liq, LA Type 3	78	Cocoa liquor from raw Latin American Type 3 beans
6	F DB U, WA Type 2	49	Fermented dried West Afrian Type 2 beans
	F Nibs U, LA Type 1	58	Fermented unroasted nibs from Latin American Type 1 beans
	F Nibs U, LA Type 1	61	Fermented unroasted nibs from Latin American Type 1 beans
	raw Nibs, LA Type 1	63	Raw unroasted nibs from Latin American Type 1 beans
	F Nibs U, SEA Type 1	71	Fermented unroasted nibs from South East Asian Type 1 beans
	F Nibs U, SEA Type 1	73	Fermented unroasted nibs from Sout East Asia Type 1 beans
	raw Nibs, SEA Type 1	75	Raw unroasted nibs from Sout East Asia Type 1 beans
	raw DB, LA Type 3	77	Cocoa liquor from raw Latin American Type 3 beans
	raw DB, LA Type 3_2	79	Cocoa liquor from raw Latin American Type 3 beans

Batch	Abbreviation in Plots	Sample No.(#)	Sample Description
7	F Liq HR, LA Type 1	88	Cocoa liquor from fermented (96 h), high-roasted Latin American Type 1 beans
	F Liq R, LA Type 1	89	Cocoa liquor from fermented (96 h), low-roasted Latin American Type 1 beans
	F Liq U, LA Type 1	90	Cocoa liquor from fermented (96 h), unroasted Latin American Type 1 beans
	raw Liq HR, LA Type 1	91	Cocoa liquor from fermented (0 h), high-roasted Latin American Type 1 beans
	raw Liq R, LA Type 1	92	Cocoa liquor from fermented (0 h), low-roasted Latin American Type 1 beans
	raw Liq U, LA Type 1	93	Cocoa liquor from fermented (0 h), unroasted Latin American Type 1 beans
8	AF Liq HR, LA Type 4	94	Cocoa liquor from alternatively fermented (96 h) beans, high-roasted Latin American Type 4 beans
	AF Liq U, LA Type 4	95	Cocoa liquor from alternatively fermented (96 h) beans, unroasted Latin American Type 4 beans
	raw Liq HR, LA Type 4	96	Cocoa liquor from alternatively fermented (0 h) beans, high-roasted Latin American Type 4 beans
	raw Liq U, LA Type 4	97	Cocoa liquor from alternatively fermented (0 h) beans, unroasted Latin American Type 4 beans
9	F Liq HR, LA Type 5	98	Cocoa liquor from fermented (96 h), high-roasted Latin American Type 5 beans
	F Liq U, LA Type 5	99	Cocoa liquor from fermented (96 h), unroasted Latin American Type 5 beans
	raw Liq HR, LA Type 5	100	Cocoa liquor from fermented (0 h), high-roasted Latin American Type 5 beans
	raw Liq U, LA Type 5	101	Cocoa liquor from fermented (0 h), unroasted Latin American Type 5 beans
10	MF WCB Liq R, LA Type 1	102	Cocoa liquor from winnowed, micro-fermented (96 h), low-roasted Latin American Type 1 Beans
	MF WCB Liq U, LA Type 1	103	Cocoa liquor from winnowed, micro-fermented (96 h), unroasted Latin American Type 1 Beans
	raw WCB Liq R, LA Type 1	104	Cocoa liquor from winnowed, micro-fermented (0 h), low-roasted Latin American Type 1 Beans
	raw WCB Liq U, LA Type 1	105	Cocoa liquor from winnowed, micro-fermented (0 h), unroasted Latin American Type 1 Beans
	AF WCB Liq R, LA Type 1	106	Cocoa liquor from winnowed, alternatively fermented (96 h), low-roasted Latin American Type 1 Beans
	AF WCB Liq U, LA Type 1	107	Cocoa liquor from winnowed, alternatively fermented (96 h), unroasted Latin American Type 1 Beans
	raw WCB Liq R, LA Type 1	108	Cocoa liquor from winnowed, alternatively fermented (0 h), low-roasted Latin American Type 1 Beans
	raw WCB Liq U, LA Type 1	109	Cocoa liquor from winnowed, alternatively fermented (0 h), unroasted Latin American Type 1 Beans
11	raw Choc U, LA Type 1	80	Chocolate from raw unroasted beans, LA Type 1
	raw Choc R, LA Type 1	81	Chocolate from raw roasted beans, LA Type 1
	F Choc U, LA Type 1	82	Chocolate from fermented unroasted beans, LA Type 1
	F Choc R, LA Type 1	83	Chocolate from fermented roasted beans, LA Type 1
12	raw DB U, LA Type 1	84	Unfermented dried Latin American Type 1 beans
	AF DB U, LA Type 1	85	Alternatively fermented dried Latin American Type 1 beans
	raw Cocoa Liq, LA Type 1	86	Cocoa Liquor from unfermented Latin American Type 1 beans
	AF Cocoa Liq, LA Type 1	87	Cocoa Liquor from alternatively fermented Latin American Type 1 beans
13	Choc #1	1	Commercial Chocolate sample
	Choc #2	2	Commercial Chocolate sample
	Choc #3	3	Commercial Chocolate sample
	Choc #4	4	Commercial Chocolate sample
	Choc #5	5	Commercial Chocolate sample
	Choc #6	6	Commercial Chocolate sample
	Liq Choc #6	7	Commercial Cocoa liquor sample

Calibration solutions:

For calibration of the marker compounds at the Waters Xevo TQ-S system (Waters, Bedford, MA, USA), a stock solution was prepared using standard solutions quantified by qNMR with concentrations and volumes according to Table S2.

Table S 2: Preparation of calibration stock solution (Marker quantification at Waters Xevo TQ-S system)

Compound	Concentration standard solution [µg/ml]	Volume used for calibration stock solution [µl][c]	Concentration in stock solution [µg/ml]	Concentration in highest calibration point [µg/ml][d]
HMG gluc C[a]	61.70	125	7.71	3.86
HMG gluc A[a]	2028	250	507	254
HOJA sulfate[a]	2891	50	145	72.3
9,12,13-(10E)-THOA[b]	200.0	125	25.0	12.5
Epicatechin[b]	1100	50	55.0	27.5
Catechin[b]	800.0	50	40.0	20.0
HOJA[a]	832.9	50	41.6	20.8

a) Concentration determined by qNMR, dissolved in d_6-DMSO, diluted in Acetonitrile/water 7/3 (V/V)
b) Concentration of commercial standard compound dissolved in Methanol and diluted in Acetonitrile/water 7/3 (V/V) with respect to purity (without qNMR determination)
c) Volume of standard solution mixed in volumetric flask (1.0 ml), filled up to mark with Acetonitrile/water 7/3 (V/V): stock solution
d) Concentration after mixing with internal standard mix (1/1; V/V)

For calibration at the AB Sciex 6500 system, these calibration solutions were mixed each with the corresponding HMG glucoside solutions 1:1 (v/v), which were prepared according to Table S3 using standard solutions quantified by qNMR.

Residues of HOJA educt in the synthesized HOJA sulfate standard were quantified using an external HOJA-calibration and respected in the overall HOJA concentration of the calibration mix.

Table S3: Preparation of calibration stock solution (HMG gluc quantification at Sciex 6500 system)

Compound	Concentration in qNMR solution [µg/mL]	Volume [µl] used for Stock solution	Volume [µl] after dilution (1/100; V/V) used for Stock solution	Concentration in Stock solution [µg/ml]	Concentration in highest calibration point [µg/ml][c]
HMG gluc G[a]	1447		282	4.08	2.04
HMG gluc F[a]	2390		70.2	1.68	0.84
HMG gluc H[a]	1167	23		26.8	13.4
HMG gluc K[a]	1774		48.4	0.86	0.43
HMG gluc D[a]	3451		117.4	4.05	2.03
HMG gluc J[a]	2438		43.4	1.06	0.53
HMG gluc M[b]	1428		68.8	0.98	0.49
HMG gluc O[b]	1721		57.8	0.99	0.50
HMG gluc E[a]	2558	8.68		22.2	11.1
HMG gluc L[b]	1284		78	1.00	0.50
HMG gluc N[a]	2479		34.2	0.85	0.42

a) qNMR meassured in d_6-DMSO

b) qNMR meassured in d_4-MeOD

c) Concentration after mixing Stock solution (HMG gluc standards) with highest calibration point of Marker calibration (1/1; V/V)

Method development

Previous profiling experiments had revealed several compounds in cocoa, which were affected by germination more than others.[22] Hereby, germinated samples had been compared with their raw equivalents by a workflow comprising principal component analysis, S-plot analysis and subsequent identification. Moreover, the detection and identification of several further HMG glucosides, which had mostly not been described in cocoa before, suggested that this second set of compounds might contain useful markers as well. Therefore, a targeted approach via UHPLC-MS/MS was chosen to evaluate the ability of these two sets of compounds as possible markers, unique to the cocoa process applied.

First, the previously defined marker candidates as well as the newly discovered HMG glucosides were tuned at the Waters system (XEVO TQ-S), and four mass transitions per compound were automatically generated by the instrument. Of these, the two most abundant transitions were used as quantifier and qualifier after confirmation that no

interference was observed during analysis of a set of test samples. Figure S 1 illustrates the chromatographic separation of the marker compounds found in the profiling by depicting the chromatograms of chosen MRM transitions. Compounds 1 and 4 ID were not confirmed with standard compounds; thus, they were not included in the final quantification method. Optimization of the binary gradient yielded several separated peaks per compound for the detected HMG glucosides A, B, and C. These peaks were assigned to stereoisomers of their respective standards. The different isomers were given numbers according to their order of elution (e.g., HMG gluc B isomer 1–3). HMG gluc A isomer 1 and HMG gluc C isomer 1 could be identified by coelution with standard compounds (see Figures S 35 f and S 45 ff). HMG gluc A isomer 2 could be additionally confirmed by ToF-MS due to a similar fragmentation as isomer 1, while both HMG gluc C isomers could not be detected by ToF-MS. HMG glucoside B and its isomers could not be obtained as pure standard compounds; therefore, tuning of these was performed using the isolate gained after HILIC fractionation followed by a solid-phase extraction (described by Brückel et al.).[22] For quantification, the HMG glucoside A standard, featuring highest structural similarity among the HMG glucoside standards available, was used as an external standard, whereas HMG glucoside I was used as an internal standard due to its high structural similarity and its absence in the test samples. As the current method performances at the Waters system did not allow detection of HMG glucosides F, G, J, K, L, M, N and O, all HMG glucosides were tuned at the Sciex system with higher performances as shown in Tables S 4 and S 5. As depicted, the supposed markers, i.e., HOJA sulfate, HOJA, THOAs, (+)-catechin, (−)-epicatechin, and HMG gluc A, B, and C and their respective isomers, were measured at the Waters system using hexyl sulfate as an internal standard for HOJA sulfate and HMG gluc I as an internal standard for HMG glucosides, HOJA, THOAs, and (+)-catechin. (−)-Epicatechin was quantified via external calibration due to the

higher concentration. The concentration range of the method was defined for each analyte to cover the concentration measured in raw, germinated and fermented bean and liquor samples.

Furthermore, the equilibration time after addition of the internal standards was determined by several work-ups with equilibration times of 1 h, 3 h, 6 h, and 12 h. These experiments revealed that equilibration times higher than 1 h were not required, as the concentrations measured after equilibration at room temperature, grinding, and defatting were not increased after longer equilibration. Thus, equilibration and extraction were considered as completed after 1 h of equilibration followed by cooling down to −18°C and grinding in the bead beater.

Tuning parameters:

Table S 4: Tuning parameters at Waters Xevo TQS system

Analyte	M-H *(m/z)*	Quant. *(m/z)*	Qual. *(m/z)*	CV (V)	CE Quant. (V)	CE Qual. (V)	IS
HOJA	225.0	59.0	97.1	36	20	24	HMG gluc I
HOJA sulfate	304.9	96.9	225.2	36	22	20	hexyl sulfate
HMG gluc A	393.1	99.1	291.3	18	14	10	HMG gluc I
HMG gluc B	395.0	99.1	125.1	34	14	16	HMG gluc I
HMG gluc C	364.9	99.0	125.1	2	14	14	HMG gluc I
9,10,13-(11E)-THOA	329.1	171.1	229.2	22	24	21	HMG gluc I
9,12,13-(10E)-THOA	329.1	211.2	229.2	22	22	21	HMG gluc I
Cat	289.1	245.0	203.0	48	14	20	
EC	289.1	245.0	109.0	48	14	24	
HMG gluc I	515.1	99.1	125.1	12	14	18	
hexyl sulfate	180.9	96.9	80	38	18	32	

12-Hydroxyjasmonic acid (HOJA), 12-hydroxyjasmonic acid sulfate (HOJA sulfate), hydroxymethyl glutaroyl glucoside A (HMG gluc A), hydroxymethyl glutaroyl glucoside B (HMG gluc B), hydroxymethyl glutaroyl glucoside C (HMG gluc C), 9,10,13-(11E)-trihydroxy octadecenoic acid (9,10,13,(11E)-THOA, 9,12,13-(10E)-trihydroxy octadecenoic acid (9,12,13,(10E)-THOA with their respective internal standard (IS) hydroxymethyl glutaroyl glucoside I (HMG gluc I) and hexyl sulfate; , catechin (Cat) and epicatechin (EC) were quantified by external calibration; mother ion (M-H), and daughter ions used as quantifier (Quant.) and qualifier (Qual.) with their respective mass/charge ratios m/z and respective tuning parameters Cone Voltage (CV) and Collision Energy (CE).

Table S 5: Tuning parameters at AB Sciex 6500 system

Analyte	M-H *(m/z)*	Quant./Qual. *(m/z)*	Dwell time (ms)	DP (V)	CE (V)	CXP (V)
HMG gluc C Isomer 1[a]	365.1	220.9[c]	8	-50	-26	-25
		98.8[d]	8	-50	-20	-11
HMG gluc D	430.9	124.9[c]	8	-35	-24	-15
		98.9[d]	8	-35	-22	-11
HMG gluc E	413.0	268.9[c]	8	-15	-24	-25
		99.0[d]	8	-15	-22	-7
HMG gluc F	435.1	291.0[c]	8	-45	-26	-35
		98.8[d]	8	-45	-22	-11
HMG gluc G	427.0	99.0[c]	8	-35	-20	-11
		142.9[d]	8	-35	-16	-9
HMG gluc H	351.0	207.0[c]	8	-5	-26	-31
		98.8[d]	8	-5	-18	-13
HMG gluc I[b]	515.0	208.9[c]	6	-40	-24	-27
		176.9[d]	6	-40	-38	-13
HMG gluc J	471.0	326.9[c]	8	-35	-28	-43
		369.1[d]	8	-35	-22	-39
HMG gluc K	515.1	371.1[c]	8	-70	-28	-35
		413.0[d]	8	-70	-20	-47
HMG gluc L	637.0	329.7[c]	8	-90	-50	-35
		492.9[d]	8	-90	-36	-27
HMG gluc M	651.1	329.4[c]	8	-95	-60	-47
		344.5[d]	8	-95	-42	-31
HMG gluc N	529.1	385.0[c]	8	-75	-26	-49
		367.0[d]	8	-75	-30	-11
HMG gluc O	575.1	431.0[c]	8	-40	-36	-49
		473.0[d]	8	-40	-30	-17
HMG gluc A Isomer 1[a]	393.2	98.7[c]	8	-80	-24	-5
		248.9[d]	8	-80	-26	-7

[a] several isomers found, isomers 1 have identical retention time with standard compound

[b] compound used as internal standard

[c] daughter ion used for quantification (quantifier)

[d] daughter ion used for identification (qualifier)

Hydroxymethyl glutaroyl glucosides (=HMG gluc) A, C, D, E, F, G, H, J, K, L, M, N and O were quantified with HMG gluc I as an internal standard (IS); mother ion (M-H), and daughter ions used as quantifier (Quant.) and qualifier (Qual.) with their respective mass/charge ratios *m/z* and respective tuning parameters Dwell Time, Declustering Potential (DP), Collision Energy (CE) and Cell Exit Potential (CXP).

Chromatogram of optimized method:

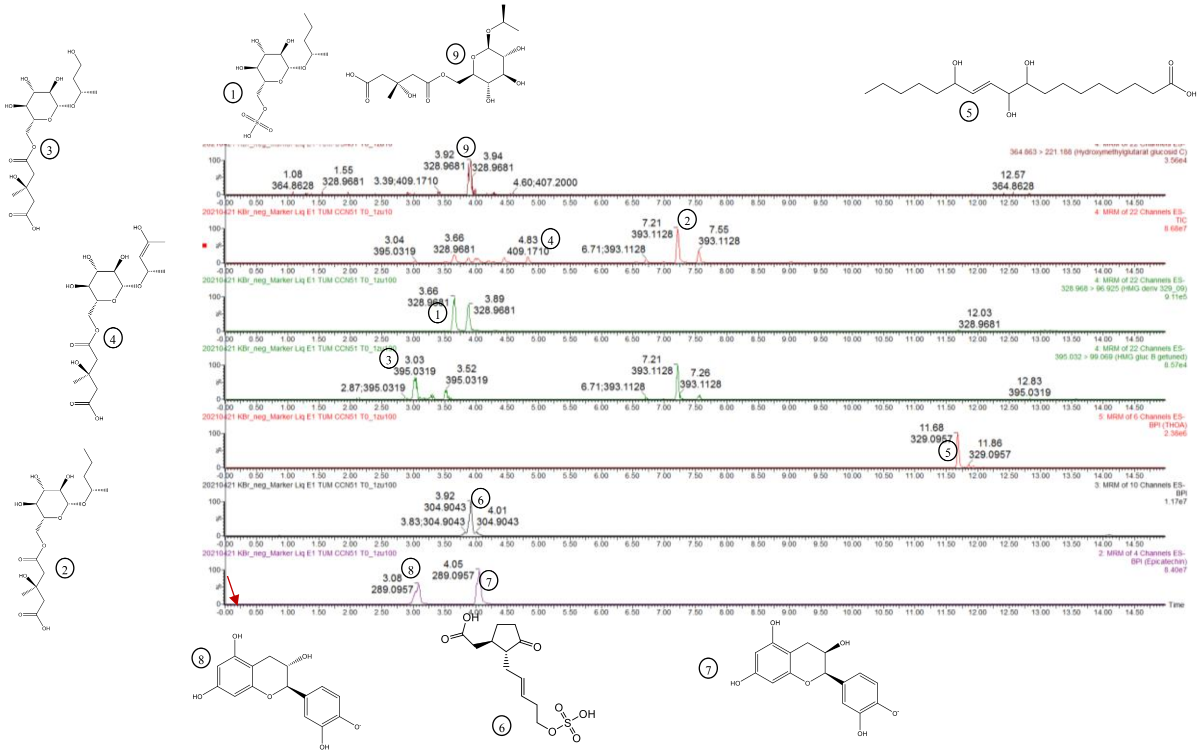

Figure S 1: MRM chromatograms of marker compounds, measured in the extract of raw cocoa bean liquor at the Waters system after compound optimization.

Results of method validation

Sensitivity. To determine the range of the quantification methods, the stock solution of the standard mix was diluted 1:2, 1:10, 1:20, 1:100, and so on until a signal-to-noise ratio of lower three was reached (Table S6). The first concentration of the calibration solution, exceeding a signal-to-noise ratio of 3, was defined as limit of detection of the instrument (LOD), while reaching a signal-to-noise ratio of 10 was defined as a limit of quantification regarding the instrument (LOQ).

Linear range. Linearity was defined as the highest concentration range where a determination coefficient of more than 0.99 could be achieved. Table S6 summarizes the results. For all chosen analytes, linearity was given in a satisfactory range to systematically cover the endogenous concentration in the three types of process.

Table S 6: Linearity range, LOD, and LOQ of the instrument determined by signal-to-noise ratio.

Concentration (ng/ml)		HMG gluc C Isomers	HOJA sulfat	HOJA	HMG gluc A Isomers	Catechin	Epicatechin	THOAs	HMG gluc B Isomers
**linearity range**	**lower limit**	1.93	3.61	10.41	12.68	10.00	13.75	1.25	1.93
	**upper limit**	3855.94	36141.30	20823.68	25351.35	20000.00	2750.00	1250.00	3855.94
	**LOD**	1.93	0.31	1.34	0.12	2.09	0.41	0.017	1.93
	**LOQ**	3.14	1.04	4.48	0.40	6.97	1.38	0.055	3.14

Concentration (ng/ml)		HMG gluc D	HMG gluc E	HMG gluc F	HMG gluc G	HMG gluc H	HMG gluc J
**linearity range**	**lower limit**	2.03	5.55	0.42	2.04	6.71	0.53
	**upper limit**	2025.62	11100.50	838.83	2040.02	13415.72	528.95
	**LOD**	0.61	1.67	0.25	3.06	2.01	0.16
	**LOQ**	2.03	5.55	0.84	10.20	6.71	0.53

Concentration (ng/ml)		HMG gluc K	HMG gluc L	HMG gluc M	HMG gluc N	HMG gluc O
**linearity range**	**lower limit**	0.43	0.50	2.46	2.12	0.50
	**upper limit**	429.34	500.71	491.16	423.97	497.45
	**LOD**	0.64	1.50	1.47	1.27	0.75
	**LOQ**	2.15	5.01	4.91	4.24	2.49

Recovery. Method accuracy was determined through spike and recovery experiments. Table S7 summarizes the results for each analyte. The recovery rates found ranged from about 80% to 120% for most analytes and spiking levels, with some outliers for the higher spiking levels of HMG gluc C isomer 1 (65.1% at the highest level).

Robustness. The standard deviations ranged between 3% and 15% for six intraday repetitions for all but one analyte, which is considered with a sufficiently high reproducibility. However, for HMG gluc M a low signal response close to LOQ was associated with lower repeatability. Given these validation results, the established methods at both MS systems were considered sufficiently accurate and robust in the chosen range. However, in the case of HMG gluc M the results of this compound should be considered rather as semi-quantitative due to the decreased repeatability at endogenous concentrations.

Table S 7: Recovery mean values and standard deviations calculated from six technical replicates after spiking of sample #85 with different equivalents (respective to the natural content) of analytes.

Spiking amount	HOJA sulfat		HOJA		HMG A		9,12,13-(10E)-THOA	
Equivalents	Recovery (%) Mean	Std dev	Recovery (%) Mean	Std dev	Recovery (%) Mean	Std dev	Recovery (%) Mean	Std dev
0.5	**114.5**	9.0	**89.9**	6.2	**71.8**	11.4	**94.9**	11.7
1	**121.1**	9.0	**91.3**	8.7	**83.9**	6.9	**96.5**	6.9
2	**111.0**	3.2	**90.4**	5.0	**89.1**	2.6	**86.2**	3.9
4	**112.3**	7.3	**85.7**	4.5	**91.0**	6.5	**86.1**	4.5

Spiking amount	HMG gluc C Isomer 1		HMG gluc D		HMG gluc E		HMG gluc F	
Equivalents	Recovery (%) Mean	Std dev	Recovery (%) Mean	Std dev	Recovery (%) Mean	Std dev	Recovery (%) Mean	Std dev
0.5	**90.9**	5.8	**102.5**	6.6	**112.1**	3.9	**104.5**	16.0
1	**92.6**	8.2	**100.9**	6.8	**121.9**	7.8	**106.7**	12.5
2	**73.6**	7.6	**104.3**	8.5	**115.6**	9.4	**103.7**	12.2
4	**65.1**	4.0	**104.0**	4.8	**117.5**	4.2	**102.3**	5.1

Spiking amount	HMG gluc G		HMG gluc H		HMG gluc J		HMG gluc K	
Equivalents	Recovery (%) Mean	Std dev	Recovery (%) Mean	Std dev	Recovery (%) Mean	Std dev	Recovery (%) Mean	Std dev
0.5	**114.5**	5.4	**115.0**	5.0	**104.9**	15.9	**106.2**	19.3
1	**125.1**	10.0	**124.8**	8.3	**108.0**	11.6	**98.8**	12.2
2	**125.7**	13.4	**124.6**	13.0	**108.5**	11.5	**97.2**	8.1
4	**124.4**	14.4	**125.7**	5.4	**105.6**	5.3	**98.7**	12.3

Spiking amount	HMG gluc M		HMG gluc N		HMG gluc O	
Equivalents	Recovery (%) Mean	Std dev	Recovery (%) Mean	Std dev	Recovery (%) Mean	Std dev
0.5	**109.6**	70.1	**96.9**	9.2	**105.1**	9.2
1	**88.3**	40.7	**109.3**	12.4	**111.1**	12.8
2	**118.0**	52.3	**110.4**	8.7	**113.0**	9.6
4	**93.5**	33.5	**113.7**	10.8	**114.0**	3.3

Results of Quantification

Comparison of absolute marker concentrations in Profiling samples

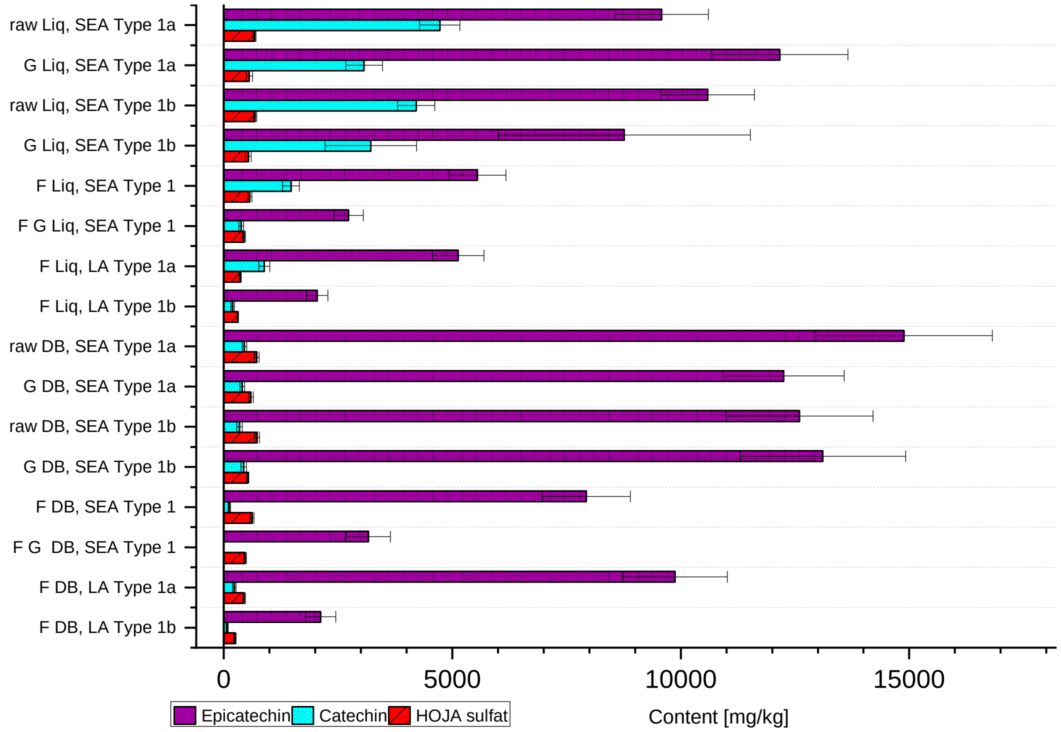

Figure S 2A: Content of marker compounds measured by UPLC-MS/MS; mean values of six replicates of raw, fermented (F) and germinated (G) cocoa liquor (Liq) and dried beans (DB) of Latin American (LA) and South East Asian (SEA) origin.

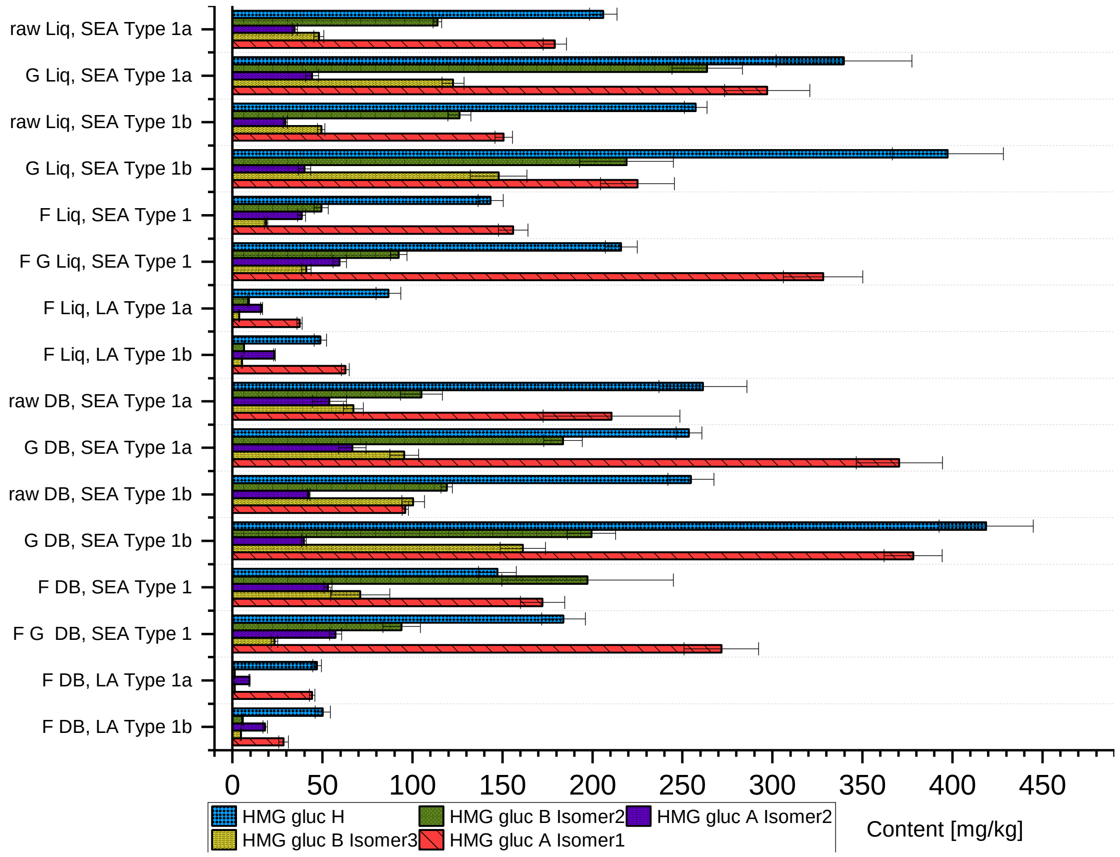

Figure S 2B: Content of marker compounds measured by UPLC-MS/MS; mean values of six replicates of raw, fermented (F) and germinated (G) cocoa liquor (Liq) and dried beans (DB) of Latin American (LA) and South East Asian (SEA) origin.

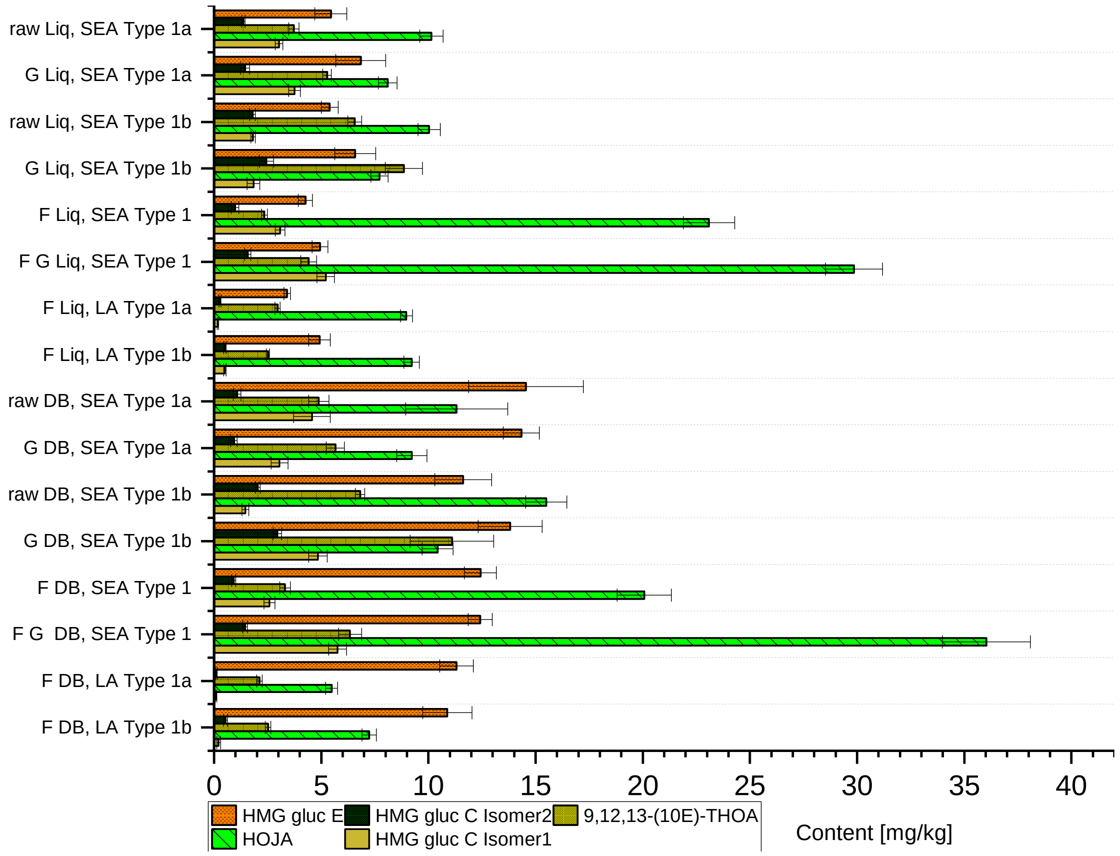

Figure S 2C: Content of marker compounds measured by UPLC-MS/MS; mean values of six replicates of raw, fermented (F) and germinated (G) cocoa liquor (Liq) and dried beans (DB) of Latin American (LA) and South East Asian (SEA) origin.

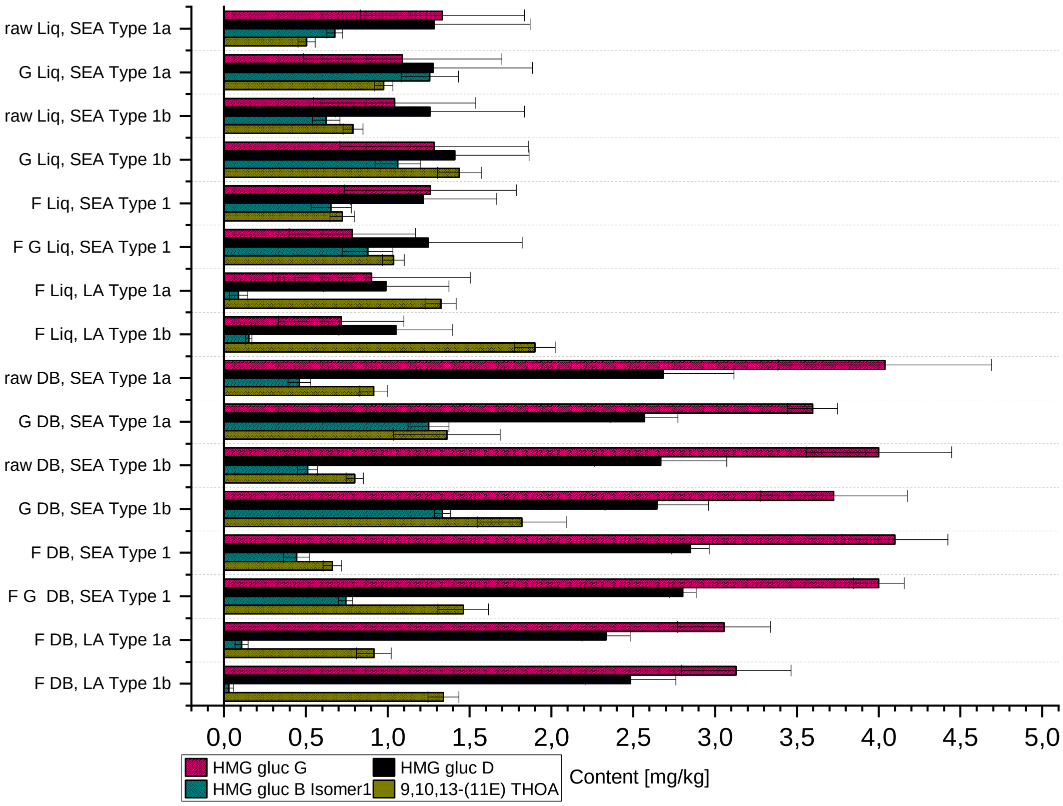

Figure S 2D: Content of marker compounds measured by UPLC-MS/MS; mean values of six replicates of raw, fermented (F) and germinated (G) cocoa liquor (Liq) and dried beans (DB) of Latin American (LA) and South East Asian (SEA) origin.

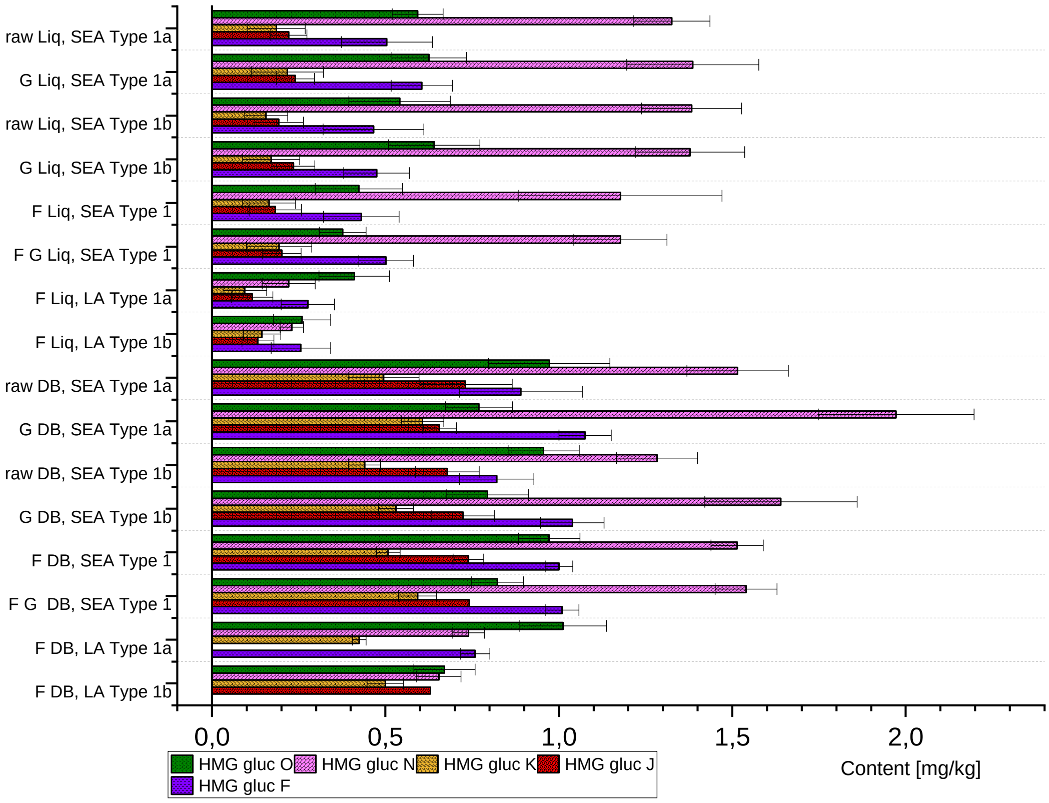

Figure S 2E: Content of marker compounds measured by UPLC-MS/MS; mean values of six replicates of raw, fermented (F) and germinated (G) cocoa liquor (Liq) and dried beans (DB) of Latin American (LA) and South East Asian (SEA) origin..

Figure S2B depicts a group of lower concentrated marker compounds, in which HMG gluc A isomer 1 is apparently found in higher concentration in all germinated samples than in their raw/fermented counterparts of the same provenience. Moreover, HMG gluc B isomers 2 and 3 are found in about twofold higher concentration in the germinated samples than in the corresponding raw samples and in all fermented samples of this batch. This also seems to account for HMG gluc H, which is upregulated in all germinated liquor samples against the non-germinated counterparts.

Figure S2C depicts the next lower concentrated group of marker candidates, where the 9,12,13-trihydroxy-(10*E*)-octadecenoic acid can be found in higher concentration in the germinated samples than in the corresponding raw samples of the same provenience. In contrast, HMG gluc E and HMG gluc C isomers 1 and 2 feature increased concentrations only in some germinated samples, whereas no unequivocal conclusion can be drawn for 12-hydroxyjasmonic acid (HOJA).

Upon comparing the germinated and the corresponding non-germinated samples of even less abundant compounds (Figure S2D), HMG gluc B isomer 1 and 9,10,13-trihydroxy-(11*E*)-octadecenoic acid follow the pattern of their isomers described above, thus confirming the findings of the S-plot. For the remaining compounds measured, i.e., HMG gluc D and G (depicted in Figure S2D) and HMG gluc F, J, K, N, and O (depicted in Figure S2E) the concentrations in germinated and raw materials are within the same magnitude. However, in the fermented samples, especially the Latin American ones, concentrations appear slightly decreased toward those in raw and germinated samples.

The standard deviations determined for (−)-epicatechin (depicted in Figure S2A) indicate a lower robustness of this quantification, which in contrast to the other compounds was solely based on external calibration. In liquor samples, THOA contents varied on a greater scale

between different sample proveniences than between raw, fermented, and germinated samples of the same provenience (Figures S2C and D). However, within one provenience, germinated samples imparted higher contents than fermented or raw samples, which is in accordance with the profiling results. Quantification of THOA markers in cocoa bean samples led to results like those of the corresponding liquors. In summary, targeted measurement of supposed marker candidates by UHPLC-MS/MS could confirm the differences in composition between germinated and non-germinated samples, which were found by the previous non-targeted approach via UHPLC-ToF/MS. Characteristic germination marker candidates as HMG glucosides A and B and their isomers could be found in up to twofold higher concentrations than in their non-germinated corresponding samples. On the other hand, (+)-catechin and 12-hydroxyjasmonic acid sulfate were observed in higher amounts in the corresponding non-germinated material.

Comparison of marker ratios in Profiling samples

Firstly, these ratios were formed for 12-hydroxyjasmonic acid sulfate (HOJA sulfate) for each sample run. Figure S3A–E depicts these ratios for different ranges. Moreover, (+)-catechin and (−)-epicatechin were downregulated by germination; thus, ratios with the also downregulated HOJA sulfate (Figures S3A, S4A, and S5A) would not establish a useful criterion for germination. However, in this respect, ratios of HMG gluc A and B consequently appeared more promising. In particular, the ratio of HMG gluc A isomer 1 with HOJA sulfate (Figure S3B) showed values ranging from about 0.1 to 0.3 for the raw and fermented samples of this set, whereas values range from 0.4 to 0.8 were achieved in almost all germinated samples. Comparable results were found for HMG gluc B isomer 3 (Figure S3B), where this ratio ranged from 0.8 to 1.6 in raw samples with considerably lower values in fermented samples. On the other hand, values from 0.2 to 0.3 were achieved in almost all but one germinated sample in this set. Moreover, HMG gluc C isomer 2 might also be useful when used in ratio to HOJA sulfate (Figure S3C). Furthermore, the fold change of the $\omega_{analyte}/\omega_{HOJA\ sulfate}$ ratio between germinated and non-germinated samples (of the same provenience) increased to a range from two to four for the most potent markers like HMG gluc A isomer 1, HMG gluc B isomers 2 and 3 (Figure S3B), HMG gluc C isomer 1 (Figure S3C), and both THOA isomers (Figures S3C/D), thus highlighting the differences between germinated and non-germinated samples.

Figures S4A–E and S5A–E depict further ratios calculated with (+)-catechin and (−)-epicatechin. The first ones significantly imparted lower values in liquor than in bean samples, even when samples of the same provenience were compared. As a result, it was not possible to define an overall criterion for differentiation of germinated and raw/fermented samples. For bean samples, the $\omega_{HMG\ gluc\ B\ isomer2}/\omega_{Cat}$ ratio was below 0.4 for the raw samples and

above this value for germinated samples (Figure S4B). However, fermented bean samples (SEA type 1, LA type 1a and 1b) spread from below 0.1–1.1, excluding this ratio as criterion for germination. Similar outcomes were retrieved for liquor samples as well as for the $\omega_{analyte}/\omega_{Cat}$ ratios of the remaining promising marker compounds.

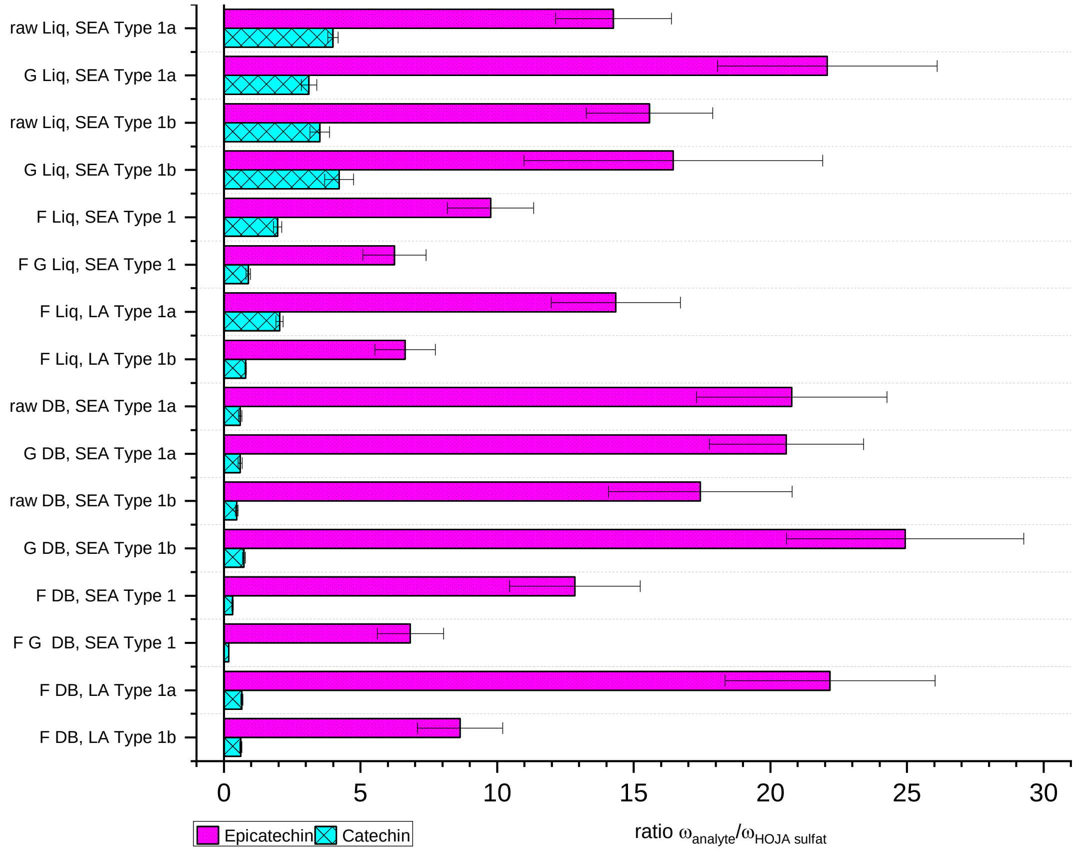

Figure S 3A: Mean values of content ratios of marker compounds $\omega_{analyte}/\omega_{HOJA\ sulfate}$, calculated for six replicates of raw, fermented (F) and germinated (G) cocoa liquor (Liq) and dried beans (DB) of Latin American (LA) and South East Asian (SEA) origin.

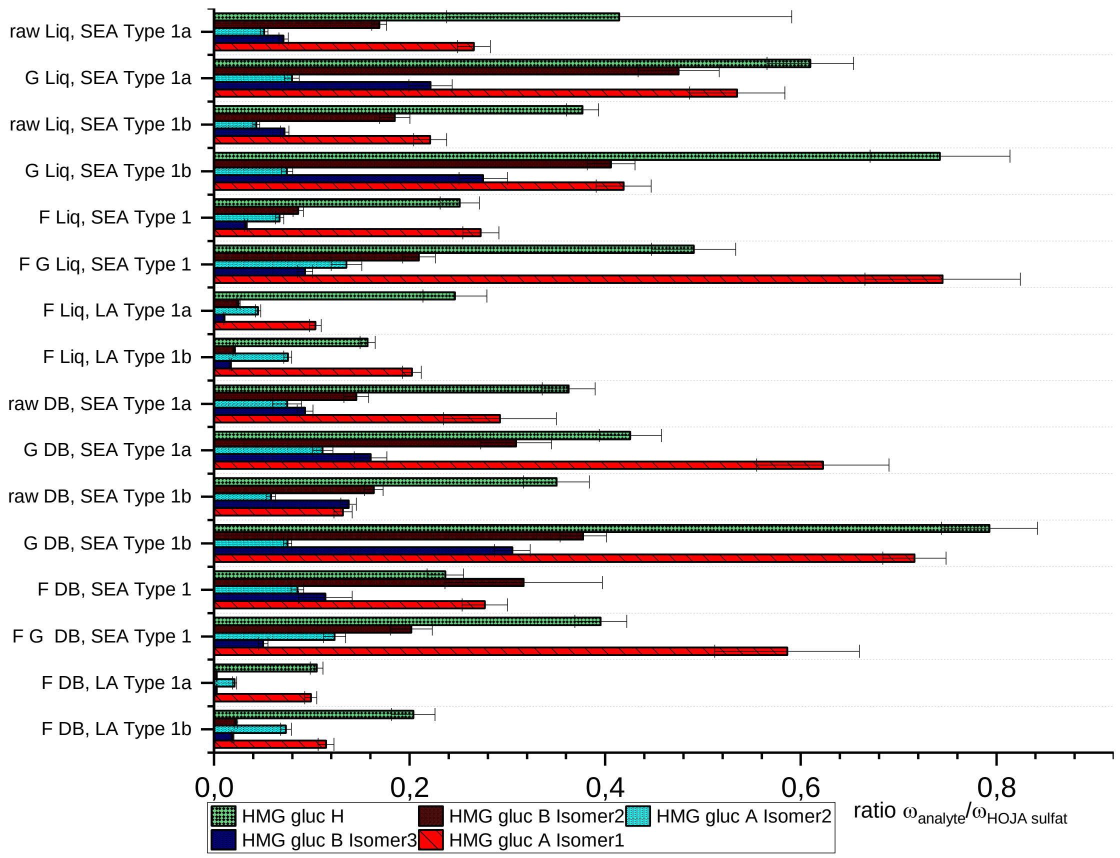

Figure S 3B: Mean values of content ratios of marker compounds $\omega_{analyte}/\omega_{HOJA\ sulfate}$, calculated for six replicates of raw, fermented (F) and germinated (G) cocoa liquor (Liq) and dried beans (DB) of Latin American (LA) and South East Asian (SEA) origin.

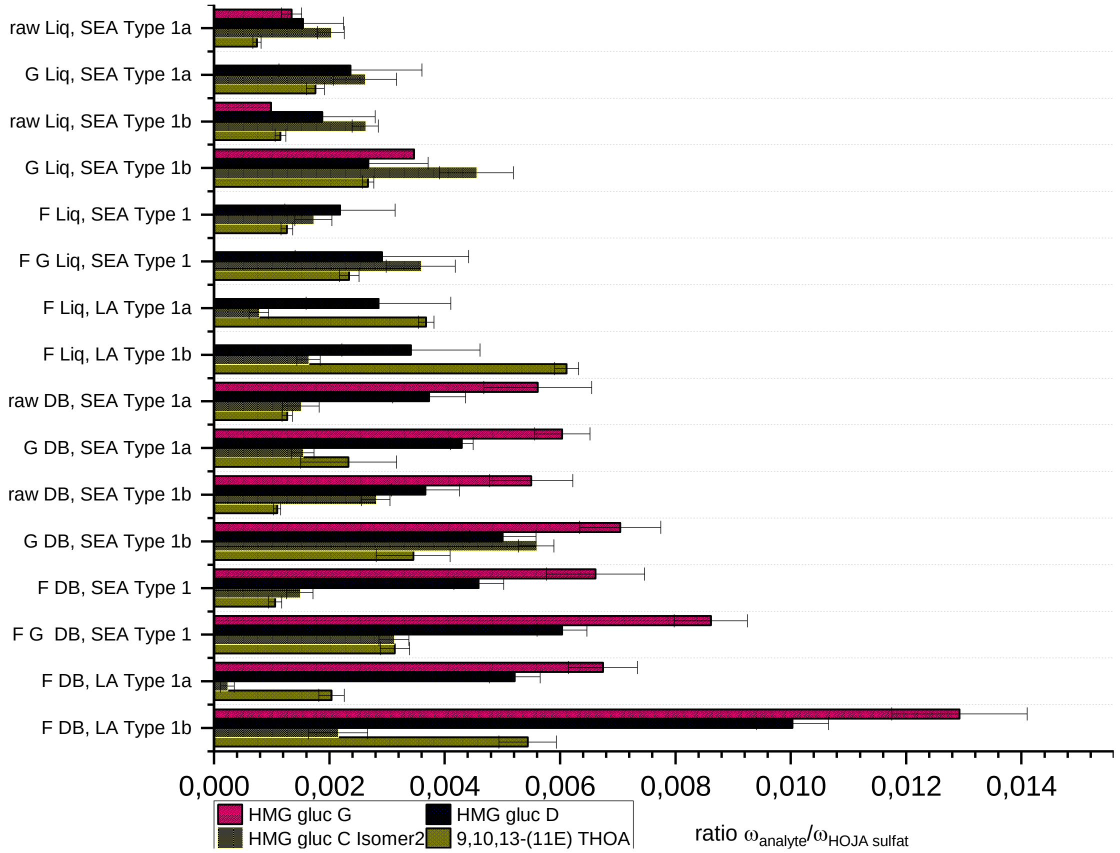

Figure S 3C: Mean values of content ratios of marker compounds $\omega_{analyte}/\omega_{HOJA\ sulfate}$, calculated for six replicates of raw, fermented (F) and germinated (G) cocoa liquor (Liq) and dried beans (DB) of Latin American (LA) and South East Asian (SEA) origin.

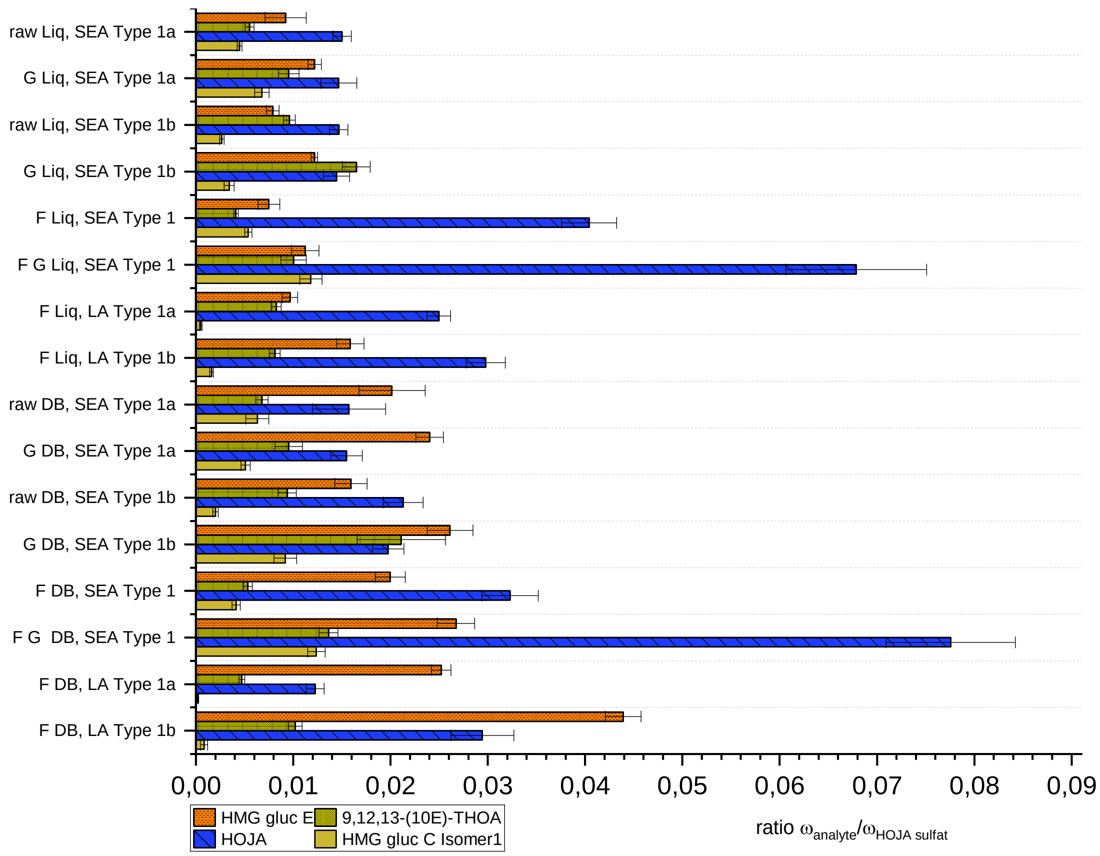

Figure S 3D: Mean values of content ratios of marker compounds $\omega_{analyte}/\omega_{HOJA\ sulfate}$, calculated for six replicates of raw, fermented (F) and germinated (G) cocoa liquor (Liq) and dried beans (DB) of Latin American (LA) and South East Asian (SEA) origin.

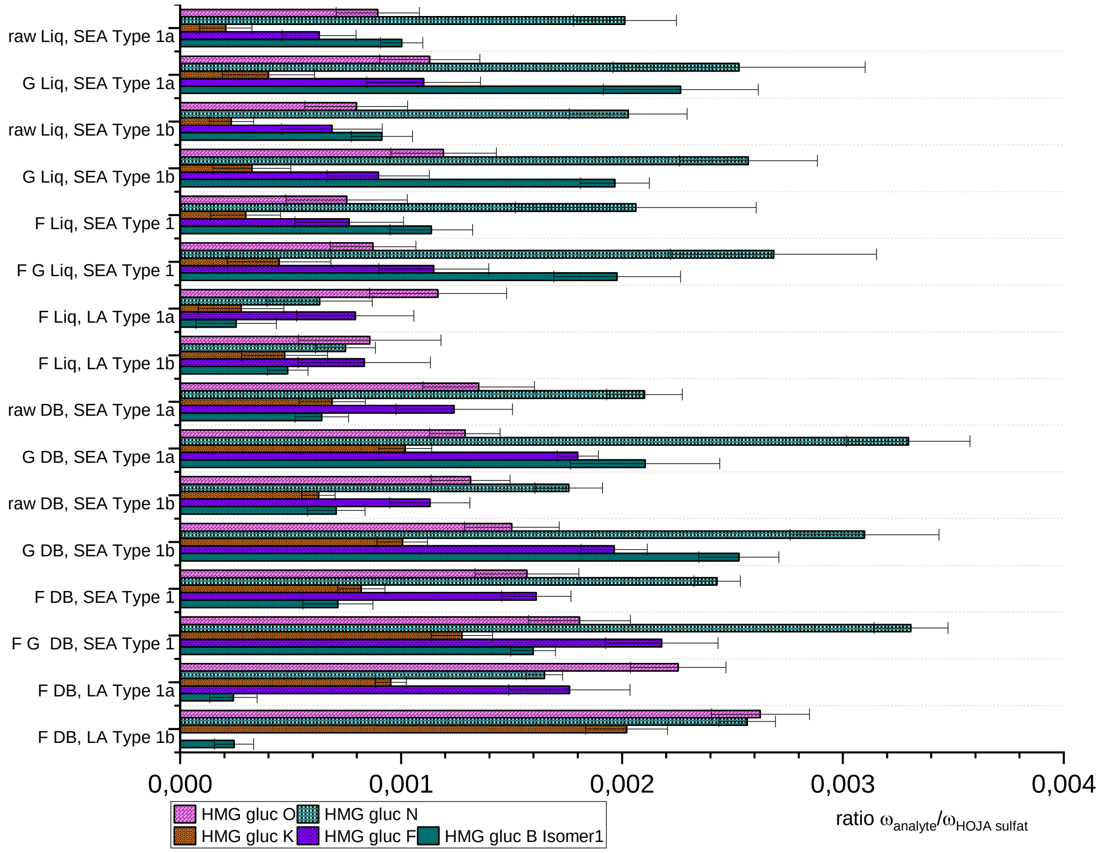

Figure S 3E: Mean values of content ratios of marker compounds $\omega_{analyte}/\omega_{HOJA\ sulfate}$, calculated for six replicates of raw, fermented (F) and germinated (G) cocoa liquor (Liq) and dried beans (DB) of Latin American (LA) and South East Asian (SEA) origin.

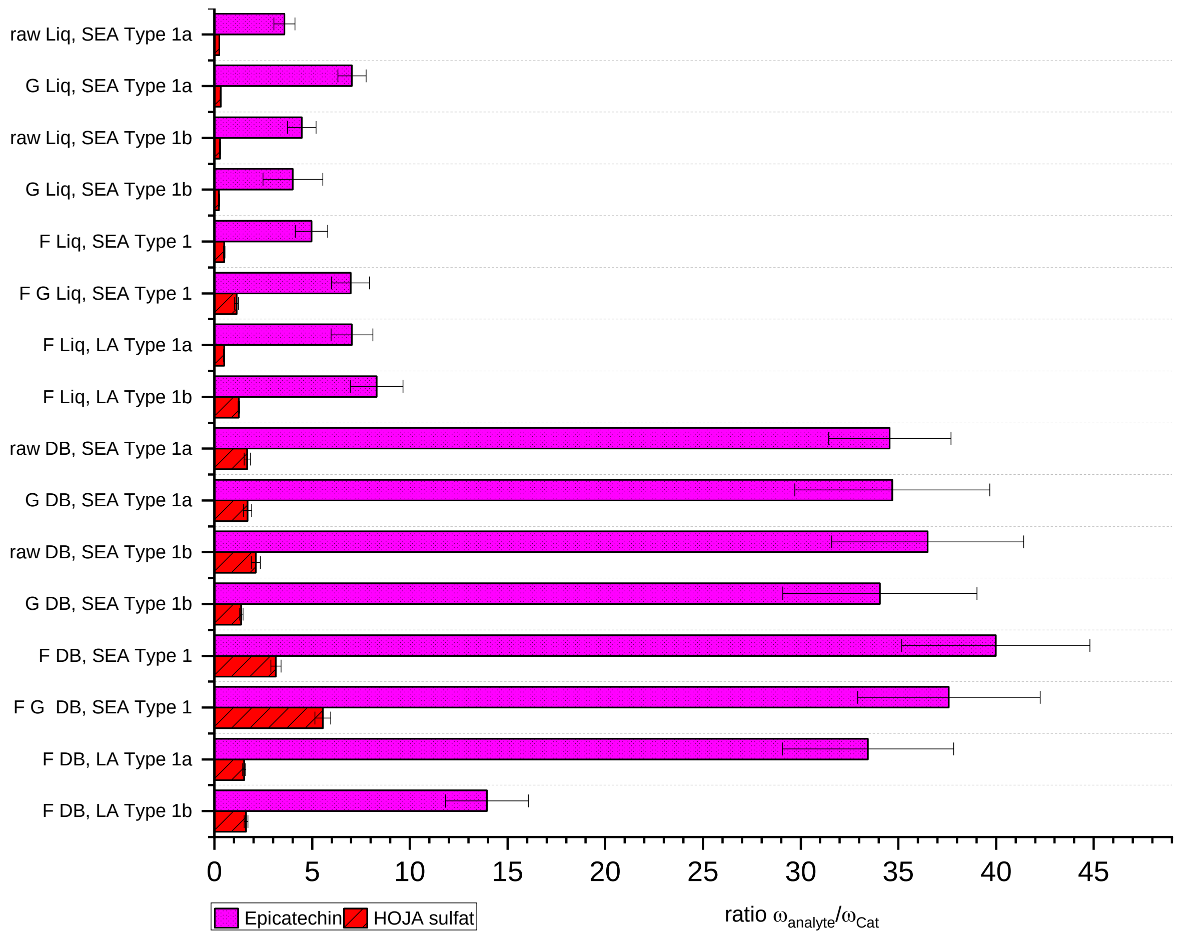

Figure S 4A: Mean values of content ratios of marker compounds $\omega_{analyte}/\omega_{Cat}$, calculated for six replicates of raw, fermented (F) and germinated (G) cocoa liquor (Liq) and dried beans (DB) of Latin American (LA) and Southeast Asian (SEA) origin.

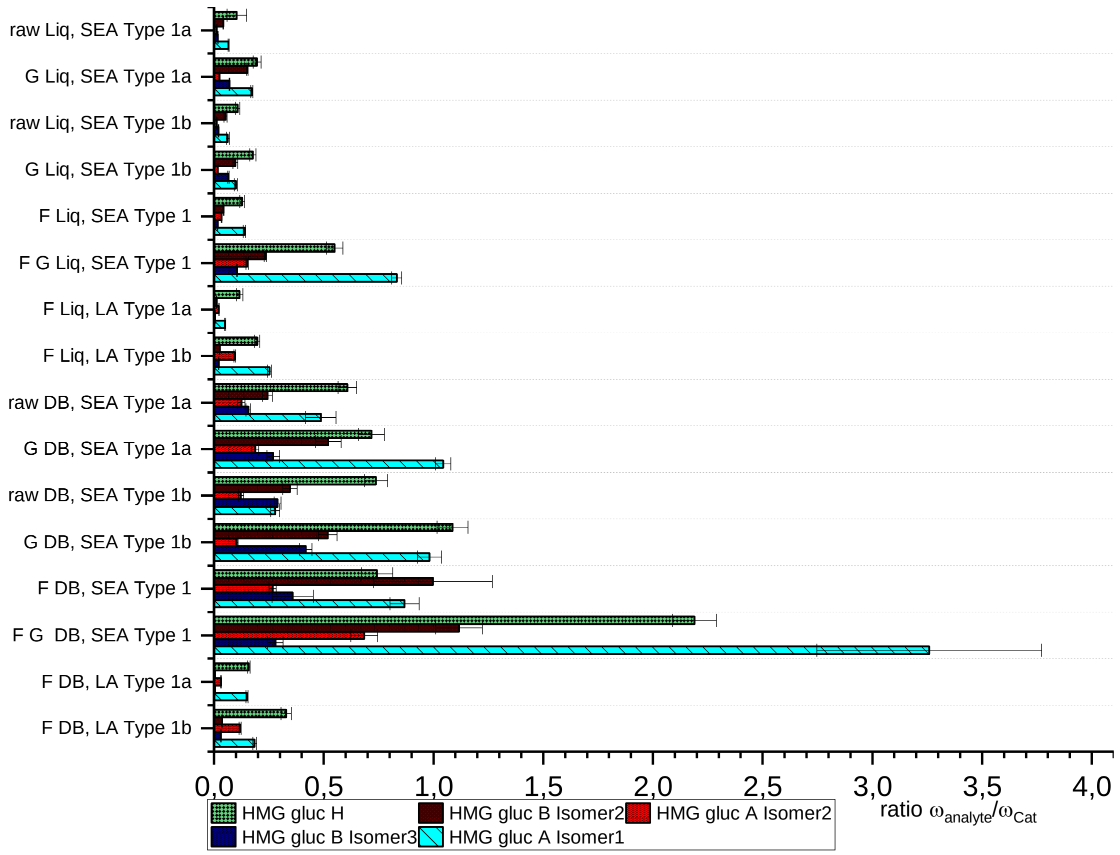

Figure S 4B: Mean values of content ratios of marker compounds $\omega_{analyte}/\omega_{Cat}$, calculated for six replicates of raw, fermented (F) and germinated (G) cocoa liquor (Liq) and dried beans (DB) of Latin American (LA) and Southeast Asian (SEA) origin.

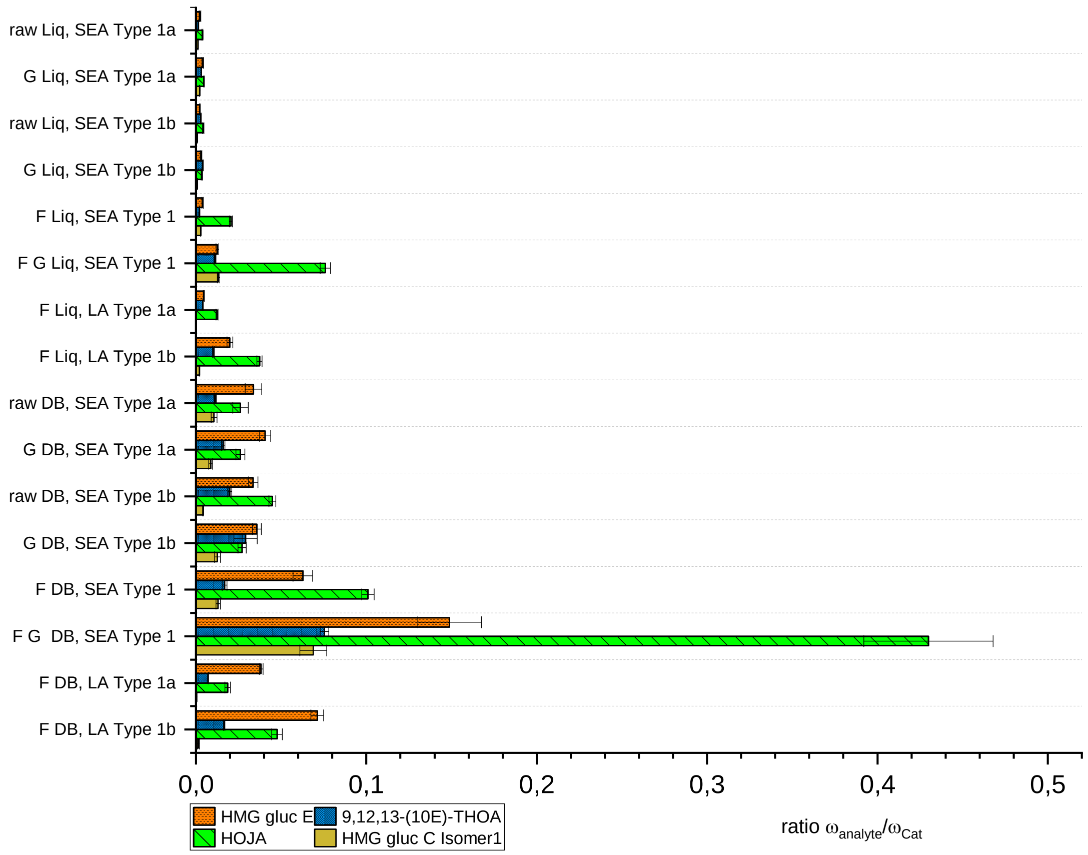

Figure S 4C: Mean values of content ratios of marker compounds $\omega_{analyte}/\omega_{Cat}$, calculated for six replicates of raw, fermented (F) and germinated (G) cocoa liquor (Liq) and dried beans (DB) of Latin American (LA) and Southeast Asian (SEA) origin.

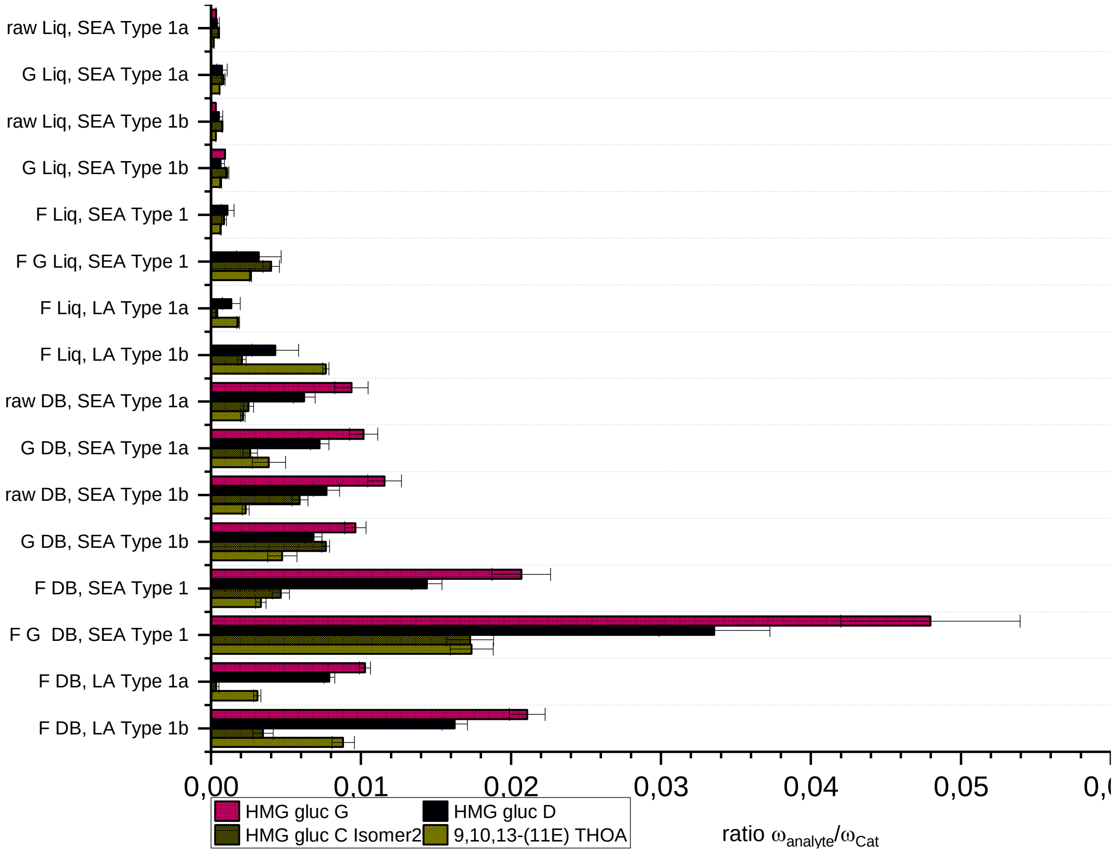

Figure S 4D: Mean values of content ratios of marker compounds $\omega_{analyte}/\omega_{Cat}$, calculated for six replicates of raw, fermented (F) and germinated (G) cocoa liquor (Liq) and dried beans (DB) of Latin American (LA) and Southeast Asian (SEA) origin.

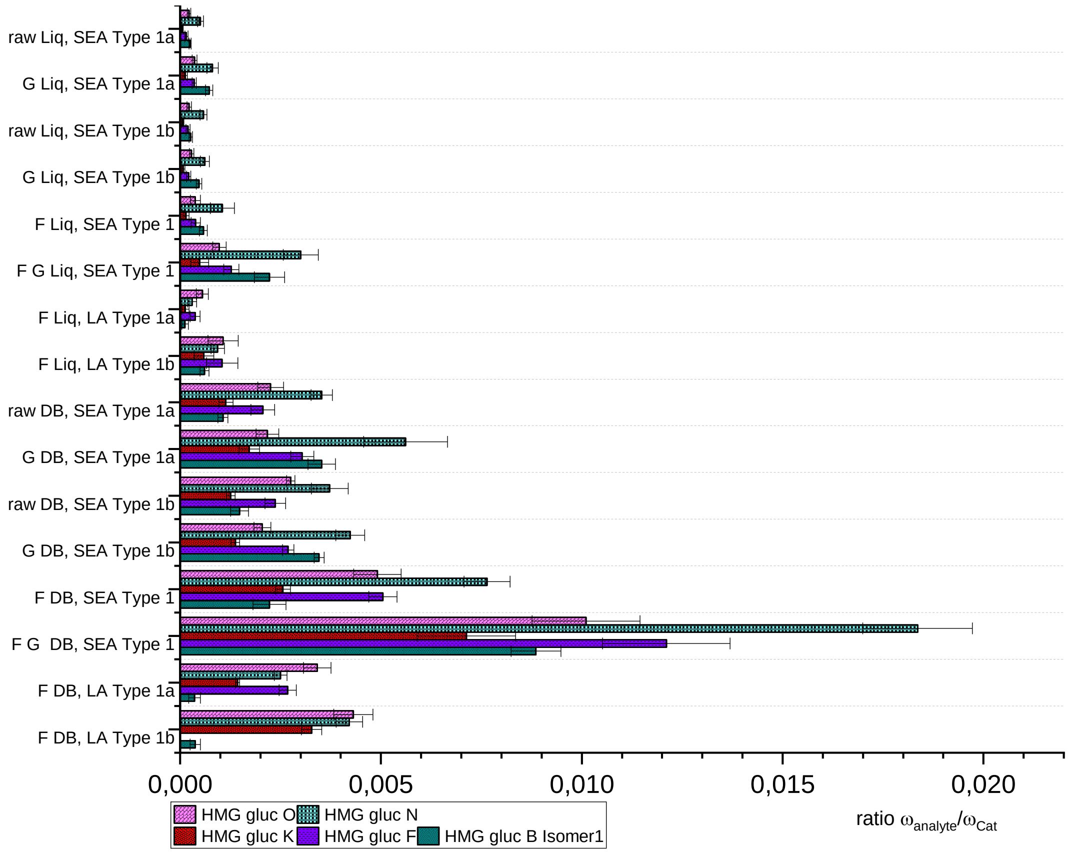

Figure S 4E: Mean values of content ratios of marker compounds $\omega_{analyte}/\omega_{Cat}$, calculated for six replicates of raw, fermented (F) and germinated (G) cocoa liquor (Liq) and dried beans (DB) of Latin American (LA) and Southeast Asian (SEA) origin.

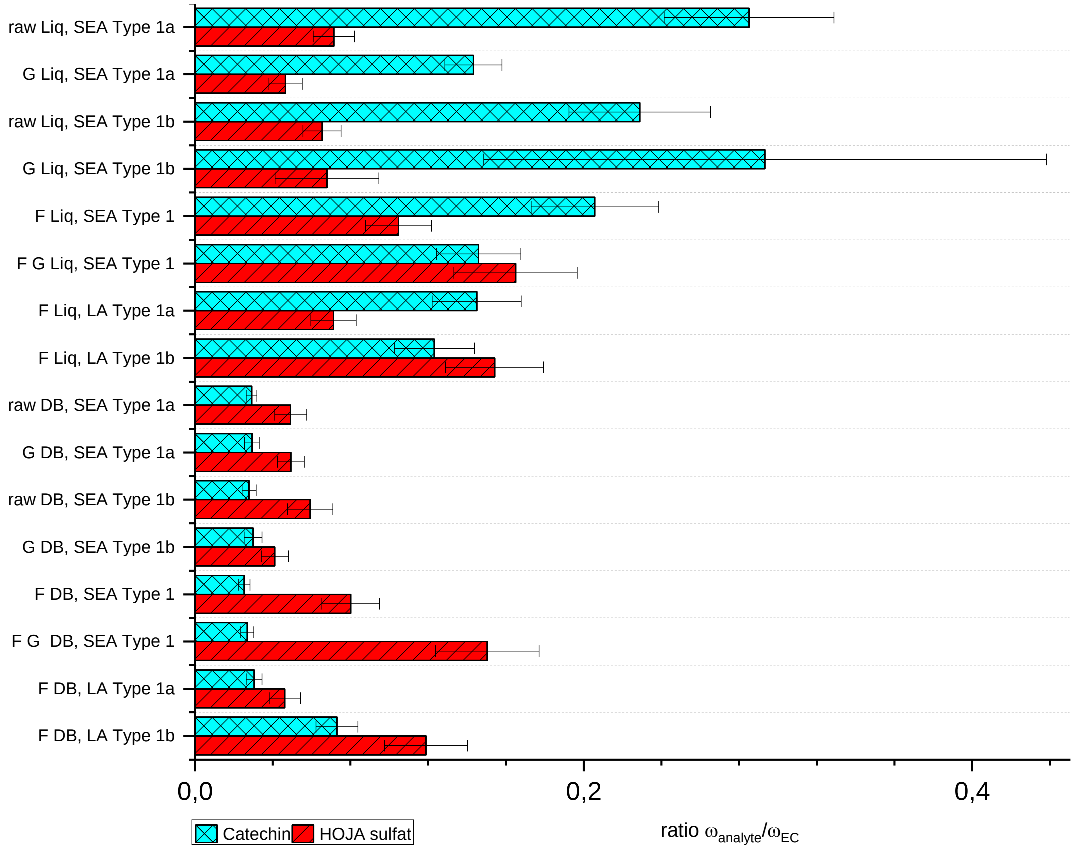

Figure S 5A: Mean values of content ratios of marker compounds $\omega_{analyte}/\omega_{EC}$, calculated for six replicates of raw, fermented (F) and germinated (G) cocoa liquor (Liq) and dried beans (DB) of Latin American (LA) and South East Asian (SEA) origin.

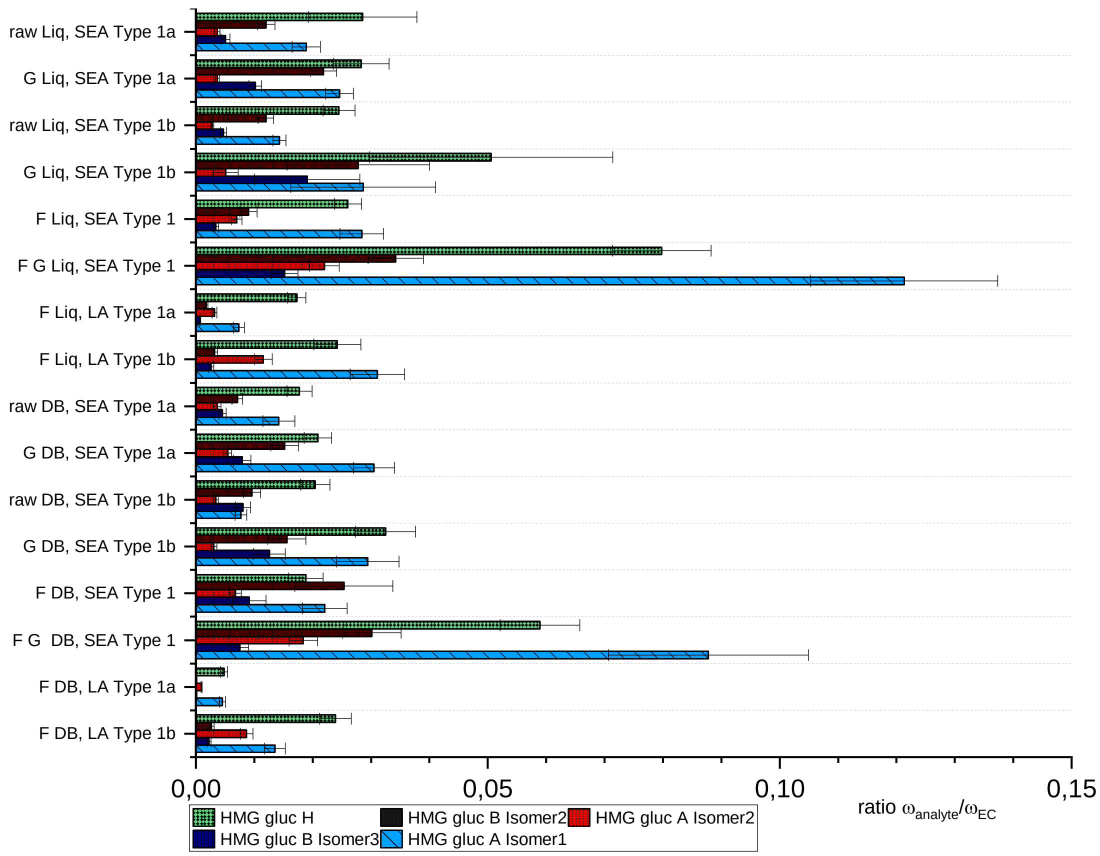

Figure S 5B: Mean values of content ratios of marker compounds $\omega_{analyte}/\omega_{EC}$, calculated for six replicates of raw, fermented (F) and germinated (G) cocoa liquor (Liq) and dried beans (DB) of Latin American (LA) and South East Asian (SEA) origin.

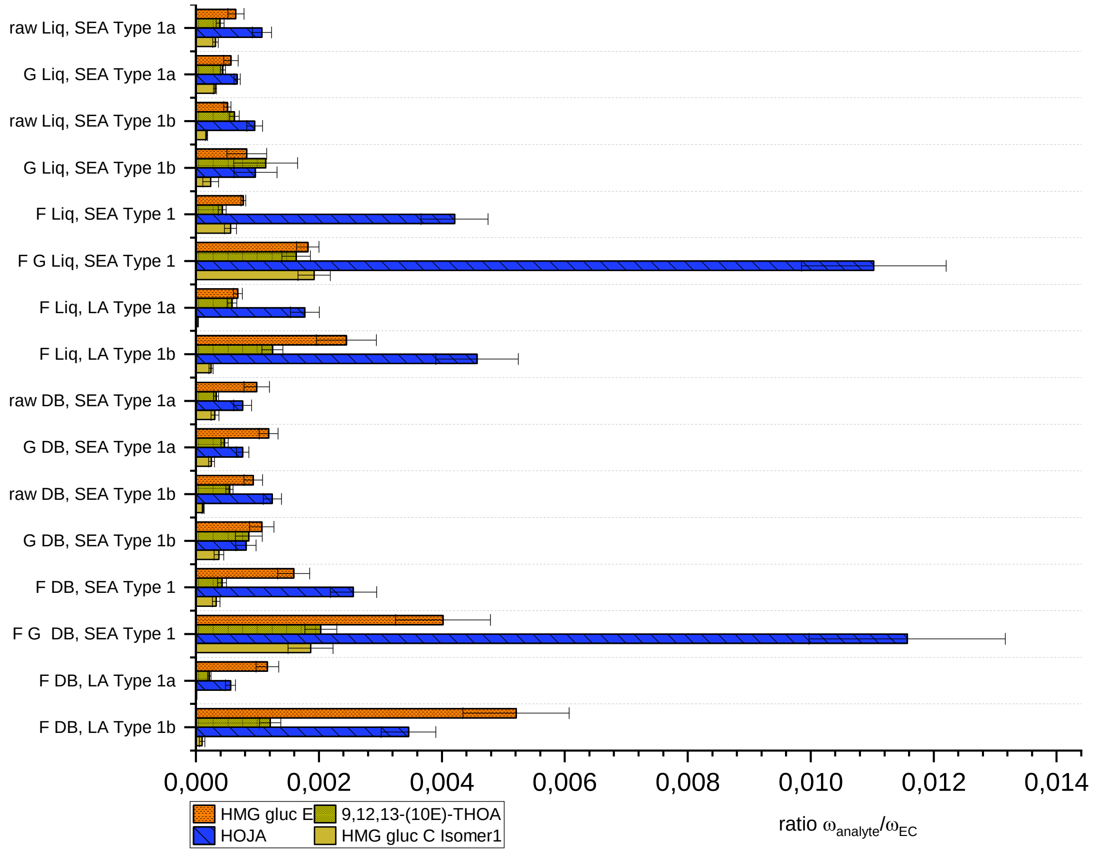

Figure S 5C: Mean values of content ratios of marker compounds $\omega_{analyte}/\omega_{EC}$, calculated for six replicates of raw, fermented (F) and germinated (G) cocoa liquor (Liq) and dried beans (DB) of Latin American (LA) and South East Asian (SEA) origin.

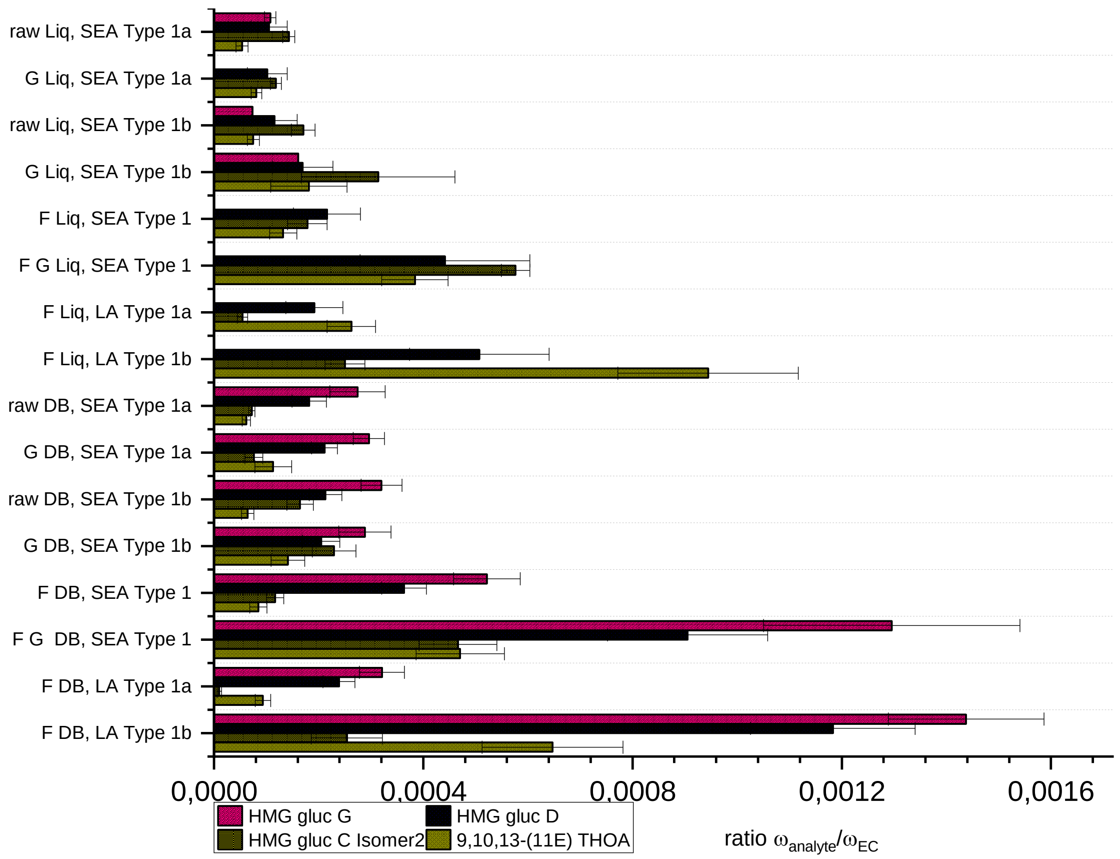

Figure S 5D: Mean values of content ratios of marker compounds $\omega_{analyte}/\omega_{EC}$, calculated for six replicates of raw, fermented (F) and germinated (G) cocoa liquor (Liq) and dried beans (DB) of Latin American (LA) and South East Asian (SEA) origin.

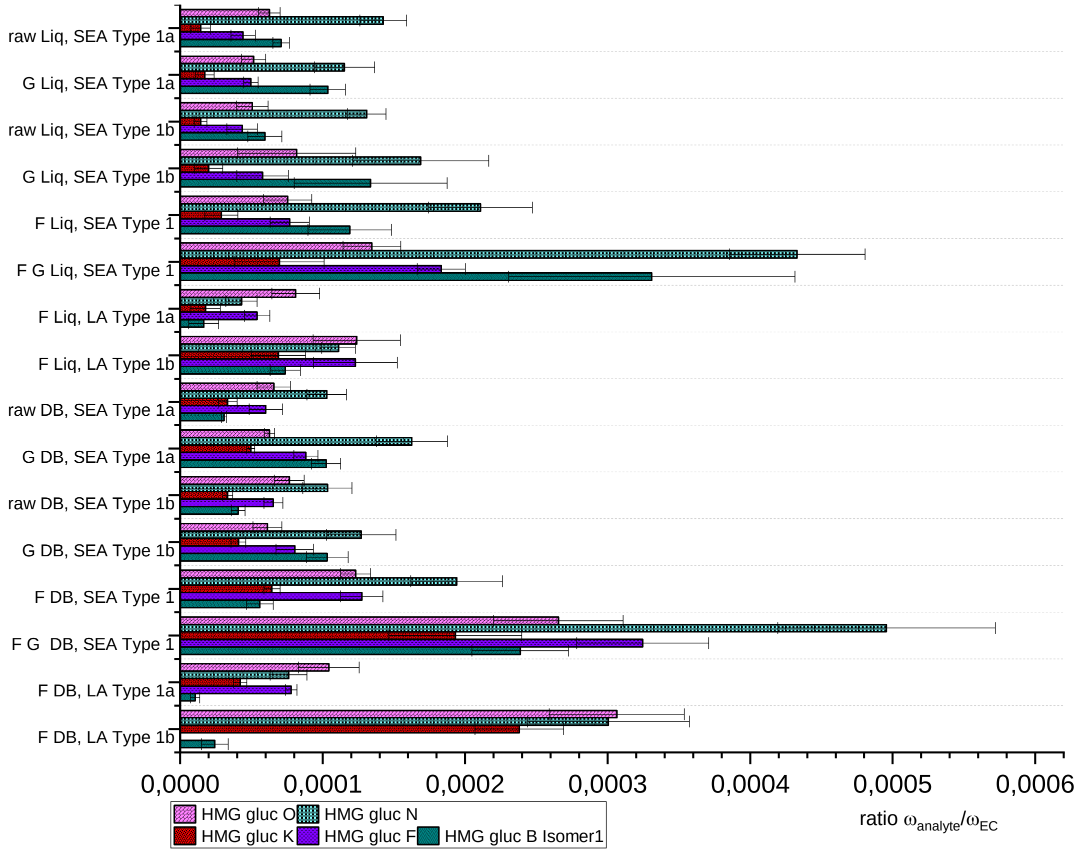

Figure S 5E: Mean values of content ratios of marker compounds $\omega_{analyte}/\omega_{EC}$, calculated for six replicates of raw, fermented (F) and germinated (G) cocoa liquor (Liq) and dried beans (DB) of Latin American (LA) and South East Asian (SEA) origin.

Results of comparison of all measured samples

Previous plots included only those samples which were used in the non-targeted profiling. However, to evaluate the overall applicability as markers, these compounds were quantified in all samples available (Table S 1). Due to the vast amount of data, these quantification results are depicted in a heatmap (Figure S 6), in which samples are grouped according to the processing type (fermentation, raw material, alternative fermentation, micro-fermentation, combination of fermentation and germination, germination) using a color code.

The results of the quantification (depicted in Table S 8 and Figure S 6) indicate that all three HMG gluc B isomers, HMG gluc A isomer 1, HMG gluc C isomer 2, and HMG gluc H impart highest contents only in the germinated samples (red; samples 8, 9, 21, and 23), whereas their contents are distinguishably lower in the remaining samples. Moreover, this accounts only for both HMG gluc A isomers in those samples (12 and 17), which were treated in a combination of fermentation and germination. Upon comparison, the concentration of HOJA sulfate appears to be highest in the raw samples corresponding to the germinated samples (10, 11, 20, and 22) but is lower in the remaining raw and fermented samples. (+)-catechin features higher contents in only some of the raw (104, 108, 86, 91, 10, and 11) and germinated (8 and 9) samples but is found in low concentrations in all the remaining samples. Here, it seems to be necessary to mention that the promising high p-value in the S-plot might be obtained due to the limitation of using only liquor samples in the profiling.[22] Contents of (−)-epicatechin also appear to be high in some raw samples (79, 84, 86, 104, 105, 108, and 109) but lower in other raw, unroasted samples (93, 97, 101) as well as raw and roasted samples (91, 92, 96, 100). Moreover, contents of (−)-epicatechin were reduced in all fermented, unroasted, and roasted samples as well as in alternatively fermented, micro-

fermented, and germinated samples, which is in accordance to literature.[18] Additionally, contents of HOJA appear to be increased in fermented Southeast Asian samples (13, 16, 71–74), while HMG gluc C isomer 1 is highest in raw and fermented West African samples (31–33, 43–45, 49, 50). HMG glucosides H and J feature highest concentrations among raw and processed samples of Southeast Asian origin.

Figure S 7 depicts the mean values of these ratios calculated for each replicate. In this plot, both HMG gluc B isomers 2 and 3 ratios appeared to be even more abundant in germinated samples than in the remaining raw and fermented samples, compared to results found for the contents. In contrast, HMG gluc A isomers 1 and 2 did not appear as universal marker compounds when used in HOJA sulfate ratios. Moreover, HMG gluc H appears to be upregulated only in germinated samples, as reflected by its content and its $\omega_{analyte}/\omega_{HOJA\ sulfate}$ ratio. Additionally, in this plot, no unequivocal marker ratio could be found characteristic for alternatively fermented samples. Within this group, differences due to the provenience (LA type 1 and LA type 4) appear to be more influential than other factors. LA type 1 samples (85, 87, 106, 107) feature highest abundances within all samples for ratios formed with HMG gluc F and O, whereas LA type 4 samples (94, 95) impart highest abundances for the ratio formed between 9,12,13-(10*E*)-trihydroxy octadecenoic acid and HOJA sulfate.

Summary of quantification

Table S 8 (following pages): Mean values and standard deviations of concentrations (mg/kg) of all quantified analytes in cocoa, liquor and chocolate samples (based on 6 technical replicates per samples). Sample codes are explained in Table S1. All samples were quantified using internal and external calibration except (+)-catechin and (-)-epicatechin, which were only quantified by external calibration.

Analyte:	HOJA sulfat		HOJA		Catechin		Epicatechin		9,12,13-(10E)-THOA		9,10,13-(11E) THOA		HMG gluc A Isomer1		HMG gluc A Isomer2		HMG gluc B Isomer1		HMG gluc B Isomer2		HMG gluc B Isomer3	
External Standard:	HOJA sulfat		HOJA		Catechin		Epicatechin		9,12,13-(10E)-THOA		9,12,13-(10E)-THOA		HMG gluc A Isomer1		HMG gluc A Isomer1		HMG gluc A Isomer1		HMG gluc A Isomer1		HMG gluc A Isomer1	
Internal Standard:	Hexylsulfat		Hexylsulfat						HMG gluc NP-022515		HMG gluc NP-022515		HMG gluc NP-022515		HMG gluc NP-022515		HMG gluc NP-022515		HMG gluc NP-022515		HMG gluc NP-022515	
Content	Mean	STD dev	Mean	STD dev	Mean	STD dev	Mean	STD dev	Mean	STD dev	Mean	STD dev	Mean	STD dev	Mean	STD dev	Mean	STD dev	Mean	STD dev	Mean	STD dev
Sample name (No.)																						
raw Liq, SEA Type 1a (#10)	676	37.2	10.1	0.6	4730	438	9582	1020	3.72	0.26	0.5	0.06	179	7.1	34.6	1.52	0.68	0.05	114	2.65	48	3.01
G Liq, SEA Type 1a (#8)	560	78.7	8.1	0.47	3073	400	12170	1488	5.27	0.22	0.98	0.06	297	25.9	44.2	4	1.26	0.19	264	21.5	123	6.7
raw Liq, SEA Type 1b (#11)	685	41	10	0.57	4213	403	10592	1024	6.56	0.35	0.79	0.07	151	5.33	29.4	1.24	0.62	0.09	126	6.99	49.3	2.28
G Liq, SEA Type 1b (#9)	540	70.4	7.72	0.44	3221	1003	8765	2758	8.86	0.96	1.44	0.15	225	22.5	40	3.74	1.06	0.15	219	28.5	148	17.3
F Liq, SEA Type 1 (#13)	573	46.6	23.1	1.31	1473	185	5549	625	2.35	0.15	0.72	0.08	156	8.93	38.5	2.37	0.65	0.13	49.4	4.29	18.6	1.04
F G Liq, SEA Type 1 (#12)	443	35	29.9	1.45	386	54.7	2734	322	4.41	0.41	1.04	0.07	328	24.1	59.6	4.03	0.88	0.17	92.4	5.03	41	2.92
F Liq, LA Type 1a (#15)	360	23.8	8.98	0.3	889	122	5128	563	2.96	0.13	1.33	0.1	37.3	1.52	16.2	0.64	0.09	0.06	8.97	0.62	3.74	0.2
F Liq, LA Type 1b (#14)	311	19.8	9.22	0.4	196	37.8	2045	234	2.51	0.07	1.9	0.14	62.7	2.4	23.4	0.52	0.15	0.02	6.42	0.27	5.26	0.37
raw DB, SEA Type 1a (#22)	722	58.9	11.3	2.62	456	49.5	14886	1933	4.88	0.52	0.91	0.09	211	41.6	53.9	10.3	0.46	0.08	105	12.8	67.1	6.12
G DB, SEA Type 1a (#23)	599	55	9.23	0.77	407	55.4	12252	1324	5.66	0.46	1.36	0.36	371	26.2	66.6	8.28	1.25	0.14	184	11.7	95.5	8.76
raw DB, SEA Type 1b (#20)	731	55.3	15.5	1.05	348	61.9	12600	1605	6.82	0.24	0.8	0.06	96	1.9	42.3	0.82	0.51	0.07	119	3.49	100	6.92
G DB, SEA Type 1b (#21)	529	27.7	10.4	0.79	437	57	13112	1813	11.1	2.13	1.82	0.3	378	17.7	39.7	1.52	1.33	0.05	199	14.7	161	13.8
F DB, SEA Type 1 (#16)	624	45.1	20.1	1.38	124	26.9	7934	960	3.31	0.27	0.66	0.06	172	13.5	53	2.43	0.44	0.09	197	52.2	71	18
F G DB, SEA Type 1 (#17)	466	31.9	36	2.25	1.45	3.55	3165	488	6.34	0.59	1.46	0.17	272	22.6	57.3	3.62	0.74	0.05	94	11.4	23.4	1.98
F DB, LA Type 1a (#18)	449	32.4	5.48	0.31	235	37.2	9872	1149	2.11	0.14	0.92	0.12	44.3	1.69	9.39	0.36	0.11	0.04	1.22	0.13	1.25	0.11
F DB, LA Type 1b (#19)	248	29	7.24	0.37	74.7	21	2125	332	2.52	0.14	1.34	0.1	28.4	3.01	18.2	1.43	0.03	0.03	5.57	0.6	4.68	0.28
raw WCB Liq U, LA Type 1 (#109)	291	42.5	3.5	0.54	474	53.3	14251	2812	10.8	4.99	0.65	0.32	69.9	4.49	11.1	1.1	0.22	0.05	2.11	0.2	2.03	0.31
AF WCB Liq U, LA Type 1 (#107)	151	17.8	2.33	0.2	105	28.1	2930	859	4.05	0.97	0.68	0.05	44.5	4.34	11.2	1.62	0.1	0.02	1.59	0.19	1.46	0.18
raw WCB Liq U, LA Type 1 (#105)	298	34.4	2.29	0.68	569	49.1	19656	4011	9.16	1.15	0.64	0.1	75	6.38	11	2.13	0.13	0.05	1.91	0.44	1.91	0.33
MF WCB Liq U, LA Type 1 (#103)	207	17	4.55	0.24	185	12.5	6133	1088	1.45	0.16	0.25	0.02	59.3	3.41	14.4	0.42	0.19	0.03	1.62	0.15	1.78	0.13
raw WCB Liq R, LA Type 1 (#108)	308	40.1	3.62	0.3	5923	231	11498	2055	6.99	0.67	0.57	0.04	82.2	6.32	13	2.07	0.13	0.04	2.13	0.23	1.97	0.21
AF WCB Liq R, LA Type 1 (#106)	107	7.69	2.39	0.2	696	53.3	2183	391	2.13	0.1	0.59	0.05	25.6	1.82	6.52	0.59	0.07	0.02	1.17	0.21	1.08	0.08
raw WCB Liq R, LA Type 1 (#104)	378	31.9	4.06	0.53	5712	358	13001	2658	6.44	0.73	0.46	0.04	70	5.5	13.5	0.95	0.31	0.06	1.67	0.2	1.38	0.06
MF WCB Liq R, LA Type 1 (#102)	193	18.8	4.78	0.36	999	85.1	3490	648	0.71	0.16	0.26	0.02	59.1	3.29	16.3	0.86	0.16	0.02	1.9	0.17	2.01	0.21
raw Liq U, LA Type 5 (#101)	569	71.2	11.8	1.23	290	16.7	10273	1835	9.99	1.51	1	0.15	198	23.3	39.8	4.67	0.94	0.2	29.3	3.66	12.4	1.47
F Liq U, LA Type 5 (#99)	418	22.9	13.8	0.71	61.4	2.73	1573	265	3.88	0.21	1.96	0.14	161	6.33	26.8	1.09	0.77	0.16	9.12	0.73	9.63	1.07
raw Liq HR, LA Type 5 (#100)	615	45.6	13	0.63	285	20	10639	2029	10.7	0.66	1.04	0.05	219	12.4	43.8	2.65	1.01	0.1	31.7	1.93	14.1	1.02
F Liq HR, LA Type 5 (#98)	404	27.4	11.3	0.56	226	9.5	1650	281	3.34	0.14	1.67	0.09	161	4.95	17.3	0.77	0.76	0.07	3.54	0.3	3.51	0.24
raw Liq U, LA Type 4 (#97)	480	73	4.58	0.95	213	32	3906	515	4.92	0.86	0.23	0.05	125	20.4	<LOQ	<LOQ	0.14	0.07	5.77	0.95	5.15	0.77
AF Liq U, LA Type 4 (#95)	259	20.5	4.76	0.57	<LOQ	<LOQ	120	7.64	14	1.7	1.05	0.06	103	11.7	<LOQ	<LOQ	0.08	0.04	3.47	0.42	3.63	0.52
raw Liq HR, LA Type 4 (#96)	406	24.8	4.07	0.34	876	46.7	2267	131	4.87	0.41	0.2	0.02	125	9.93	<LOQ	<LOQ	0.13	0.02	4.29	0.39	4.1	0.24
AF Liq HR, LA Type 4 (#94)	112	12.2	2.13	0.23	22.3	3.29	152	19	4.95	0.6	0.39	0.06	38.5	5.04	<LOQ	<LOQ	0.03	0.02	1.88	0.3	1.92	0.24
raw Liq U, LA Type 1 (#93)	304	19.6	0.59	0.07	412	27.6	8761	507	0.61	0.08	0.04	0.01	64.2	5.67	1.58	0.64	0.1	0.03	7	0.61	1.02	0.15
F Liq U, LA Type 1 (#90)	361	21.2	2.53	0.32	277	16.7	6549	376	0.78	0.06	0.24	0.03	93.2	4.28	11.2	1.1	0.06	0.03	2.04	0.12	1.3	0.1
raw Liq R, LA Type 1 (#92)	373	17.8	0.7	0.38	1336	79.2	9588	495	0.46	0.05	0.02	0.01	76.6	6.49	0.34	0.44	0.1	0.03	2.09	0.19	1.2	0.1
F Liq R, LA Type 1 (#89)	321	61.6	1.83	0.3	501	107	5299	970	0.58	0.09	0.26	0.04	83.7	14	5.15	1.18	0.08	0.02	3.3	0.66	0.89	0.2
raw Liq HR, LA Type 1 (#91)	385	317	0.95	0.76	3426	1311	10939	3951	0.6	0.47	0.03	0.02	76.1	61.5	0.4	0.71	0.1	0.09	2.18	1.75	0.84	0.69
F Liq HR, LA Type 1 (#88)	325	20.2	2.94	0.26	900	64.6	4050	291	1.31	0.14	0.6	0.02	86.7	6.67	3.81	1.48	0.1	0.02	2.52	0.14	1.03	0.08
raw DB U, LA Type 1 (#84)	380	14.3	2.94	0.49	332	25.4	19062	3674	12.8	0.36	0.62	0.04	55.8	1.47	35.3	2.76	0.65	0.11	7.28	0.16	2.91	0.15
AF DB U, LA Type 1 (#85)	182	11.2	3.42	0.22	108	6.47	5732	863	3.7	0.78	0.39	0.02	120	3.82	33.8	1.86	0.31	0.09	3.86	0.22	2.73	0.29
raw Liq, LA Type 1 (#86)	213	34.2	2.49	0.18	3330	387	8499	1584	4.11	0.83	0.28	0.05	60.1	9.3	15.1	2.27	0.32	0.05	3.62	0.57	2.1	0.36
AF Liq, LA Type 1 (#87)	137	7.91	3.59	0.27	606	52.4	3135	532	4.07	0.25	0.68	0.06	62.4	3.27	20	1.25	0.14	0.04	2.51	0.17	2.09	0.19

Analyte:	HOJA sulfat		HOJA		Catechin		Epicatechin		9,12,13-(10E)-THOA		9,10,13-(11E) THOA		HMG gluc A Isomer1		HMG gluc A Isomer2		HMG gluc B Isomer1		HMG gluc B Isomer2		HMG gluc B Isomer3	
External Standard:	HOJA sulfat		HOJA		Catechin		Epicatechin		9,12,13-(10E)-THOA		9,12,13-(10E)-THOA		HMG gluc A Isomer1		HMG gluc A Isomer1		HMG gluc A Isomer1		HMG gluc A Isomer1		HMG gluc A Isomer1	
Internal Standard:	Hexylsulfat		Hexylsulfat						HMG gluc NP-022515		HMG gluc NP-022515		HMG gluc NP-022515		HMG gluc NP-022515		HMG gluc NP-022515		HMG gluc NP-022515		HMG gluc NP-022515	
Content	Mean	STD dev	Mean	STD dev	Mean	STD dev	Mean	STD dev	Mean	STD dev	Mean	STD dev	Mean	STD dev	Mean	STD dev	Mean	STD dev	Mean	STD dev	Mean	STD dev
Sample name (No.)																						
raw Nibs, SEA Type 1 (#75)	587	36.1	20.6	0.85	179	7.66	5839	388	3.19	0.29	0.65	0.05	244	10.8	41.8	2.37	0.27	0.05	39.7	1.13	13	0.31
F Nibs U, SEA Type 1 (#73)	524	25.6	23.5	0.42	136	5.29	4821	136	3.99	0.18	0.92	0.08	202	8.46	39.7	3.1	0.24	0.02	20	1.41	12.8	0.38
F Nibs U, SEA Type 1 (#71)	537	32.1	23.5	2.87	145	4.83	5249	234	3.54	0.26	0.69	0.09	171	10.8	41.3	4.34	0.21	0.03	20.4	1.48	10.4	0.88
raw Nibs, LA Type 1 (#63)	558	39.1	10.5	0.9	305	33.5	5504	479	3.43	0.25	1.13	0.13	73.3	3.95	4.29	1.56	0.13	0.04	8.28	1	5.66	0.53
F Nibs U, LA Type 1 (#61)	388	21.2	8.18	0.44	119	5.29	3332	184	3.12	0.28	1.6	0.04	78.2	3.81	18.2	3.38	0.07	0.04	4.51	0.24	2.75	0.23
F Nibs U, LA Type 1 (#58)	298	12.6	8.68	0.69	97.4	2.58	2842	54.6	4.15	0.25	2.31	0.29	42.1	3.97	11	0.93	0.03	0.03	6.69	1.02	3.29	0.49
raw DB, LA Type 3 (#77)	425	29.1	6.37	0.56	733	12.8	9326	231	1.03	0.08	0.37	0.03	27.6	2.22	1.3	1.66	0.03	0.03	0.95	0.03	0.9	0.13
raw DB, LA Type 3_2 (#79)	229	59.2	8.65	2.38	827	301	11521	4774	1.14	0.33	0.43	0.11	27	6.48	11	2.08	0.1	0.05	1.09	0.26	1.31	0.18
F DB U, WA Type 2 (#49)	471	20.5	17.5	0.88	198	13.9	2338	137	7.76	0.89	1.3	0.08	173	11.4	7.39	0.9	0.21	0.06	12.2	1.19	13.4	1.58
raw Liq, SEA Type 1 (#76)	352	13.9	22.2	0.72	1219	61.9	4801	176	3.42	0.17	0.61	0.03	236	7.97	42.5	1.74	0.8	0.17	36.5	0.96	21.6	0.84
F Liq, SEA Type 1 (#74)	321	26.6	24.6	1.9	866	41.8	3480	96.1	3.77	0.29	0.72	0.06	223	14.6	40.1	3.34	0.85	0.12	36.9	2.79	26	2.25
F Liq, SEA Type 1 (#72)	250	53.6	17.8	3.39	734	151	3157	634	2.49	0.64	0.51	0.13	122	24.6	25.9	5.17	0.44	0.08	21.6	4.09	12.2	2.51
raw Liq, LA Type 1 (#64)	390	17.5	9.62	0.56	720	13.5	3330	85.6	2.78	0.17	0.63	0.04	79.3	2.98	9.34	0.33	0.48	0.03	7.69	0.19	4.56	0.17
F Liq, LA Type 1 (#62)	290	20.6	11.8	1.1	452	30.4	2260	183	3.12	0.28	1.3	0.11	117	8.31	23.6	1.82	0.49	0.07	17.7	1.17	10.9	0.95
F Liq, LA Type 1 (#60)	199	8.87	6.66	0.2	423	28.9	2166	130	2.83	0.2	1.55	0.13	37.2	1.75	10.9	0.57	0.11	0.03	4.41	0.34	2.58	0.1
raw Liq, LA Type 3 (#78)	190	5.87	10.1	0.61	626	30.4	4802	284	1.37	0.1	0.56	0.06	24.5	1.99	10.2	0.74	0.02	0.03	1.23	0.15	1.46	0.15
F Liq, WA Type 2 (#50)	166	11.8	9.19	0.46	139	17.1	595	53.5	2.19	0.17	0.79	0.03	84.3	3.76	12.1	0.47	0.36	0.05	7.17	0.63	4.49	0.27
F Nibs U, WA Type 2a (#31)	313	42.2	10.2	1.03	27.8	17.2	2204	872	3.01	0.3	1.19	0.15	75	16.6	32.4	7.37	0.19	0.06	6.91	0.96	4.51	0.77
F Nibs U, WA Type 2b (#32)	278	36.8	11.6	1.25	67.9	8.83	2332	810	4.31	1.16	0.94	0.2	175	35	9.74	1.58	0.32	0.09	2.89	0.58	1.44	0.18
F Nibs U, WA Type 2c (#33)	267	26.5	5.82	0.38	15.8	14.9	1802	630	1.88	0.25	0.9	0.06	93.9	4.83	10.7	0.83	0.29	0.09	4.44	0.31	2.42	0.15
F Nibs U, LA Type 1a (#28)	190	23.8	10.1	0.86	73.7	9.01	1899	686	3.42	0.3	0.6	0.06	46	4.2	10.2	0.62	0.18	0.06	3.15	0.3	2.73	0.19
F Nibs U, LA Type 1b (#29)	172	27.1	9.68	1.47	74.6	8.82	1887	709	3.26	0.4	0.59	0.11	37	4.83	18.3	2.38	0.11	0.03	2.16	0.4	2.09	0.45
F Nibs U, LA Type 1c (#30)	194	21.5	9.48	0.85	207	46.2	2860	1025	3.18	0.4	0.82	0.13	34.4	4.54	13.4	1.91	0.14	0.05	3.88	0.72	2.86	0.54
F Liq, WA Type 2a (#43)	337	30.3	13.9	0.54	275	62.7	1988	717	3.15	0.13	1.44	0.03	142	4.01	16.5	0.8	0.51	0.1	10.5	0.47	5.42	0.31
F Liq, WA Type 2b (#44)	368	47.8	13.2	0.72	394	109	2252	838	3.63	0.39	1.63	0.14	113	8.8	19.6	1.46	0.43	0.13	12.7	1.15	8.06	0.68
F Liq, WA Type 2c (#45)	320	28.3	12.3	1.15	247	57.3	1900	696	2.94	0.21	1.4	0.13	133	7.65	17.6	1	0.43	0.06	9.1	0.54	5.88	0.48
F Liq, LA Type 1a (#40)	203	20.3	12.2	0.7	352	95.9	1417	521	3.7	0.19	0.78	0.08	60.4	3.25	15.2	0.68	0.18	0.04	2.96	0.15	2.48	0.22
F Liq, LA Type 1b (#41)	202	18.6	10.7	0.97	599	180	2191	849	3.42	0.28	0.91	0.06	39.2	2.44	12	0.87	0.13	0.05	2.09	0.18	1.78	0.11
F Liq, LA Type 1c (#42)	229	18.6	10.7	0.62	803	253	3028	1137	3.62	0.08	0.99	0.07	50.6	1.85	15.2	0.67	0.22	0.05	2.54	0.22	2.21	0.12
raw Choc U, LA Type 1 (#80)	197	50.1	1.67	0.37	438	127	13781	4521	0.55	0.17	0.02	0.01	62.7	14.7	6.64	1.67	0.24	0.06	10	2.47	1.76	0.32
F Choc U, LA Type 1 (#82)	303	26.9	4.8	0.43	386	15.2	12377	1964	0.9	0.05	0.3	0.03	109	4.09	22.3	1.04	0.3	0.08	3.84	0.24	2.66	0.08
raw Choc R, LA Type 1 (#81)	201	34.5	1.75	0.08	1252	79.5	12725	2115	0.37	0.05	0.02	0.01	65.7	5.4	8.01	0.87	0.23	0.02	2.74	0.34	1.66	0.15
F Choc R, LA Type 1 (#83)	261	27	3.36	0.34	597	50.4	9088	1635	0.6	0.09	0.29	0.04	91.8	9.81	12.1	1.44	0.26	0.05	5.78	0.52	1.77	0.17
Choc #1 (#1)	112	4.54	7.95	0.57	66.8	2.61	316	35.3	1.71	0.05	0.68	0.04	42.4	1.96	6.31	0.35	0.17	0.04	3.35	0.39	2.28	0.19
Choc #2 (#2)	138	17.1	8.08	1.22	74.2	7.32	422	58.3	1.68	0.2	0.7	0.07	58.7	8.04	8.11	0.91	0.23	0.05	4.69	0.75	3.16	0.48
Choc #3 (#3)	124	21.4	8.03	1.6	85	15.2	546	95.5	1.7	0.25	0.67	0.11	45.2	7.58	7.27	0.97	0.17	0.05	4.46	0.83	2.71	0.52
Choc #4 (#4)	133	19	9.41	1.15	160	22.6	635	124	2.04	0.29	0.98	0.19	27	3.94	5.57	0.67	0.15	0.04	2.9	0.39	1.88	0.31
Choc #5 (#5)	121	11	6.63	0.68	60.3	6.54	443	63.6	1.43	0.12	0.52	0.07	46.4	4	5.99	0.7	0.16	0.04	2.92	0.21	2.1	0.14
Choc #6 (#6)	145	4.33	7.76	0.44	187	9.31	1844	256	1.82	0.12	0.74	0.04	41.4	0.42	7.91	0.26	0.18	0.03	4.75	0.23	2.76	0.22
Liq Choc #6 (#7)	158	19.4	12.3	1.51	222	23.4	677	120	3.42	0.39	1.25	0.14	55.1	6.87	12.3	1.59	0.17	0.02	7.11	0.98	3.59	0.54

Analyte:	HMG gluc C Isomer1		HMG gluc C Isomer2		HMG gluc D		HMG gluc E		HMG gluc F		HMG gluc G		HMG gluc H		HMG gluc J		HMG gluc K		HMG gluc L		HMG gluc M		HMG gluc N		HMG gluc O	
External Standard:	HMG gluc C Isomer1		HMG gluc C Isomer1		HMG gluc D		HMG gluc E		HMG gluc F		HMG gluc G		HMG gluc H		HMG gluc J		HMG gluc K		HMG gluc L		HMG gluc M		HMG gluc N		HMG gluc O	
Internal Standard:	HMG gluc NP-022515		HMG gluc NP-022515		NP-007735		NP-014823		NP-015777		NP-018505		NP-021228		NP-022828		NP-023425		NP-023820		NP-023821		NP-024048		NP-023236	
**Content**	**Mean**	STD dev	**Mean**	STD dev	**Mean**	STD dev	**Mean**	STD dev	**Mean**	STD dev	**Mean**	STD dev	**Mean**	STD dev	**Mean**	STD dev	**Mean**	STD dev	**Mean**	STD dev	**Mean**	STD dev	**Mean**	STD dev	**Mean**	STD dev
**Sample name (No.)**																										
**raw Liq, SEA Type 1a (#10)**	**3.03**	0.19	**1.36**	0.09	**1.03**	0.44	**6.21**	1.28	**0.42**	0.1	**0.31**	0.48	**276**	107	**0.19**	0.04	**0.14**	0.08	**<LOQ**	<LOQ	**<LOQ**	<LOQ	**1.36**	0.12	**0.6**	0.11
**G Liq, SEA Type 1a (#8)**	**3.75**	0.3	**1.44**	0.23	**1.28**	0.61	**6.85**	1.16	**0.6**	0.09	**<LOQ**	<LOQ	**340**	37.7	**0.24**	0.06	**0.22**	0.1	**<LOQ**	<LOQ	**<LOQ**	<LOQ	**1.39**	0.19	**0.63**	0.11
**raw Liq, SEA Type 1b (#11)**	**1.82**	0.12	**1.79**	0.15	**1.26**	0.58	**5.4**	0.39	**0.47**	0.15	**0.12**	0.29	**257**	6.31	**0.19**	0.07	**0.16**	0.06	**<LOQ**	<LOQ	**<LOQ**	<LOQ	**1.38**	0.14	**0.54**	0.15
**G Liq, SEA Type 1b (#9)**	**1.84**	0.33	**2.44**	0.36	**1.41**	0.45	**6.58**	0.96	**0.47**	0.09	**0.33**	0.81	**397**	30.9	**0.23**	0.06	**0.17**	0.08	**<LOQ**	<LOQ	**<LOQ**	<LOQ	**1.38**	0.16	**0.64**	0.13
**F Liq, SEA Type 1 (#13)**	**3.08**	0.25	**0.99**	0.19	**1.22**	0.45	**4.26**	0.33	**0.43**	0.11	**<LOQ**	<LOQ	**143**	7.01	**0.18**	0.08	**0.16**	0.08	**<LOQ**	<LOQ	**<LOQ**	<LOQ	**1.18**	0.29	**0.42**	0.13
**F G Liq, SEA Type 1 (#12)**	**5.21**	0.45	**1.57**	0.15	**1.25**	0.58	**4.94**	0.37	**0.5**	0.08	**<LOQ**	<LOQ	**216**	8.87	**0.2**	0.06	**0.19**	0.09	**<LOQ**	<LOQ	**<LOQ**	<LOQ	**1.18**	0.13	**0.31**	0.17
**F Liq, LA Type 1a (#15)**	**0.18**	0.04	**0.28**	0.05	**0.66**	0.59	**2.27**	1.76	**0.18**	0.15	**<LOQ**	<LOQ	**57.8**	45.1	**0.06**	0.07	**0.06**	0.07	**<LOQ**	<LOQ	**<LOQ**	<LOQ	**0.15**	0.13	**0.27**	0.23
**F Liq, LA Type 1b (#14)**	**0.5**	0.06	**0.51**	0.06	**1.05**	0.35	**4.92**	0.51	**0.26**	0.09	**<LOQ**	<LOQ	**48.8**	3.45	**0.13**	0.05	**0.14**	0.05	**<LOQ**	<LOQ	**<LOQ**	<LOQ	**0.15**	0.12	**0.17**	0.15
**raw DB, SEA Type 1a (#22)**	**4.57**	0.94	**1.08**	0.19	**2.68**	0.43	**14.5**	2.68	**0.89**	0.18	**4.04**	0.65	**261**	24.5	**0.73**	0.13	**0.49**	0.1	**<LOQ**	<LOQ	**<LOQ**	<LOQ	**1.52**	0.15	**0.97**	0.17
**G DB, SEA Type 1a (#23)**	**3.05**	0.43	**0.92**	0.17	**2.57**	0.2	**14.3**	0.85	**1.08**	0.08	**3.6**	0.15	**254**	7.12	**0.66**	0.05	**0.61**	0.06	**<LOQ**	<LOQ	**<LOQ**	<LOQ	**1.97**	0.22	**0.77**	0.1
**raw DB, SEA Type 1b (#20)**	**1.46**	0.18	**2.04**	0.12	**2.67**	0.4	**11.6**	1.32	**0.82**	0.11	**4**	0.44	**255**	12.8	**0.68**	0.09	**0.29**	0.23	**<LOQ**	<LOQ	**<LOQ**	<LOQ	**1.28**	0.12	**0.96**	0.1
**G DB, SEA Type 1b (#21)**	**4.84**	0.47	**2.95**	0.2	**2.64**	0.32	**13.8**	1.49	**1.04**	0.09	**3.73**	0.45	**419**	26.2	**0.72**	0.09	**0.53**	0.05	**<LOQ**	<LOQ	**<LOQ**	<LOQ	**1.64**	0.22	**0.79**	0.12
**F DB, SEA Type 1 (#16)**	**2.58**	0.28	**0.92**	0.1	**2.85**	0.11	**12.4**	0.75	**1**	0.04	**4.1**	0.32	**147**	10.5	**0.37**	0.41	**0.51**	0.03	**<LOQ**	<LOQ	**<LOQ**	<LOQ	**1.51**	0.08	**0.97**	0.09
**F G DB, SEA Type 1 (#17)**	**5.76**	0.46	**1.45**	0.12	**2.8**	0.08	**12.4**	0.56	**1.01**	0.05	**4**	0.16	**184**	12.1	**0.12**	0.3	**0.59**	0.05	**<LOQ**	<LOQ	**<LOQ**	<LOQ	**1.54**	0.09	**0.69**	0.34
**F DB, LA Type 1a (#18)**	**0.09**	0.04	**0.1**	0.05	**2.33**	0.15	**11.3**	0.79	**0.25**	0.39	**1.53**	1.68	**47**	2.34	**<LOQ**	<LOQ	**0.35**	0.17	**<LOQ**	<LOQ	**<LOQ**	<LOQ	**0.74**	0.05	**1.01**	0.12
**F DB, LA Type 1b (#19)**	**0.21**	0.1	**0.53**	0.11	**2.48**	0.28	**10.9**	1.15	**<LOQ**	<LOQ	**1.56**	1.73	**50.2**	4.14	**0.1**	0.26	**0.5**	0.05	**<LOQ**	<LOQ	**<LOQ**	<LOQ	**0.22**	0.34	**0.22**	0.35
**raw WCB Liq U, LA Type 1 (#109)**	**0.33**	0.03	**<LOQ**	<LOQ	**2.16**	0.58	**6.83**	2.39	**0.98**	0.13	**0.89**	1.39	**7.21**	1.23	**0.15**	0.24	**<LOQ**	<LOQ	**<LOQ**	<LOQ	**<LOQ**	<LOQ	**0.66**	0.09	**0.8**	0.19
**AF WCB Liq U, LA Type 1 (#107)**	**0.12**	0.02	**0.05**	0.03	**2.4**	0.58	**6.86**	1.98	**1.08**	0.09	**3.74**	0.44	**14.6**	1.23	**<LOQ**	<LOQ	**0.42**	0.24	**<LOQ**	<LOQ	**<LOQ**	<LOQ	**0.28**	0.31	**0.67**	0.36
**raw WCB Liq U, LA Type 1 (#105)**	**0.22**	0.09	**0.01**	0.01	**2.93**	1.17	**8.39**	3.41	**0.68**	0.54	**2.32**	2.7	**10.8**	3.45	**0.09**	0.21	**0.12**	0.18	**<LOQ**	<LOQ	**<LOQ**	<LOQ	**<LOQ**	<LOQ	**1.01**	0.34
**MF WCB Liq U, LA Type 1 (#103)**	**0.36**	0.06	**0.65**	0.08	**2.62**	0.65	**7.07**	2.63	**1.18**	0.11	**3.77**	0.49	**70.7**	4.97	**<LOQ**	<LOQ	**0.23**	0.35	**<LOQ**	<LOQ	**<LOQ**	<LOQ	**0.54**	0.29	**0.9**	0.13
**raw WCB Liq R, LA Type 1 (#108)**	**0.28**	0.03	**0.01**	0.01	**2.67**	0.68	**7.81**	2.83	**0.57**	0.63	**3.33**	1.77	**8.81**	1.3	**0.08**	0.21	**0.06**	0.14	**<LOQ**	<LOQ	**<LOQ**	<LOQ	**0.33**	0.36	**1**	0.33
**AF WCB Liq R, LA Type 1 (#106)**	**0.07**	0.02	**0.06**	0.01	**1.95**	1.09	**7.06**	1.89	**0.73**	0.58	**2.52**	2	**16**	1.13	**<LOQ**	<LOQ	**<LOQ**	<LOQ	**<LOQ**	<LOQ	**<LOQ**	<LOQ	**<LOQ**	<LOQ	**0.89**	0.17
**raw WCB Liq R, LA Type 1 (#104)**	**0.59**	0.11	**<LOQ**	0.01	**3.09**	1.2	**8.24**	3.68	**1.58**	0.45	**5.48**	1.36	**10.6**	3.29	**<LOQ**	<LOQ	**0.4**	0.38	**<LOQ**	<LOQ	**<LOQ**	<LOQ	**1.43**	0.29	**1.18**	0.43
**MF WCB Liq R, LA Type 1 (#102)**	**0.28**	0.04	**0.52**	0.07	**2.91**	0.91	**7.9**	3.26	**1.01**	0.57	**4.66**	1.1	**55.1**	4.4	**<LOQ**	<LOQ	**0.13**	0.21	**<LOQ**	<LOQ	**<LOQ**	<LOQ	**0.16**	0.26	**0.68**	0.38
**raw Liq U, LA Type 5 (#101)**	**10.5**	1.23	**1.27**	0.21	**2.81**	0.78	**8.82**	2.96	**0.93**	0.5	**5.33**	1.08	**189**	34.7	**0.42**	0.38	**0.61**	0.19	**<LOQ**	<LOQ	**<LOQ**	<LOQ	**1.9**	0.3	**1.02**	0.32
**F Liq U, LA Type 5 (#99)**	**9.83**	0.48	**0.59**	0.06	**3.66**	0.95	**9.87**	3.67	**1.66**	0.18	**5.72**	0.62	**66.8**	1.82	**0.7**	0.55	**0.59**	0.34	**<LOQ**	<LOQ	**<LOQ**	<LOQ	**1.75**	0.2	**0.15**	0.36
**raw Liq HR, LA Type 5 (#100)**	**11.7**	0.66	**1.47**	0.16	**3.17**	0.85	**9.9**	2.83	**1.11**	0.57	**6.14**	0.69	**209**	12.2	**0.21**	0.33	**0.69**	0.16	**<LOQ**	<LOQ	**<LOQ**	<LOQ	**2.01**	0.11	**1.06**	0.28
**F Liq HR, LA Type 5 (#98)**	**9.3**	0.42	**0.39**	0.05	**2.41**	0.76	**6.46**	2.37	**1.08**	0.17	**4.24**	0.58	**35.9**	3.04	**0.11**	0.27	**0.52**	0.17	**<LOQ**	<LOQ	**<LOQ**	<LOQ	**1.41**	0.13	**<LOQ**	<LOQ
**raw Liq U, LA Type 4 (#97)**	**2.12**	0.39	**0.05**	0.06	**1.47**	0.19	**4.34**	0.34	**<LOQ**	<LOQ	**<LOQ**	<LOQ	**11**	1.14	**<LOQ**	<LOQ	**0.7**	0.11	**<LOQ**	<LOQ	**<LOQ**	<LOQ	**<LOQ**	<LOQ	**<LOQ**	<LOQ
**AF Liq U, LA Type 4 (#95)**	**1.68**	0.26	**0.05**	0.06	**1.17**	0.61	**3.96**	0.5	**<LOQ**	<LOQ	**<LOQ**	<LOQ	**8.32**	1.23	**<LOQ**	<LOQ	**0.69**	0.08	**<LOQ**	<LOQ	**<LOQ**	<LOQ	**<LOQ**	<LOQ	**<LOQ**	<LOQ
**raw Liq HR, LA Type 4 (#96)**	**2.21**	0.17	**0.04**	0.05	**1.48**	0.17	**4.14**	0.38	**<LOQ**	<LOQ	**<LOQ**	<LOQ	**9.86**	0.93	**<LOQ**	<LOQ	**0.71**	0.09	**<LOQ**	<LOQ	**<LOQ**	<LOQ	**0.1**	0.25	**0.07**	0.18
**AF Liq HR, LA Type 4 (#94)**	**0.59**	0.08	**0.02**	0.03	**0.42**	0.49	**2.04**	0.43	**<LOQ**	<LOQ	**<LOQ**	<LOQ	**7.44**	1.05	**<LOQ**	<LOQ	**0.35**	0.05	**<LOQ**	<LOQ	**<LOQ**	<LOQ	**<LOQ**	<LOQ	**<LOQ**	<LOQ
**raw Liq U, LA Type 1 (#93)**	**0.22**	0.02	**0.02**	0.02	**1.02**	0.13	**5.45**	0.29	**0.26**	0.13	**<LOQ**	<LOQ	**5.78**	0.4	**<LOQ**	<LOQ	**0.03**	0.08	**<LOQ**	<LOQ	**<LOQ**	<LOQ	**<LOQ**	<LOQ	**0.47**	0.12
**F Liq U, LA Type 1 (#90)**	**0.41**	0.05	**0.19**	0.09	**1.36**	0.13	**8.5**	0.53	**0.21**	0.23	**0.24**	0.59	**48.2**	3.32	**<LOQ**	<LOQ	**0.14**	0.16	**<LOQ**	<LOQ	**<LOQ**	<LOQ	**<LOQ**	<LOQ	**0.47**	0.25
**raw Liq R, LA Type 1 (#92)**	**0.34**	0.04	**0.06**	0.05	**1.43**	0.12	**5.96**	0.52	**0.22**	0.25	**<LOQ**	<LOQ	**9.58**	0.93	**<LOQ**	<LOQ	**<LOQ**	<LOQ	**<LOQ**	<LOQ	**<LOQ**	<LOQ	**<LOQ**	<LOQ	**0.19**	0.29
**F Liq R, LA Type 1 (#89)**	**0.36**	0.11	**0.21**	0.09	**1.05**	0.15	**6.17**	1.51	**0.21**	0.16	**0.86**	0.69	**41.9**	9.91	**0.03**	0.08	**0.13**	0.15	**<LOQ**	<LOQ	**<LOQ**	<LOQ	**<LOQ**	<LOQ	**0.41**	0.13
**raw Liq HR, LA Type 1 (#91)**	**0.26**	0.21	**0.02**	0.04	**1.84**	0.54	**7.93**	3.04	**0.52**	0.11	**0.37**	0.91	**7.99**	2.35	**<LOQ**	<LOQ	**<LOQ**	<LOQ	**<LOQ**	<LOQ	**<LOQ**	<LOQ	**0.12**	0.3	**0.41**	0.4
**F Liq HR, LA Type 1 (#88)**	**0.31**	0.04	**0.13**	0.11	**1.59**	0.14	**6.72**	0.75	**0.23**	0.26	**0.24**	0.58	**32.7**	1.61	**<LOQ**	<LOQ	**0.13**	0.2	**<LOQ**	<LOQ	**<LOQ**	<LOQ	**<LOQ**	<LOQ	**<LOQ**	<LOQ
**raw DB U, LA Type 1 (#84)**	**0.63**	0.08	**0.02**	0.03	**3.86**	1.73	**10.7**	3.29	**1.1**	0.65	**5.73**	1.33	**13.6**	3.07	**0.18**	0.29	**0.65**	0.37	**<LOQ**	<LOQ	**<LOQ**	<LOQ	**0.16**	0.39	**1.26**	0.43
**AF DB U, LA Type 1 (#85)**	**0.55**	0.04	**0.34**	0.02	**3.64**	0.91	**14.3**	3.51	**1.6**	0.19	**<LOQ**	<LOQ	**40.1**	3.02	**<LOQ**	<LOQ	**0.23**	0.37	**<LOQ**	<LOQ	**<LOQ**	<LOQ	**0.27**	0.42	**1.45**	0.35
**raw Liq, LA Type 1 (#86)**	**0.38**	0.05	**<LOQ**	<LOQ	**3.41**	0.93	**8.86**	3.41	**<LOQ**	<LOQ	**5.38**	0.71	**13.7**	1.28	**0.11**	0.26	**<LOQ**	<LOQ	**<LOQ**	<LOQ	**<LOQ**	<LOQ	**<LOQ**	<LOQ	**1.31**	0.3
**AF Liq, LA Type 1 (#87)**	**0.32**	0.05	**0.13**	0.01	**2.78**	0.8	**7.6**	2.81	**1.02**	0.51	**4.23**	0.55	**27.4**	2.13	**<LOQ**	<LOQ	**0.34**	0.4	**<LOQ**	<LOQ	**<LOQ**	<LOQ	**0.1**	0.23	**1.04**	0.24

Analyte:	HMG gluc C Isomer1		HMG gluc C Isomer2		HMG gluc D		HMG gluc E		HMG gluc F		HMG gluc G		HMG gluc H		HMG gluc J		HMG gluc K		HMG gluc L		HMG gluc M		HMG gluc N		HMG gluc O	
External Standard:	HMG gluc C Isomer1		HMG gluc C Isomer1		HMG gluc D		HMG gluc E		HMG gluc F		HMG gluc G		HMG gluc H		HMG gluc J		HMG gluc K		HMG gluc L		HMG gluc M		HMG gluc N		HMG gluc O	
Internal Standard:	HMG gluc NP-022515		HMG gluc NP-022515		NP-007735		NP-014823		NP-015777		NP-018505		NP-021228		NP-022828		NP-023425		NP-023820		NP-023821		NP-024048		NP-023236	
**Content**	**Mean**	STD dev	**Mean**	STD dev	**Mean**	STD dev	**Mean**	STD dev	**Mean**	STD dev	**Mean**	STD dev	**Mean**	STD dev	**Mean**	STD dev	**Mean**	STD dev	**Mean**	STD dev	**Mean**	STD dev	**Mean**	STD dev	**Mean**	STD dev
**Sample name (No.)**																										
**raw Nibs, SEA Type 1 (#75)**	**3.02**	0.19	**1.15**	0.06	**2.67**	0.52	**4.89**	0.34	**1.64**	0.37	**4.71**	0.79	**192**	10.4	**0.36**	0.19	**0.24**	0.27	**<LOQ**	<LOQ	**<LOQ**	<LOQ	**2.12**	0.36	**0.67**	0.35
**F Nibs U, SEA Type 1 (#73)**	**2.09**	0.25	**0.91**	0.06	**2.29**	0.15	**4.1**	0.91	**1.34**	0.11	**3.88**	0.18	**190**	10	**0.39**	0.03	**0.34**	0.17	**<LOQ**	<LOQ	**<LOQ**	<LOQ	**1.95**	0.14	**0.34**	0.38
**F Nibs U, SEA Type 1 (#71)**	**2.1**	0.15	**0.85**	0.11	**2.34**	0.59	**4.81**	1.7	**1.44**	0.41	**4.08**	0.81	**199**	12.4	**0.25**	0.21	**0.24**	0.19	**<LOQ**	<LOQ	**<LOQ**	<LOQ	**2**	0.2	**0.61**	0.34
**raw Nibs, LA Type 1 (#63)**	**3.16**	0.25	**0.37**	0.06	**2.99**	0.43	**5.62**	1.91	**<LOQ**	<LOQ	**2.21**	2.45	**70.1**	3.37	**<LOQ**	<LOQ	**0.43**	0.21	**<LOQ**	<LOQ	**<LOQ**	<LOQ	**1.65**	0.29	**0.82**	0.44
**F Nibs U, LA Type 1 (#61)**	**1.92**	0.15	**0.34**	0.17	**2.39**	0.55	**5.17**	1.68	**0.19**	0.47	**3.86**	0.84	**75.9**	5.26	**<LOQ**	<LOQ	**0.17**	0.19	**<LOQ**	<LOQ	**<LOQ**	<LOQ	**1.08**	0.26	**0.16**	0.39
**F Nibs U, LA Type 1 (#58)**	**0.6**	0.17	**0.23**	0.03	**2.04**	0.13	**3.42**	0.46	**0.18**	0.43	**3.35**	0.16	**66.5**	6.92	**<LOQ**	<LOQ	**<LOQ**	<LOQ	**<LOQ**	<LOQ	**<LOQ**	<LOQ	**0.16**	0.4	**0.57**	0.3
**raw DB, LA Type 3 (#77)**	**0.26**	0.03	**0.02**	0.03	**3.15**	0.36	**6.1**	1.18	**<LOQ**	<LOQ	**3.08**	2.42	**6.61**	0.5	**0.08**	0.19	**0.08**	0.2	**<LOQ**	<LOQ	**<LOQ**	<LOQ	**<LOQ**	<LOQ	**1.26**	0.18
**raw DB, LA Type 3_2 (#79)**	**0.16**	0.08	**0.03**	0.05	**1.51**	0.25	**3.63**	0.43	**<LOQ**	<LOQ	**2.69**	2.13	**6.27**	0.73	**<LOQ**	<LOQ	**<LOQ**	<LOQ	**<LOQ**	<LOQ	**<LOQ**	<LOQ	**<LOQ**	<LOQ	**1.33**	0.31
**F DB U, WA Type 2 (#49)**	**6.42**	0.76	**0.69**	0.12	**3.21**	0.15	**5.39**	2.98	**<LOQ**	<LOQ	**3.04**	2.37	**52.5**	2.06	**0.35**	0.27	**0.7**	0.1	**<LOQ**	<LOQ	**<LOQ**	<LOQ	**1.88**	0.26	**0.15**	0.36
**raw Liq, SEA Type 1 (#76)**	**2.2**	0.22	**0.85**	0.11	**4.47**	2.28	**27.2**	2.47	**2.58**	0.27	**1.1**	2.69	**212**	19.4	**0.5**	0.25	**0.29**	0.32	**<LOQ**	<LOQ	**<LOQ**	<LOQ	**2.79**	0.75	**0.94**	0.51
**F Liq, SEA Type 1 (#74)**	**2.4**	0.22	**0.84**	0.1	**5.06**	0.81	**26.2**	3.61	**2.44**	0.37	**1.93**	3.06	**167**	24.5	**0.56**	0.1	**0.52**	0.27	**<LOQ**	<LOQ	**<LOQ**	<LOQ	**2.84**	0.78	**0.49**	0.55
**F Liq, SEA Type 1 (#72)**	**1.61**	0.36	**0.54**	0.13	**3.06**	1.56	**18.3**	2.61	**1.38**	0.7	**1.32**	2.05	**123**	31.5	**0.25**	0.2	**0.3**	0.24	**<LOQ**	<LOQ	**<LOQ**	<LOQ	**2.09**	0.65	**0.65**	0.34
**raw Liq, LA Type 1 (#64)**	**3.5**	0.19	**0.28**	0.05	**3.34**	0.13	**16.3**	0.35	**0.73**	0.8	**0.62**	1.52	**57.9**	4.6	**<LOQ**	<LOQ	**0.3**	0.15	**<LOQ**	<LOQ	**<LOQ**	<LOQ	**1.09**	0.06	**0.56**	0.28
**F Liq, LA Type 1 (#62)**	**4.01**	0.5	**0.52**	0.05	**3.78**	0.63	**20.3**	3.25	**0.23**	0.55	**<LOQ**	<LOQ	**79.5**	8.7	**<LOQ**	<LOQ	**0.19**	0.22	**<LOQ**	<LOQ	**<LOQ**	<LOQ	**1.1**	0.26	**0.2**	0.49
**F Liq, LA Type 1 (#60)**	**0.31**	0.05	**0.19**	0.04	**3.23**	1.67	**19.8**	2.71	**0.24**	0.6	**<LOQ**	<LOQ	**62.9**	7.35	**<LOQ**	<LOQ	**<LOQ**	<LOQ	**<LOQ**	<LOQ	**<LOQ**	<LOQ	**0.17**	0.42	**0.68**	0.37
**raw Liq, LA Type 3 (#78)**	**0.22**	0.12	**0.03**	0.03	**4.24**	2.12	**25**	2.53	**<LOQ**	<LOQ	**<LOQ**	<LOQ	**18.3**	1.72	**0.08**	0.19	**0.09**	0.21	**<LOQ**	<LOQ	**<LOQ**	<LOQ	**<LOQ**	<LOQ	**1.26**	0.17
**F Liq, WA Type 2 (#50)**	**4.31**	0.32	**0.28**	0.06	**3.57**	0.89	**15.6**	8.31	**<LOQ**	<LOQ	**<LOQ**	<LOQ	**39.4**	4.65	**0.23**	0.19	**0.53**	0.15	**<LOQ**	<LOQ	**<LOQ**	<LOQ	**1.4**	0.37	**0.14**	0.35
**F Nibs U, WA Type 2a (#31)**	**2.38**	0.8	**1**	0.09	**1.18**	0.09	**2.72**	0.52	**<LOQ**	<LOQ	**2.83**	1.4	**139**	22.8	**<LOQ**	<LOQ	**0.7**	0.34	**<LOQ**	<LOQ	**<LOQ**	<LOQ	**1.25**	0.14	**<LOQ**	<LOQ
**F Nibs U, WA Type 2b (#32)**	**11.3**	2.55	**0.23**	0.07	**0.99**	0.06	**2.72**	0.32	**<LOQ**	<LOQ	**2.73**	0.17	**37.8**	4.48	**<LOQ**	<LOQ	**0.41**	0.32	**<LOQ**	<LOQ	**<LOQ**	<LOQ	**1.5**	0.2	**0.08**	0.19
**F Nibs U, WA Type 2c (#33)**	**9.72**	0.87	**0.34**	0.03	**1.2**	0.09	**6.11**	0.92	**<LOQ**	<LOQ	**3.5**	0.31	**43.4**	3.39	**<LOQ**	<LOQ	**0.66**	0.33	**<LOQ**	<LOQ	**<LOQ**	<LOQ	**1.47**	0.16	**0.21**	0.32
**F Nibs U, LA Type 1a (#28)**	**0.23**	0.05	**0.12**	0.02	**1.24**	0.05	**5.54**	0.42	**<LOQ**	<LOQ	**<LOQ**	<LOQ	**17.6**	1.25	**<LOQ**	<LOQ	**0.65**	0.33	**<LOQ**	<LOQ	**<LOQ**	<LOQ	**0.14**	0.35	**<LOQ**	<LOQ
**F Nibs U, LA Type 1b (#29)**	**0.29**	0.04	**0.13**	0.06	**1.17**	0.05	**6.32**	0.85	**0.15**	0.36	**2.72**	1.34	**29.5**	3.63	**<LOQ**	<LOQ	**0.12**	0.3	**<LOQ**	<LOQ	**<LOQ**	<LOQ	**<LOQ**	<LOQ	**<LOQ**	<LOQ
**F Nibs U, LA Type 1c (#30)**	**0.26**	0.11	**0.12**	0.07	**1.41**	0.18	**4.61**	0.73	**<LOQ**	<LOQ	**3.32**	1.7	**28.5**	3.93	**<LOQ**	<LOQ	**<LOQ**	<LOQ	**<LOQ**	<LOQ	**<LOQ**	<LOQ	**<LOQ**	<LOQ	**<LOQ**	<LOQ
**F Liq, WA Type 2a (#43)**	**6.65**	0.38	**0.66**	0.05	**0.76**	0.14	**1.85**	0.47	**<LOQ**	<LOQ	**1.08**	0.26	**56**	12	**<LOQ**	<LOQ	**0.15**	0.12	**<LOQ**	<LOQ	**<LOQ**	<LOQ	**0.97**	0.1	**0.04**	0.1
**F Liq, WA Type 2b (#44)**	**6.43**	0.61	**0.88**	0.07	**0.82**	0.15	**2.31**	0.58	**<LOQ**	<LOQ	**1.67**	0.26	**85.1**	18.8	**<LOQ**	<LOQ	**0.15**	0.12	**<LOQ**	<LOQ	**<LOQ**	<LOQ	**0.92**	0.2	**<LOQ**	<LOQ
**F Liq, WA Type 2c (#45)**	**5.69**	0.33	**0.6**	0.1	**0.65**	0.34	**2.35**	0.43	**0.05**	0.13	**1.3**	0.27	**65.3**	11.1	**<LOQ**	<LOQ	**0.22**	0.02	**<LOQ**	<LOQ	**<LOQ**	<LOQ	**0.97**	0.19	**<LOQ**	<LOQ
**F Liq, LA Type 1a (#40)**	**0.35**	0.09	**0.21**	0.03	**0.94**	0.2	**5.11**	1.01	**<LOQ**	<LOQ	**1.45**	0.35	**28**	5.46	**<LOQ**	<LOQ	**0.03**	0.08	**<LOQ**	<LOQ	**<LOQ**	<LOQ	**0.64**	0.08	**<LOQ**	<LOQ
**F Liq, LA Type 1b (#41)**	**0.29**	0.06	**0.14**	0.06	**0.87**	0.16	**3.8**	0.59	**<LOQ**	<LOQ	**1.27**	0.26	**19.9**	2.31	**<LOQ**	<LOQ	**<LOQ**	<LOQ	**<LOQ**	<LOQ	**<LOQ**	<LOQ	**0.43**	0.24	**<LOQ**	<LOQ
**F Liq, LA Type 1c (#42)**	**0.27**	0.06	**0.15**	0.04	**0.92**	0.12	**4.72**	0.64	**<LOQ**	<LOQ	**1.33**	0.21	**22**	3.36	**<LOQ**	<LOQ	**0.05**	0.12	**<LOQ**	<LOQ	**<LOQ**	<LOQ	**0.61**	0.11	**<LOQ**	<LOQ
**raw Choc U, LA Type 1 (#80)**	**0.2**	0.06	**0.02**	0.02	**2.88**	0.92	**8.71**	3.87	**1.08**	0.62	**1.7**	2.65	**13.1**	3.82	**<LOQ**	<LOQ	**<LOQ**	<LOQ	**<LOQ**	<LOQ	**<LOQ**	<LOQ	**<LOQ**	<LOQ	**1.16**	0.3
**F Choc U, LA Type 1 (#82)**	**0.48**	0.06	**0.25**	0.05	**3.89**	0.97	**15.3**	3.99	**0.74**	0.81	**5.24**	0.83	**69.9**	4.26	**0.18**	0.43	**0.57**	0.48	**<LOQ**	<LOQ	**<LOQ**	<LOQ	**<LOQ**	<LOQ	**1.39**	0.34
**raw Choc R, LA Type 1 (#81)**	**0.24**	0.04	**0.06**	0.01	**2.66**	0.62	**8.38**	2.08	**0.73**	0.6	**1.54**	1.7	**14.6**	1.34	**<LOQ**	<LOQ	**0.09**	0.21	**<LOQ**	<LOQ	**<LOQ**	<LOQ	**0.36**	0.28	**0.96**	0.21
**F Choc R, LA Type 1 (#83)**	**0.37**	0.06	**0.2**	0.06	**2.87**	0.74	**9.5**	2.89	**1.19**	0.21	**3.85**	0.79	**52.5**	6.87	**0.34**	0.38	**0.31**	0.26	**<LOQ**	<LOQ	**<LOQ**	<LOQ	**0.56**	0.31	**0.93**	0.23
**Choc #1 (#1)**	**2**	0.22	**0.15**	0.05	**0.77**	1.19	**7.2**	1.46	**<LOQ**	<LOQ	**<LOQ**	<LOQ	**25.1**	0.86	**<LOQ**	<LOQ	**<LOQ**	<LOQ	**<LOQ**	<LOQ	**<LOQ**	<LOQ	**0.29**	0.32	**<LOQ**	<LOQ
**Choc #2 (#2)**	**2.85**	0.43	**0.23**	0.04	**0.43**	1.05	**8.4**	1.81	**<LOQ**	<LOQ	**2.3**	2.63	**31.7**	5.24	**<LOQ**	<LOQ	**<LOQ**	<LOQ	**<LOQ**	<LOQ	**<LOQ**	<LOQ	**0.4**	0.47	**<LOQ**	<LOQ
**Choc #3 (#3)**	**2.48**	0.52	**0.2**	0.06	**1.23**	1.59	**7.39**	2.36	**0.14**	0.34	**1.8**	2.2	**30.6**	5.9	**<LOQ**	<LOQ	**<LOQ**	<LOQ	**<LOQ**	<LOQ	**<LOQ**	<LOQ	**0.45**	0.38	**<LOQ**	<LOQ
**Choc #4 (#4)**	**1.26**	0.24	**0.19**	0.04	**0.7**	1.08	**6.1**	1.28	**<LOQ**	<LOQ	**1.5**	2.33	**27.3**	4.09	**<LOQ**	<LOQ	**<LOQ**	<LOQ	**<LOQ**	<LOQ	**<LOQ**	<LOQ	**0.11**	0.27	**<LOQ**	<LOQ
**Choc #5 (#5)**	**2.98**	0.33	**0.17**	0.03	**1.38**	1.16	**5.08**	0.77	**<LOQ**	<LOQ	**1.94**	1.53	**21.8**	1.51	**<LOQ**	<LOQ	**<LOQ**	<LOQ	**<LOQ**	<LOQ	**<LOQ**	<LOQ	**0.49**	0.24	**<LOQ**	<LOQ
**Choc #6 (#6)**	**1.86**	0.08	**0.21**	0.04	**3.34**	1.92	**10.2**	1.05	**<LOQ**	<LOQ	**3.01**	3.36	**41.7**	1.83	**<LOQ**	<LOQ	**<LOQ**	<LOQ	**<LOQ**	<LOQ	**<LOQ**	<LOQ	**0.12**	0.29	**<LOQ**	<LOQ
**Liq Choc #6 (#7)**	**2.26**	0.26	**0.24**	0.07	**1.96**	1.11	**6.92**	0.75	**<LOQ**	<LOQ	**4.36**	0.49	**33.4**	2	**<LOQ**	<LOQ	**0.14**	0.23	**<LOQ**	<LOQ	**<LOQ**	<LOQ	**0.42**	0.32	**<LOQ**	<LOQ

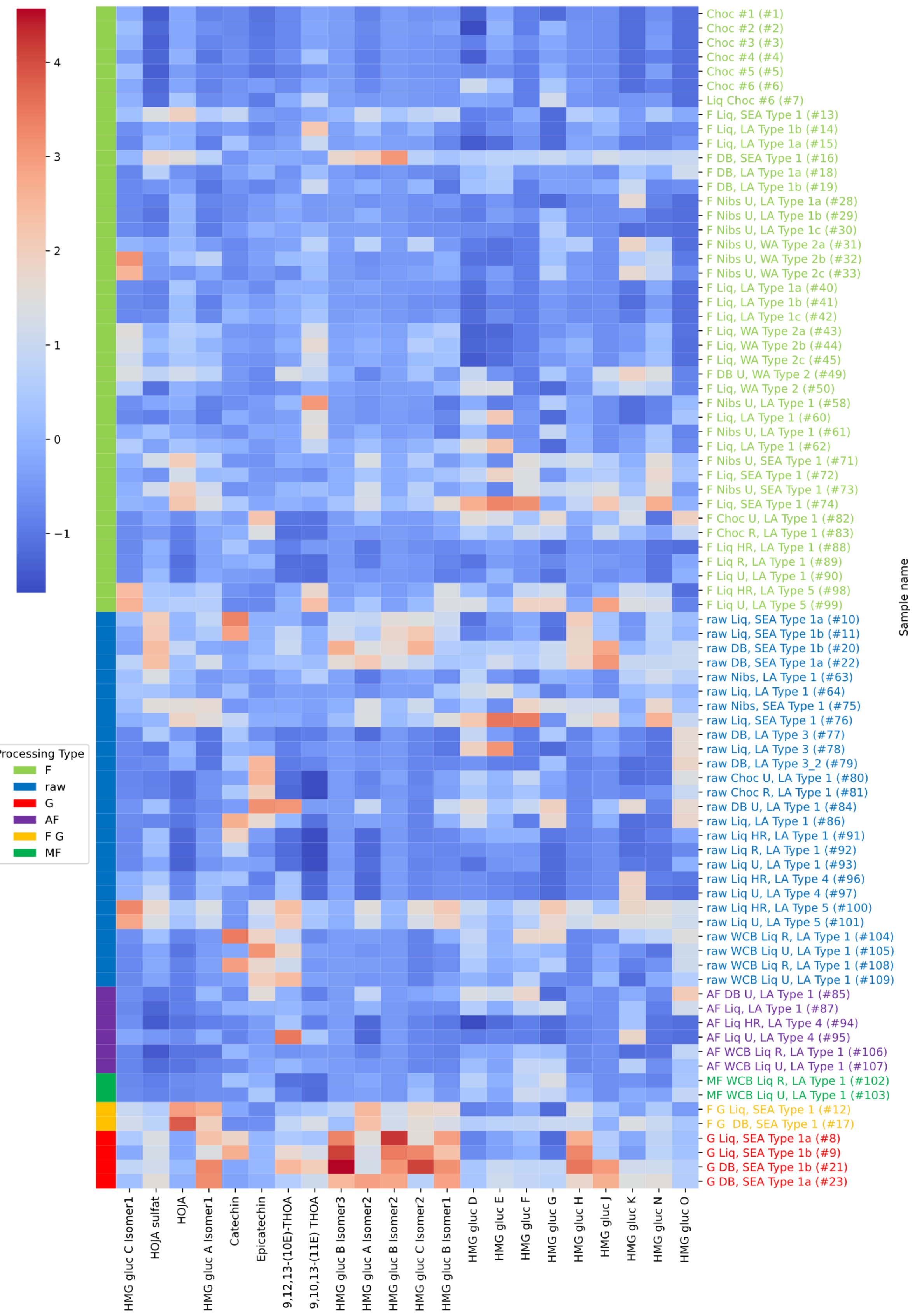

Figure S 6: Heatmap of mean values (six measured replicates) of contents ω of all measured marker candidates in all samples, arranged by process type: Fermented (F; in light green), raw (blue), germinated (G; red), alternatively fermented (AF; purple), combination of fermentation and germination (F G; orange), and micro-fermented (MF; dark green). Contents are standardized by z-score, (high concentrations in red, low concentrations in blue according to color scale.

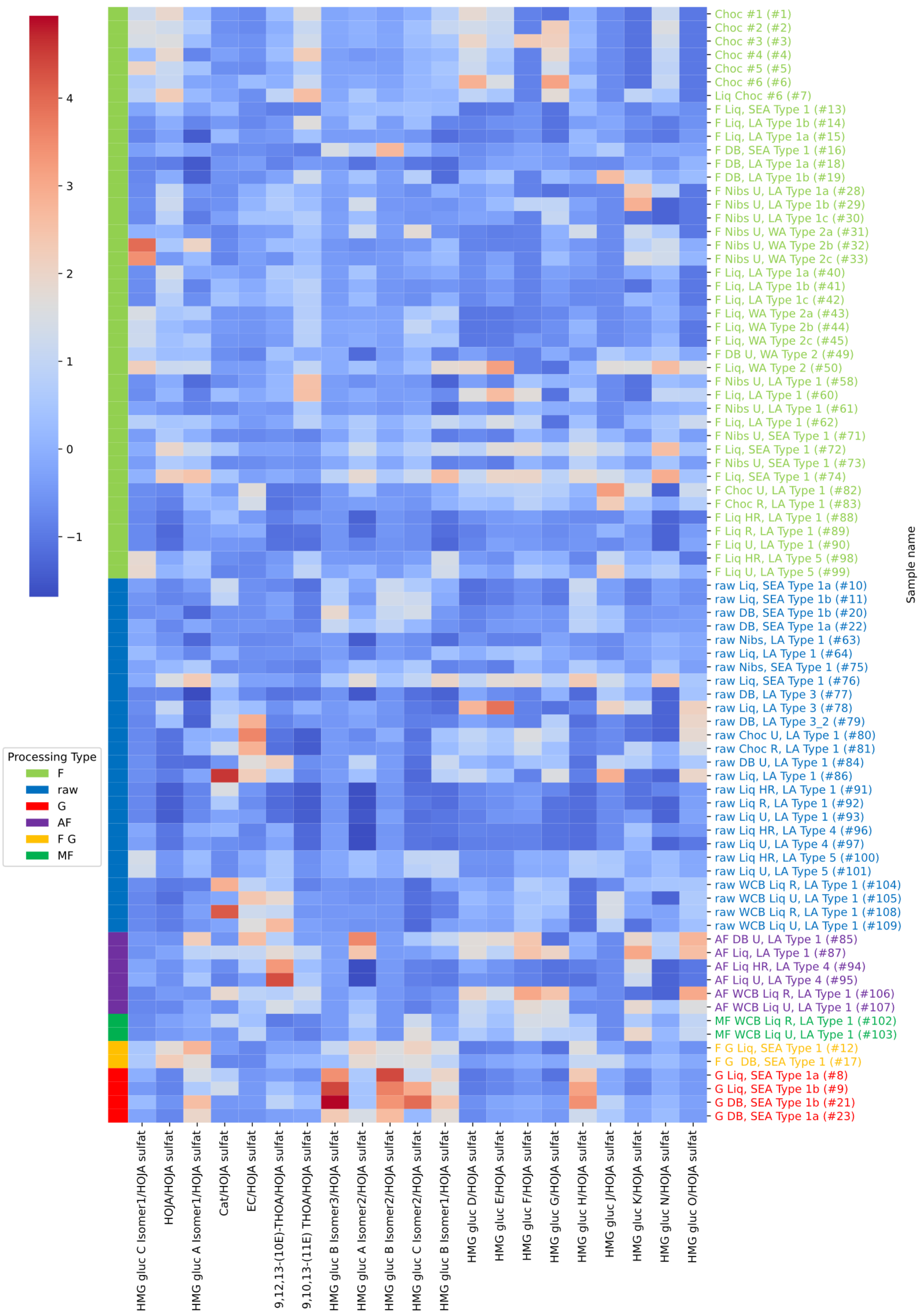

Figure S 7: Heatmap of mean values (six measured replicates) of content ratios $\omega_{analyte}/\omega_{HOJA\ sulfate}$ of all measured marker candidates in all samples, arranged by process type: Fermented (F; in light green), raw (blue), germinated (G; red), alternatively fermented (AF; purple), combination of fermentation and germination (F G; orange), and micro-fermented (MF; dark green). Contents are standardized by z-score, (high concentrations in red, low concentrations in blue according to color scale).

Boxplots: Concentrations (mg/kg) of promising marker compounds

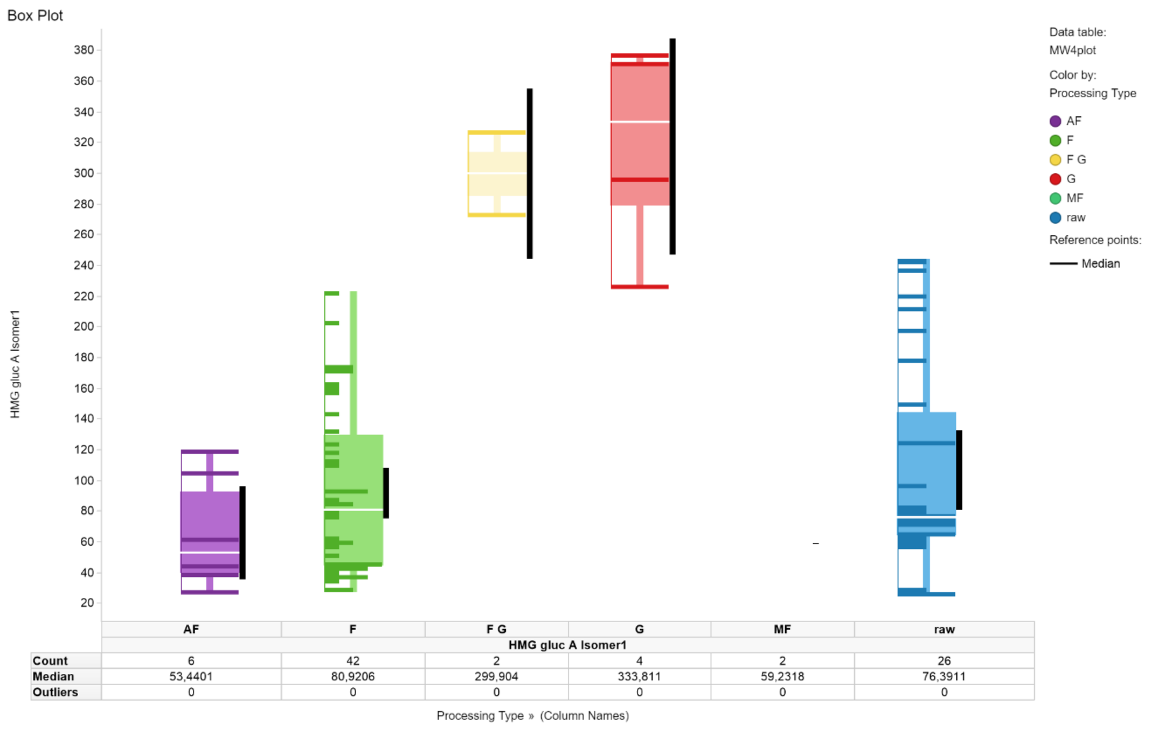

Figure S 8: Boxplot of mean values of $\omega_{HMG\ gluc\ A\ Isomer\ 1}$, highlighting the distribution of samples grouped by process type: Fermented (F; in light green), raw (blue), germinated (G; red), alternatively fermented (AF; purple), combination of fermentation and germination (F G; orange), and micro-fermented (MF; dark green). The black bar marks a confidence interval on a level of 95%.

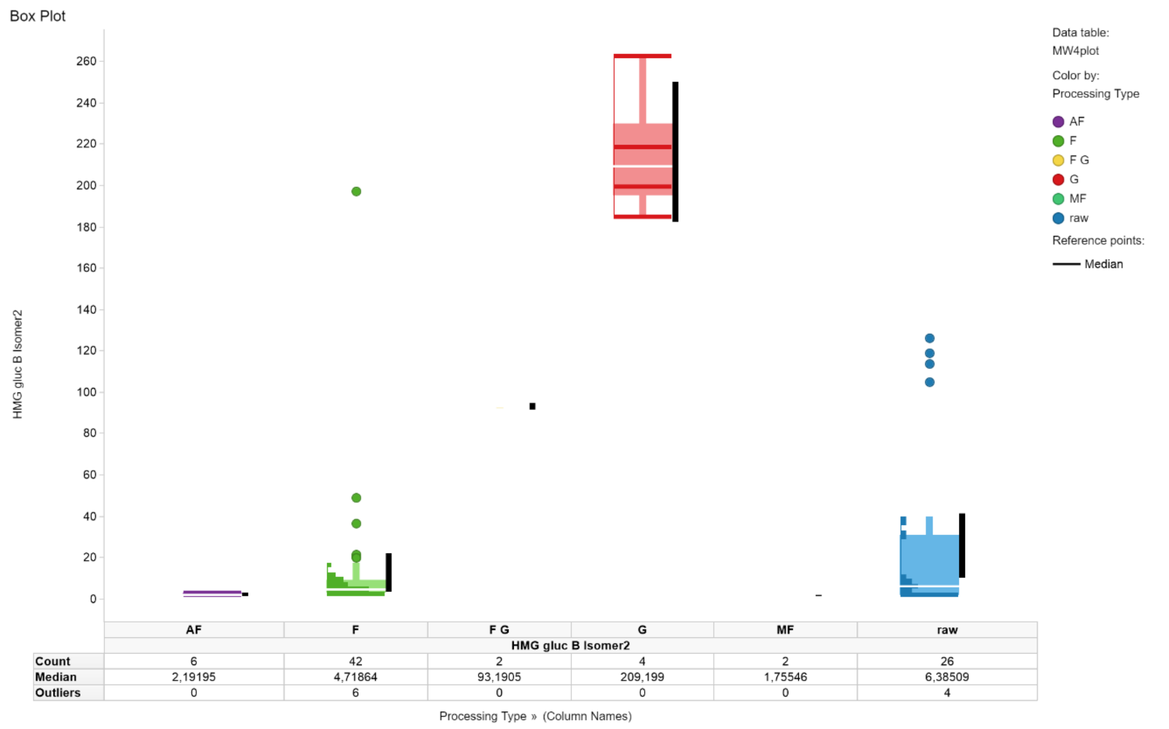

Figure S 9: Boxplot of mean values of $\omega_{HMG\ gluc\ B\ Isomer\ 2}$, highlighting the distribution of samples grouped by process type: Fermented (F; in light green), raw (blue), germinated (G; red), alternatively fermented (AF; purple), combination of fermentation and germination (F G; orange), and micro-fermented (MF; dark green). The black bar marks a confidence interval on a level of 95%.

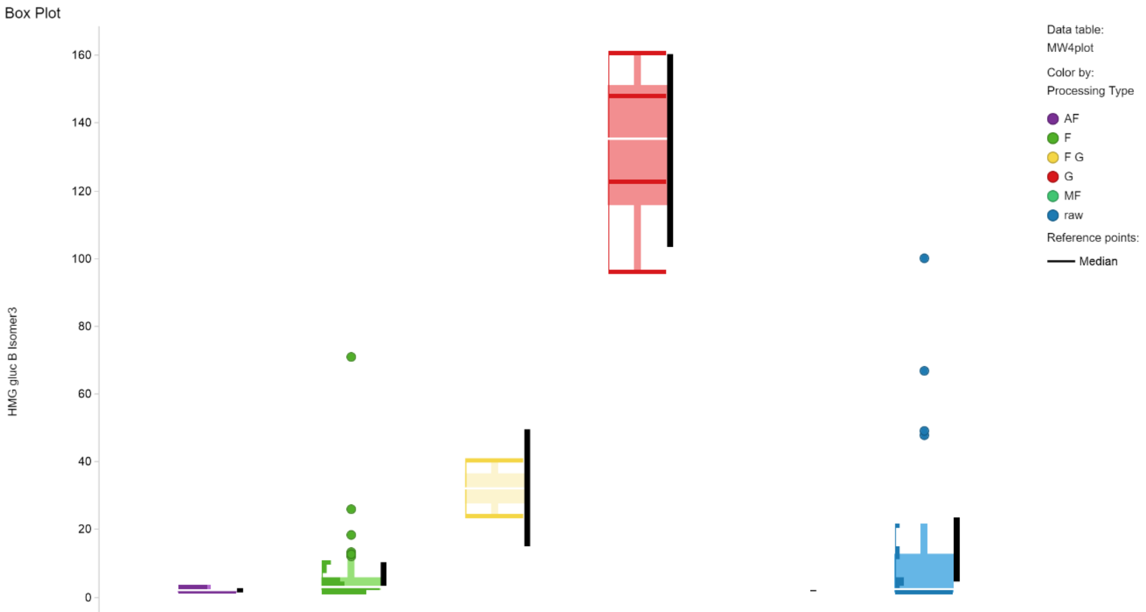

	AF	F	F G	G	MF	raw
	HMG gluc B Isomer3					
**Count**	6	42	2	4	2	26
**Median**	2,00429	3,00859	32,2252	135,231	1,89231	2,5048
**Outliers**	0	6	0	0	0	4

Processing Type » (Column Names)

Figure S 10: Boxplot of mean values of $\omega_{HMG\ gluc\ B\ Isomer\ 3}$, highlighting the distribution of samples grouped by process type: Fermented (F; in light green), raw (blue), germinated (G; red), alternatively fermented (AF; purple), combination of fermentation and germination (F G; orange), and micro-fermented (MF; dark green). The black bar marks a confidence interval on a level of 95%.

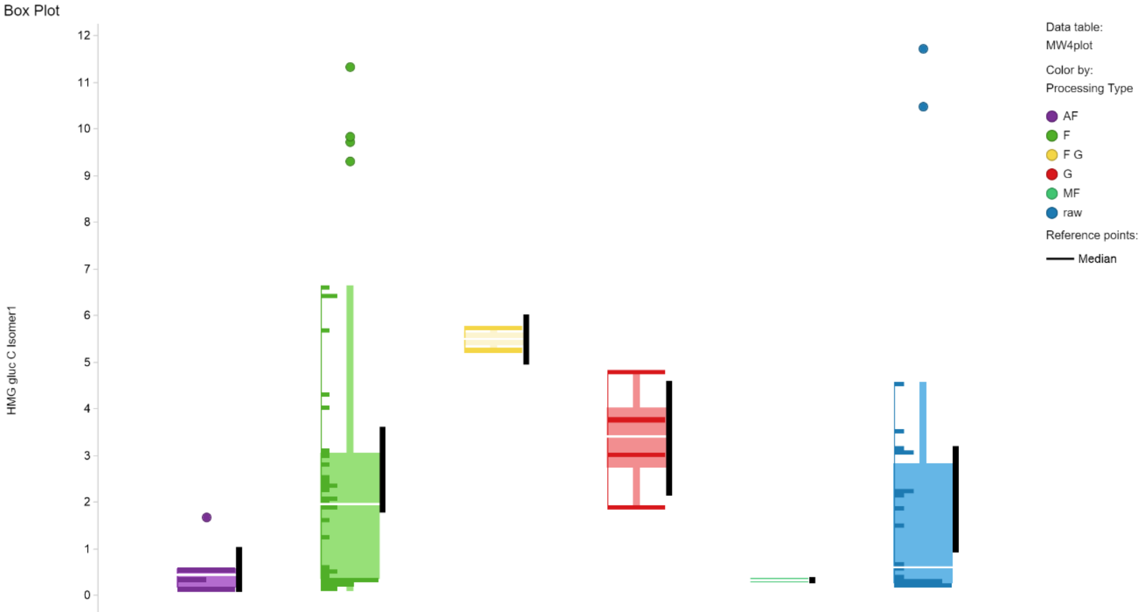

	AF	F	F G	G	MF	raw
	HMG gluc C Isomer1					
**Count**	6	42	2	4	2	26
**Median**	0,433991	1,96262	5,48615	3,40416	0,323601	0,609695
**Outliers**	1	4	0	0	0	2

Processing Type » (Column Names)

Figure S 11: Boxplot of mean values of $\omega_{HMG\ gluc\ C\ Isomer\ 1}$, highlighting the distribution of samples grouped by process type: Fermented (F; in light green), raw (blue), germinated (G; red), alternatively fermented (AF; purple), combination of fermentation and germination (F G; orange), and micro-fermented (MF; dark green). The black bar marks a confidence interval on a level of 95%.

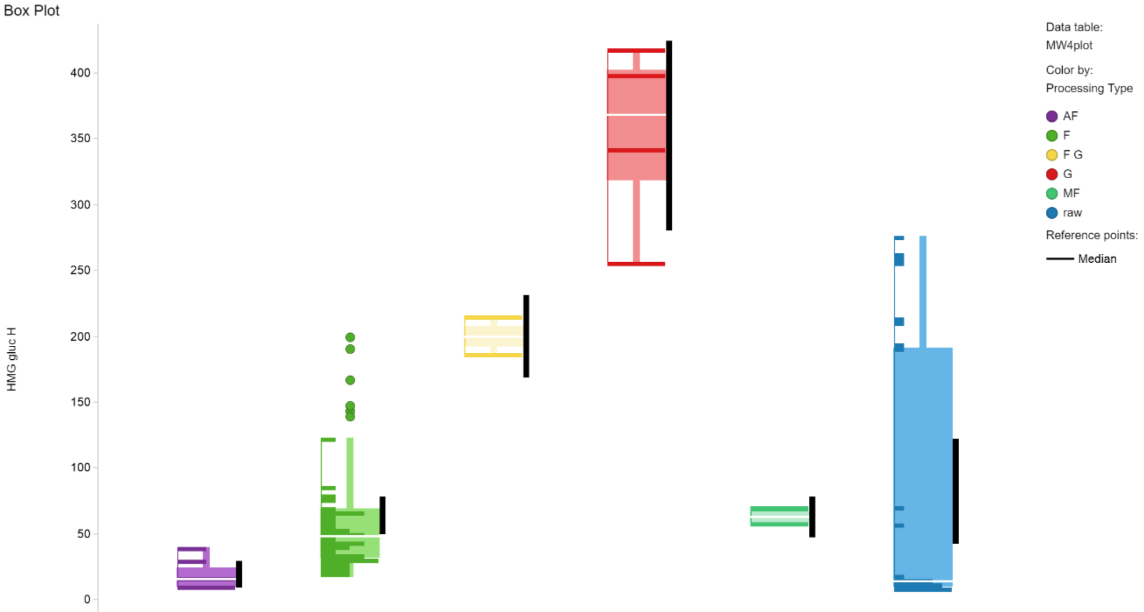

	AF	F	F G	G	MF	raw
	HMG gluc H					
**Count**	6	42	2	4	2	26
**Median**	15,317	48,4934	199,977	368,604	62,9184	13,6662
**Outliers**	0	6	0	0	0	0

Processing Type » (Column Names)

Figure S 12: Boxplot of mean values of $\omega_{HMG\ gluc\ H}$, highlighting the distribution of samples grouped by process type: Fermented (F; in light green), raw (blue), germinated (G; red), alternatively fermented (AF; purple), combination of fermentation and germination (F G; orange), and micro-fermented (MF; dark green). The black bar marks a confidence interval on a level of 95%.

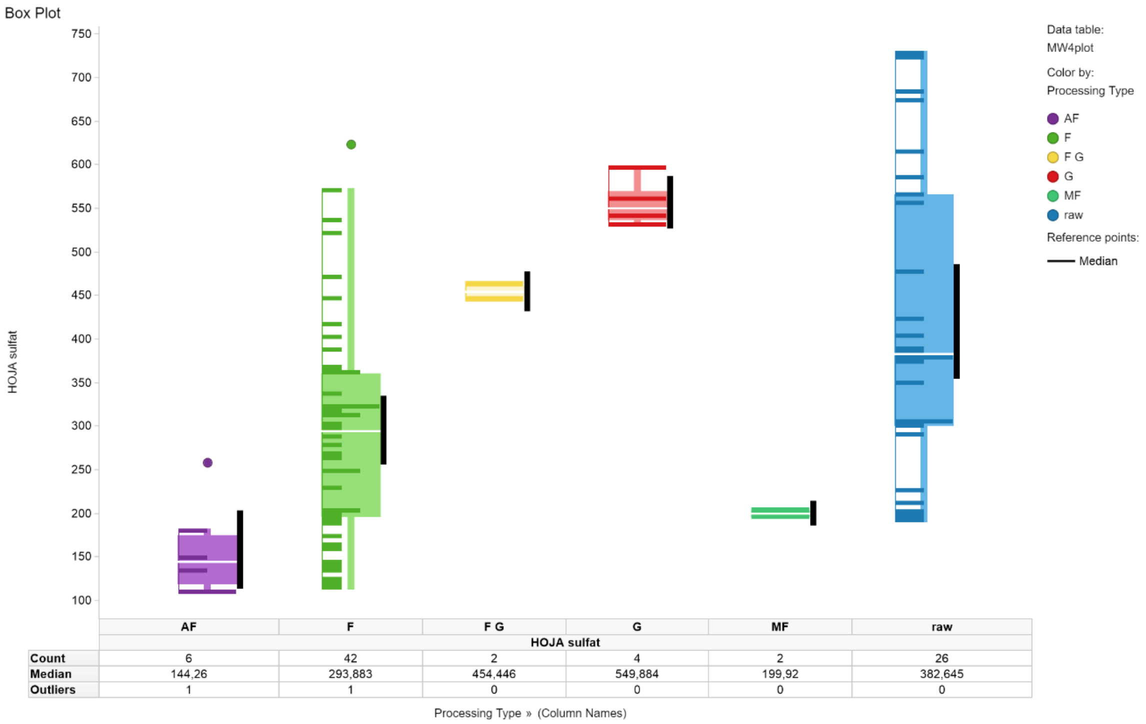

Figure S 13: Boxplot of mean values of $\omega_{HOJA\ sulfate}$, highlighting the distribution of samples grouped by process type: Fermented (F; in light green), raw (blue), germinated (G; red), alternatively fermented (AF; purple), combination of fermentation and germination (F G; orange), and micro-fermented (MF; dark green). The black bar marks a confidence interval on a level of 95%.

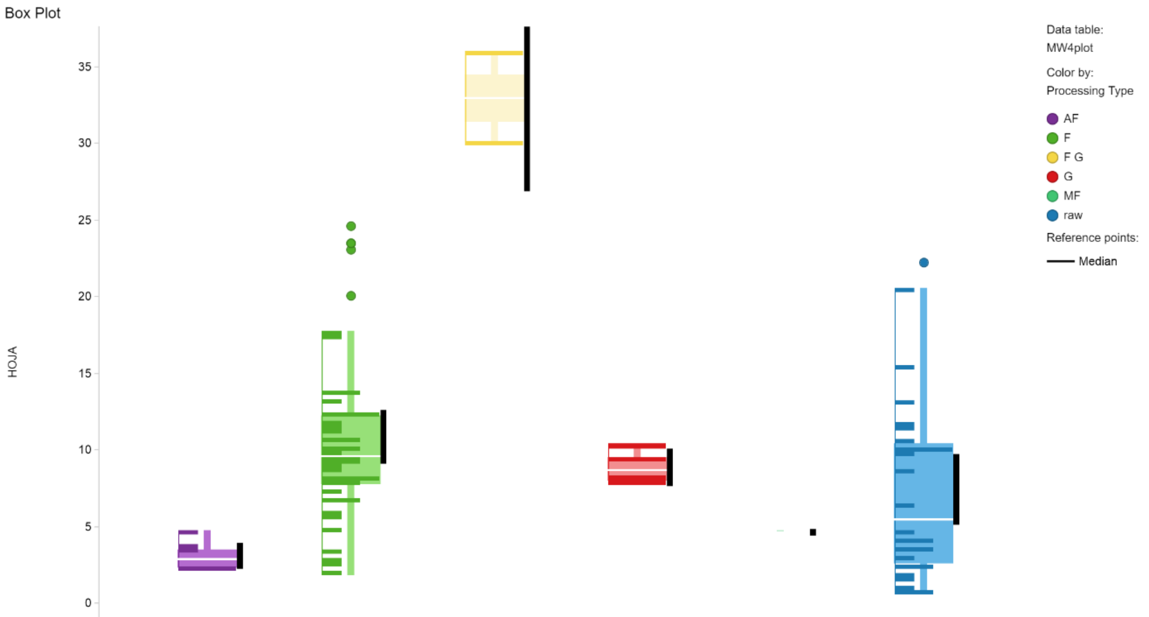

	AF	F	F G	G	MF	raw
**Count**	6	42	2	4	2	26
**Median**	2,90239	9,58265	32,9447	8,66634	4,66446	5,47539
**Outliers**	0	5	0	0	0	1

Figure S 14: Boxplot of mean values of ω_{HOJA}, highlighting the distribution of samples grouped by process type: Fermented (F; in light green), raw (blue), germinated (G; red), alternatively fermented (AF; purple), combination of fermentation and germination (F G; orange), and micro-fermented (MF; dark green). The black bar marks a confidence interval on a level of 95%.

Boxplots: concentration ratios (ω/ω) of promising marker compounds

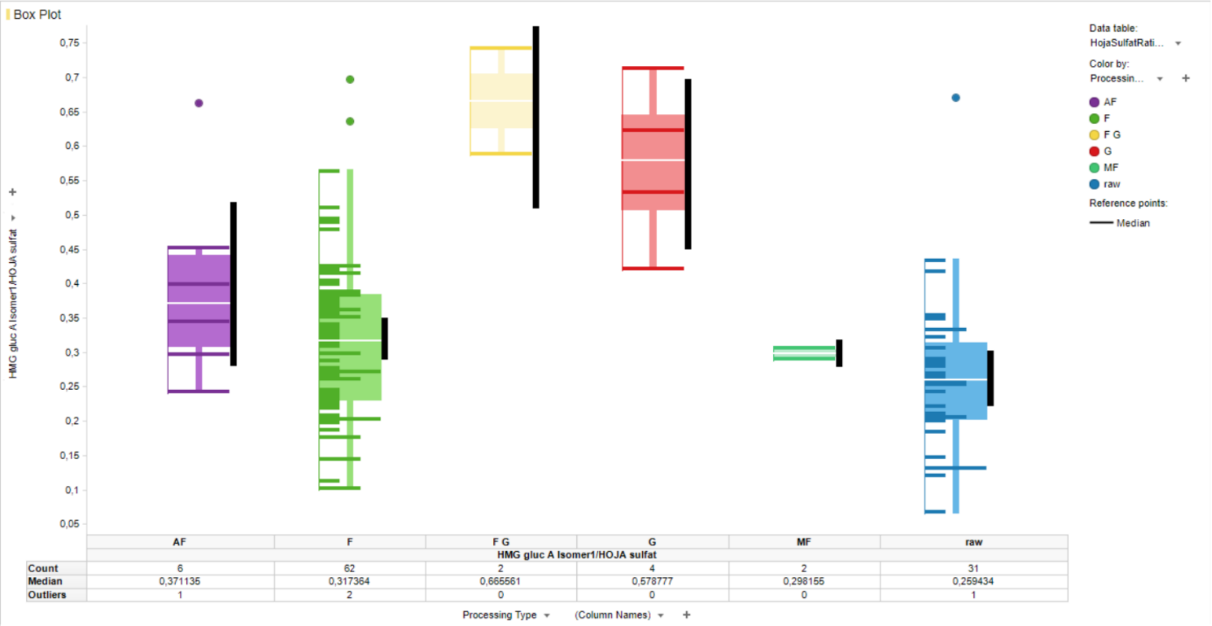

Figure S 15: Boxplot of mean values of content ratios $\omega_{HMG\ gluc\ A\ Isomer\ 1}/\omega_{HOJA\ sulfate}$, highlighting the distribution of samples grouped by process type: Fermented (F; in light green), raw (blue), germinated (G; red), alternatively fermented (AF; purple), combination of fermentation and germination (F G; orange), and micro-fermented (MF; dark green). The black bar marks a confidence interval on a level of 95%.

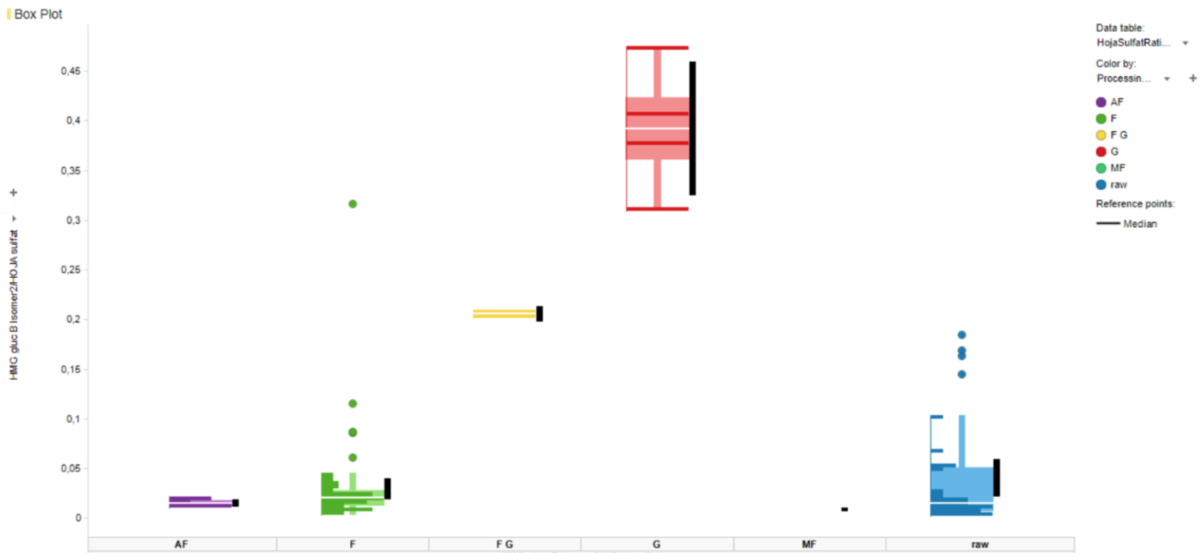

	AF	F	F G	G	MF	raw
	HMG gluc B Isomer2/HOJA sulfat					
**Count**	6	62	2	4	2	31
**Median**	0,0150399	0,0210458	0,205656	0,391756	0,00883874	0,0148395
**Outliers**	0	5	0	0	0	4

Processing Type (Column Names) +

Figure S 16: Boxplot of mean values of content ratios $\omega_{HMG\ gluc\ B\ Isomer\ 2}/\omega_{HOJA\ sulfate}$, highlighting the distribution of samples grouped by process type: Fermented (F; in light green), raw (blue), germinated (G; red), alternatively fermented (AF; purple), combination of fermentation and germination (F G; orange), and micro-fermented (MF; dark green). The black bar marks a confidence interval on a level of 95%.

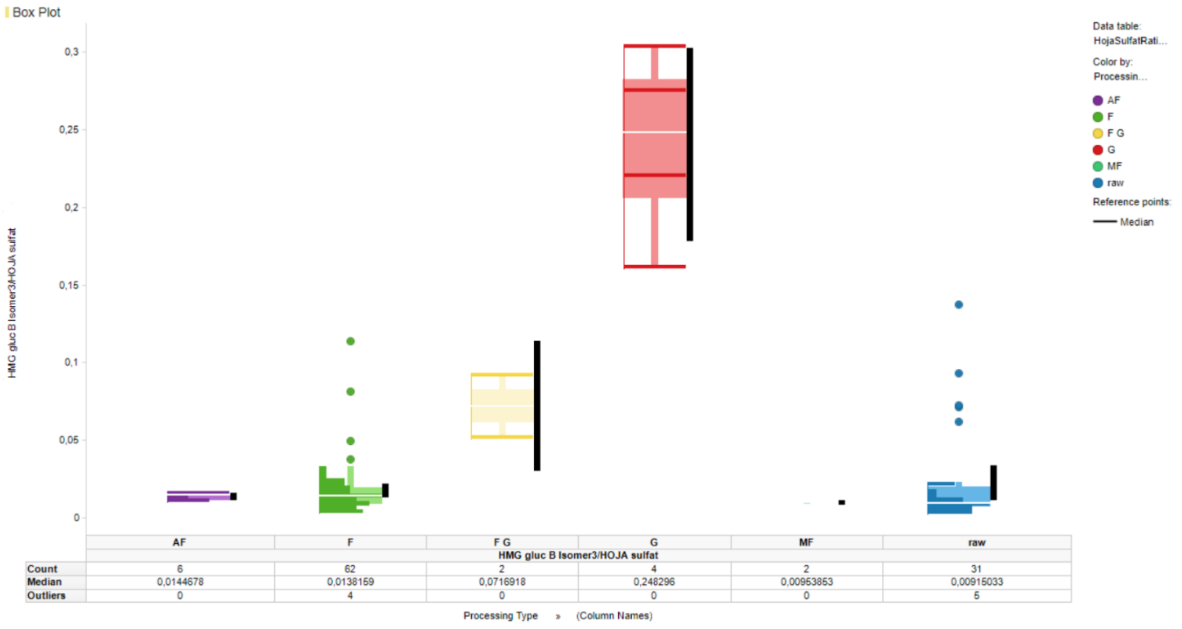

Figure S 17: Boxplot of mean values of content ratios $\omega_{HMG\ gluc\ B\ Isomer\ 3}/\omega_{HOJA\ sulfate}$, highlighting the distribution of samples grouped by process type: Fermented (F; in light green), raw (blue), germinated (G; red), alternatively fermented (AF; purple), combination of fermentation and germination (F G; orange), and micro-fermented (MF; dark green). The black bar marks a confidence interval on a level of 95%.

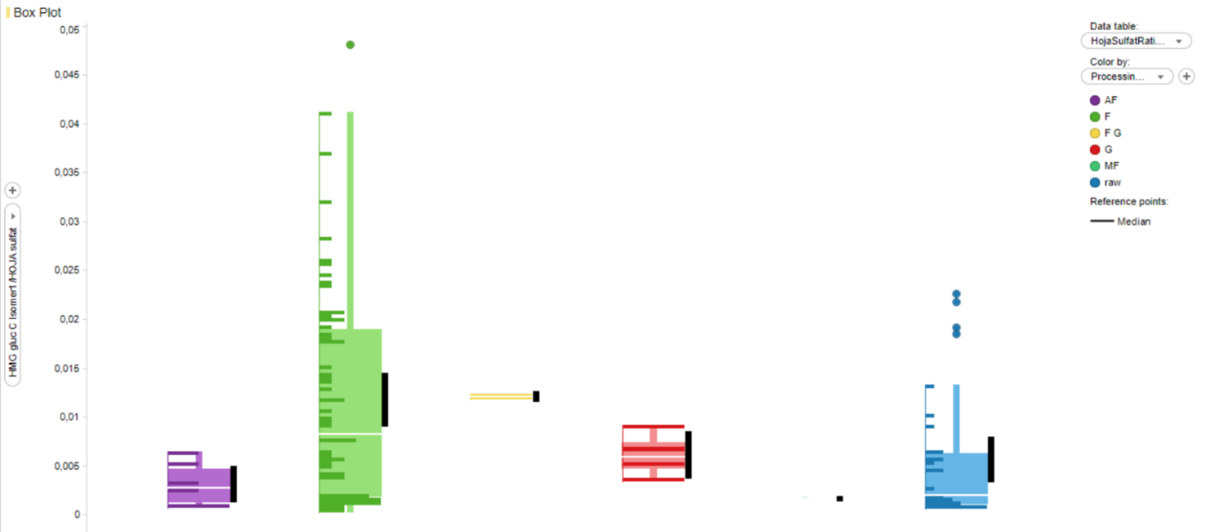

	AF	F	F G	G	MF	raw
	HMG gluc C Isomer1/HOJA sulfat					
**Count**	6	62	2	4	2	31
**Median**	0,00268631	0,00823417	0,0120957	0,0059343	0,00162762	0,00201032
**Outliers**	0	1	0	0	0	4

Processing Type (Column Names)

Figure S 18: Boxplot of mean values of content ratios $\omega_{HMG\ gluc\ C\ Isomer\ 1}/\omega_{HOJA\ sulfate}$, highlighting the distribution of samples grouped by process type: Fermented (F; in light green), raw (blue), germinated (G; red), alternatively fermented (AF; purple), combination of fermentation and germination (F G; orange), and micro-fermented (MF; dark green). The black bar marks a confidence interval on a level of 95%.

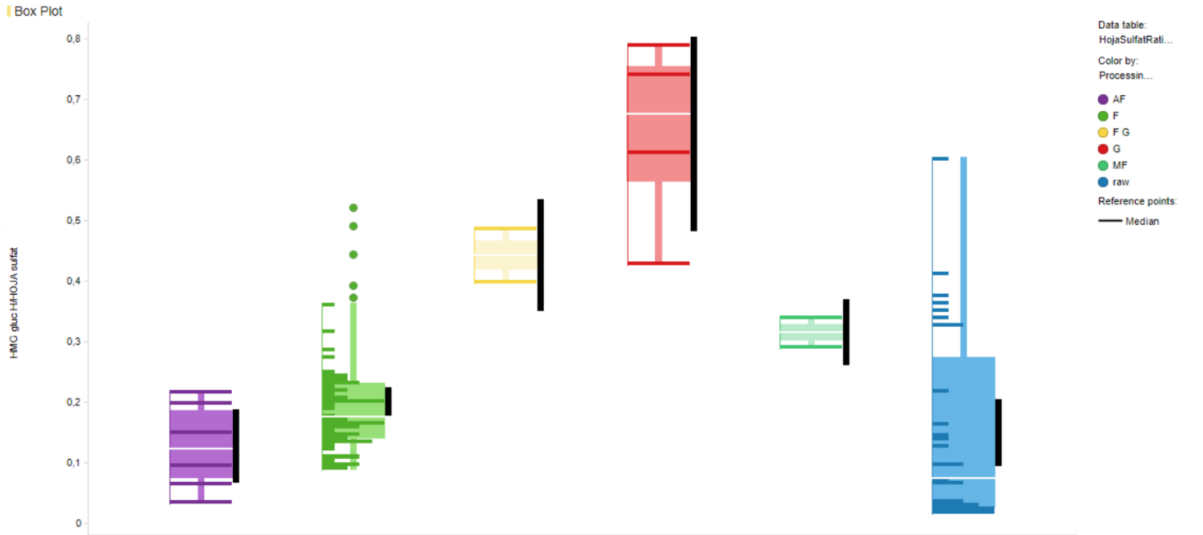

	AF	F	F G	G	MF	raw
	HMG gluc H/HOJA sulfat					
Count	6	62	2	4	2	31
Median	0,123222	0,176574	0,44292	0,676084	0,315582	0,0745013
Outliers	0	5	0	0	0	0

Processing Type » (Column Names)

Figure S 19: Boxplot of mean values of content ratios $\omega_{HMG\ gluc\ H}/\omega_{HOJA\ sulfate}$, highlighting the distribution of samples grouped by processing type: Fermented (F; in light green), raw (blue), germinated (G; red), alternatively fermented (AF; purple), combination of fermentation and germination (F G; orange), and micro-fermented (MF; dark green). The black bar marks a confidence interval on a level of 95%.

Results of data analysis

Unsupervised clustering

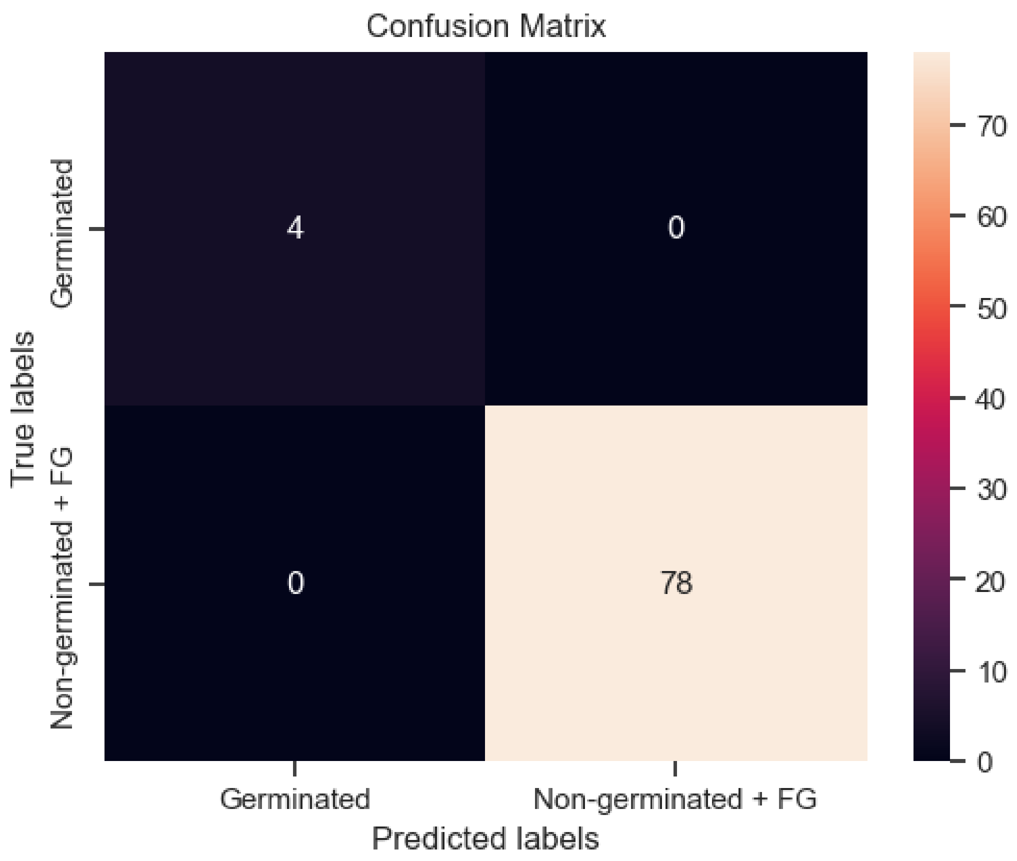

Figure S 20: Confusion matrix highlighting the fit of clusters with actual classes of processing types. All four G-type samples were clustered together, whereas both fermented and germinated (FG) samples were found as outliers.

Table S 9: Metrics of HDBSCAN, depicting the predicted clusters of germinated samples and remaining samples (Non-germinated+FG).

	precision	recall	f1-score	support
Germinated	1.00	1.00	1.00	4
Non-Germinated + FG	1.00	1.00	1.00	78
accuracy			1.00	82
macro avg	1.00	1.00	1.00	82
weighted avg	1.00	1.00	1.00	82

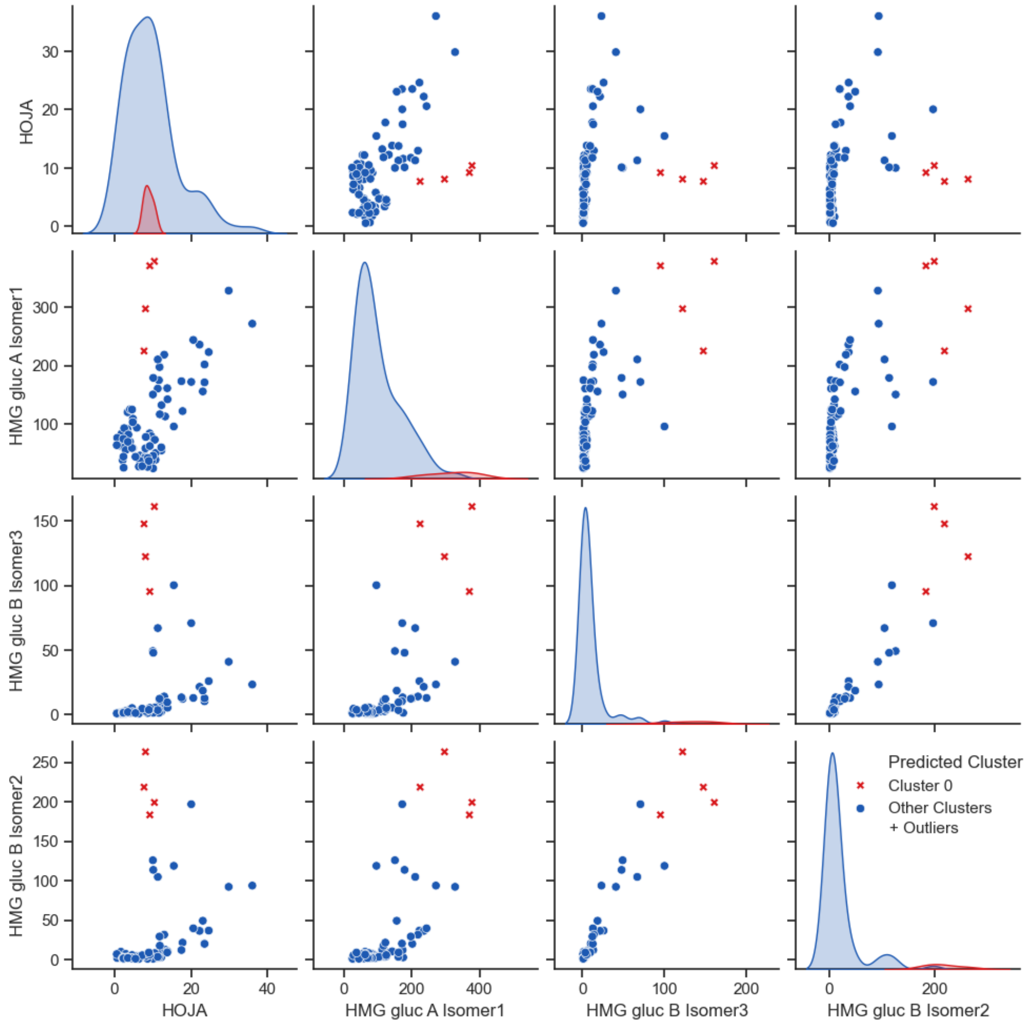

Figure S 21: Pair plot (Scatter plot matrix) of 4 selected features, visualizing the predicted unsupervised clusters from the HDBSCAN output: cluster 0 contains only germinated samples (red), while all other four clusters and outliers are colored blue.

Table S 10: Clusters and processing type counts based on PCA dimensions: Here, without supervision, the algorithm was able to put all the G-samples into one cluster (1), while, again, both the FG samples were excluded as outliers (-1).

Cluster	Processing Type	Support
-1	AF	3
	F	3
	F G	2
	raw	3
0	F	1
	raw	4
1	G	4
2	F	4
	raw	2
3	F	9
	raw	1
4	AF	2
	F	2
	raw	2
5	F	3
	raw	2
6	F	1
	raw	8
7	F	3
8	AF	1
	F	4
	MF	2
	raw	3
9	F	3
10	F	3
	raw	1
11	F	6

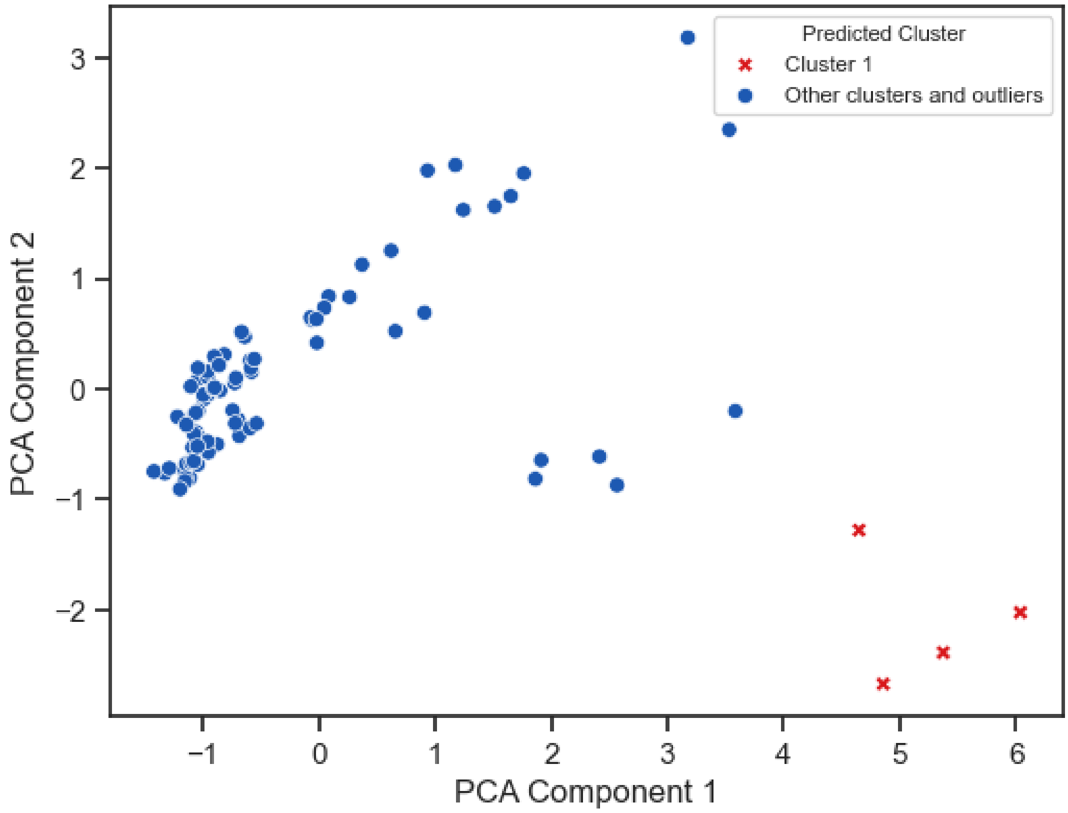

Figure S 22: Scatter plot visualizing the results of clustering by HDBSCAN after two-dimensional PCA, highlighting the clustering of germinated samples (Cluster 1, red) while all other 11 clusters and outliers are colored blue.

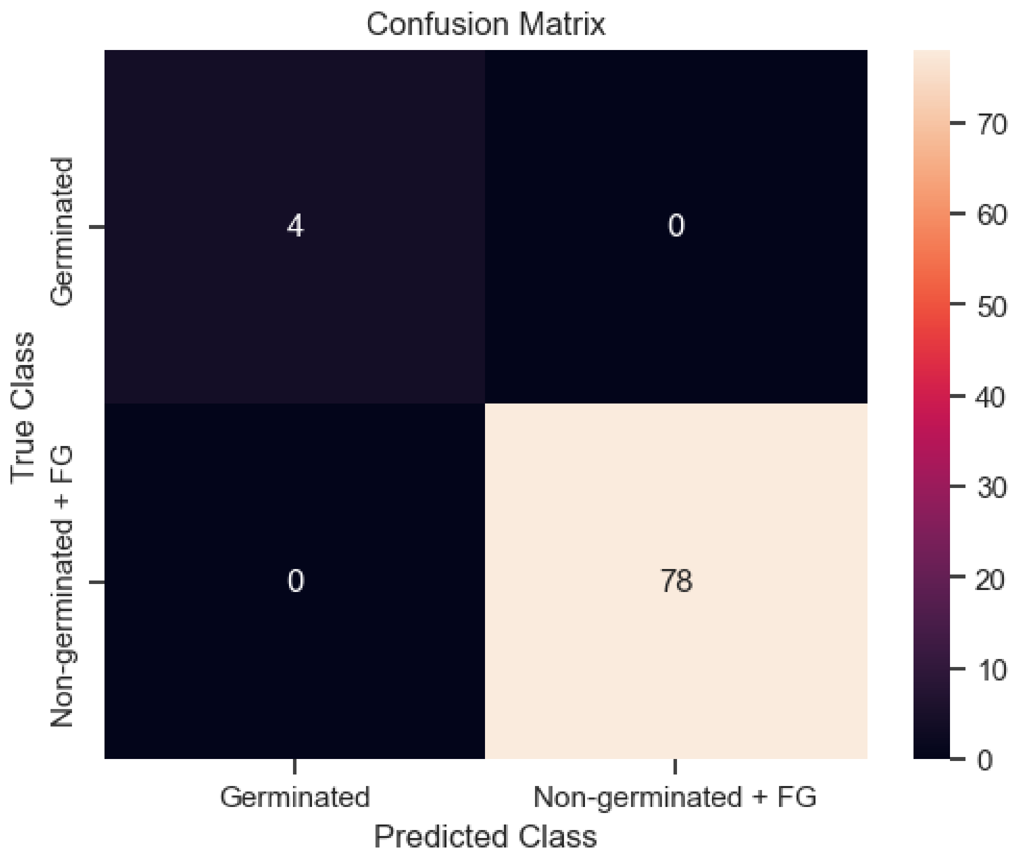

Figure S 23: Confusion matrix comparing the clustering of G-type samples with the predicted clusters (Germinated) according to the dimensions of PCA.

Table S 11: Metrics of HDBSCAN after PCA, depicting the predicted clusters of germinated samples and remaining samples (Non-germinated+FG).

	precision	recall	f1-score	support
Germinated	1.00	1.00	1.00	4
Non-Germinated + FG	1.00	1.00	1.00	78
accuracy			1.00	82
macro avg	1.00	1.00	1.00	82
weighted avg	1.00	1.00	1.00	82

Supervised classification by logistic regression

1st Classifier:

Table S 12: Logistic Regression classification results of G- and FG-type samples in the 67% training set of partition 1 of the 1st classifier with the predicted class (Germinated+FG) trained on the two PCA dimensions.

	precision	recall	f1-score	support
Germinated + FG	1.00	1.00	1.00	4
Non-Germinated	1.00	1.00	1.00	50
accuracy			1.00	54
macro avg	1.00	1.00	1.00	54
weighted avg	1.00	1.00	1.00	54

Table S 13: Logistic Regression classification results of G- and FG-type samples in the 33% test set of partition 1 of the 1st classifier predicting the target class (Germinated+FG) using the two PCA dimensions.

	precision	recall	f1-score	support
Germinated + FG	1.00	1.00	1.00	2
Non-Germinated	1.00	1.00	1.00	26
accuracy			1.00	28
macro avg	1.00	1.00	1.00	28
weighted avg	1.00	1.00	1.00	28

Table S 14: Logistic Regression classification results of G- and FG-type samples in the 67% training set of partition 2 of the 1st classifier with the predicted class (Germinated+FG) trained on the two PCA dimensions.

	precision	recall	f1-score	support
Germinated + FG	1.00	1.00	1.00	4
Non-Germinated	1.00	1.00	1.00	51
accuracy			1.00	55
macro avg	1.00	1.00	1.00	55
weighted avg	1.00	1.00	1.00	55

Table S 15: Logistic Regression classification results of G- and FG-type samples in the 33% test set of partition 2 of the 1st classifier predicting the target class (Germinated+FG) using the two PCA dimensions.

	precision	recall	f1-score	support
Germinated + FG	1.00	1.00	1.00	2
Non-Germinated	1.00	1.00	1.00	25
accuracy			1.00	27
macro avg	1.00	1.00	1.00	27
weighted avg	1.00	1.00	1.00	27

Table S 16: Logistic Regression classification results of G- and FG-type samples in the 67% training set of partition 3 of the 1st classifier with the predicted class (Germinated+FG) trained on the two PCA dimensions.

	precision	recall	f1-score	support
Germinated + FG	1.00	0.75	0.86	4
Non-germinated	0.98	1.00	0.99	51
accuracy			0.98	55
macro avg	0.99	0.88	0.92	55
weighted avg	0.98	0.98	0.98	55

Table S 17: Logistic Regression classification results of G- and FG-type samples in the 33% test set of partition 3 of the 1st classifier predicting the target class (Germinated+FG) using the two PCA dimensions.

	precision	recall	f1-score	support
Germinated + FG	1.00	1.00	1.00	2
Non-Germinated	1.00	1.00	1.00	25
accuracy			1.00	27
macro avg	1.00	1.00	1.00	27
weighted avg	1.00	1.00	1.00	27

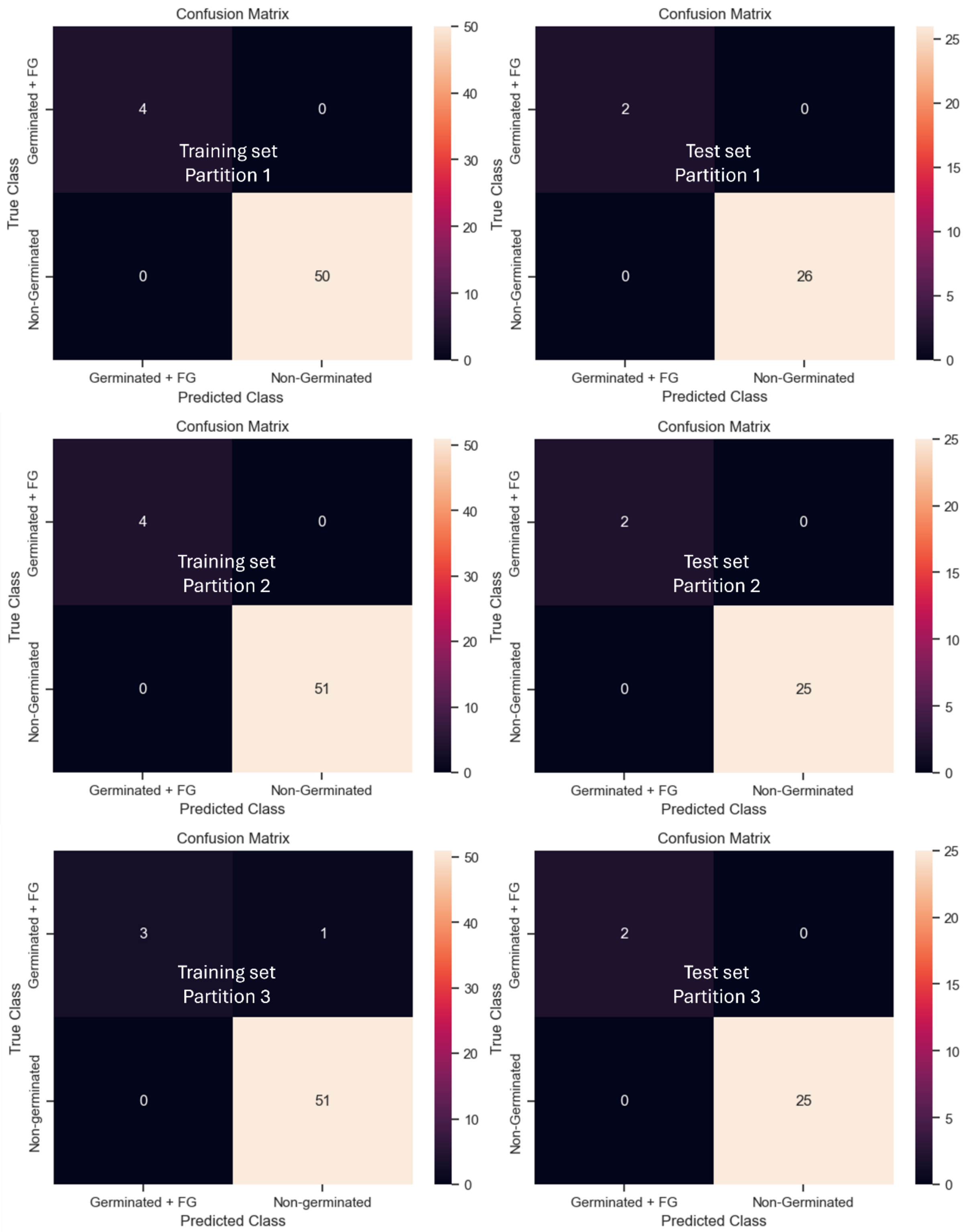

Figure S 24: Confusion matrices comparing the classification of G- and FG-type samples in the training/test sets of 3 partitions of the 1st classifier with the predicted class (Germinated+FG) trained on the two PCA dimensions. Only in the training set of partition 3 the FG sample was classified wrong.

2nd Classifier:

Training set:

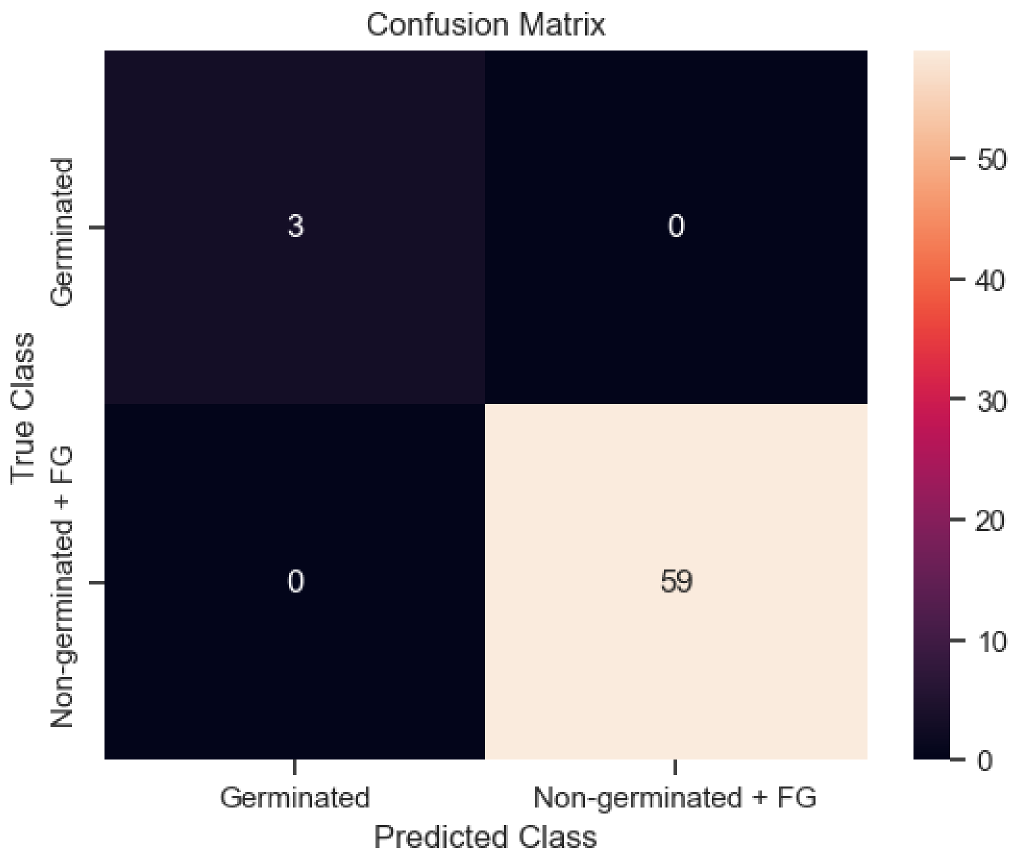

Figure S 25: Confusion matrix comparing the classification of G-type samples in the training set of partition 1 of the 2nd classifier with the predicted class (Germinated) trained on the two PCA dimensions. Confusion matrices of the training sets of partitions 2, 3 and 4 had the same outcome.

Table S 18: Logistic Regression classification results of G-type samples in the training set of partition 1 of the 2nd classifier with the predicted class (Germinated) trained on the two PCA dimensions. Classification results of the training sets of partitions 2, 3 and 4 had the same outcome.

	precision	recall	f1-score	support
Germinated	1.00	1.00	1.00	3
Non-germinated + FG	1.00	1.00	1.00	59
accuracy			1.00	62
macro avg	1.00	1.00	1.00	62
weighted avg	1.00	1.00	1.00	62

Test set:

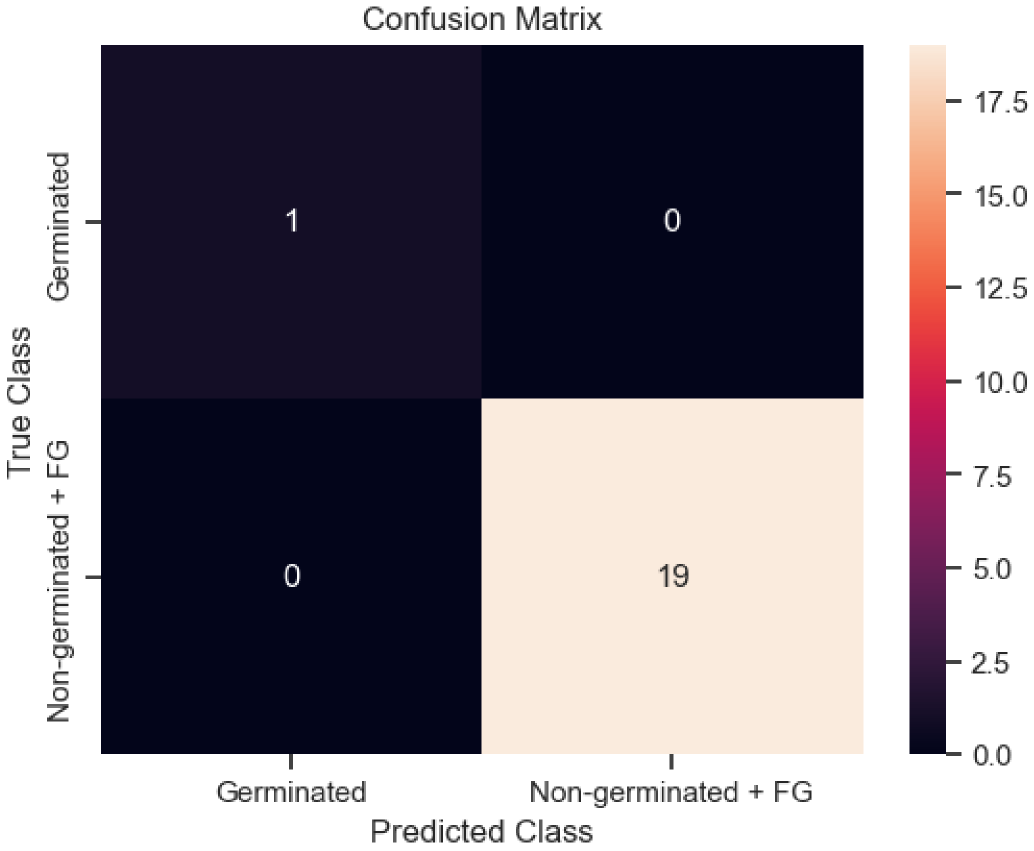

Figure S 26: Confusion matrix comparing the classification of G-type samples in the test set of partition 1 of the 2nd classifier with the predicted class (Germinated) trained on the two PCA dimensions. Confusion matrices of the test sets of partitions 2, 3 and 4 had the same outcome.

Table S 19: Logistic Regression classification results of G-type samples in the test set of partition 1 of the 2nd classifier predicting the target class (Germinated) using the two PCA dimensions. Classification results of the test sets of partitions 2, 3 and 4 had the same outcome.

	precision	recall	f1-score	support
Germinated	1.00	1.00	1.00	1
Non-germinated + FG	1.00	1.00	1.00	19
accuracy			1.00	20
macro avg	1.00	1.00	1.00	20
weighted avg	1.00	1.00	1.00	20

3rd Classifier:

Training set:

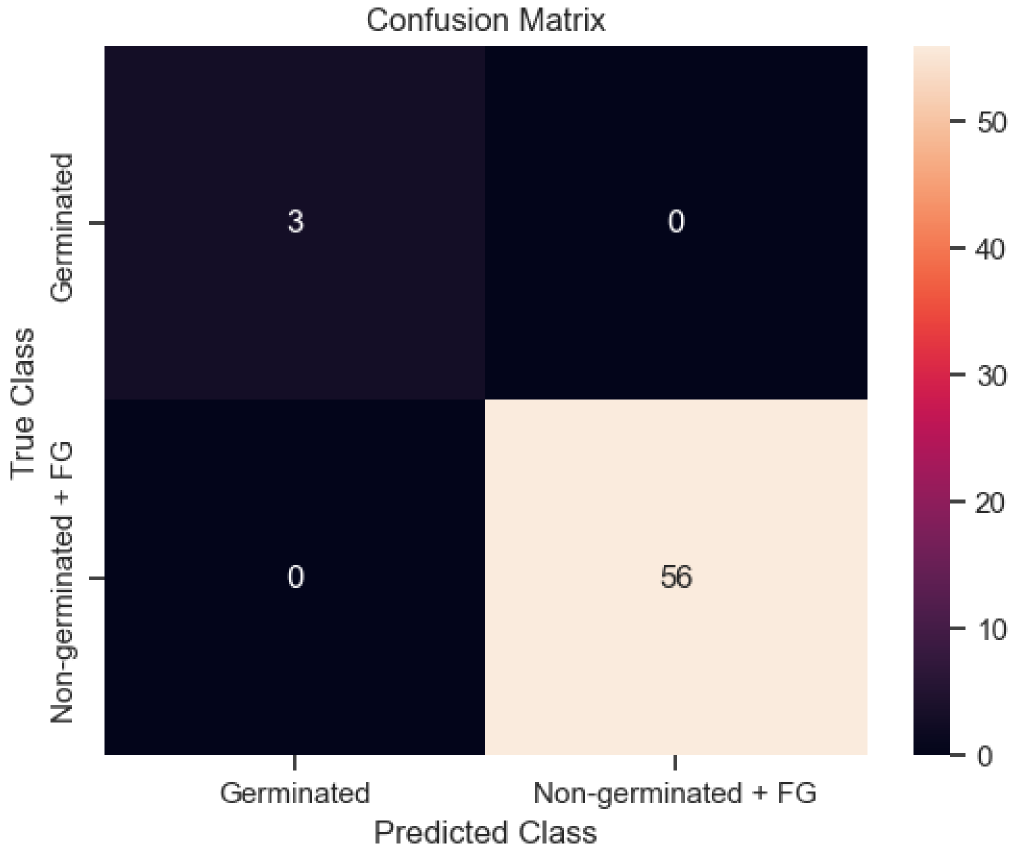

Figure S 27: Confusion matrix comparing the classification of G-type samples in the training set of partition 1 of the 3rd classifier with the predicted class (Germinated) trained on the two PCA dimensions. Confusions matrices of the training sets of partitions 2, 3 and 4 had the same outcome.

Table S 20: Logistic Regression classification results of G-type samples in the training set of partition 1 of the 3rd classifier with the predicted class (Germinated) trained on the two PCA dimensions. Classification results of the training sets of partitions 2, 3 and 4 had the same outcome.

	precision	recall	f1-score	support
Germinated	1.00	1.00	1.00	3
Non-germinated + FG	1.00	1.00	1.00	56
accuracy			1.00	59
macro avg	1.00	1.00	1.00	59
weighted avg	1.00	1.00	1.00	59

Test set:

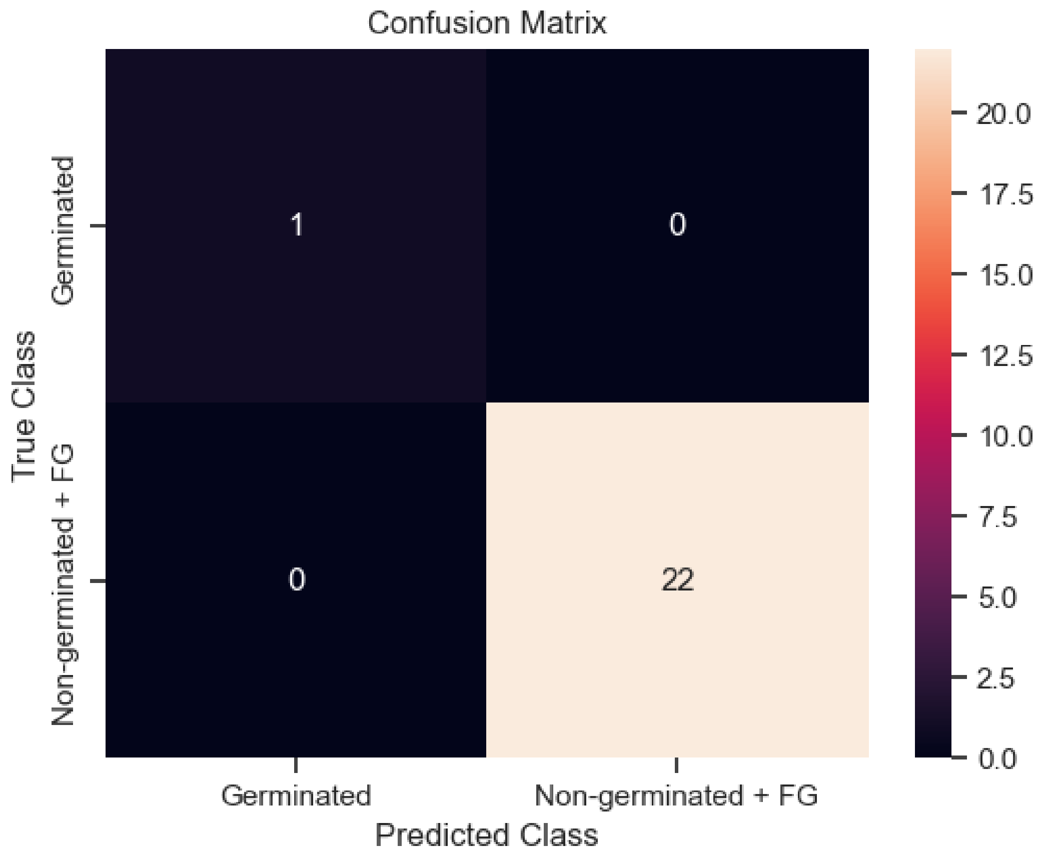

Figure S 28: Confusion matrix comparing the classification of G-type samples in the test set of partition 1 of the 3rd classifier with the predicted class (Germinated) trained on the two PCA dimensions. Confusion matrices of the test sets of partitions 2, 3 and 4 had the same outcome.

Table S 21: Logistic Regression classification results of G-type samples in the test set of partition 1 of the 3rd classifier predicting the target class (Germinated) using the two PCA dimensions. Classification results of the test sets of partitions 2, 3 and 4 had the same outcome.

	precision	recall	f1-score	support
Germinated	1.00	1.00	1.00	1
Non-germinated + FG	1.00	1.00	1.00	22
accuracy			1.00	23
macro avg	1.00	1.00	1.00	23
weighted avg	1.00	1.00	1.00	23

Appendix: Raw data of measurements

Chromatograms of marker quantification: Calibration solution

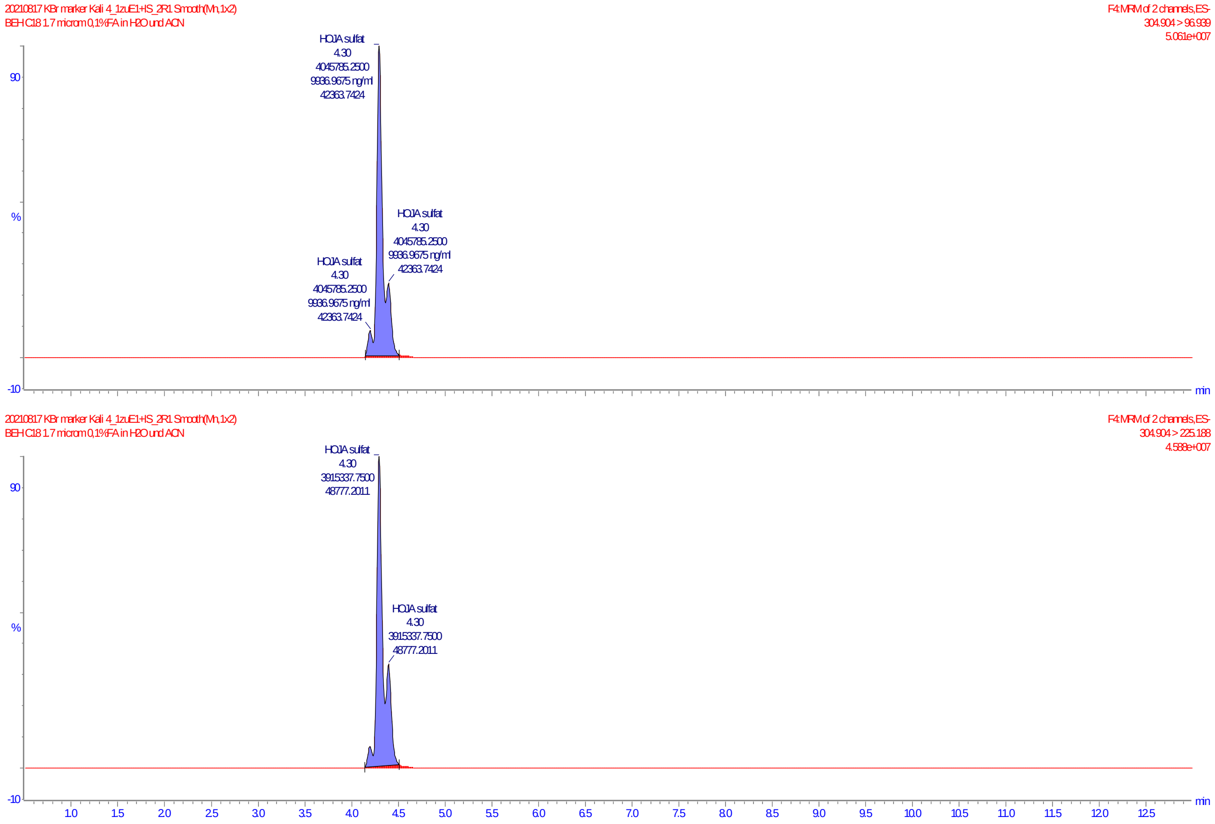

Figure S29: MRM chromatograms of quantifier (top) and qualifier (bottom) of HOJA sulfate standard in calibration solution 1:10 (v/v).

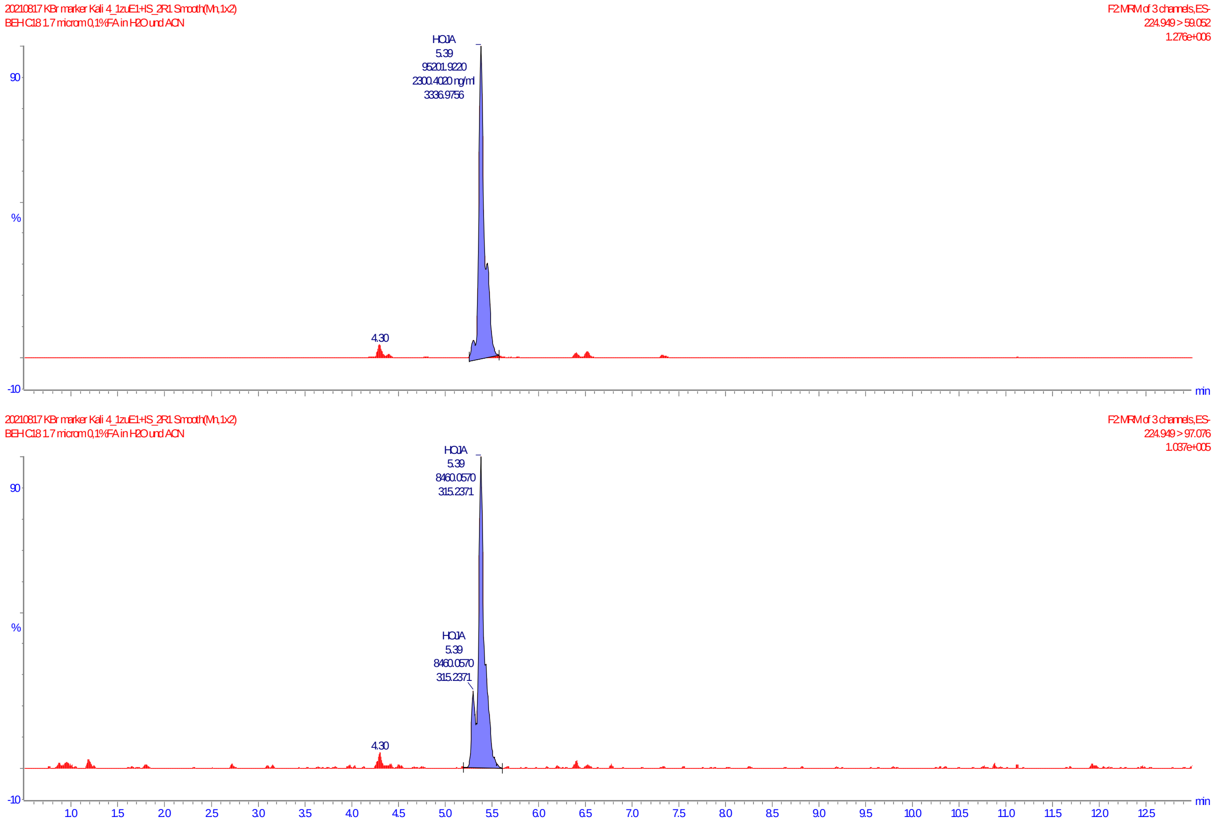

Figure S30: MRM chromatograms of quantifier (top) and qualifier (bottom) of HOJA standard in calibration solution 1:10 (v/v).

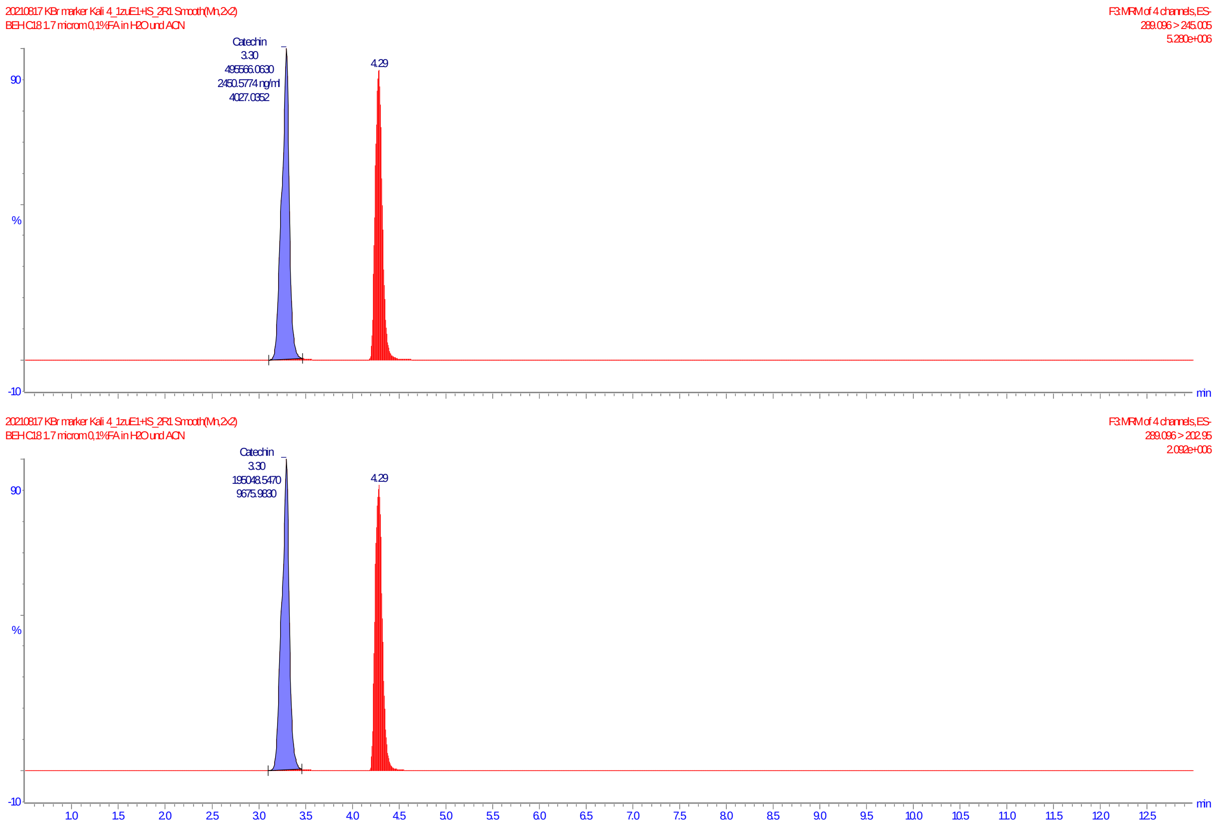

Figure S31: MRM chromatograms of quantifier (top) and qualifier (bottom) of (+)-catechine standard in calibration solution 1:10 (v/v).

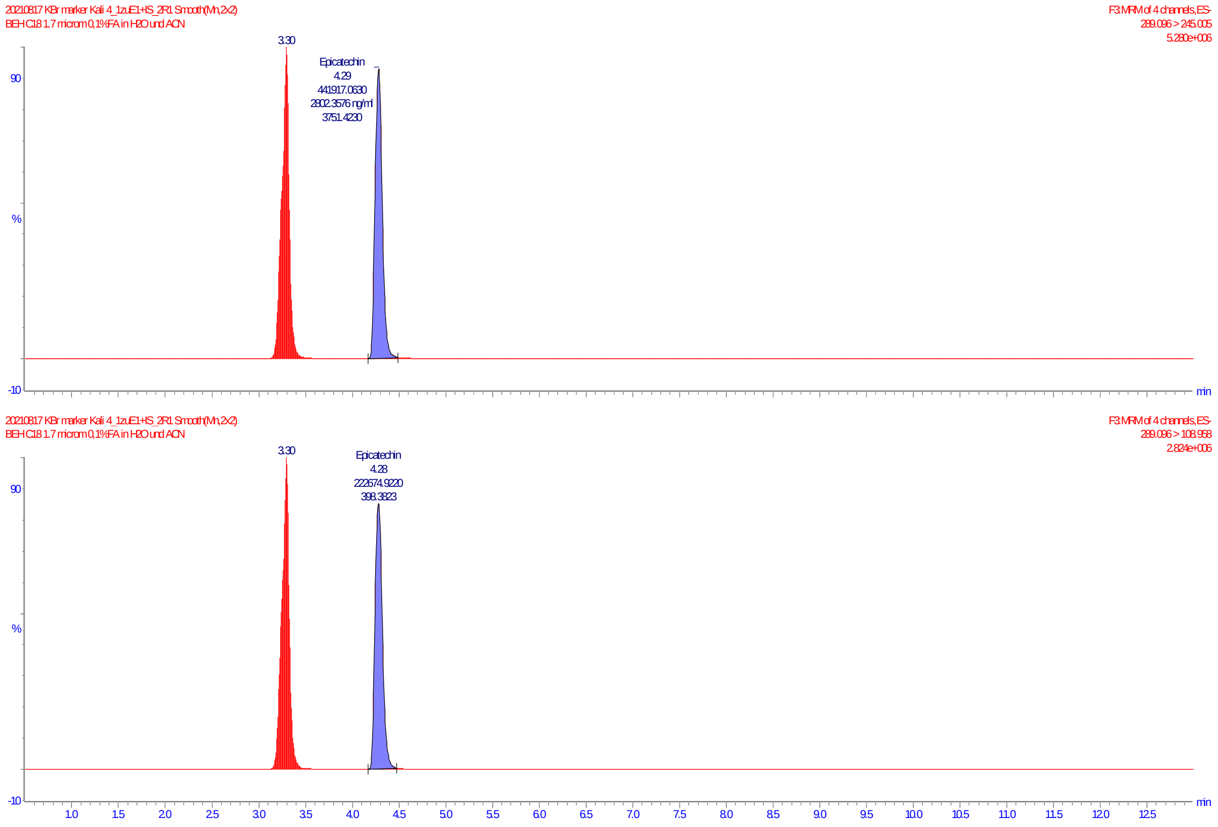

Figure S32: MRM chromatograms of quantifier (top) and qualifier (bottom) of (-)-epicatechine in calibration solution 1:10 (v/v).

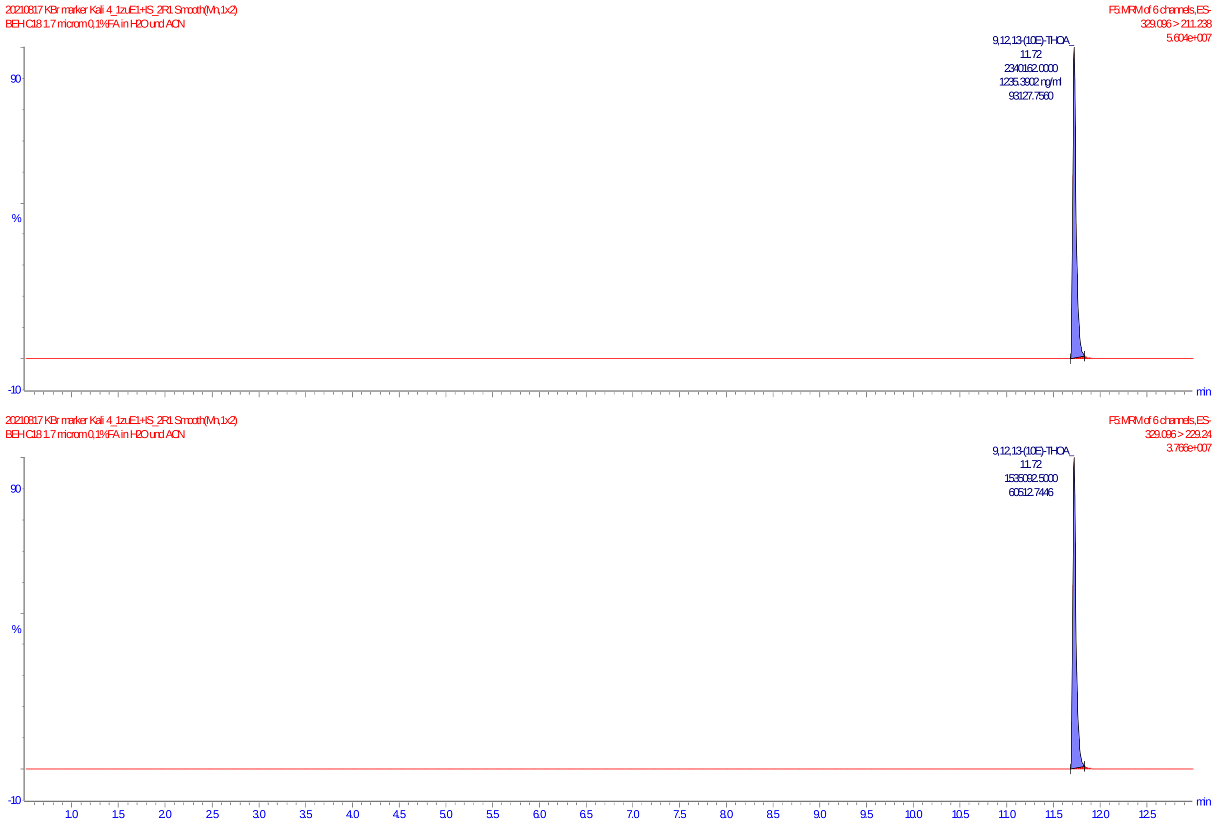

Figure S33: MRM chromatograms of quantifier (top) and qualifier (bottom) of 9,12,13-(10E)-THOA standard in calibration solution 1:10 (v/v).

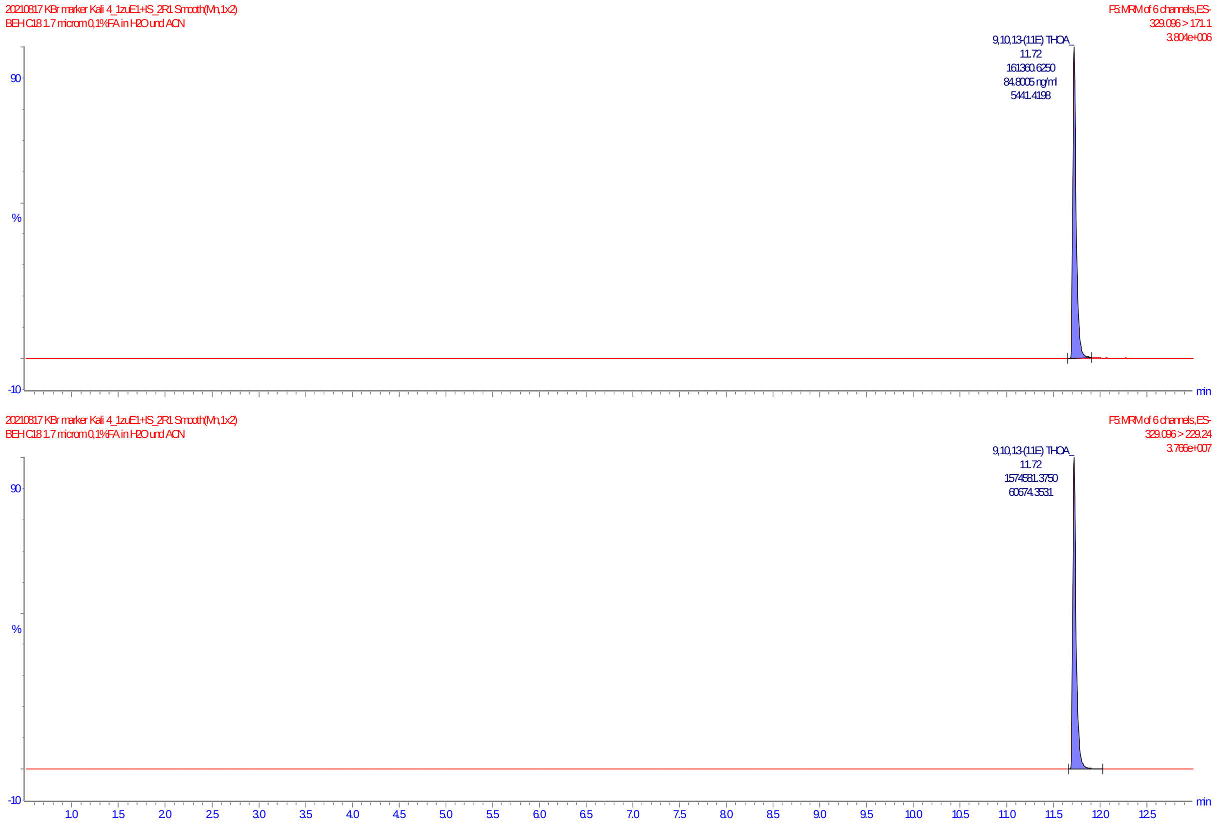

Figure S34: MRM chromatograms of quantifier (top) and qualifier (bottom) of 9,10,13-(11E)-THOA measured with the 9,12,13-(10E)-THOA standard in calibration solution 1:10 (v/v).

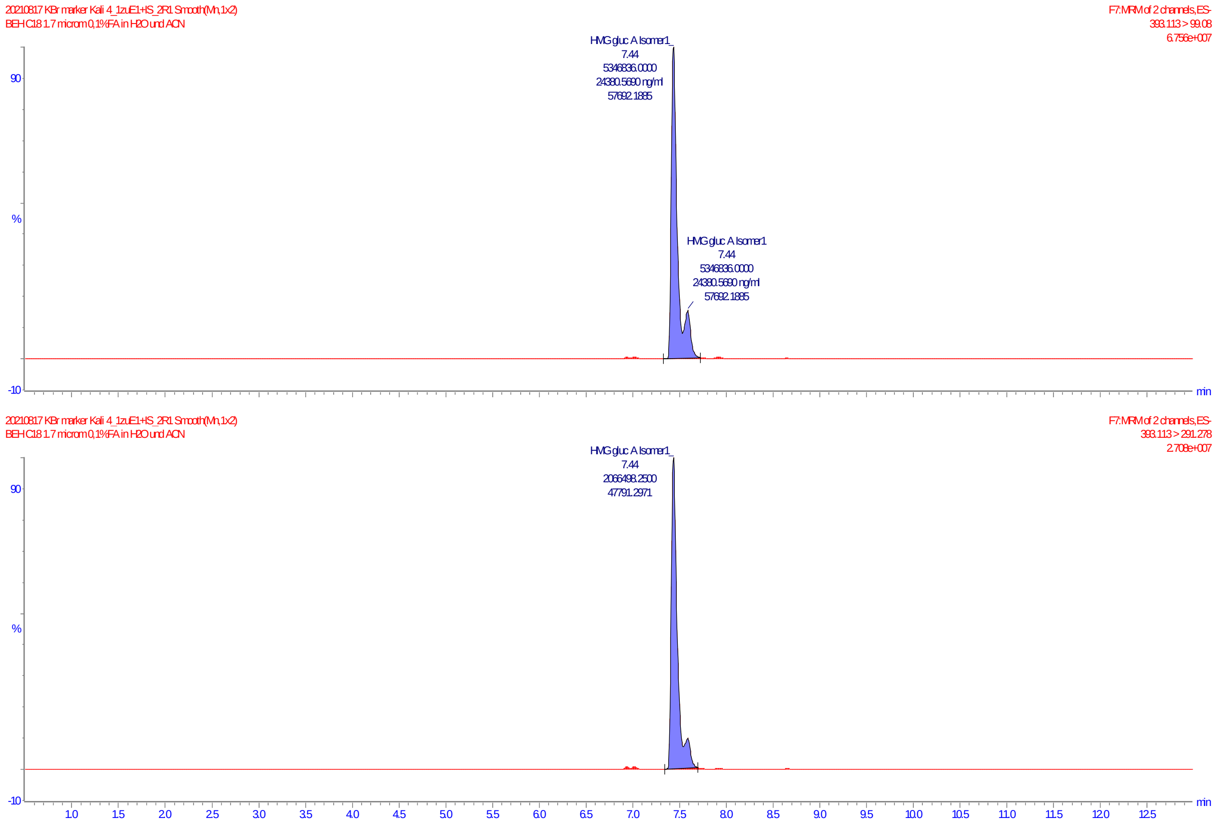

Figure S35: MRM chromatograms of quantifier (top) and qualifier (bottom) of HMG gluc A isomer 1 in calibration solution 1:10 (v/v).

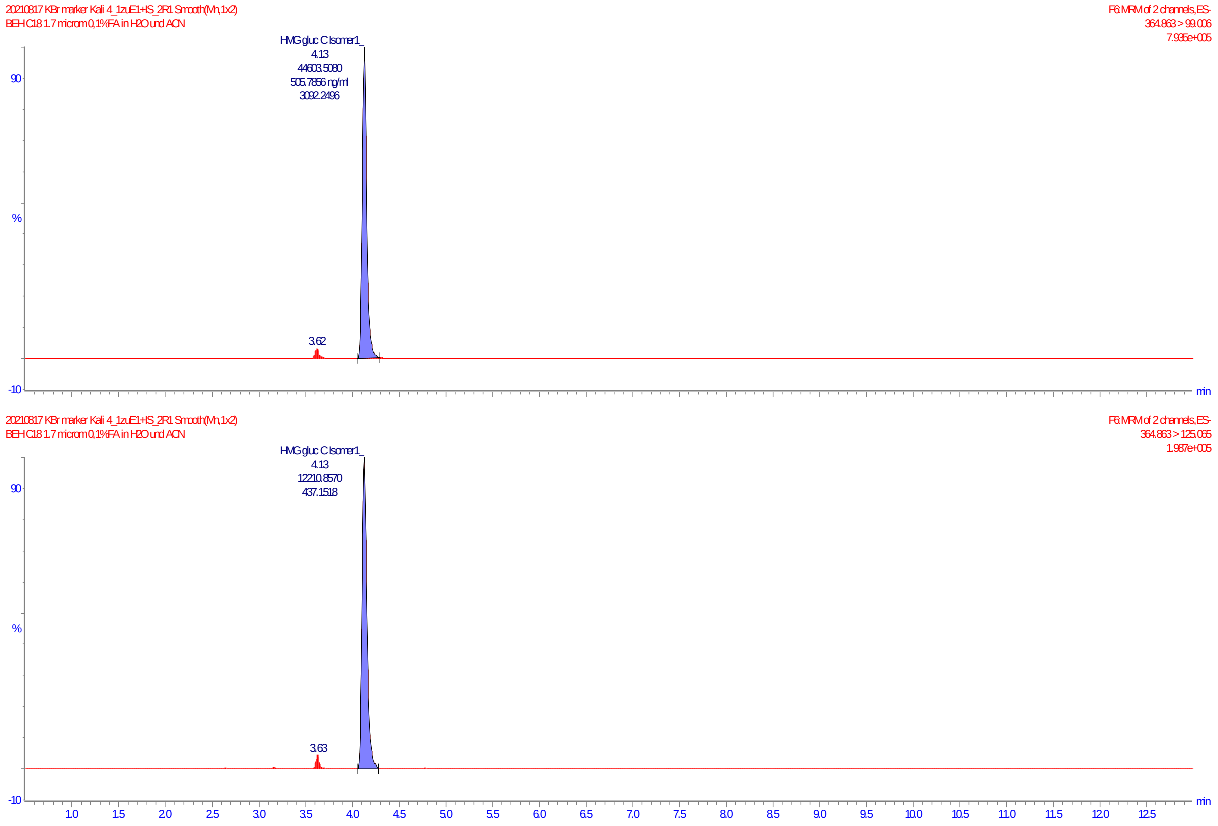

Figure S36: MRM chromatograms of quantifier (top) and qualifier (bottom) of HMG gluc C isomer 1 in calibration solution 1:10 (v/v).

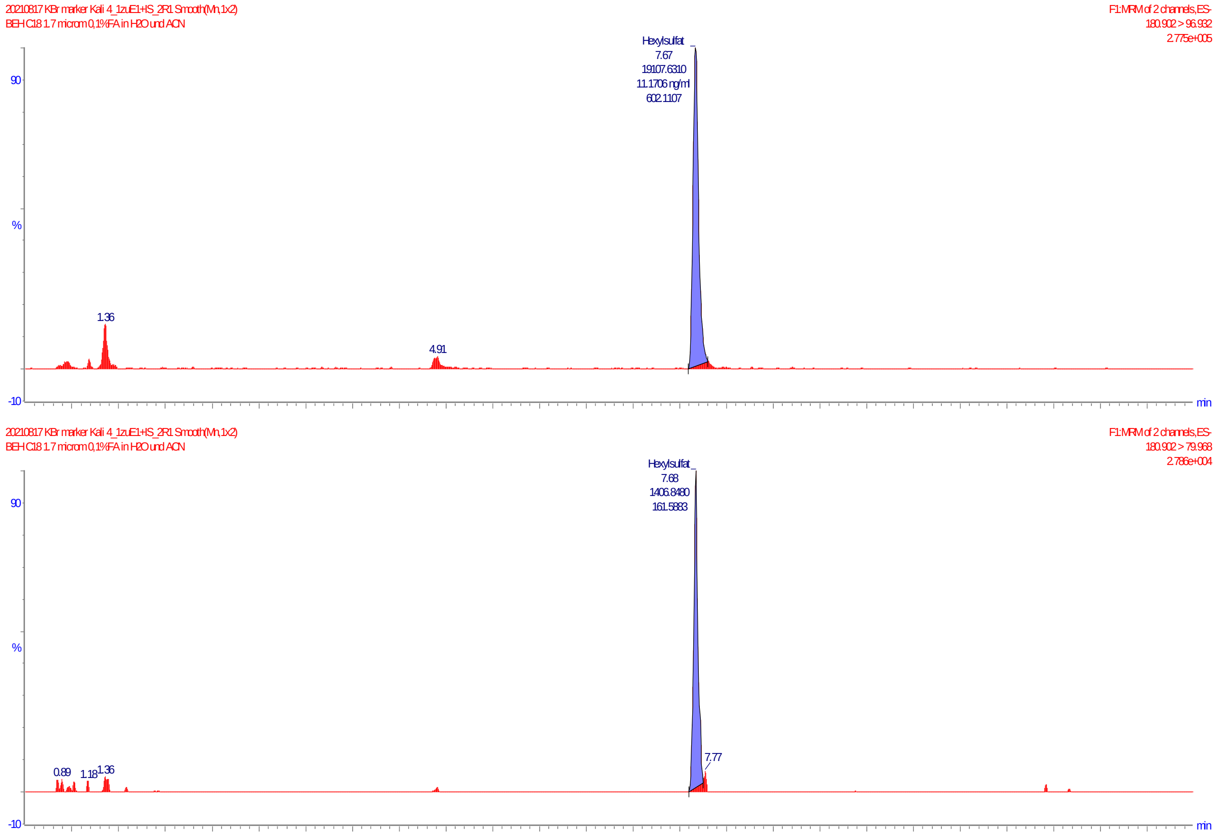

Figure S37: MRM chromatograms of quantifier (top) and qualifier (bottom) of hexyl sulfate (IS) in calibration solution 1:10 (v/v).

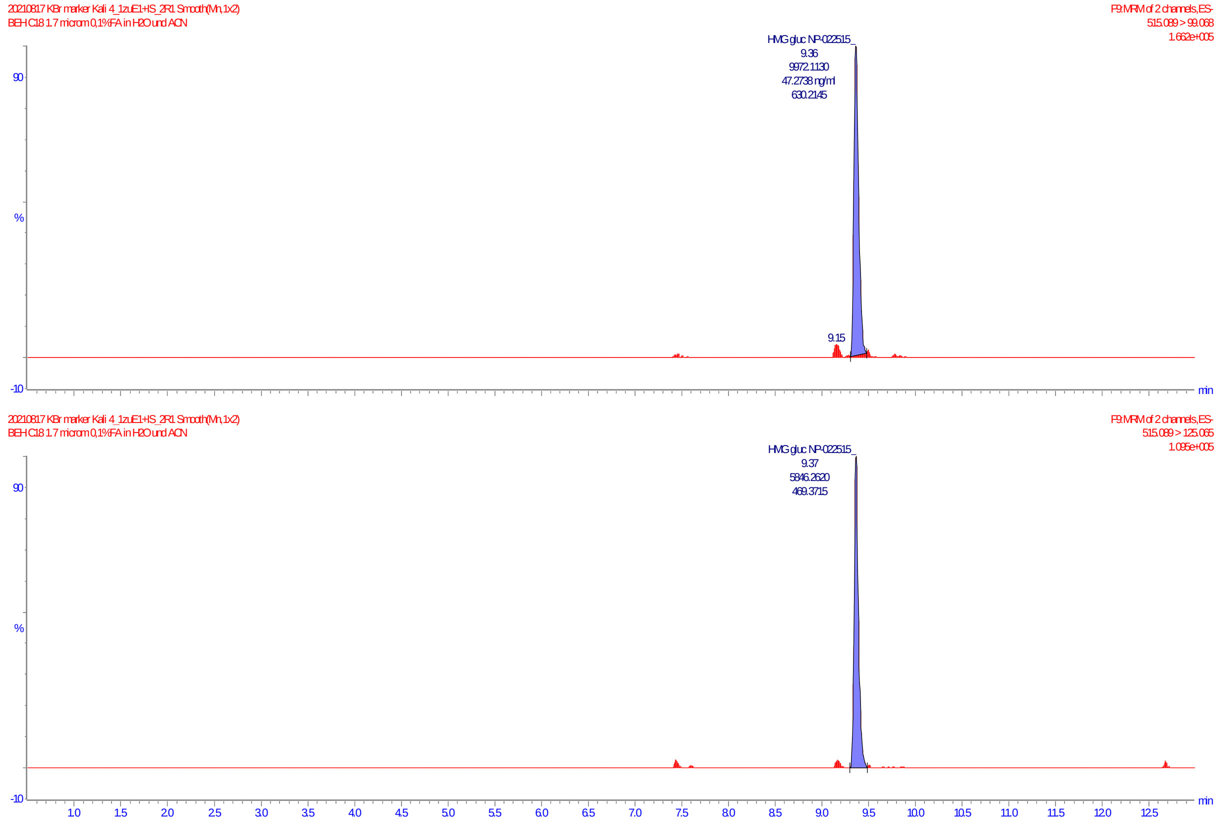

Figure S38: MRM chromatograms of quantifier (top) and qualifier (bottom) of HMG gluc I (IS) in calibration solution 1:10 (v/v).

Chromatograms of marker quantification: Sample solution

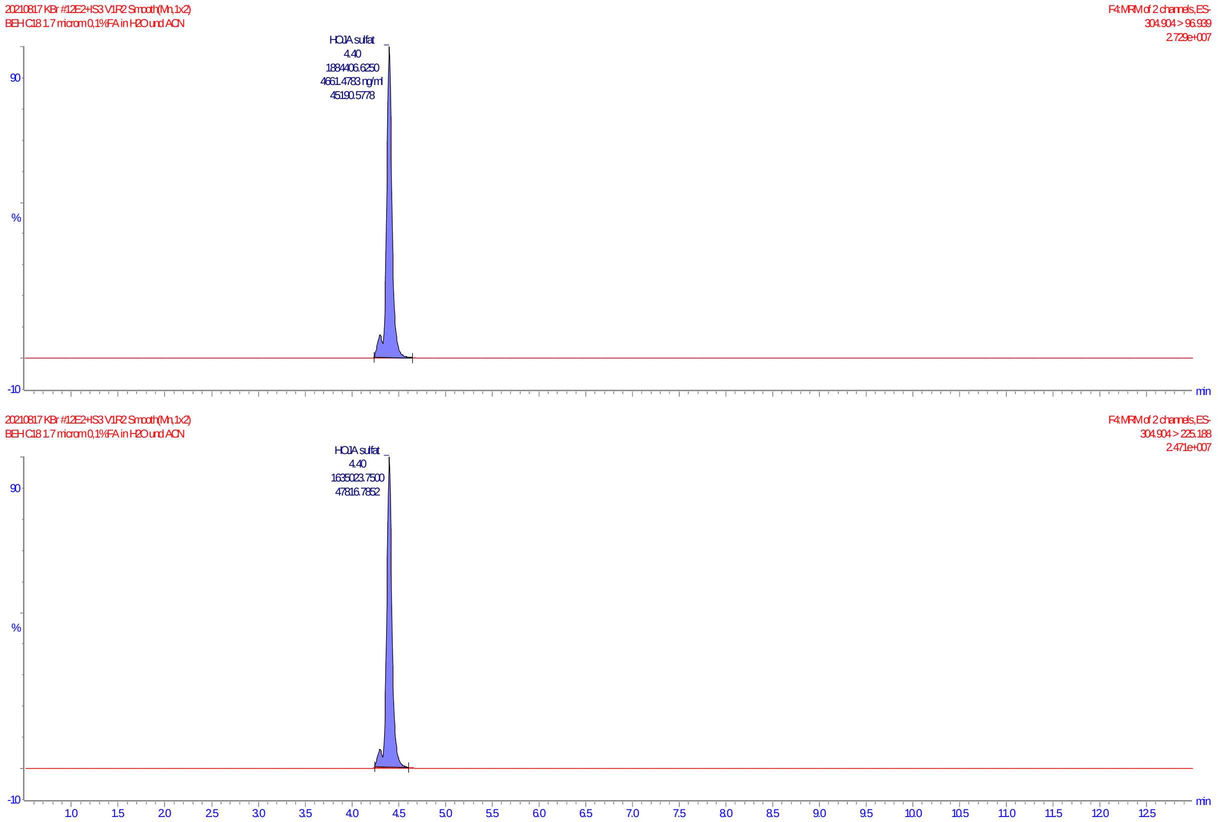

Figure S39: MRM chromatograms of quantifier (top) and qualifier (bottom) of HOJA sulfate in extract of sample #12, 1:10 (v/v).

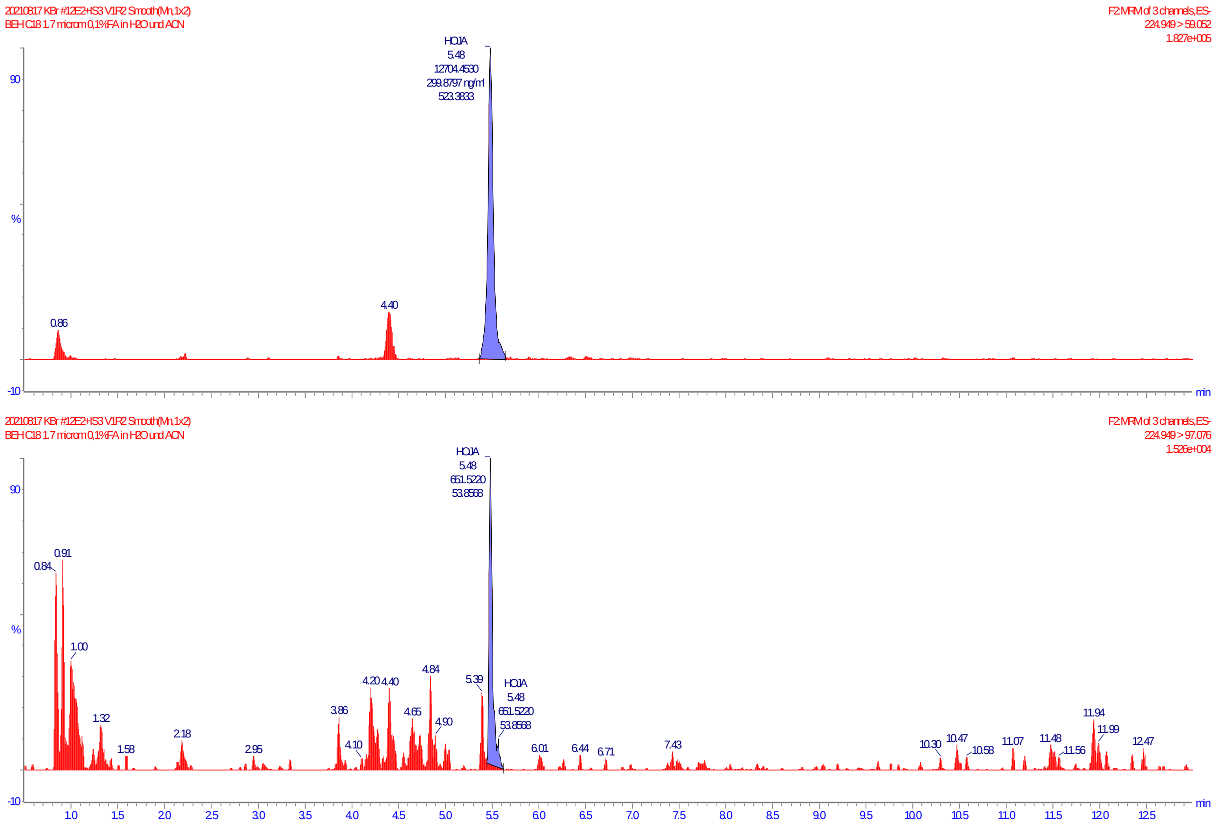

Figure S40: MRM chromatograms of quantifier (top) and qualifier (bottom) of HOJA in extract of sample #12, 1:10 (v/v).

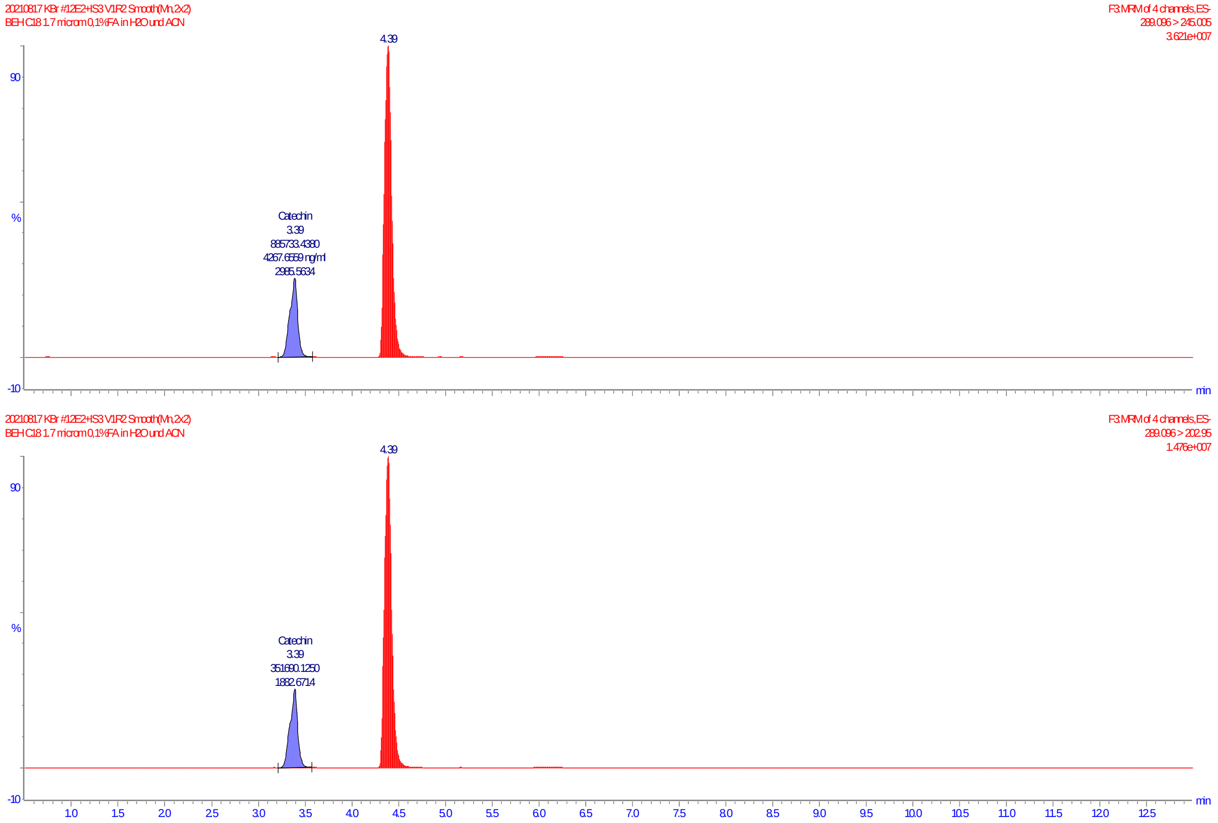

Figure S41: MRM chromatograms of quantifier (top) and qualifier (bottom) of (+)-catechine in extract of sample #12, 1:10 (v/v).

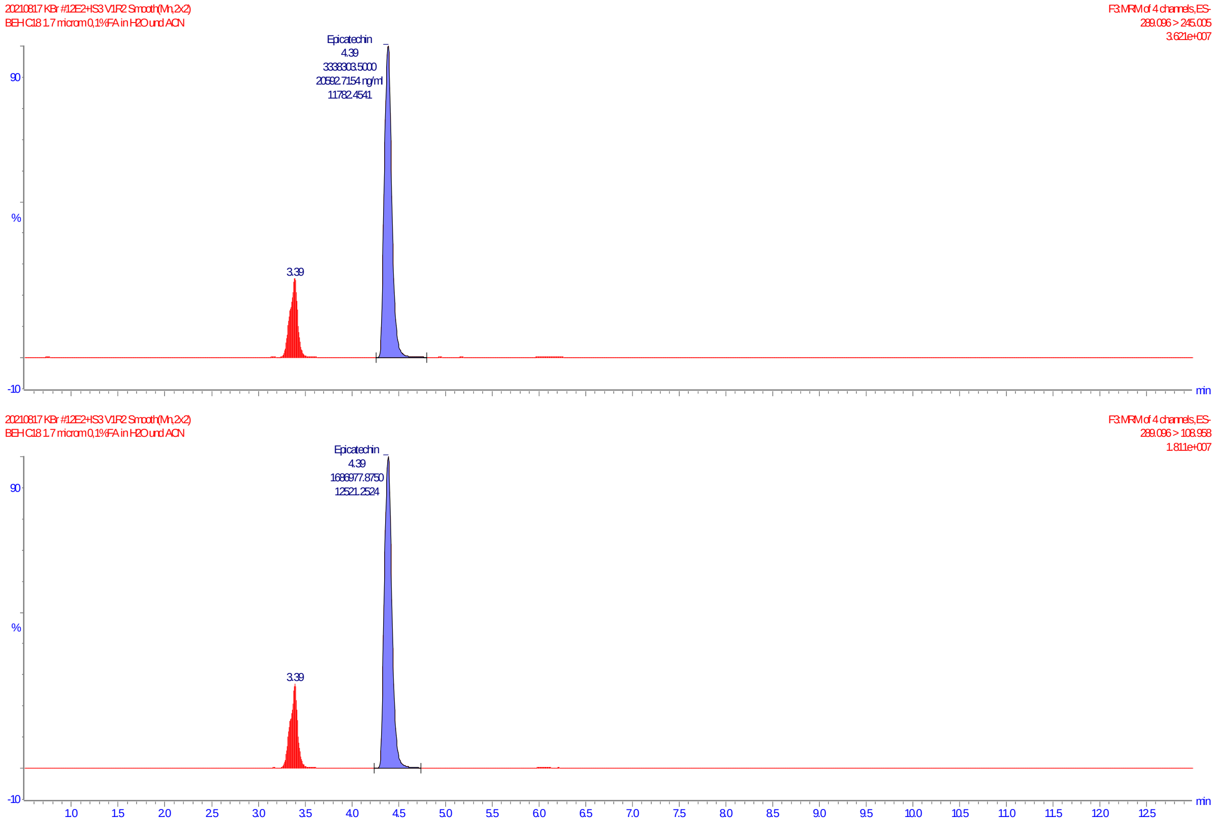

Figure S42: MRM chromatograms of quantifier (top) and qualifier (bottom) of (-)-epicatechine in extract of sample #12, 1:10 (v/v).

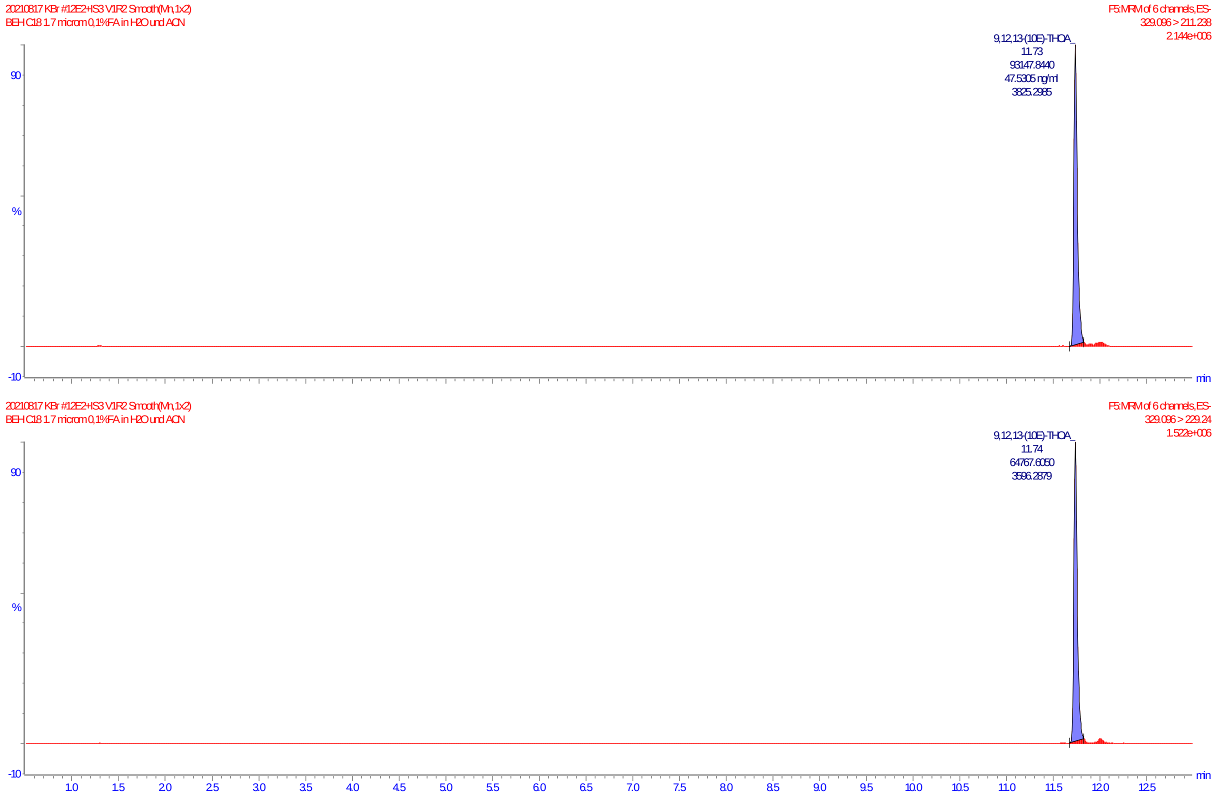

Figure S43: MRM chromatograms of quantifier (top) and qualifier (bottom) of 9,12,13-(10E)-THOA in extract of sample #12, 1:10 (v/v).

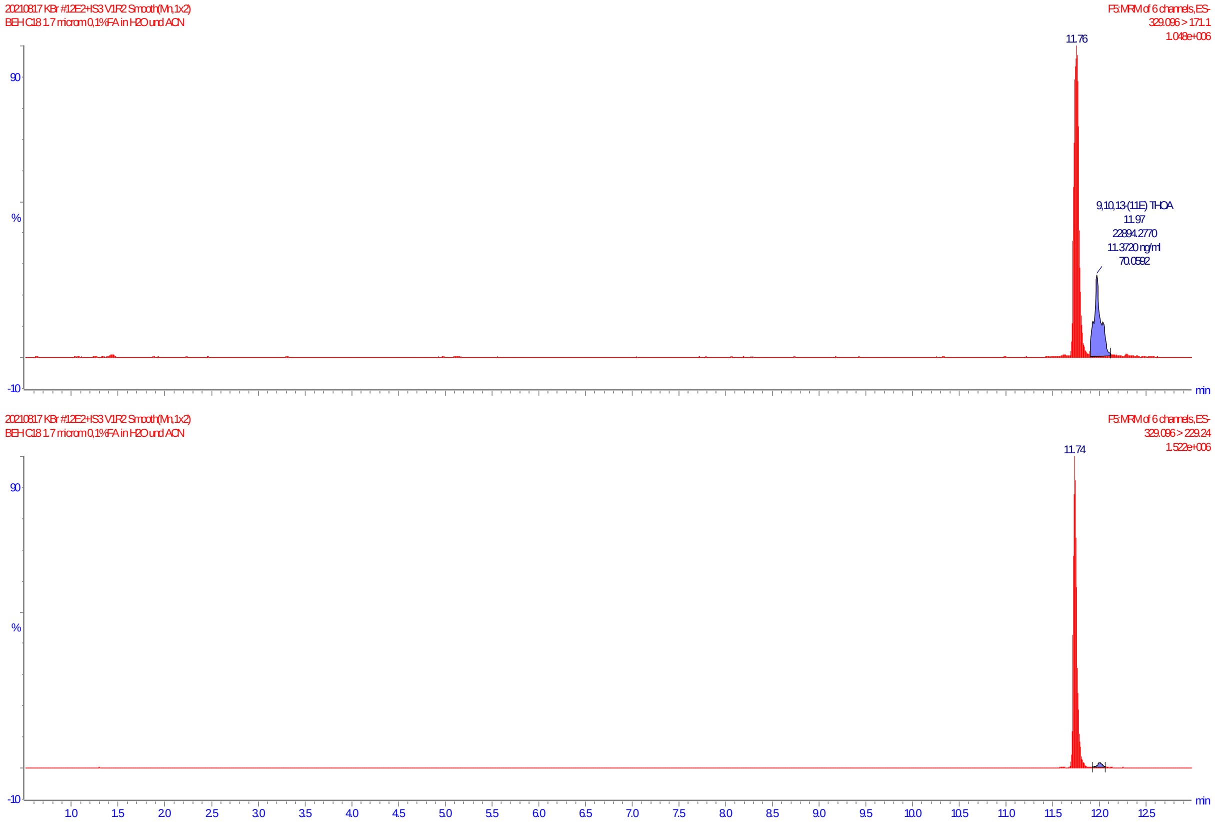

Figure S44: MRM chromatograms of quantifier (top) and qualifier (bottom) of 9,10,13-(11E)-THOA in extract of sample #12, 1:10 (v/v).

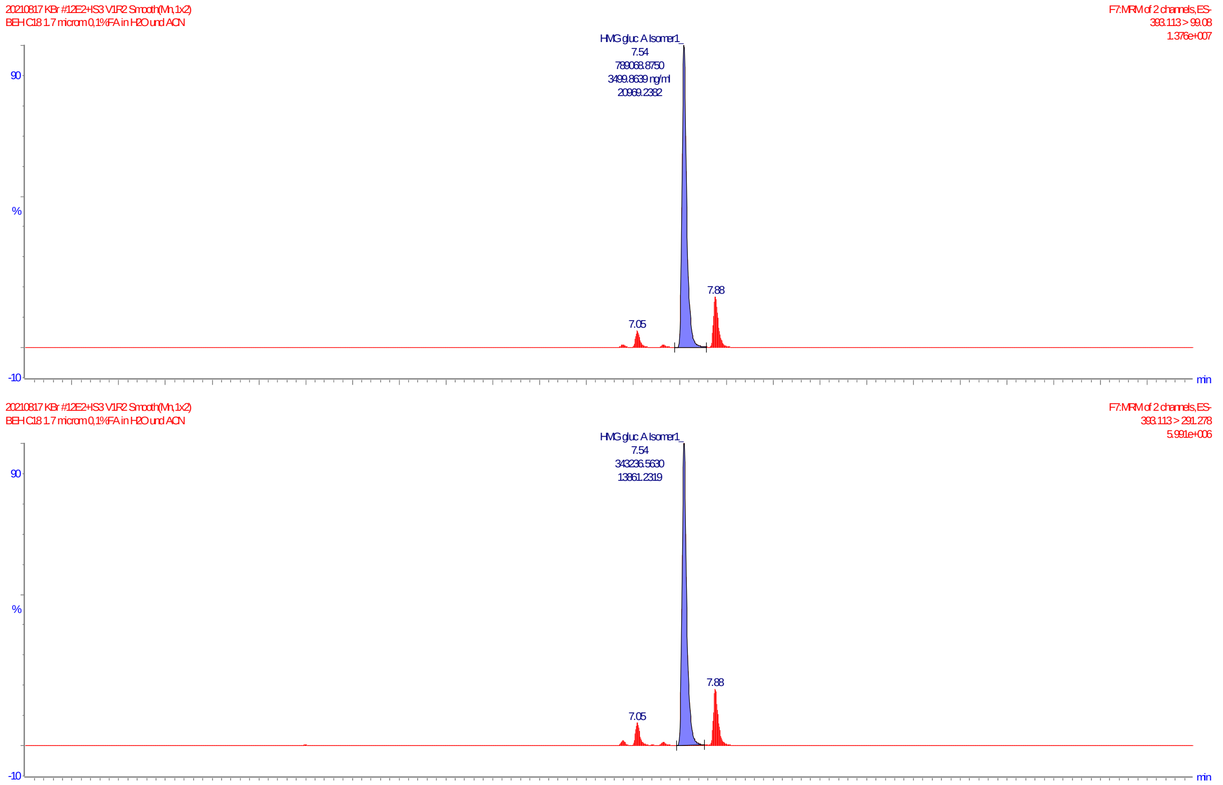

Figure S45: MRM chromatograms of quantifier (top) and qualifier (bottom) of HMG gluc A isomer 1 in extract of sample #12, 1:10 (v/v).

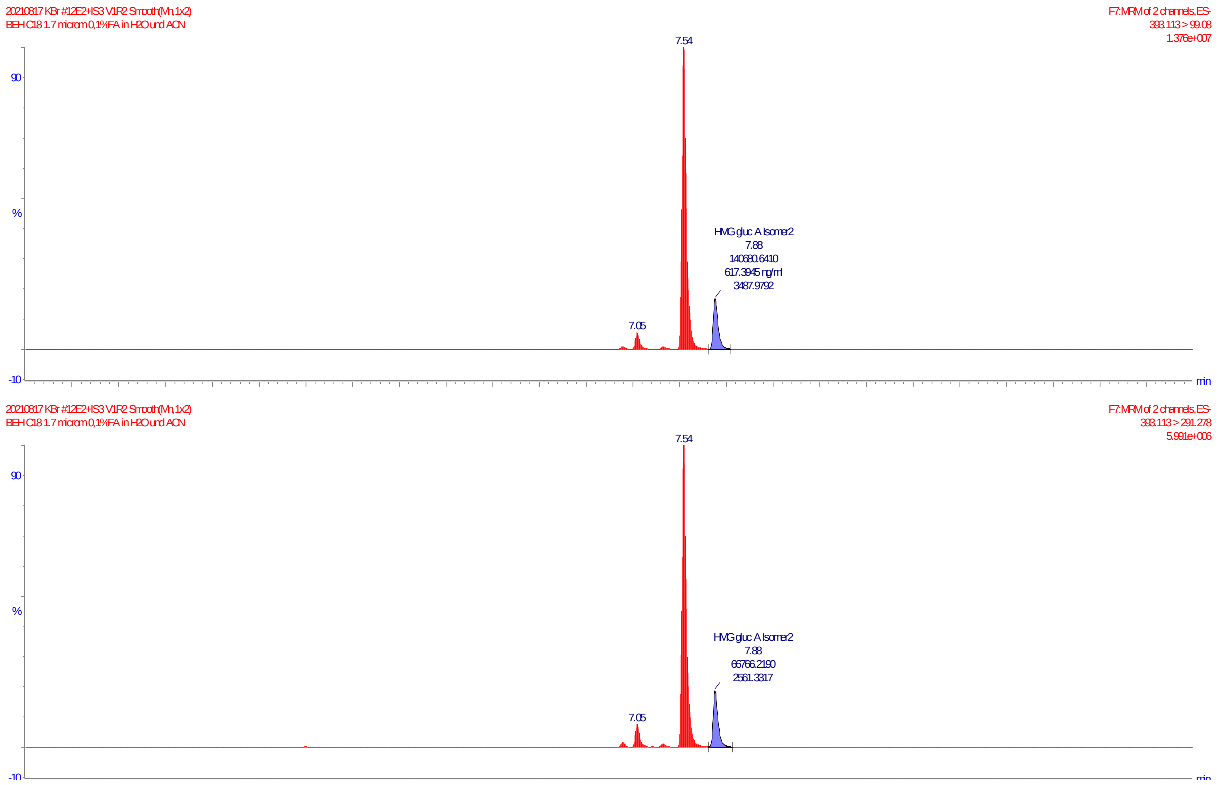

Figure S46: MRM chromatograms of quantifier (top) and qualifier (bottom) of HMG gluc A isomer 2 in extract of sample #12, 1:10 (v/v).

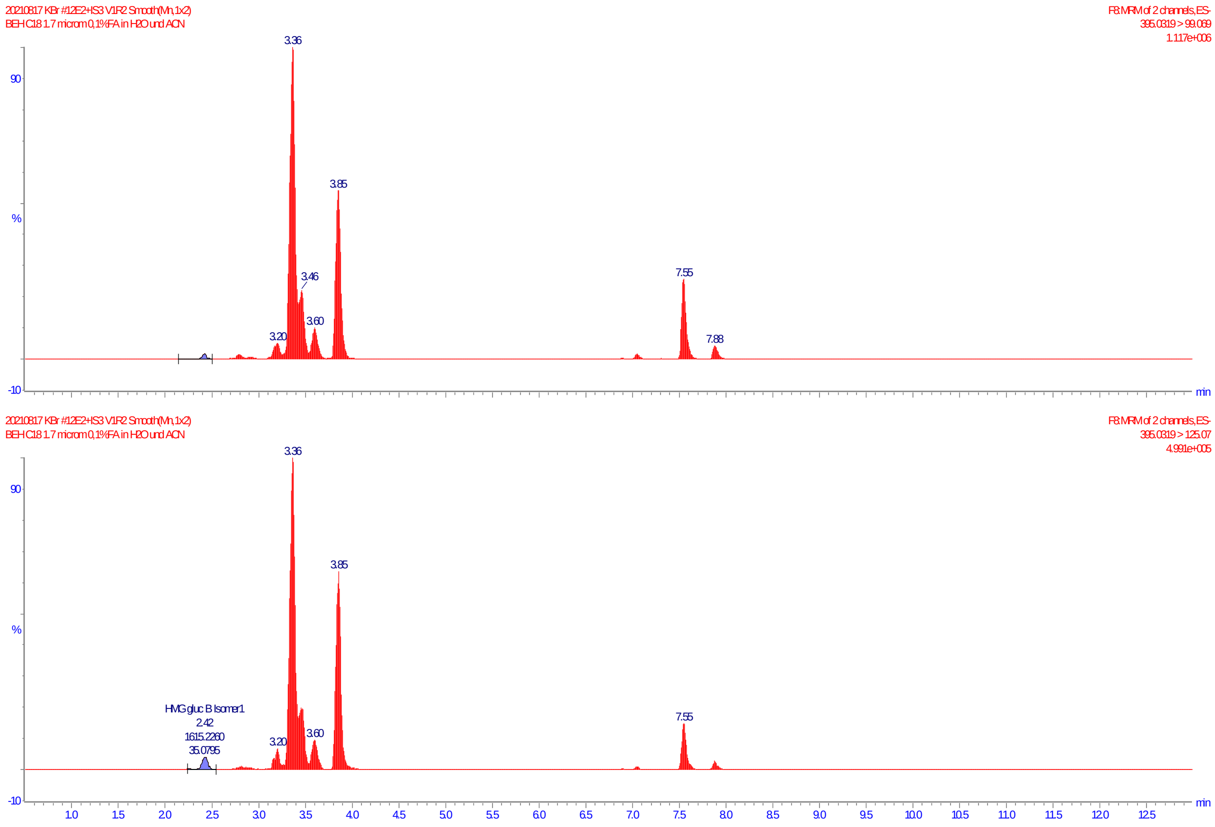

Figure S47: MRM chromatograms of quantifier (top) and qualifier (bottom) of HMG gluc B isomer 1 in extract of sample #12, 1:10 (v/v).

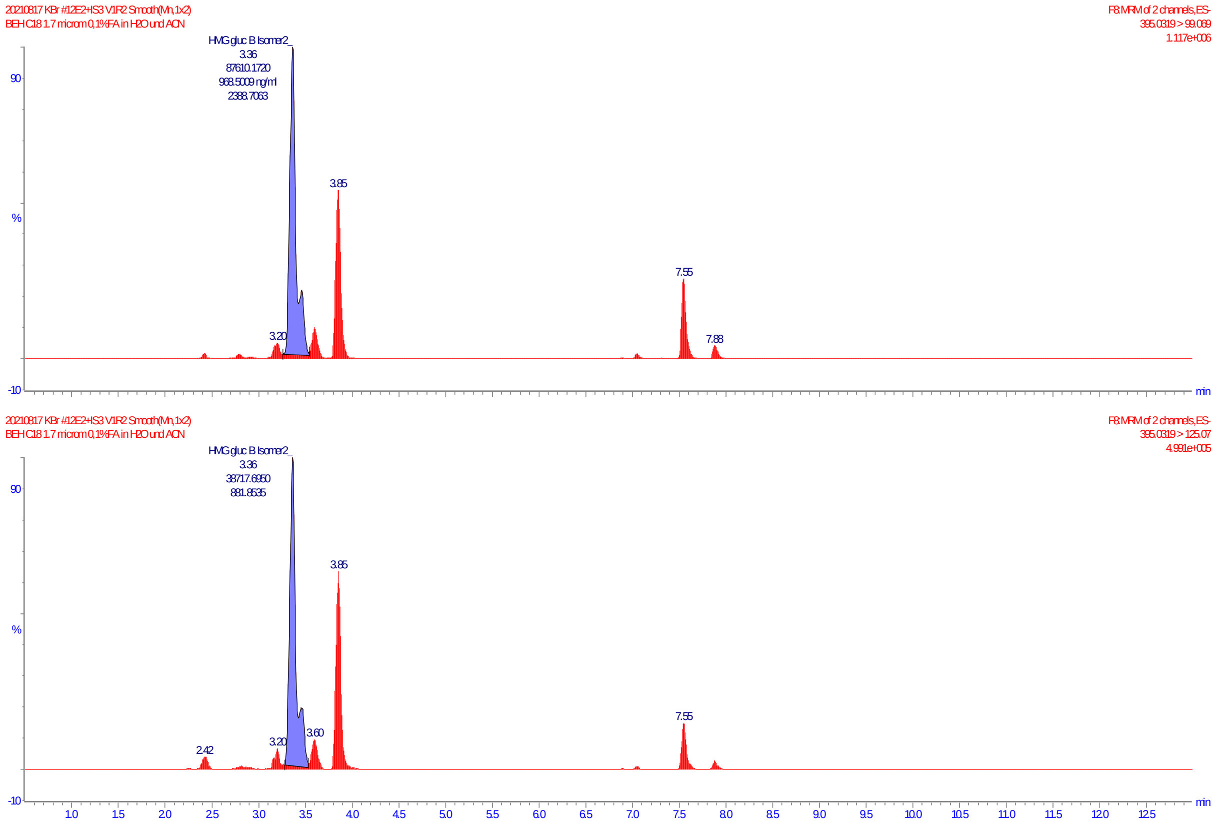

Figure S48: MRM chromatograms of quantifier (top) and qualifier (bottom) of HMG gluc B isomer 2 in extract of sample #12, 1:10 (v/v).

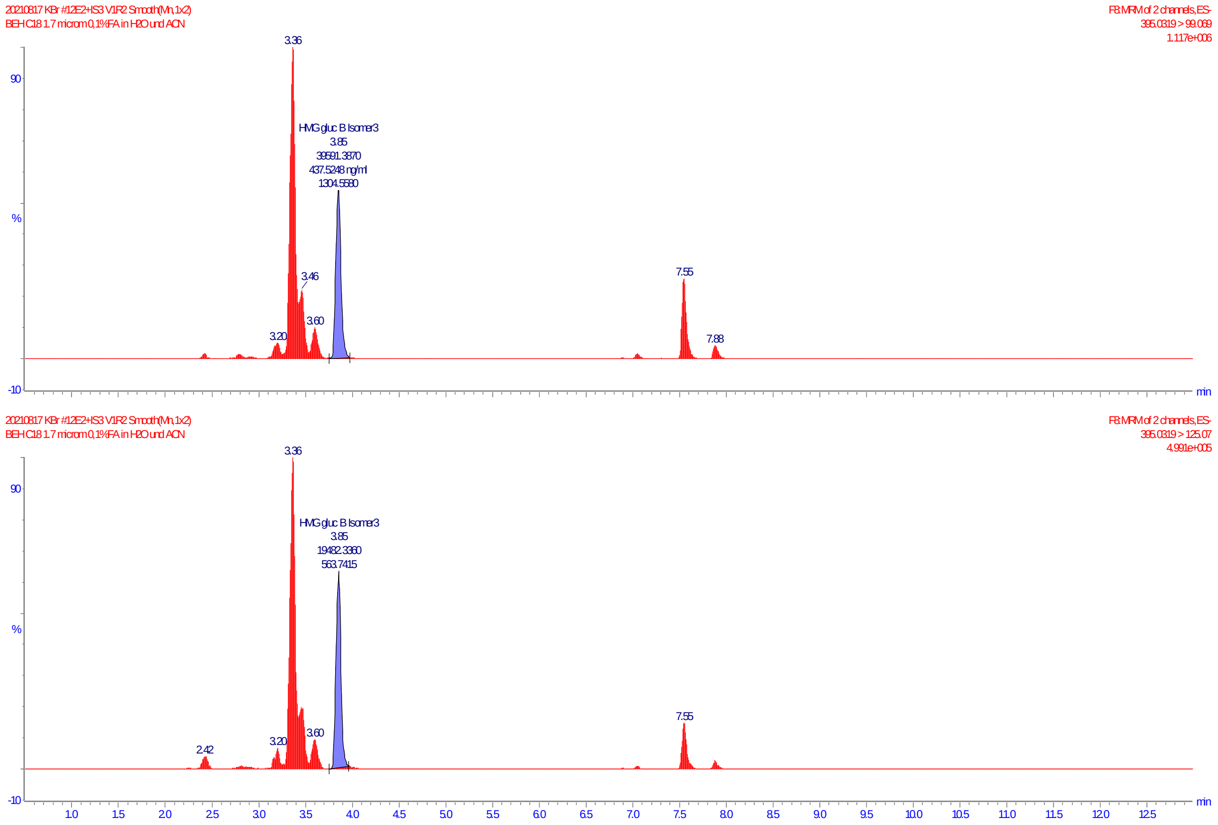

Figure S49: MRM chromatograms of quantifier (top) and qualifier (bottom) of HMG gluc B isomer 3 in extract of sample #12, 1:10 (v/v).

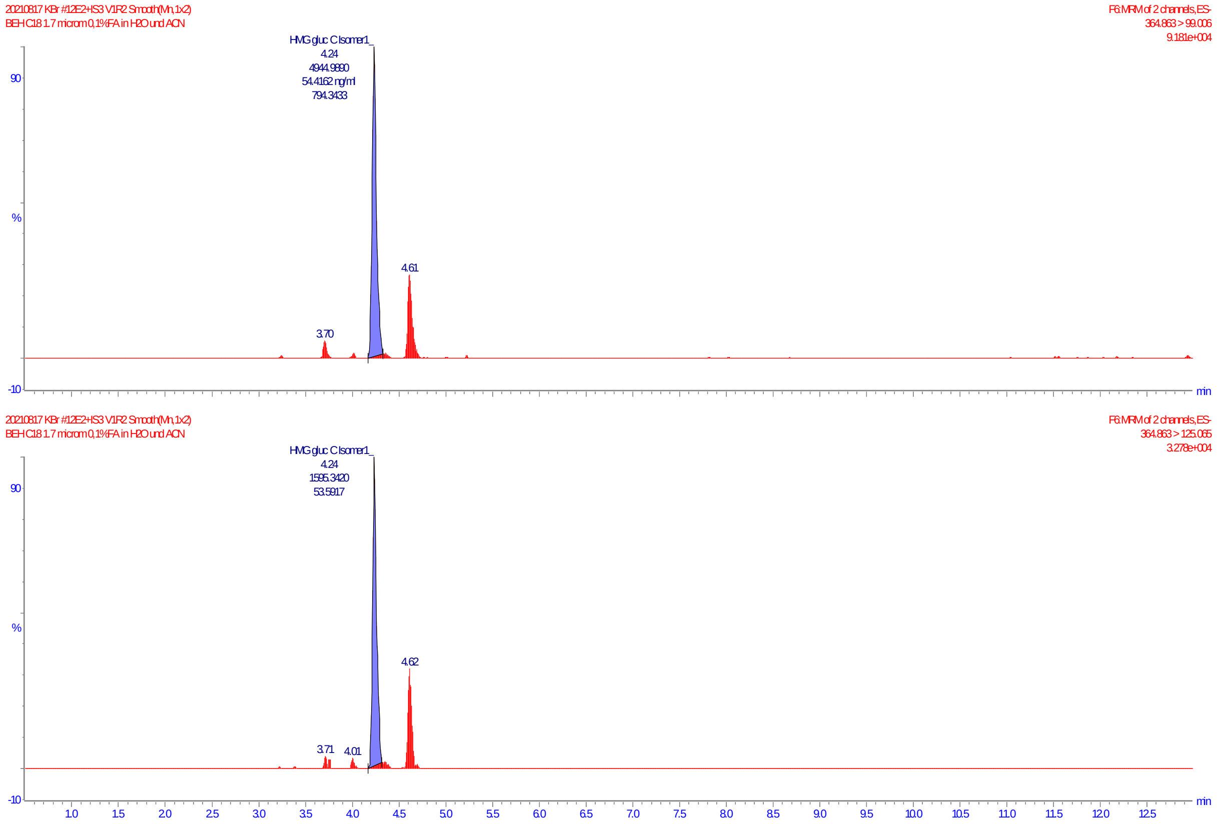

Figure S50: MRM chromatograms of quantifier (top) and qualifier (bottom) of HMG gluc C isomer 1 in extract of sample #12, 1:10 (v/v).

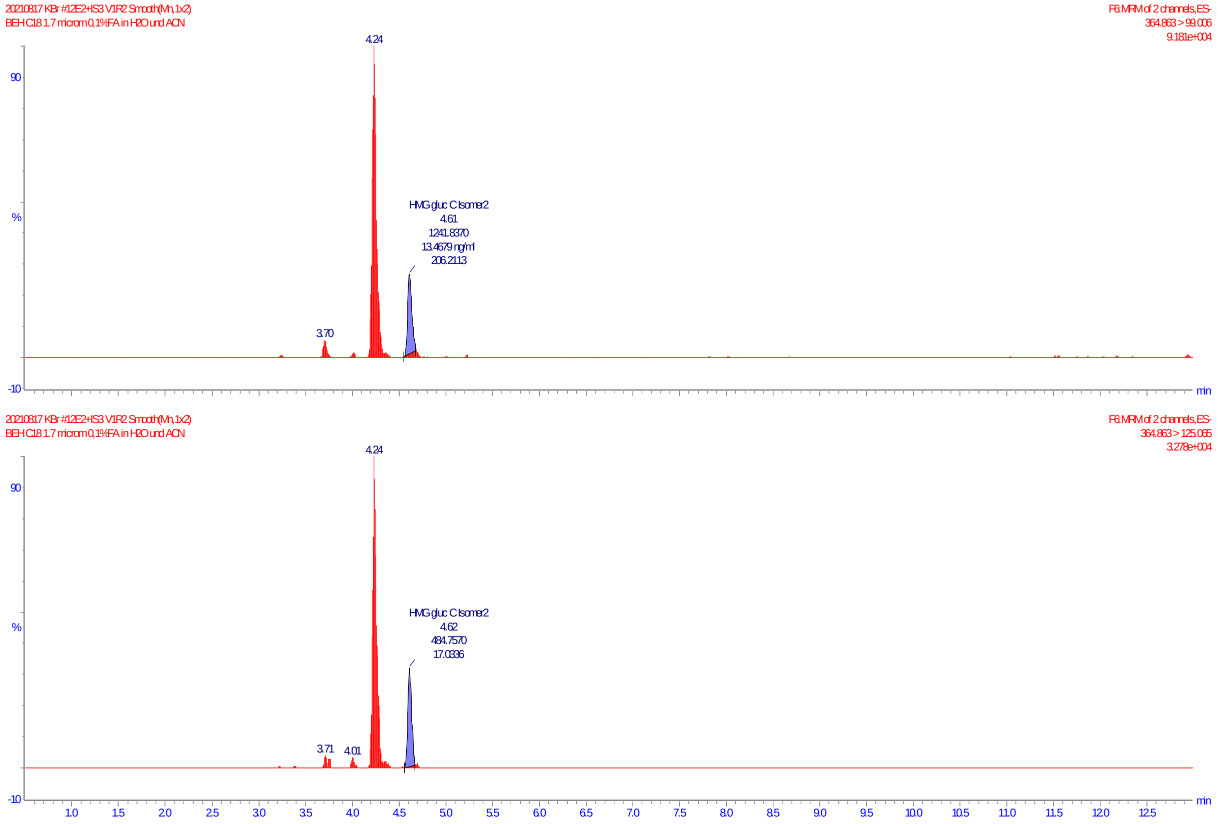

Figure S51: MRM chromatograms of quantifier (top) and qualifier (bottom) of HMG gluc C isomer 2 in extract of sample #12, 1:10 (v/v).

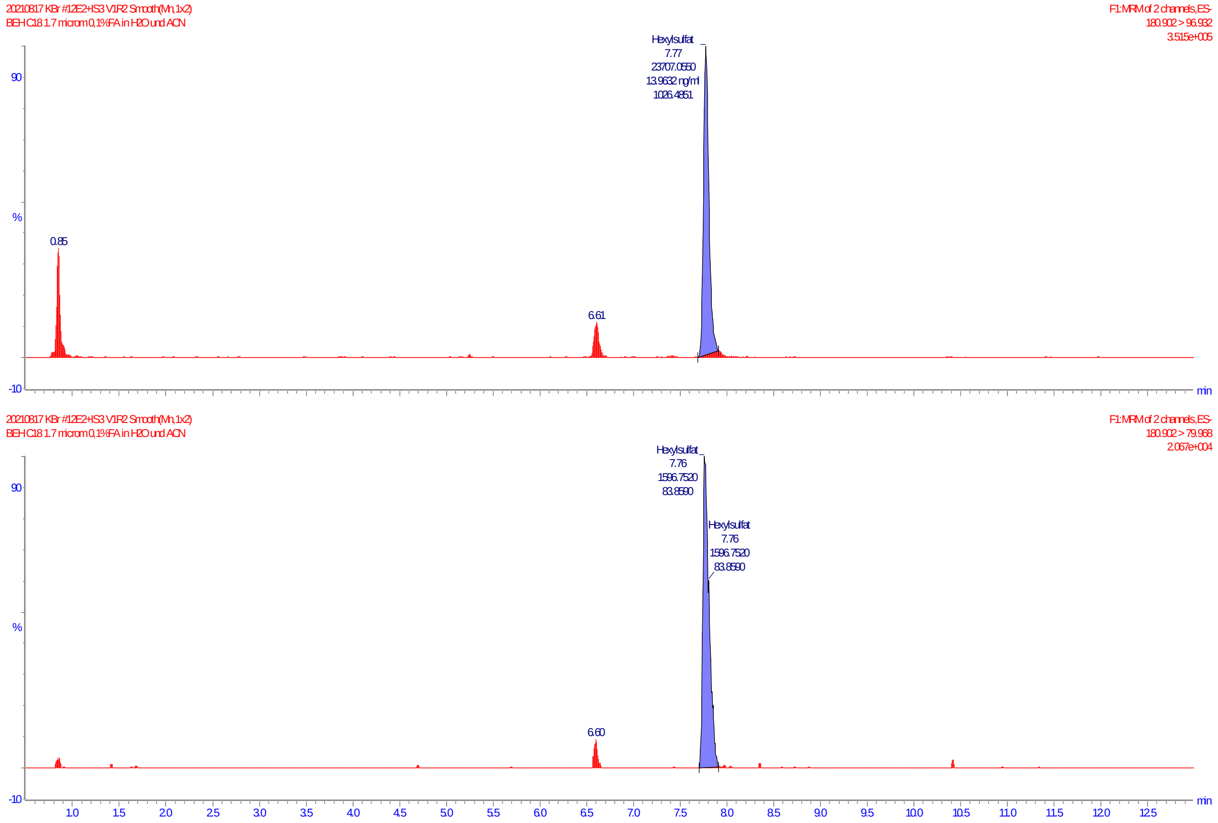

Figure S52: MRM chromatograms of quantifier (top) and qualifier (bottom) of hexyl sulfate (IS) in extract of sample #12, 1:10 (v/v).

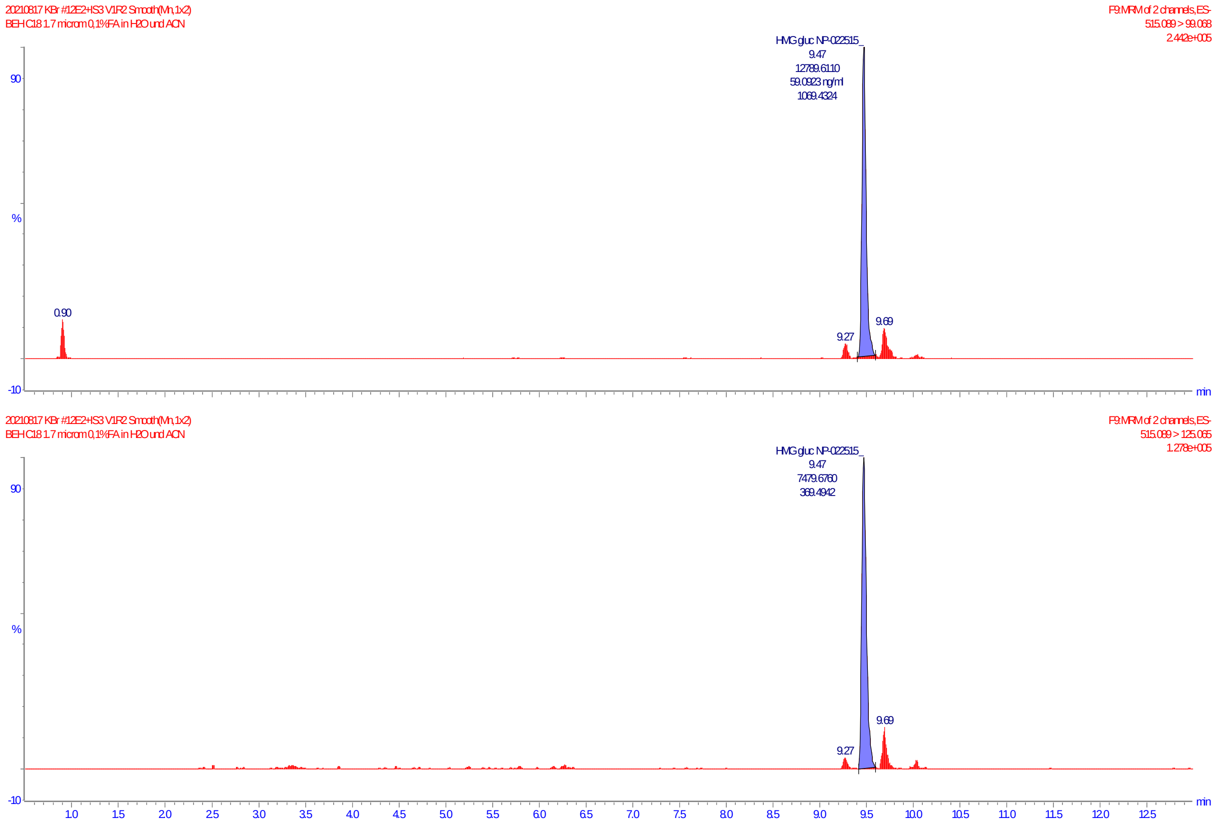

Figure S53: MRM chromatograms of quantifier (top) and qualifier (bottom) of HMG gluc I (IS) in extract of sample #12, 1:10 (v/v).

Chromatograms of quantification of further HMG glucosides: Calibration solution

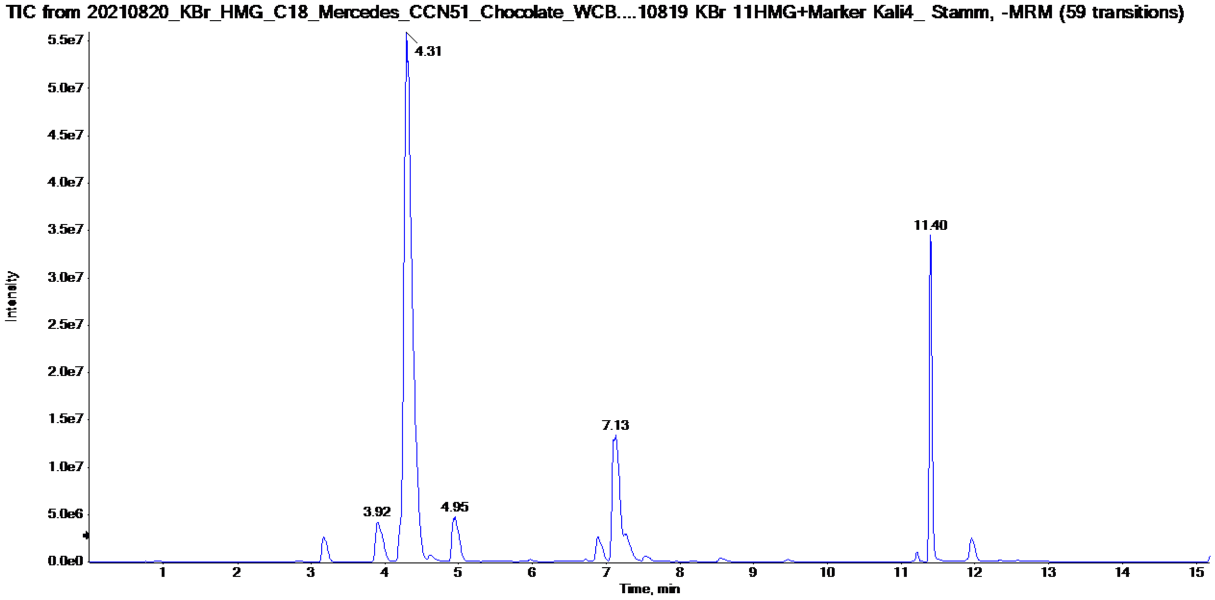

Figure S54:TIC of all MRMs

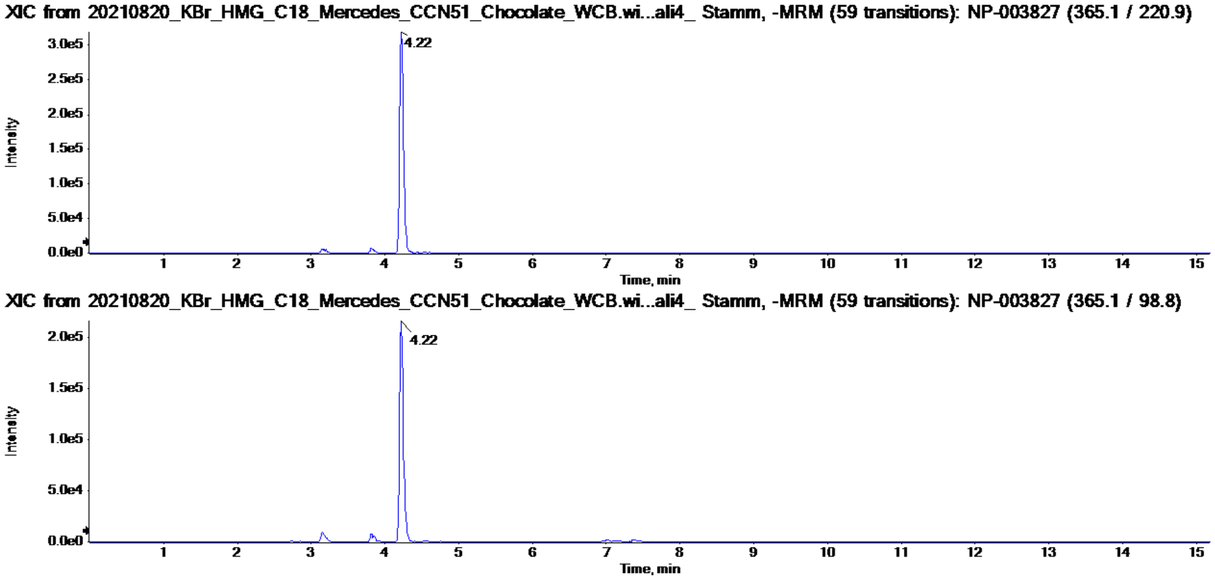

Figure S55: HMG gluc C isomer 1 in calibration stock solution, XIC with traces of quantifier (upper) and qualifier (bottom) mrm transitions.

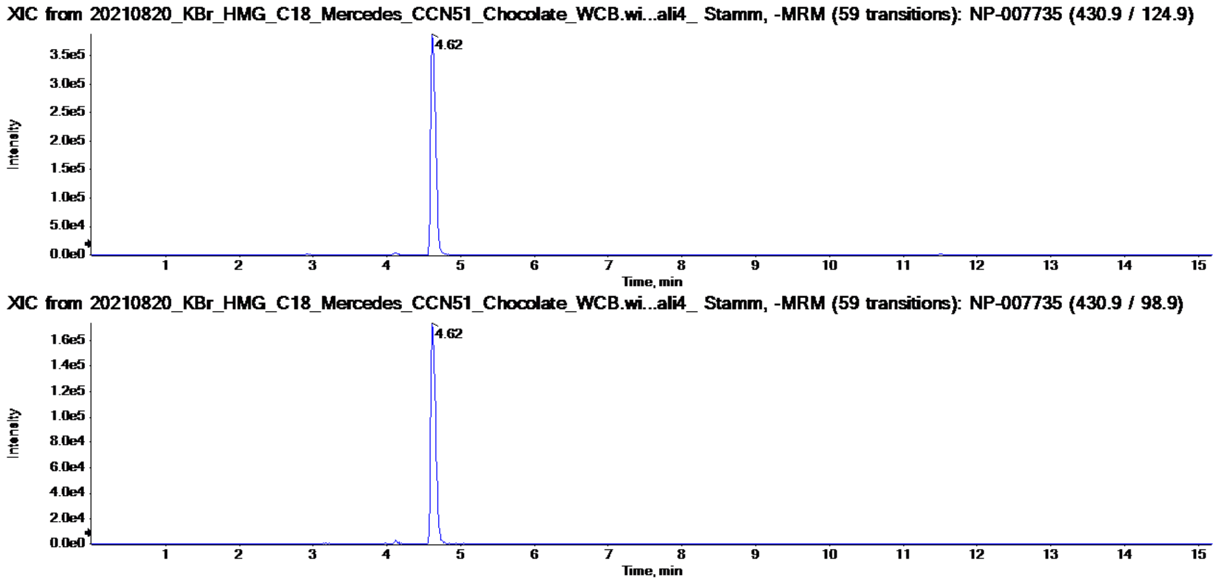

Figure S56: HMG gluc D in calibration stock solution, XIC with traces of quantifier (top) and qualifier (bottom) mrm transitions.

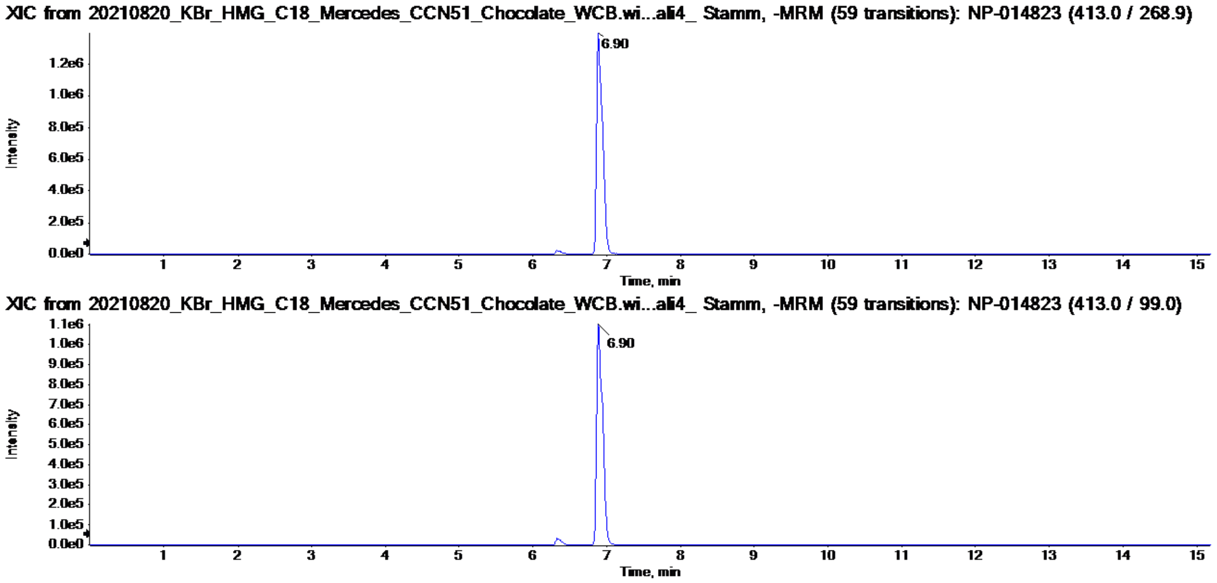

Figure S57: HMG gluc E in calibration stock solution, XIC with traces of quantifier (top) and qualifier (bottom) mrm transitions.

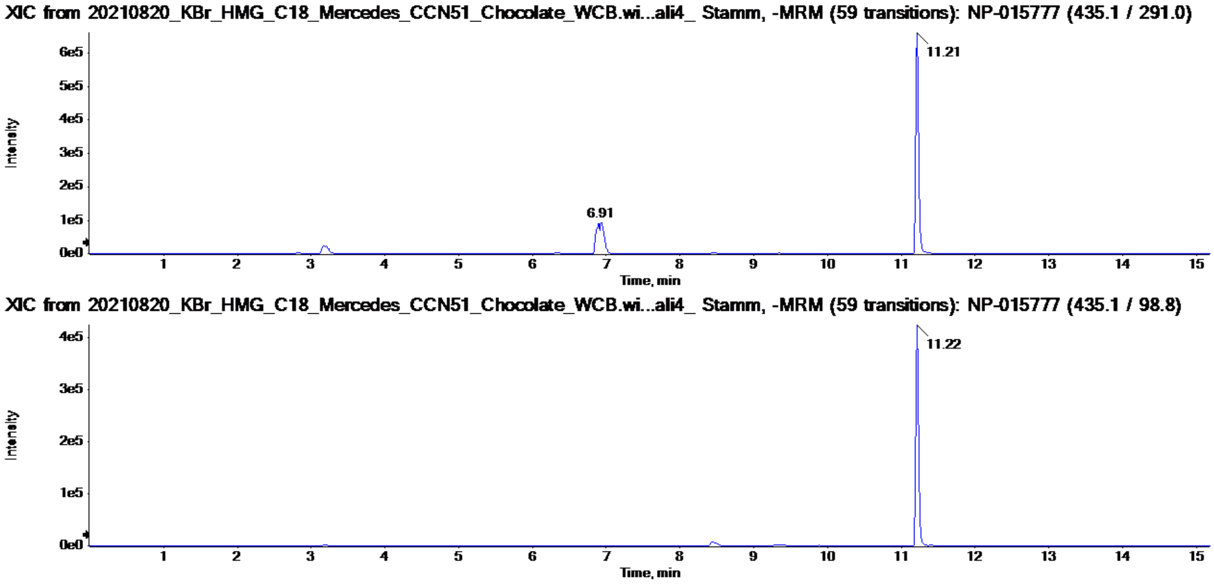

Figure S58: HMG gluc F in calibration stock solution, XIC with traces of quantifier (top) and qualifier (bottom) mrm transitions.

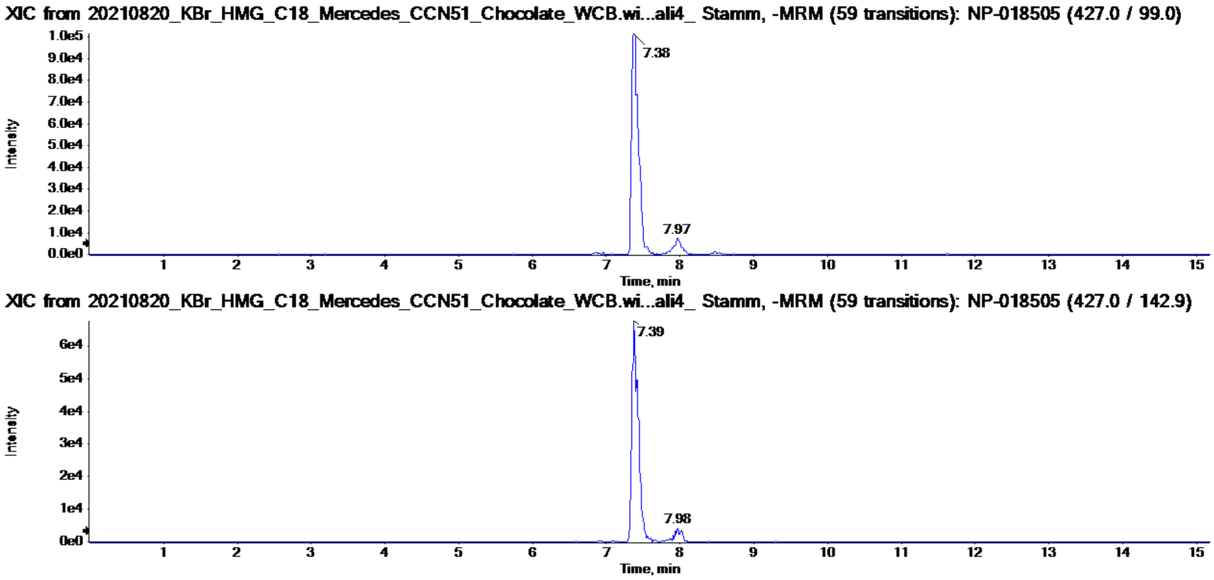

Figure S59: HMG gluc G in calibration stock solution, XIC with traces of quantifier (top) and qualifier (bottom) mrm transitions.

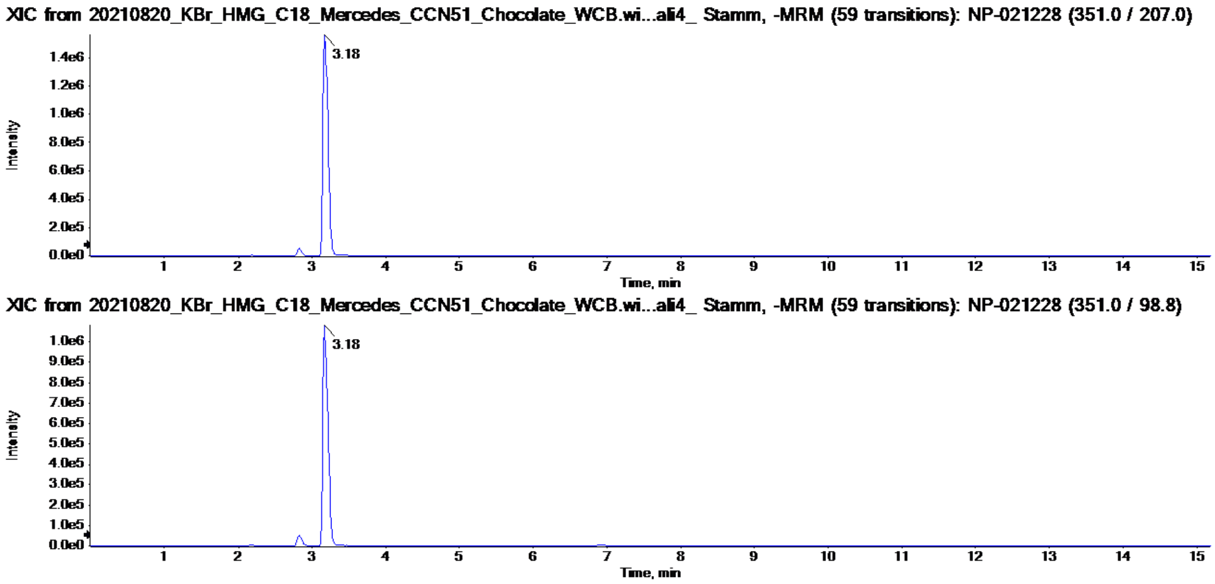

Figure S60: HMG gluc H in calibration stock solution, XIC with traces of quantifier (top) and qualifier (bottom) mrm transitions.

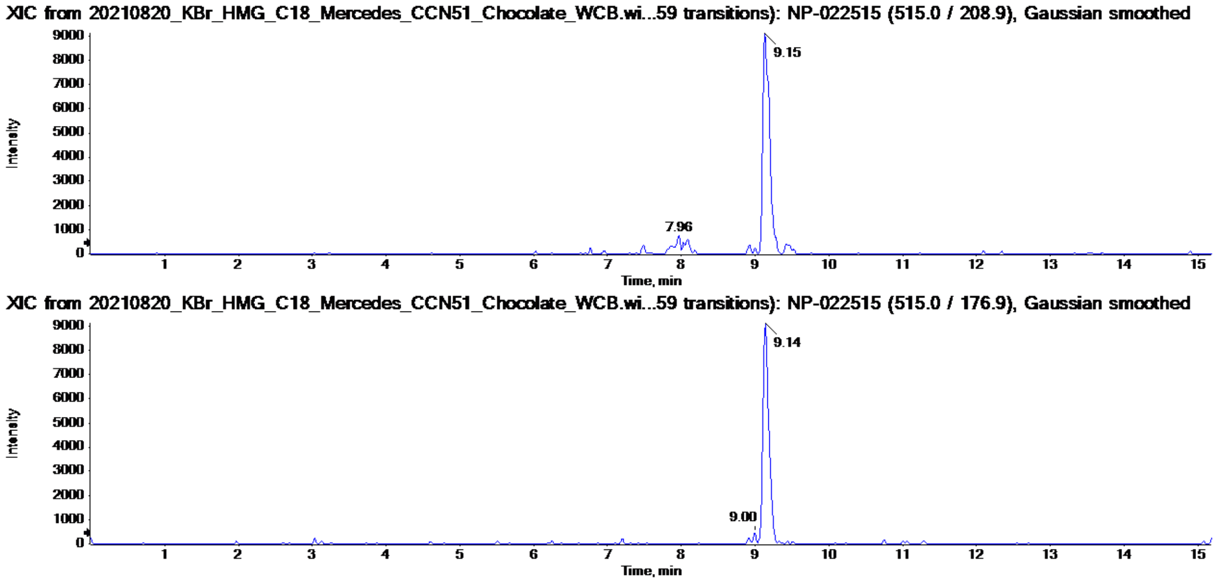

Figure S 61: HMG gluc I (internal standard) in calibration stock solution, XIC with traces of quantifier (top) and qualifier (bottom) mrm transitions.

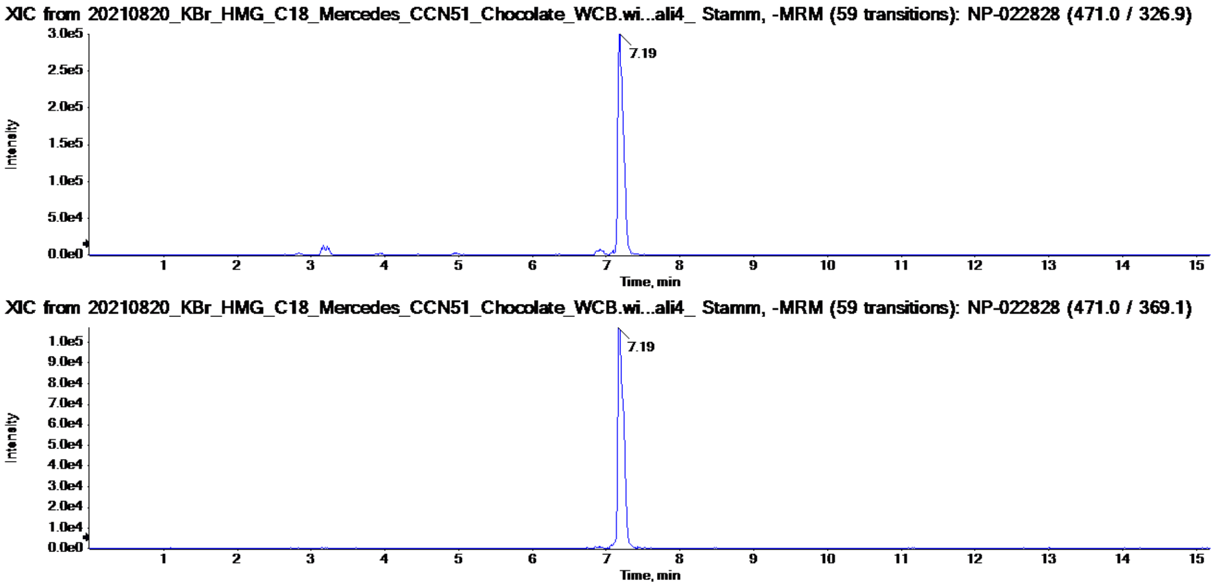

Figure S 62: HMG gluc J in calibration stock solution, XIC with traces of quantifier (top) and qualifier (bottom) mrm transitions.

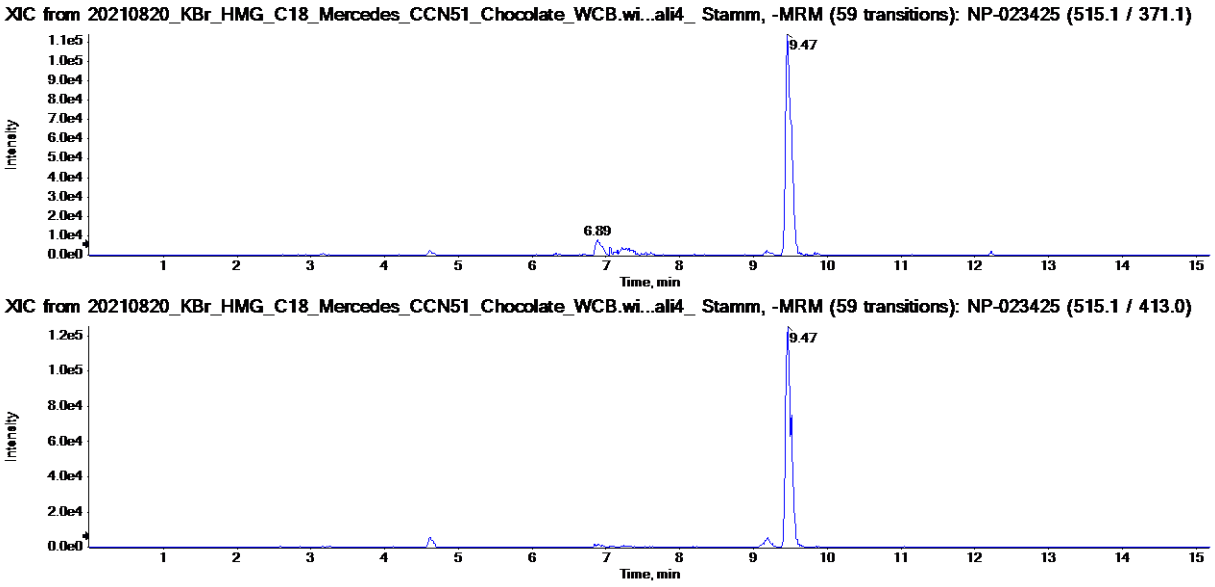

Figure S 63: HMG gluc K in calibration stock solution, XIC with traces of quantifier (top) and qualifier (bottom) mrm transitions.

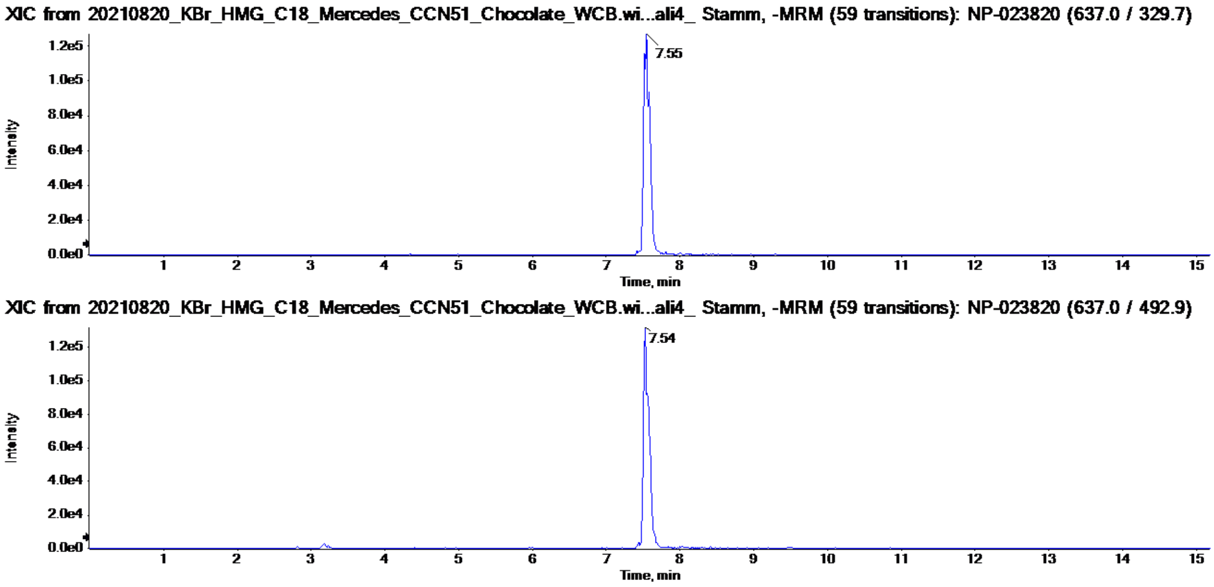

Figure S 64: HMG gluc L in calibration stock solution, XIC with traces of quantifier (top) and qualifier (bottom) mrm transitions.

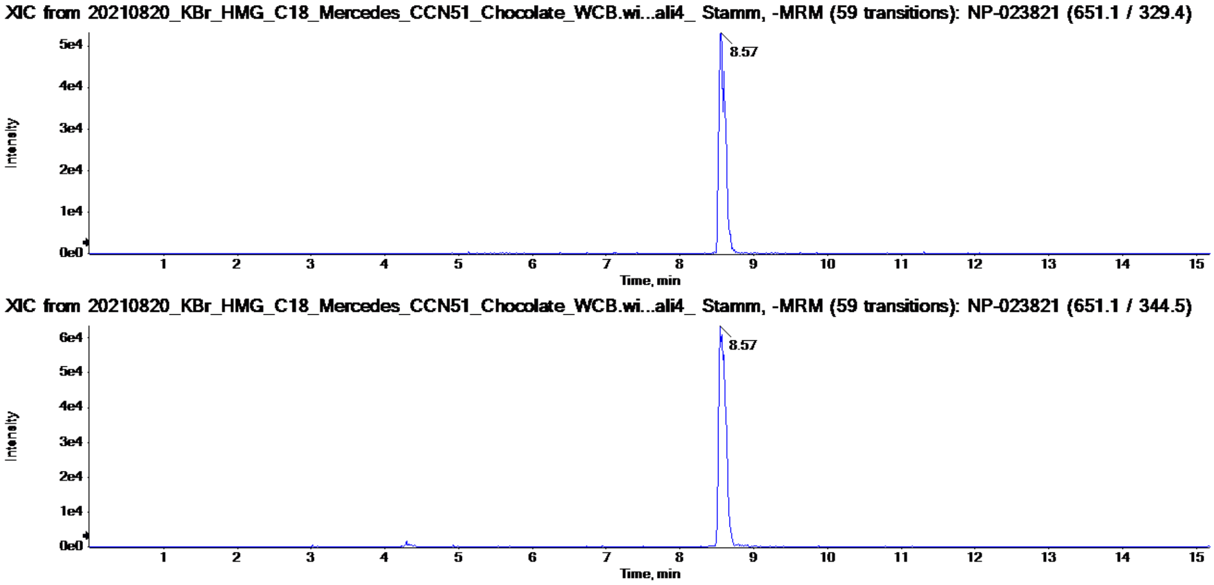

Figure S 65: HMG gluc M in calibration stock solution, XIC with traces of quantifier (top) and qualifier (bottom) mrm transitions.

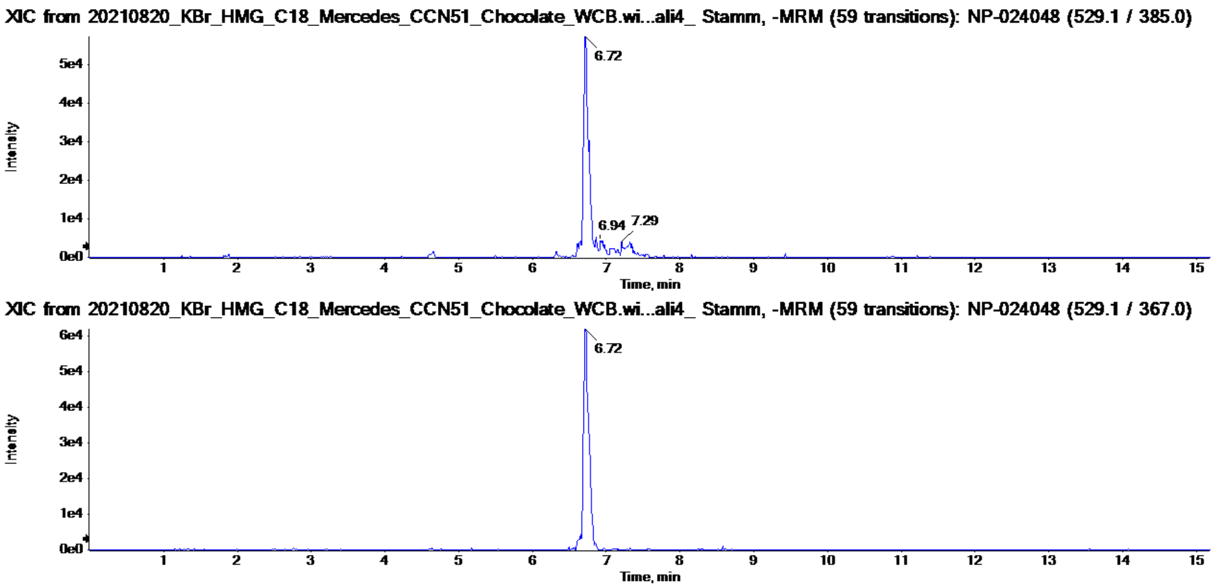

Figure S 66: HMG gluc N in calibration stock solution, XIC with traces of quantifier (top) and qualifier (bottom) mrm transitions.

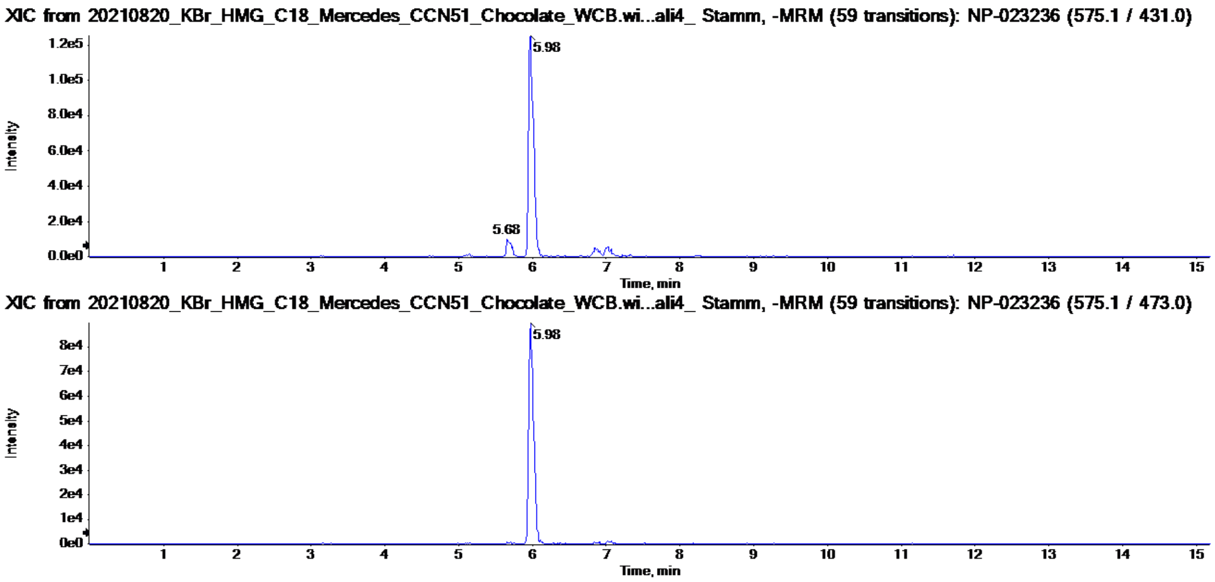

Figure S 67: HMG gluc O in calibration stock solution, XIC with traces of quantifier (top) and qualifier (bottom) mrm transitions.

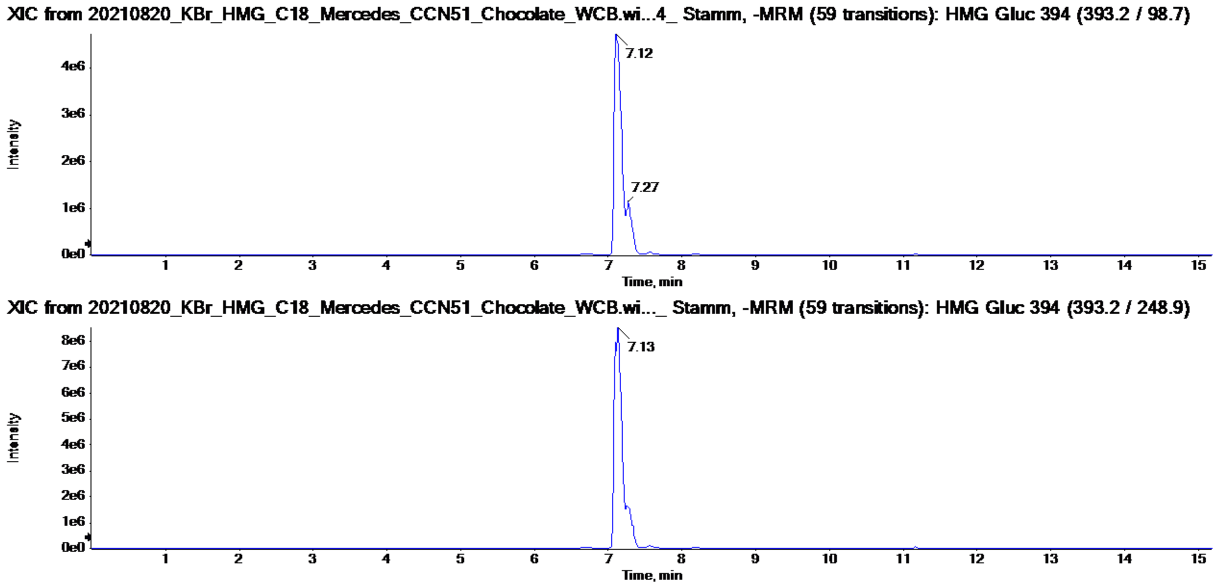

Figure S 68: HMG gluc A isomer 1 in calibration stock solution, XIC with traces of quantifier (top) and qualifier (bottom) mrm transitions.

Mass spectra (UPLC-ESI-qTOF-MS) of standard compounds:

HMG gluc C

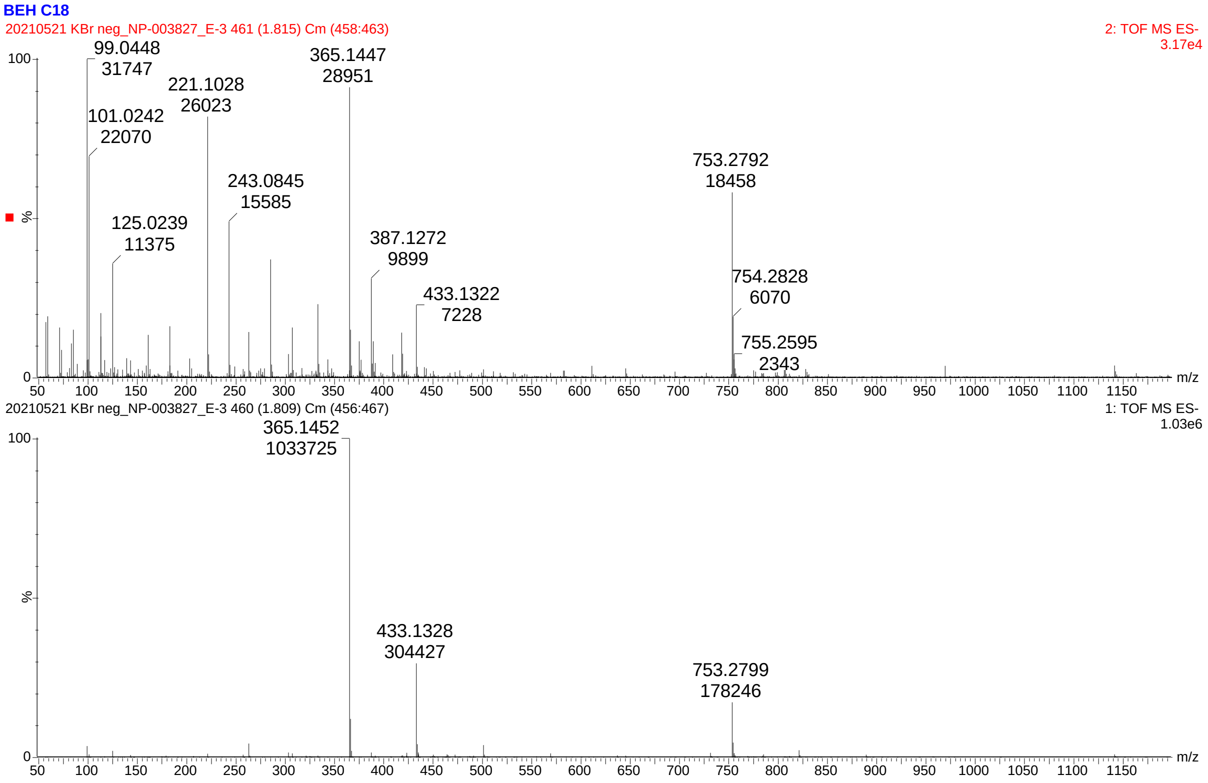

Figure S 69: High collision energy MS^E (top) and low collision energy MS^E (bottom) of standard solution.

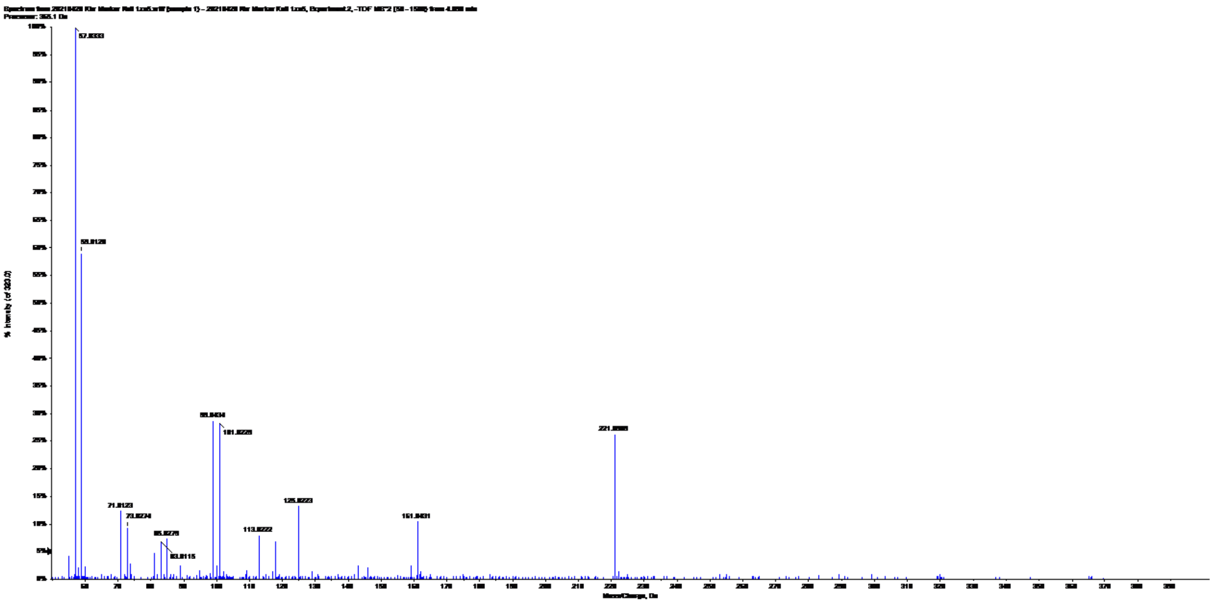

Figure S 70: MS^2 spectrum of standard solution.

HMG gluc A

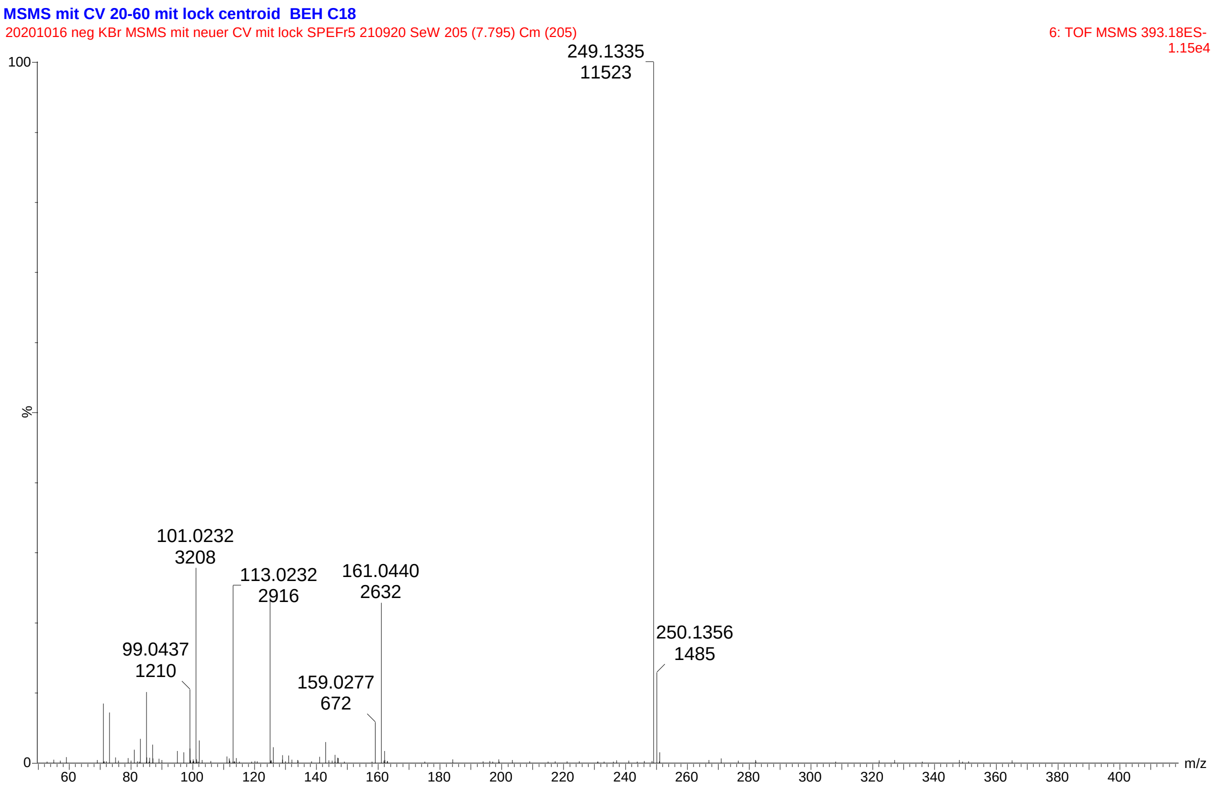

Figure S 71: ToF-MS/MS spectrum with precursor m/z 393.175 of SPE-enriched acetone/water-extract of raw cocoa beans.

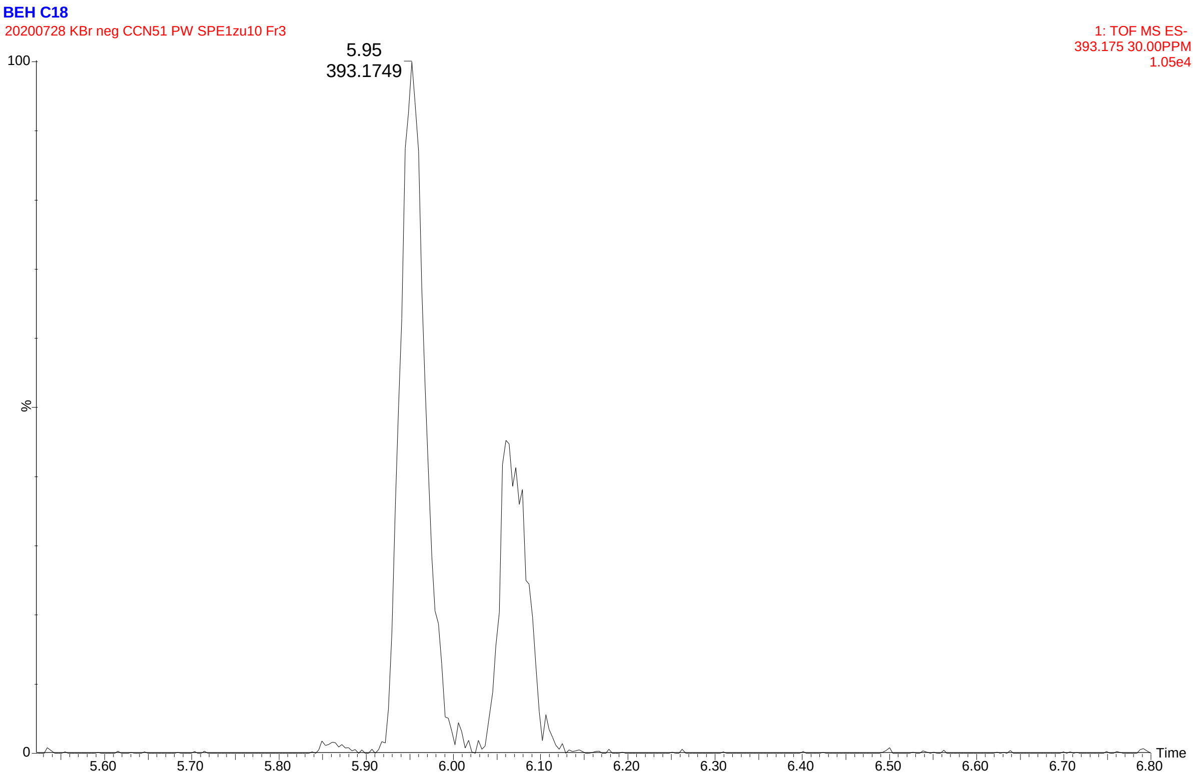

Figure S72: Extracted trace of m/z 393.175 (+/-30 ppm) from UPLC-ToF-MS chromatogram (in MS^E mode) with low collision energy, measured in SPE enriched acetone/water-extract of raw cocoa beans.

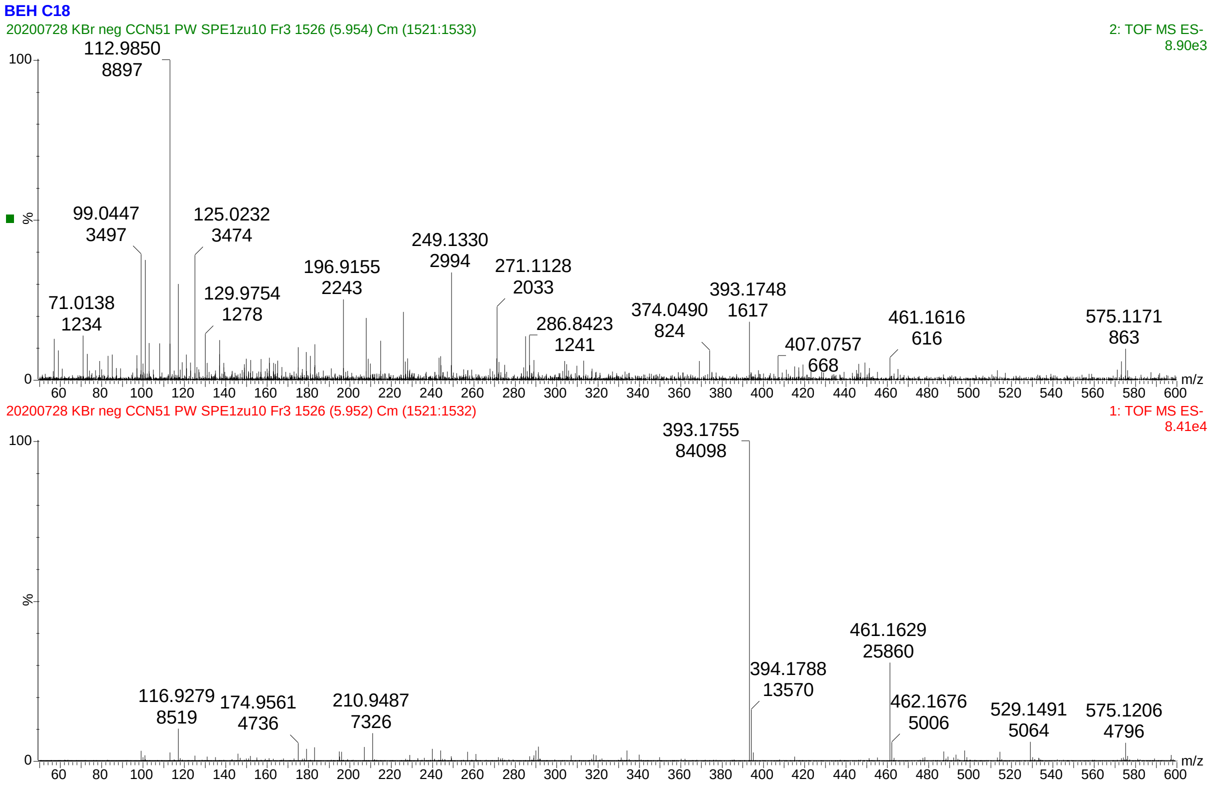

Figure S73: ToF-MS^E spectra of first peak (5.95 min) with high CE (top) and low CE (bottom).

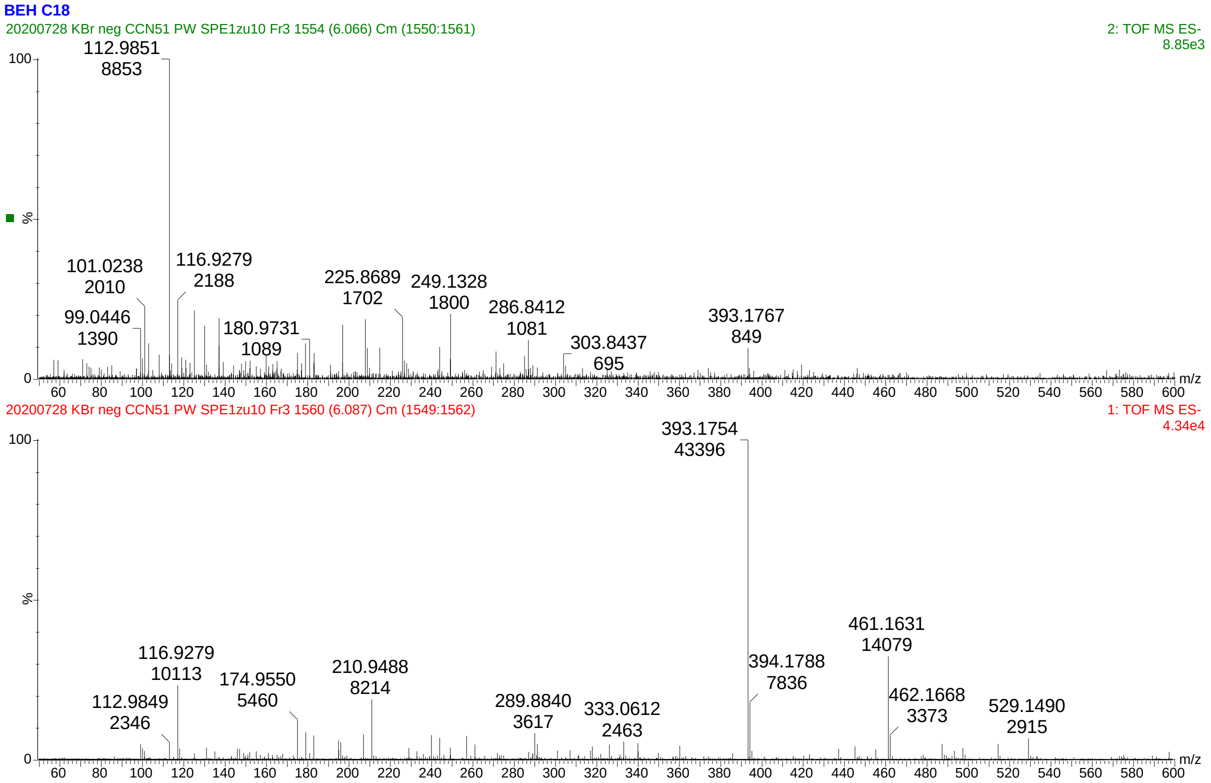

Figure S74: ToF-MS^E spectra of second peak (6.09 min) with high CE (top) and low CE (bottom).

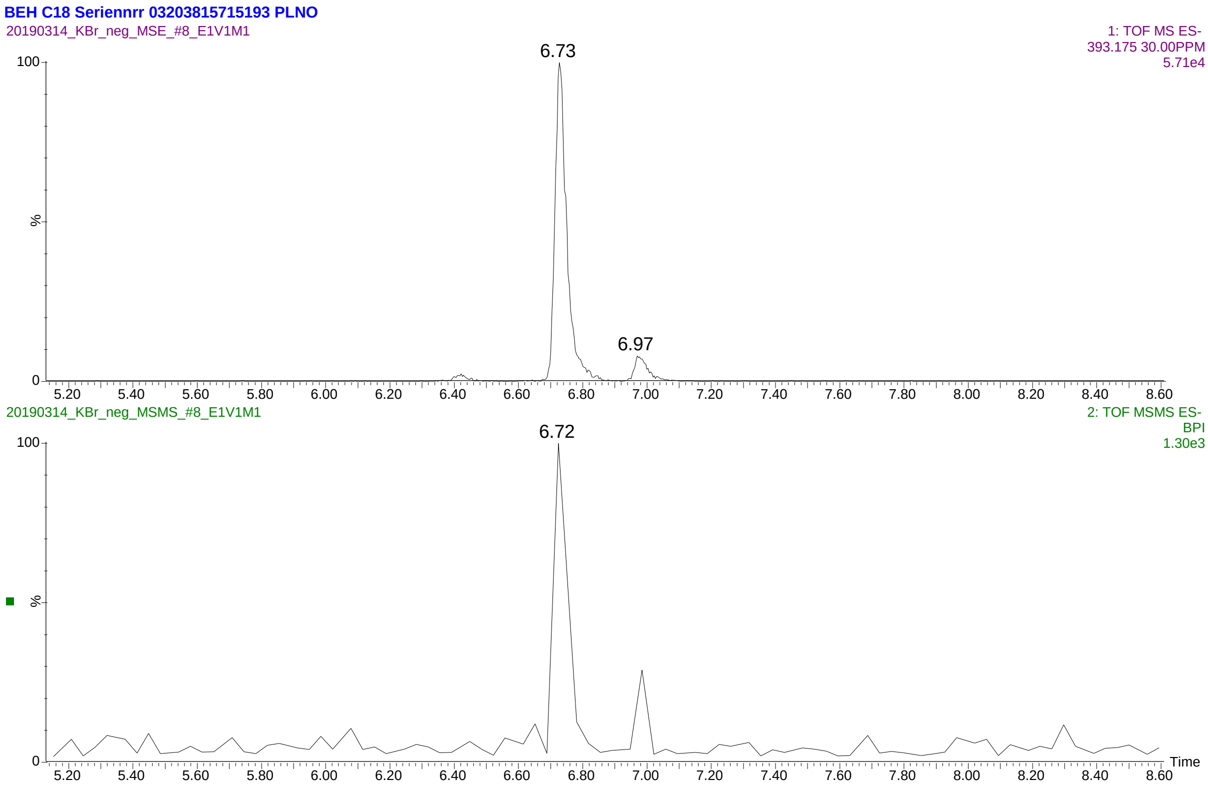

Figure S75: Extracted trace of m/z 393.175 (+/-30 ppm) from UPLC-ToF-MS chromatogram (top, in MS^E mode) with low collision energy and BPI chromatogram of UPLC-ToF-MS run (bottom, in MS^2 scan mode) of methanol/water extract from cocoa sample #8.

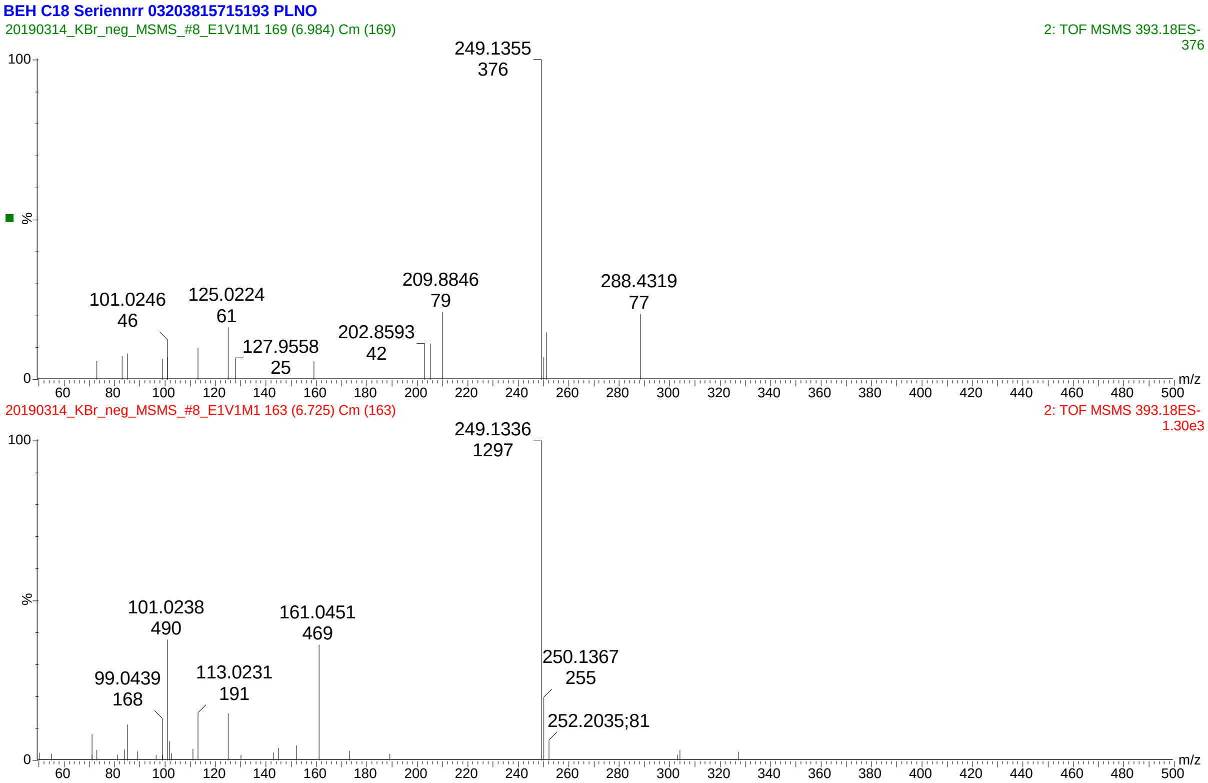

Figure S76: ToF-MS/MS spectra with precursor m/z 393.175 of peaks at 6.98 min (top) and at 6.73 min (bottom) of methanol/water extract from cocoa sample #8.

Comparison HMG gluc A and HMG gluc B:

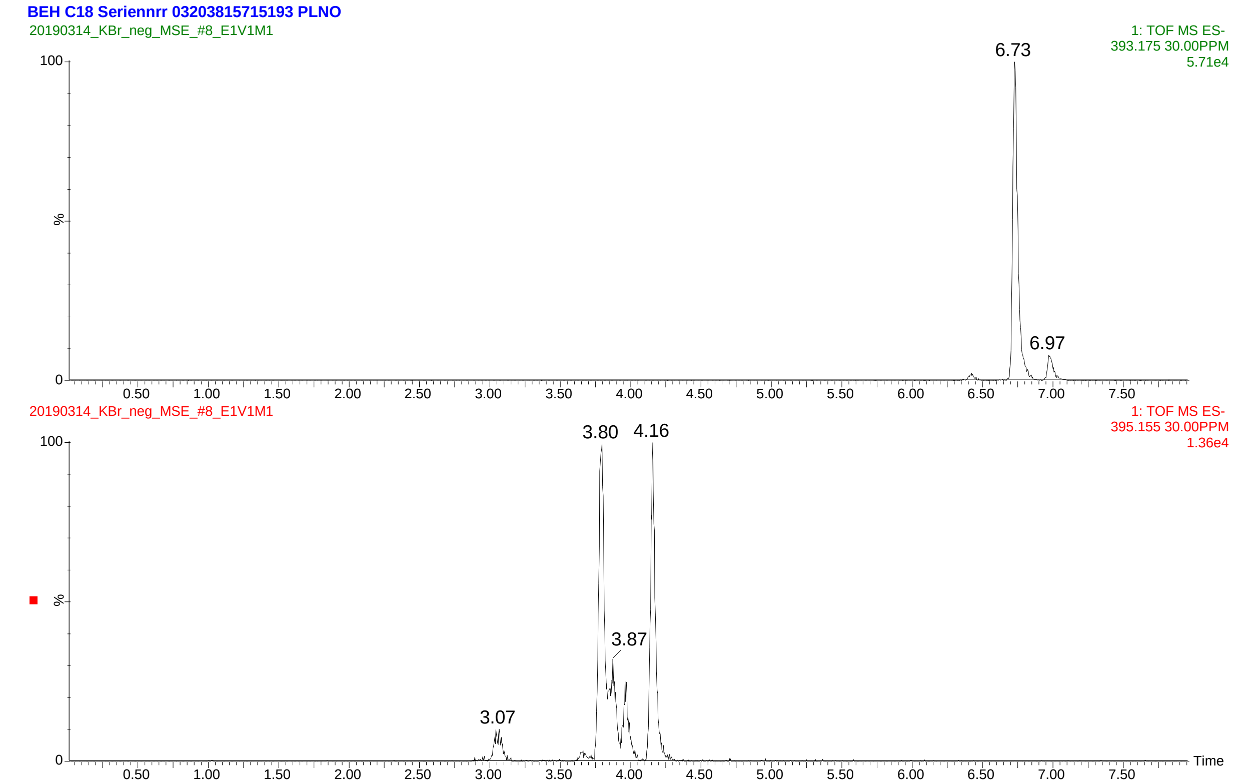

Figure S77: Extracted traces of m/z 393.175 (+/-30 ppm) (top) and of m/z 395.155 (+/- 30ppm) (bottom) from UPLC-ToF-MS chromatogram (in MS^E mode) with low collision energy of methanol/water extract from cocoa sample #8

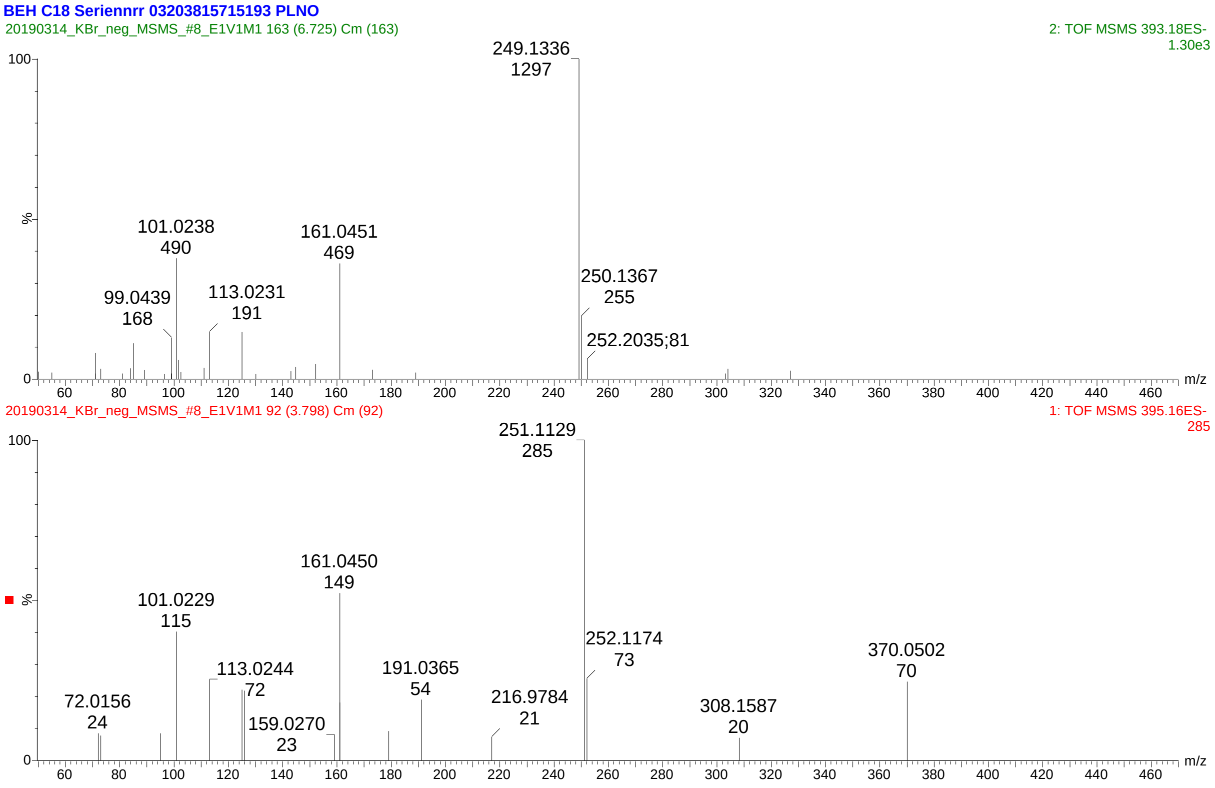

Figure S 78: ToF-MS/MS spectra with precursors m/z 393.175 at 6.73 min (top) and m/z 393.155 at 3.80 min (bottom) of methanol/water extract from cocoa sample #8.

HMG gluc B

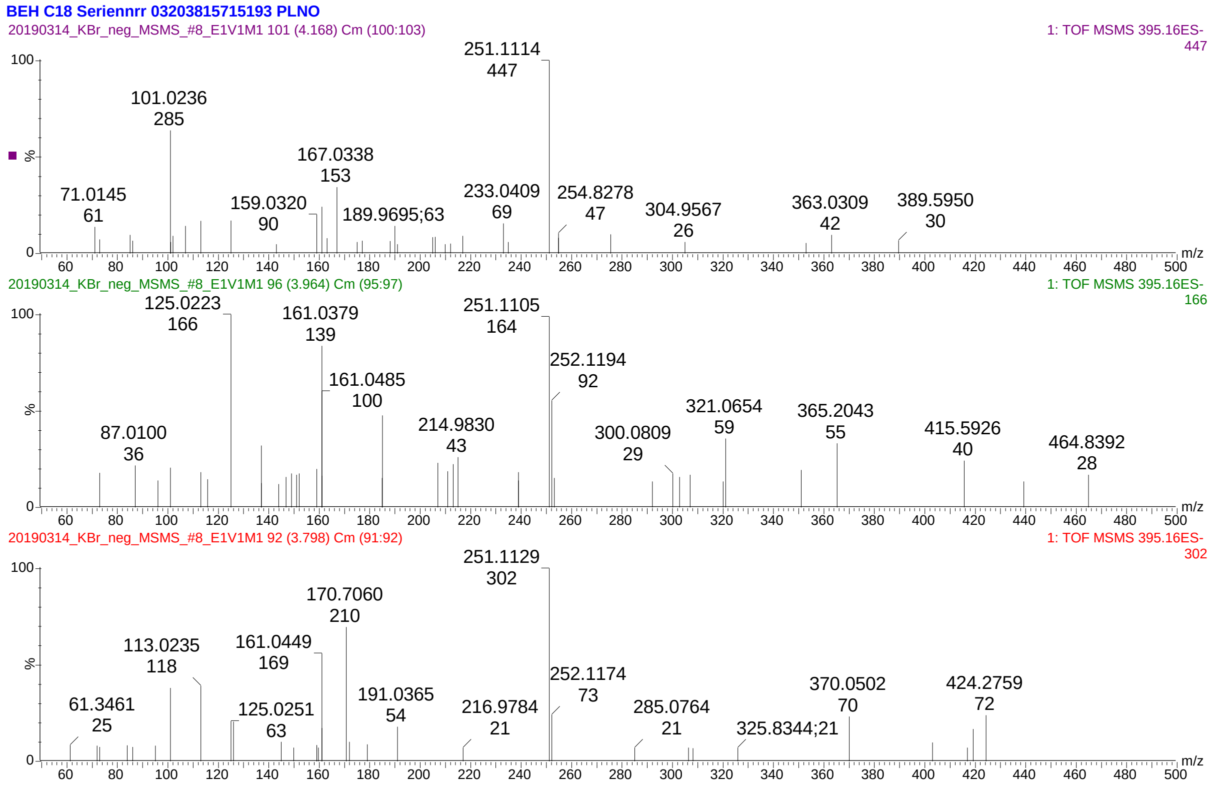

Figure S79: ToF-MS/MS spectra with precursor m/z 395.16 of peaks at 4.17 min (top), at 3.97 min (Centre) and at 3.80 min (bottom) of methanol/water extract from cocoa sample #8.

Enrichment by SPE

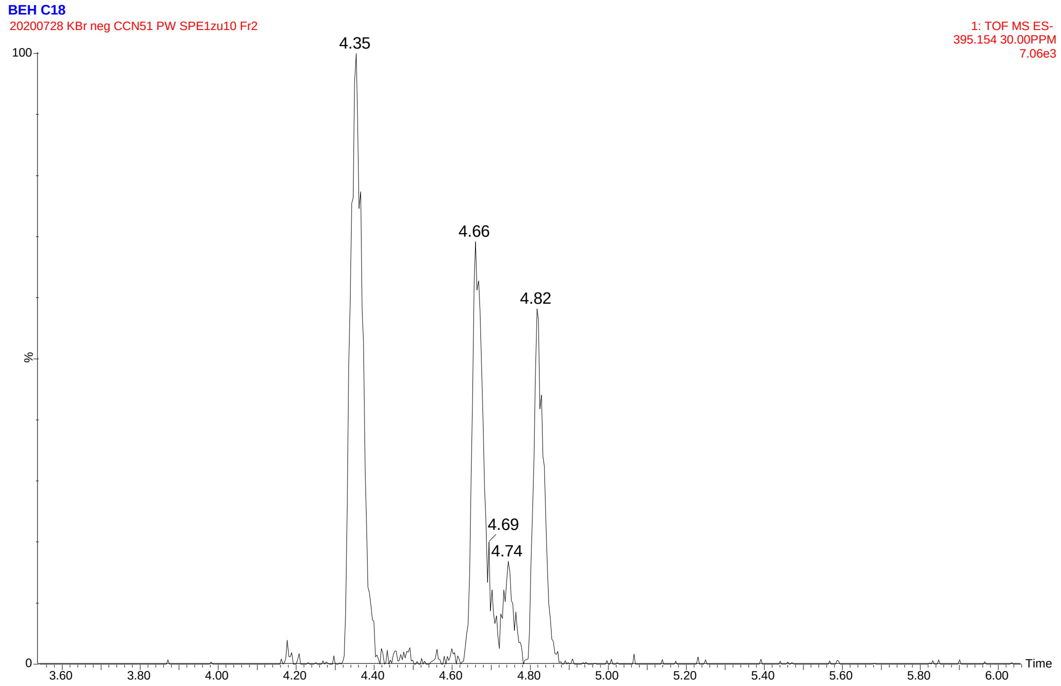

Figure S80: Extracted trace of m/z 395.154 (+/-30 ppm) from UPLC-ToF-MS chromatogram (in MS^E mode) with low collision energy, measured in SPE enriched acetone/water-extract of raw cocoa beans.

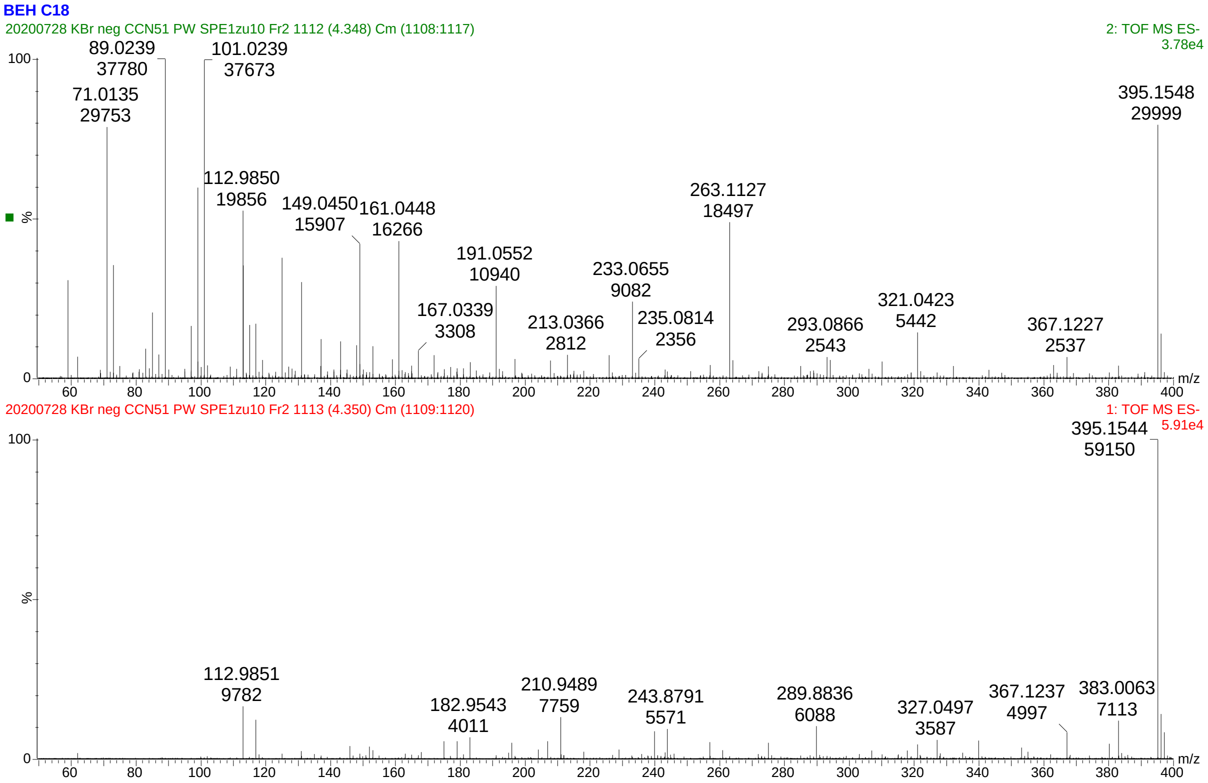

Figure S81: ToF-MS^E spectra of first peak (4.35 min) with high CE (top) and low CE (bottom).

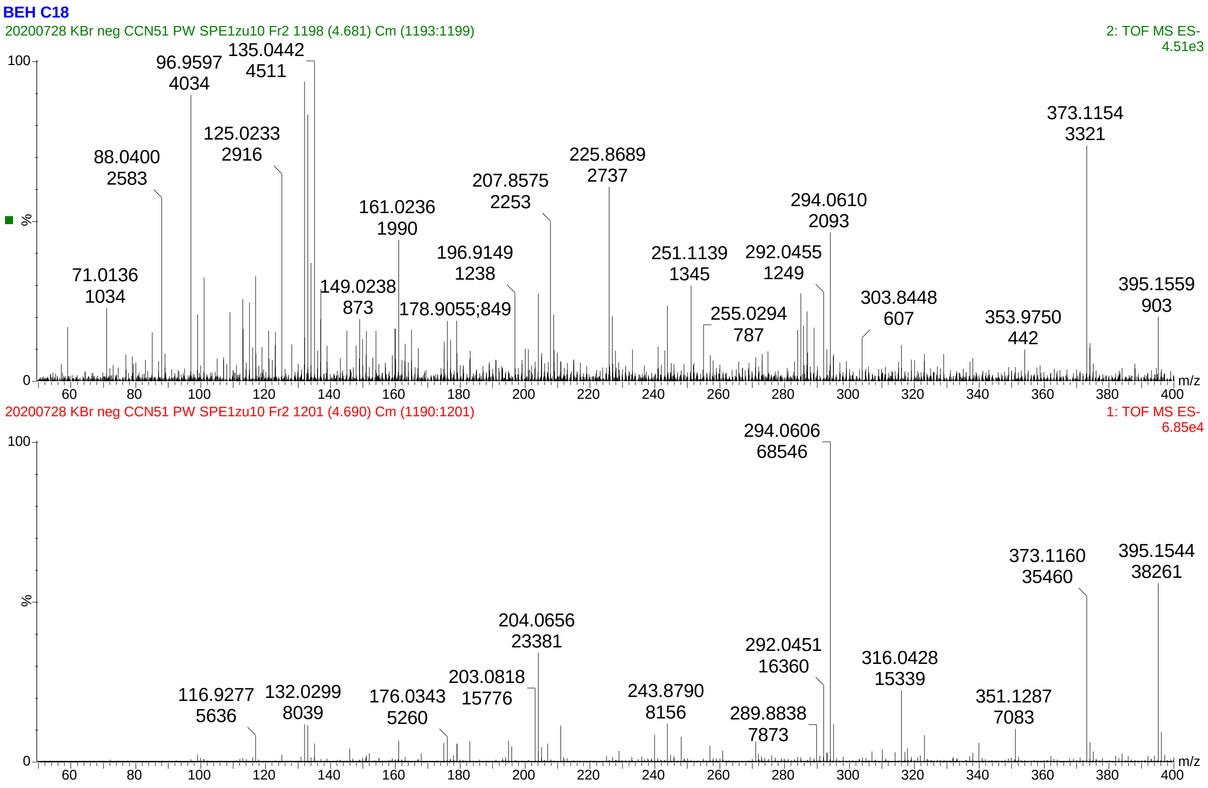

Figure S82: ToF-MS^E spectra of 2nd peak (4.68 min) with high CE (top) and low CE (bottom).

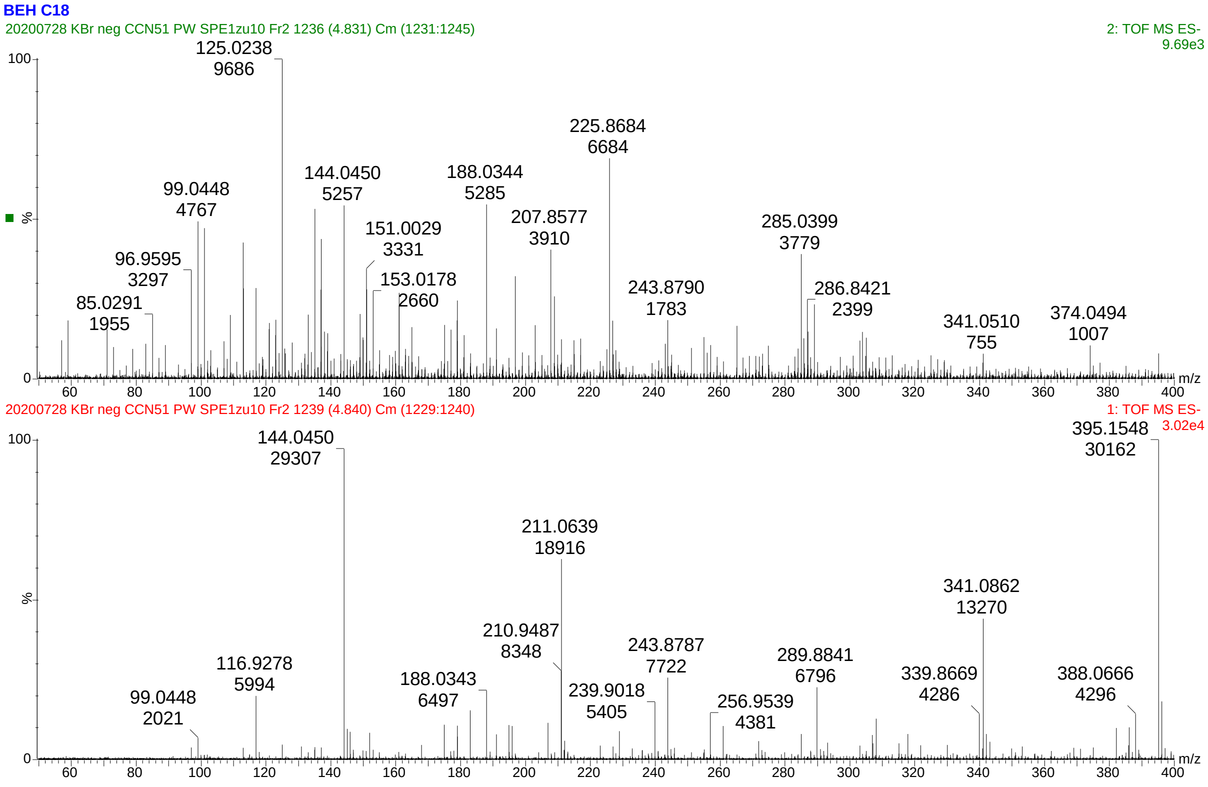

Figure S83: ToF-MS^E spectra of 3rd peak (4.84 min) with high CE (top) and low CE (bottom).

Enrichment by Prep HPLC

Isomer 1

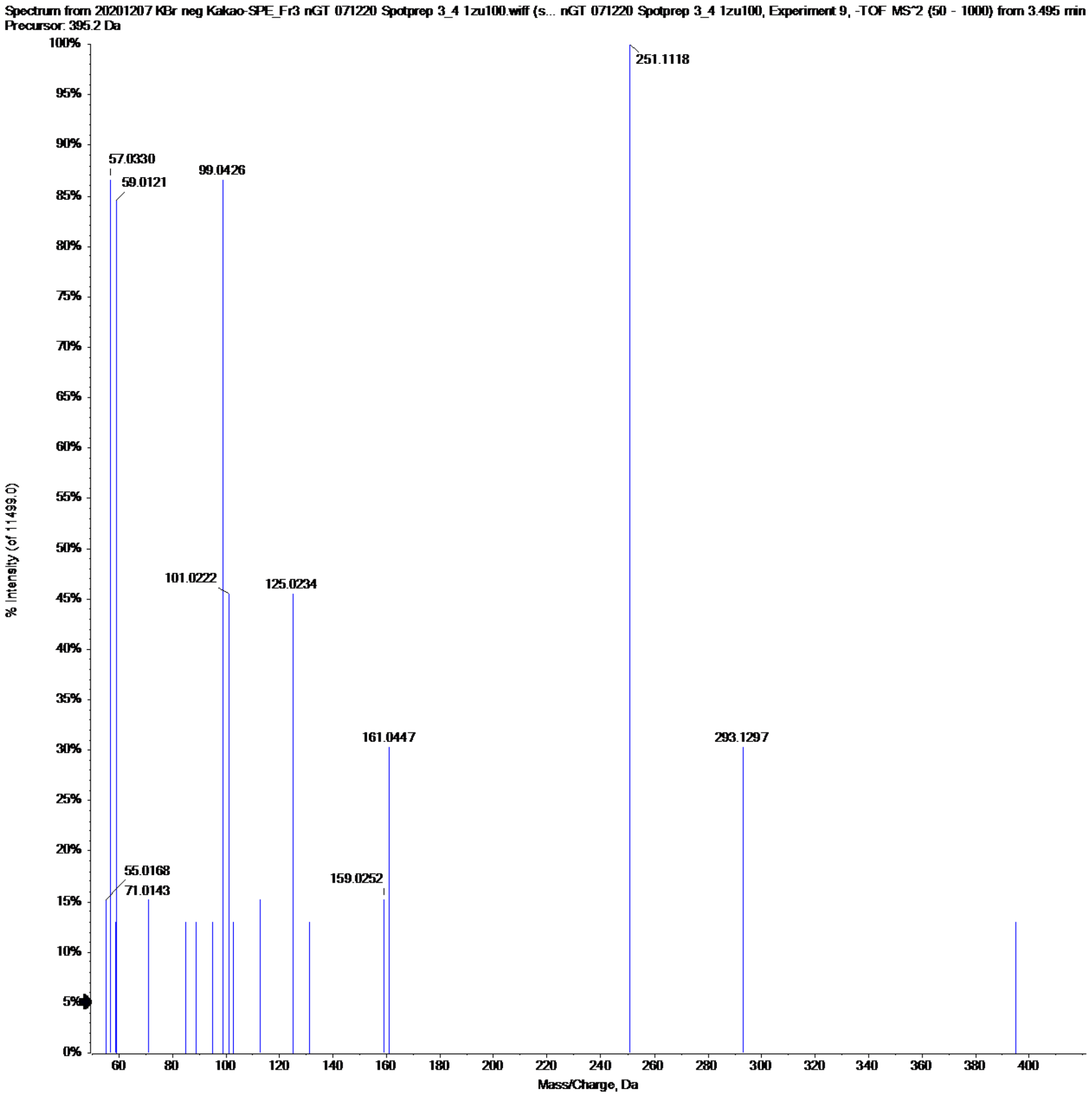

Figure S 84: qToF-MS/MS spectrum of first isomer (3.5 min) with precursors m/z 395.2, measured in SPE/ HPLC enriched acetone/water-extract of raw cocoa beans.

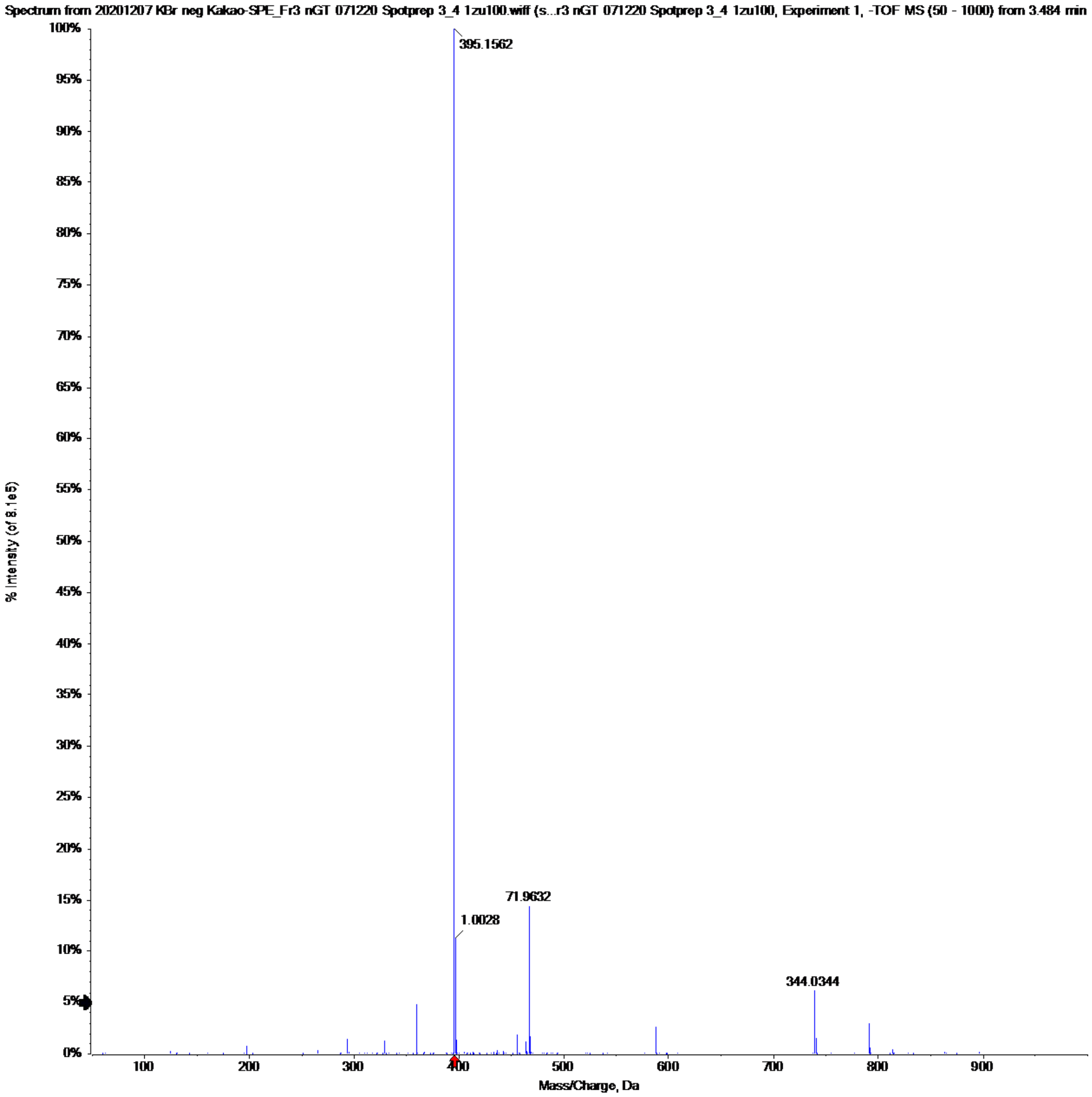

Figure S 85: qToF-MS spectrum (survey scan) of first isomer (3.5 min), measured in SPE/ HPLC enriched acetone/water-extract of raw cocoa beans.

Isomer 2

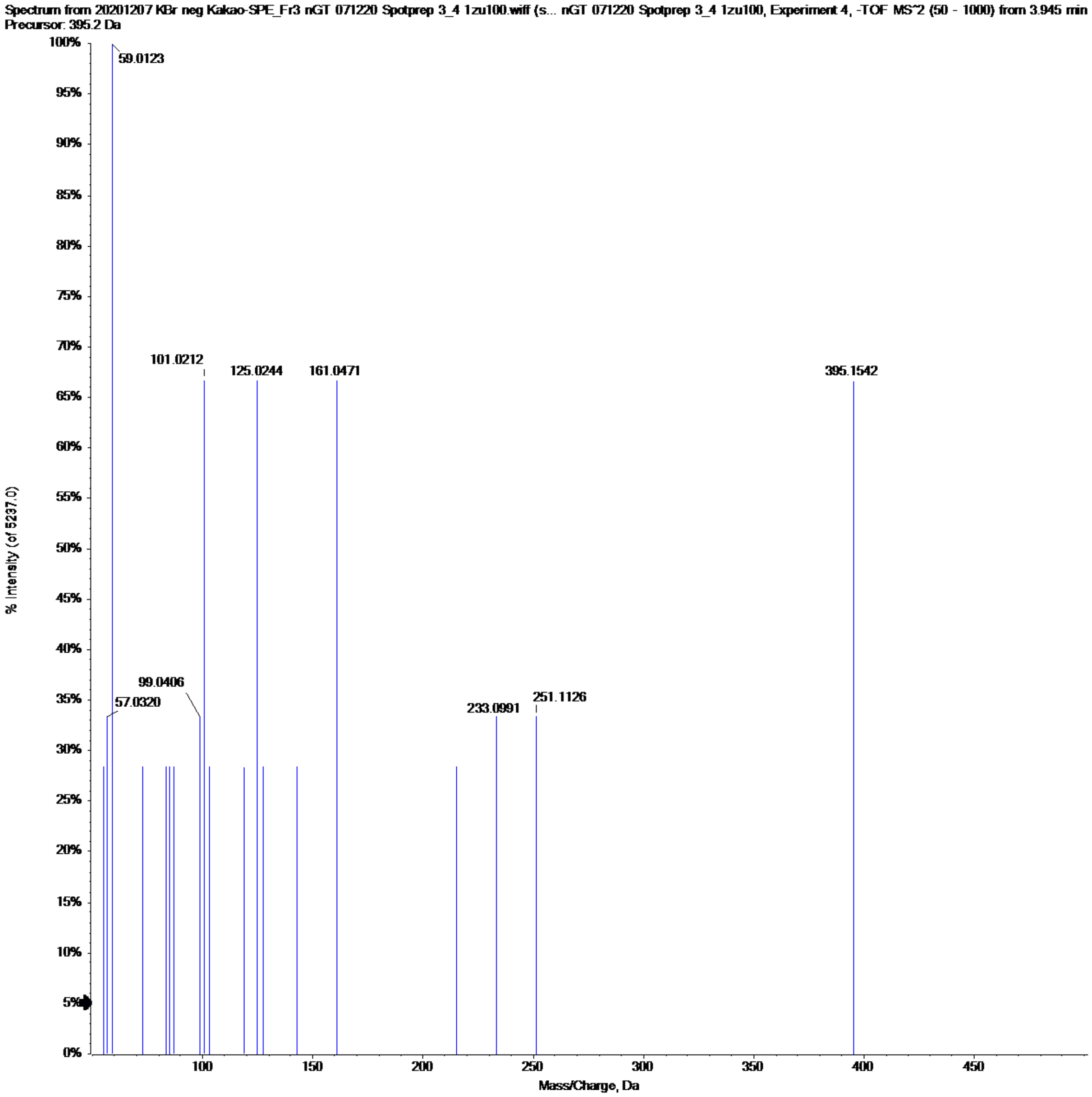

Figure S 86: qToF-MS/MS spectrum of second isomer (3.9 min) with precursors m/z 395.2, measured in SPE/ HPLC enriched acetone/water-extract of raw cocoa beans.

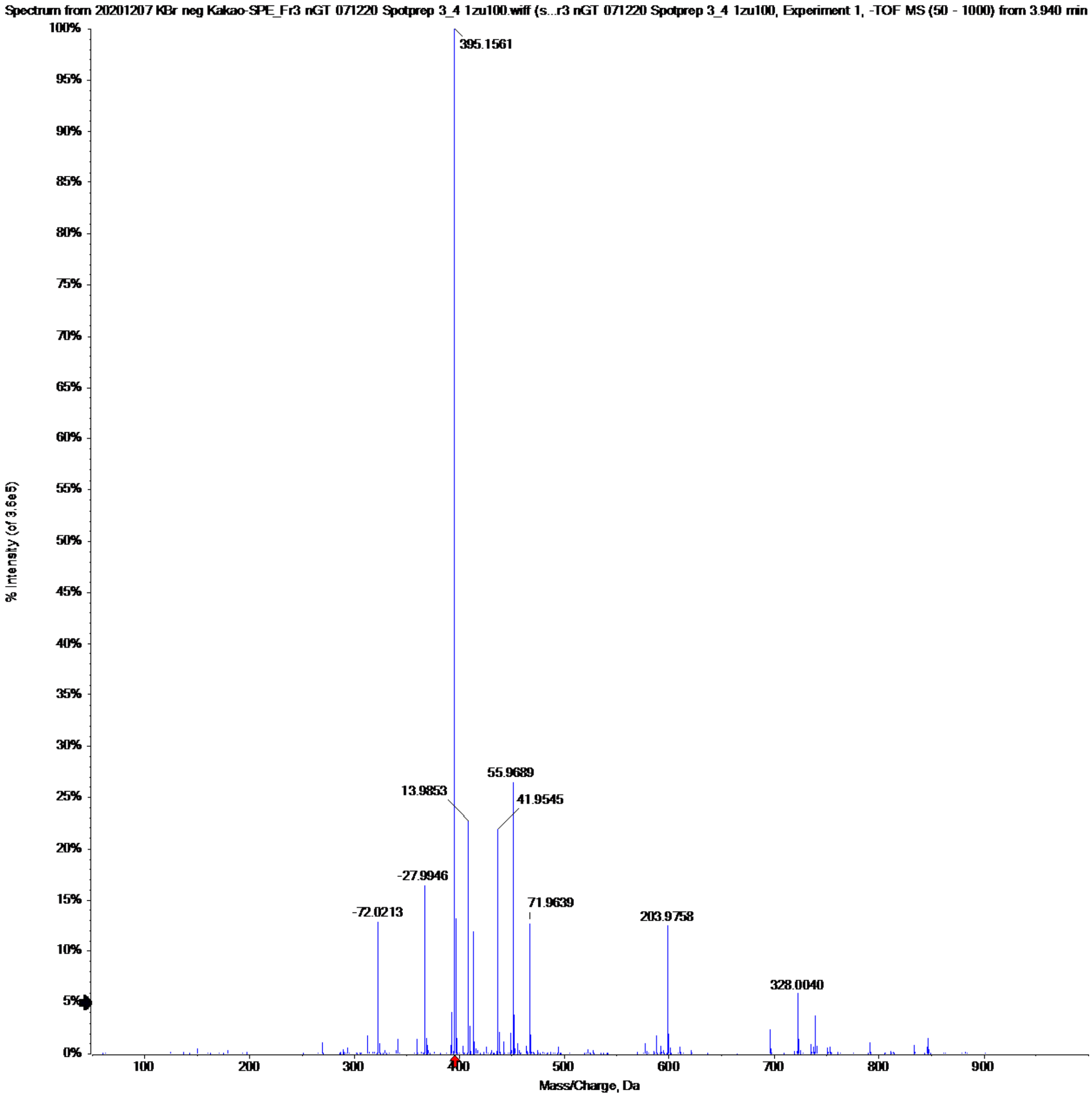

Figure S 87: qToF-MS spectrum (survey scan) of second isomer (3.9 min), measured in SPE/ HPLC enriched acetone/water-extract of raw cocoa beans.

HOJA sulfate

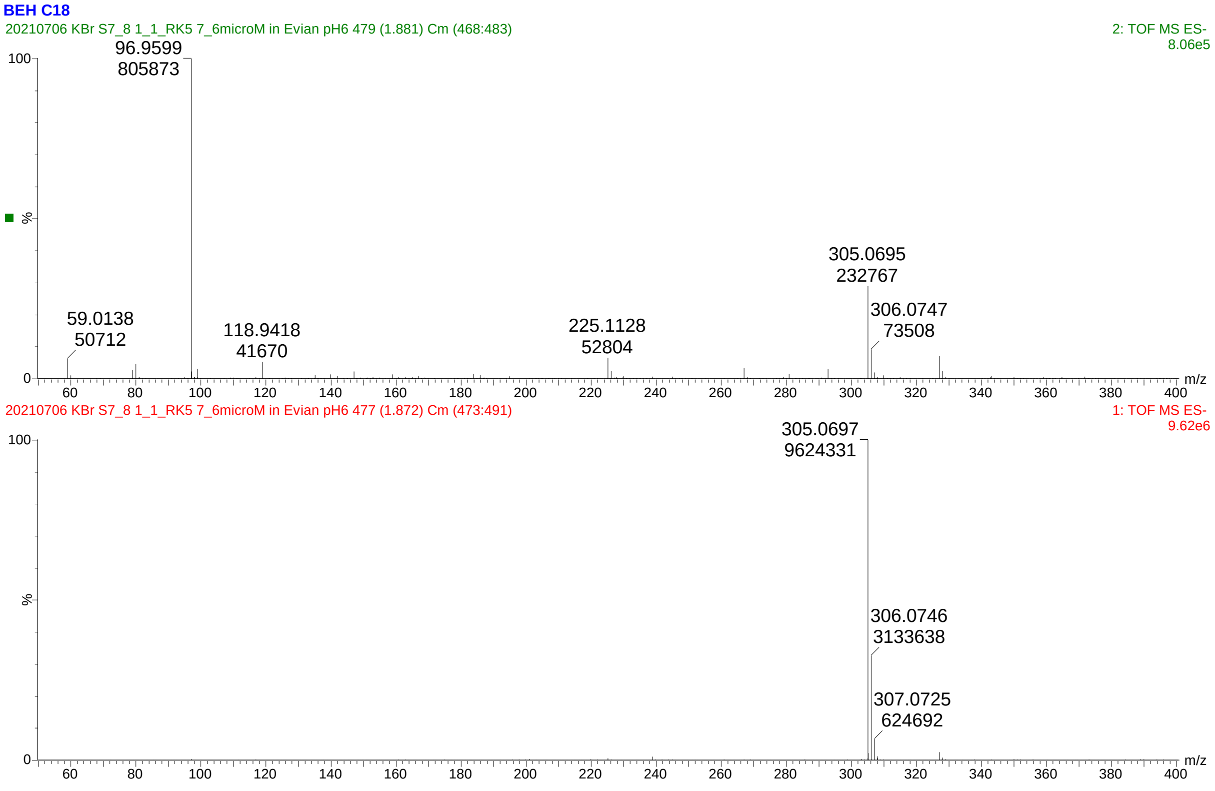

Figure S 88: ToF-MS^E spectra of HOJA sulfate standard solution with high CE (top) and low CE (bottom).

NP007735: HMG gluc D

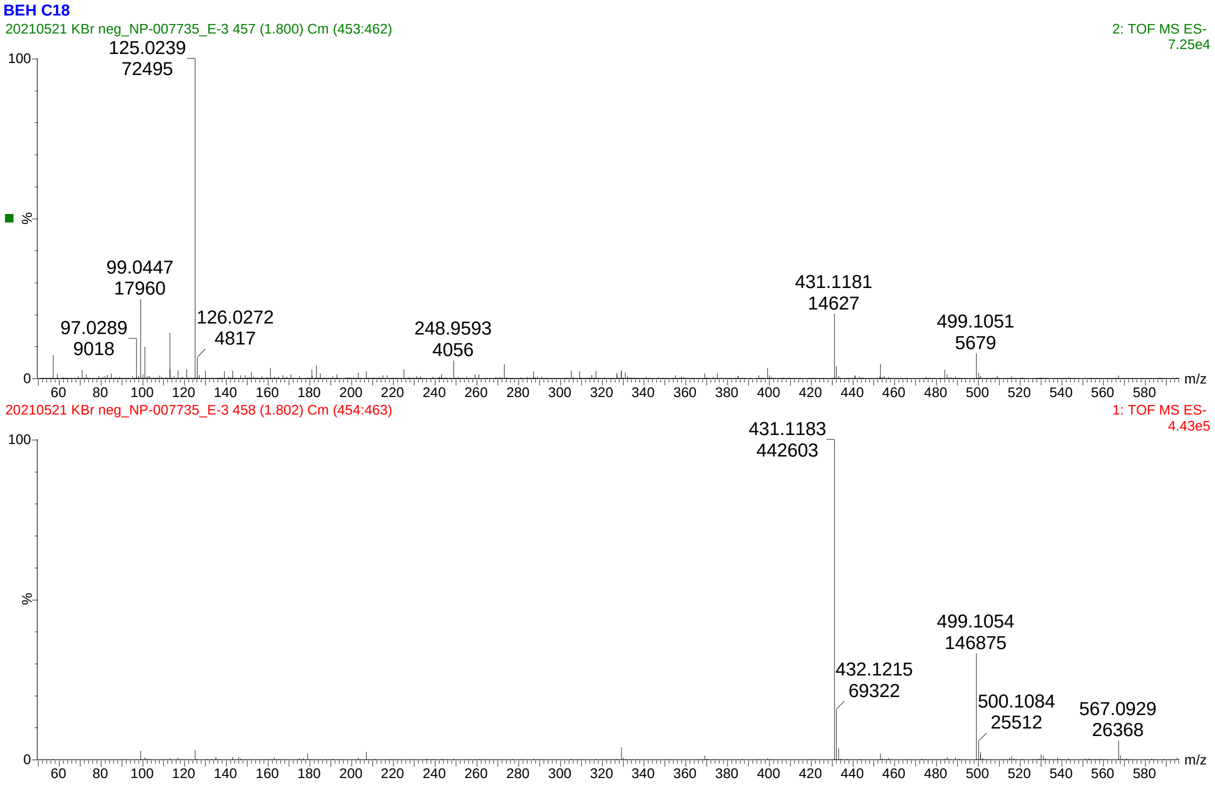

Figure S 89: ToF-MS^E spectra of HMG gluc D standard solution with high CE (top) and low CE (bottom).

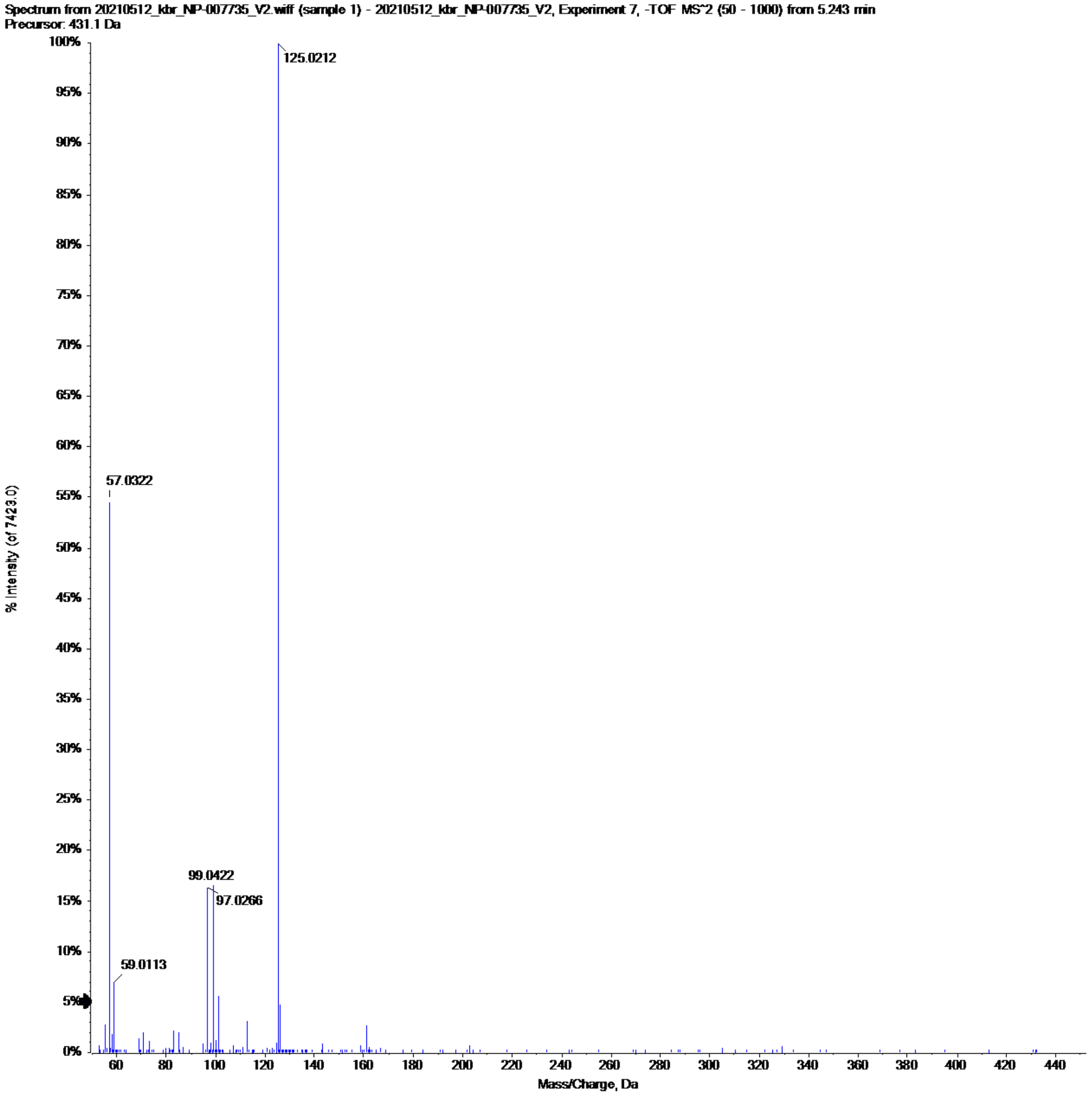

Figure S 90: qToF-MS/MS spectrum of standard solution.

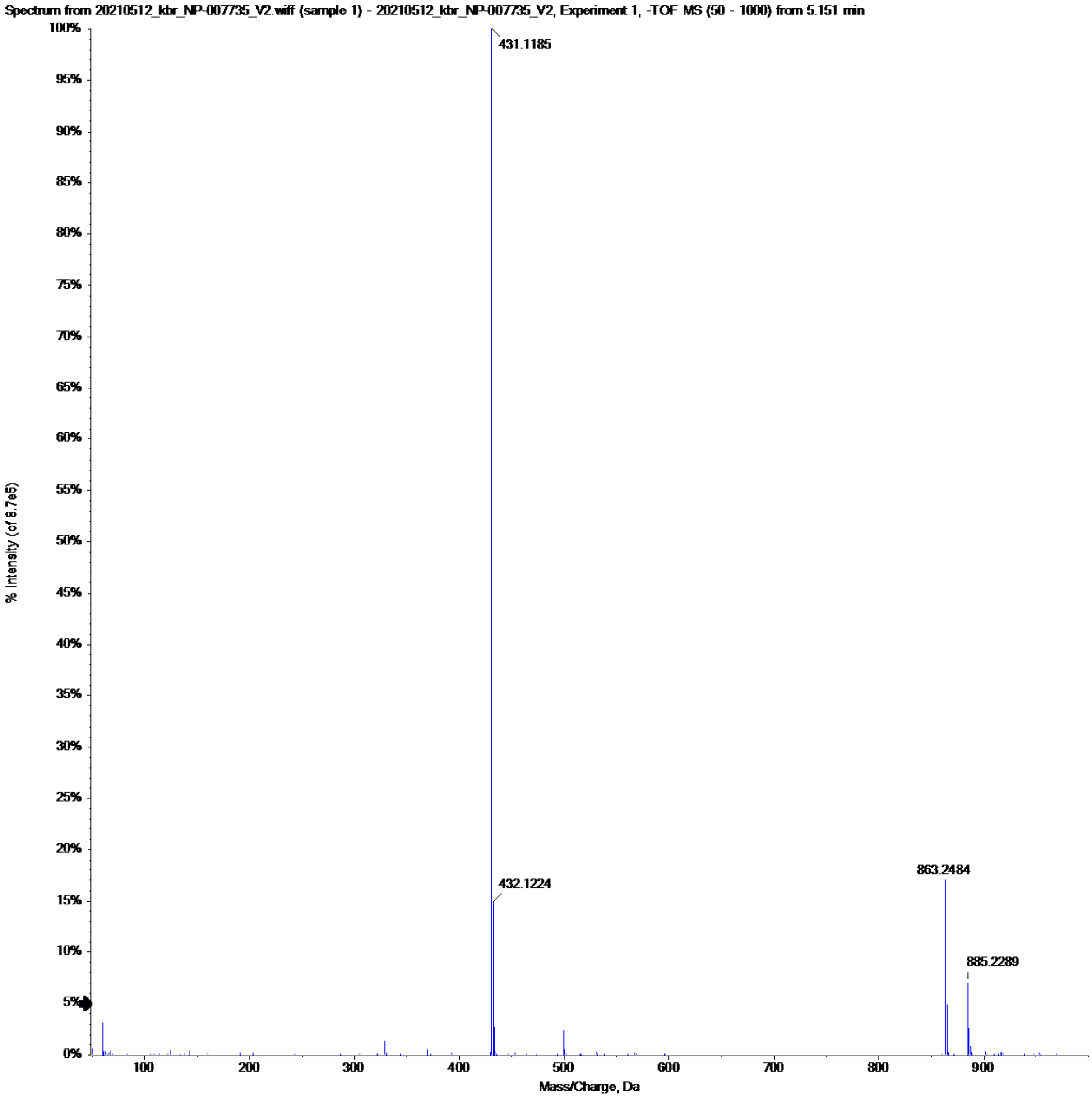

Figure S 91: qToF-MS spectrum (survey scan) of standard solution.

NP014823: HMG gluc E

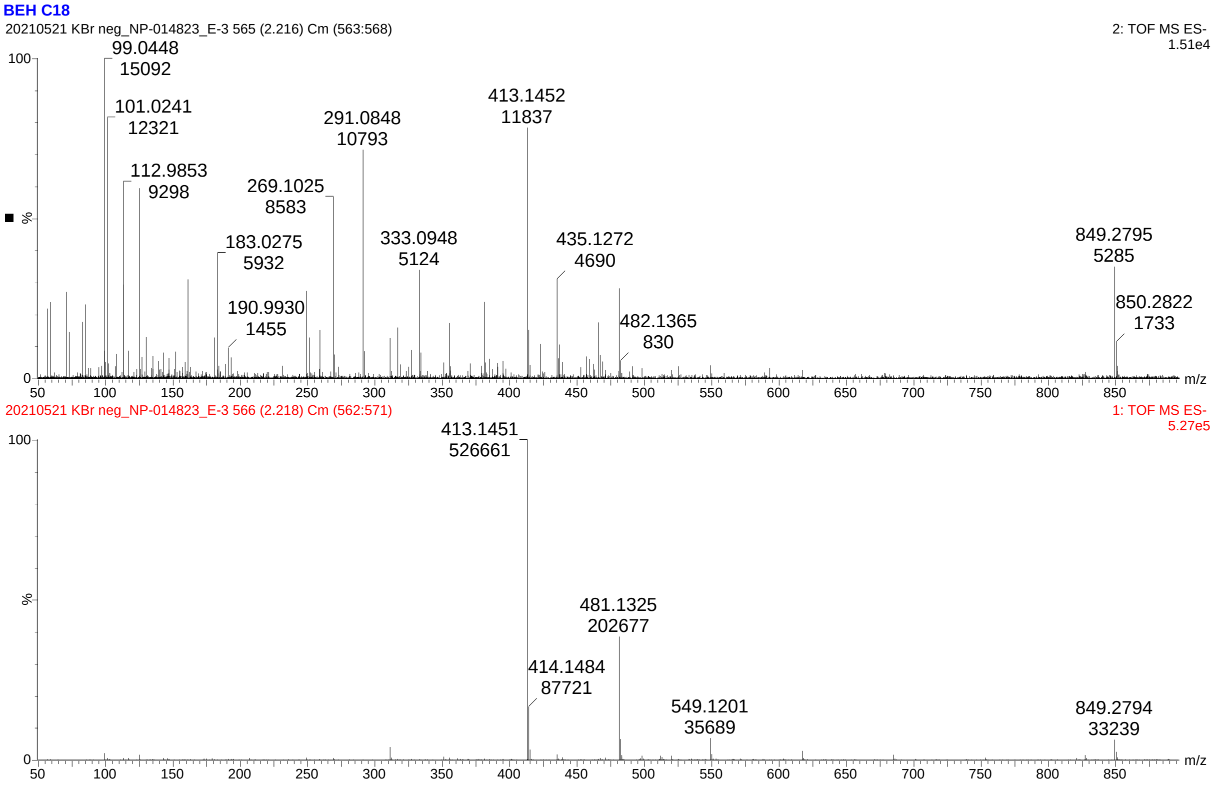

Figure S 92: ToF-MS^E spectra of HMG gluc E standard solution with high CE (top) and low CE (bottom).

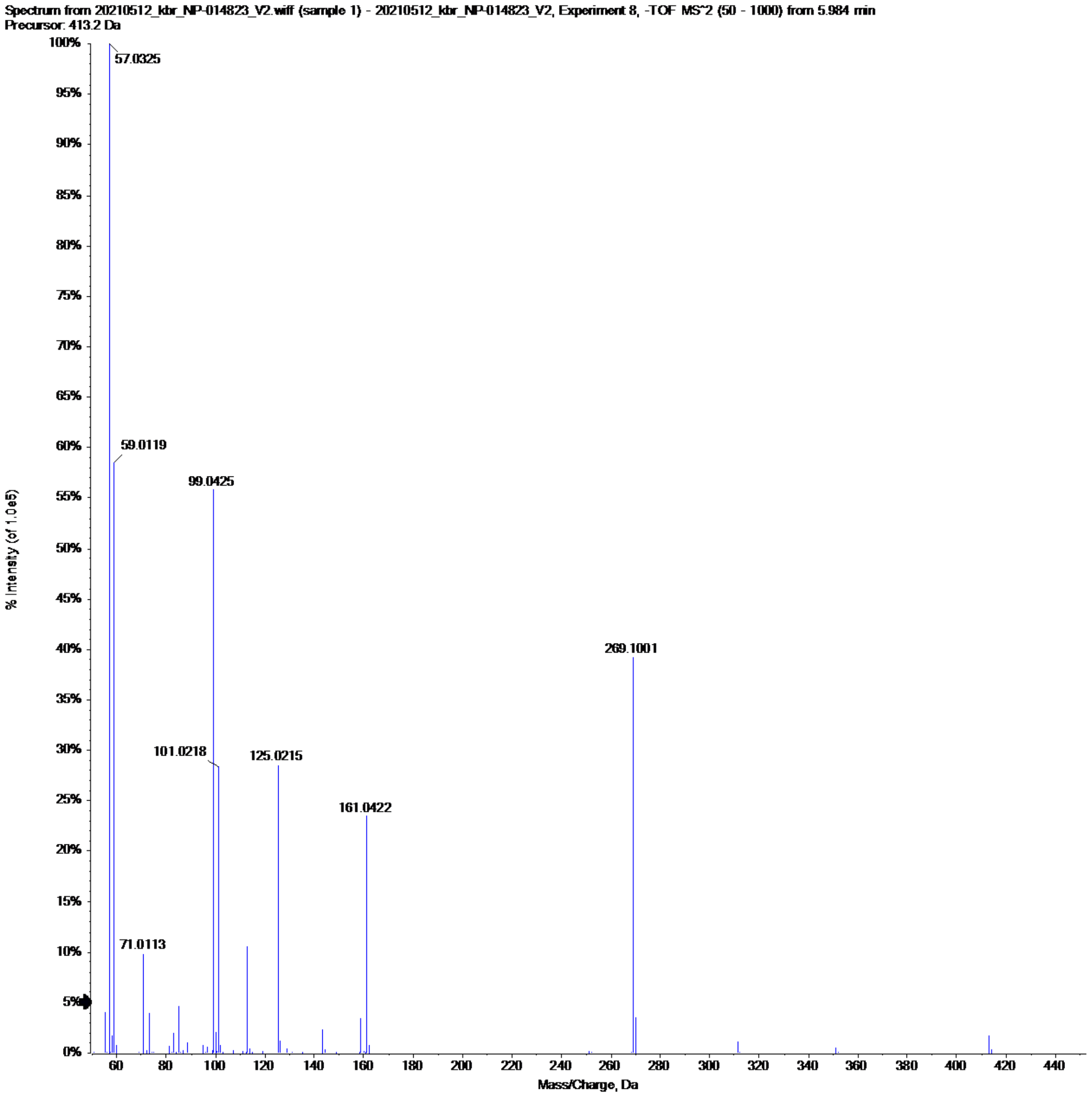

Figure S 93: qToF-MS/MS spectrum of standard solution.

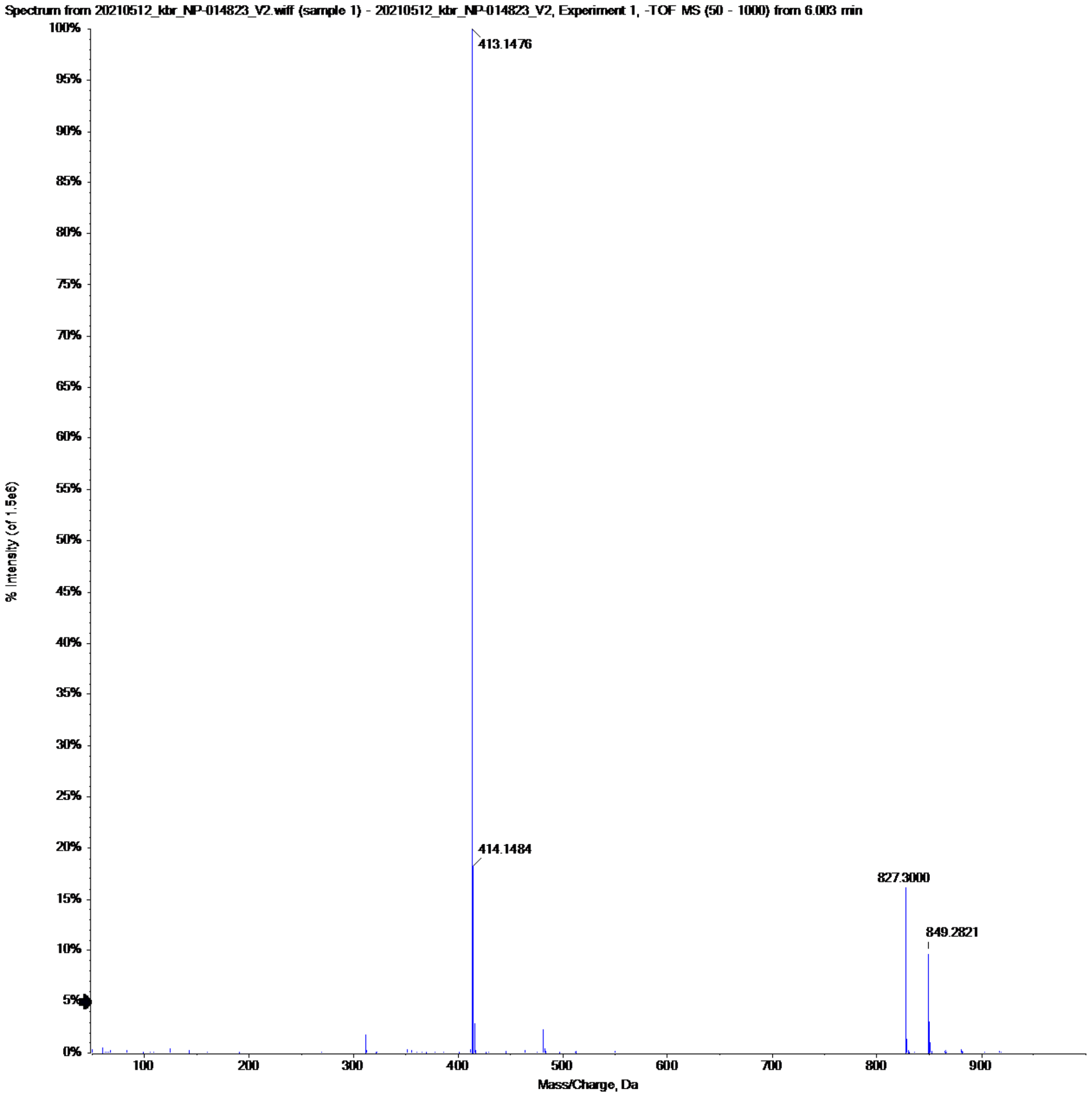

Figure S 94: qToF-MS spectrum (survey scan) of standard solution.

NP015777: HMG gluc F

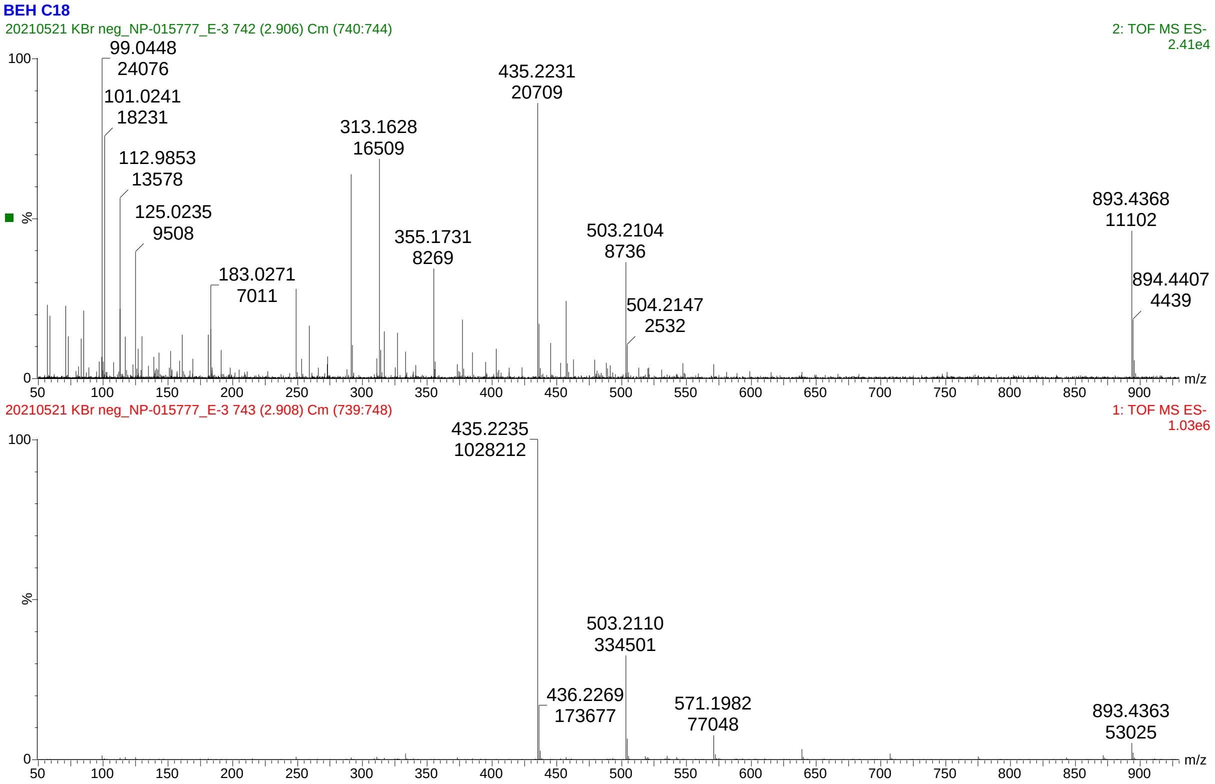

Figure S 95: ToF-MS^E spectra of HMG gluc F standard solution with high CE (top) and low CE (bottom).

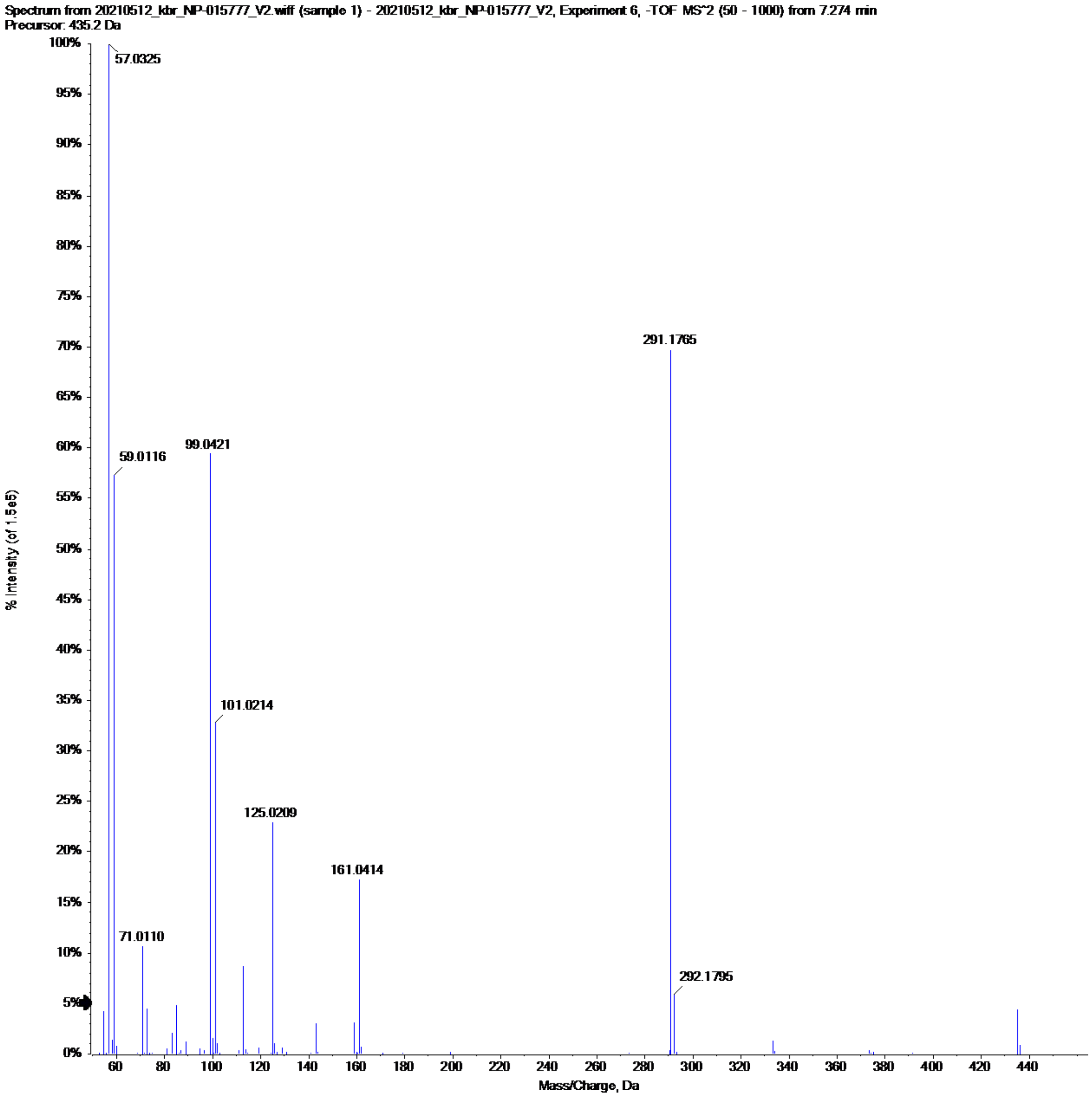

Figure S 96: qToF-MS/MS spectrum of standard solution.

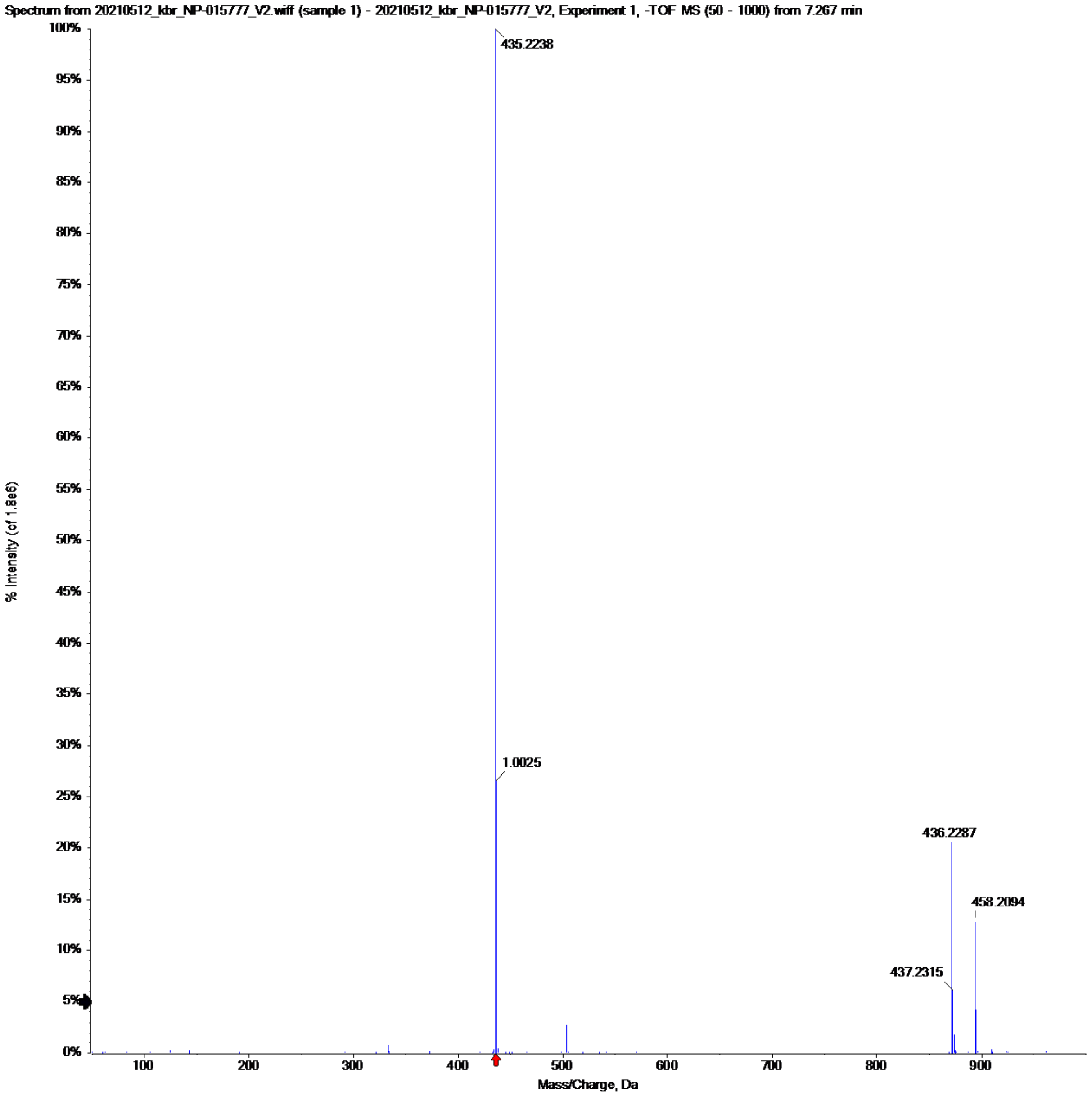

Figure S 97: qToF-MS spectrum (survey scan) of standard solution.

NP018595: HMG gluc G

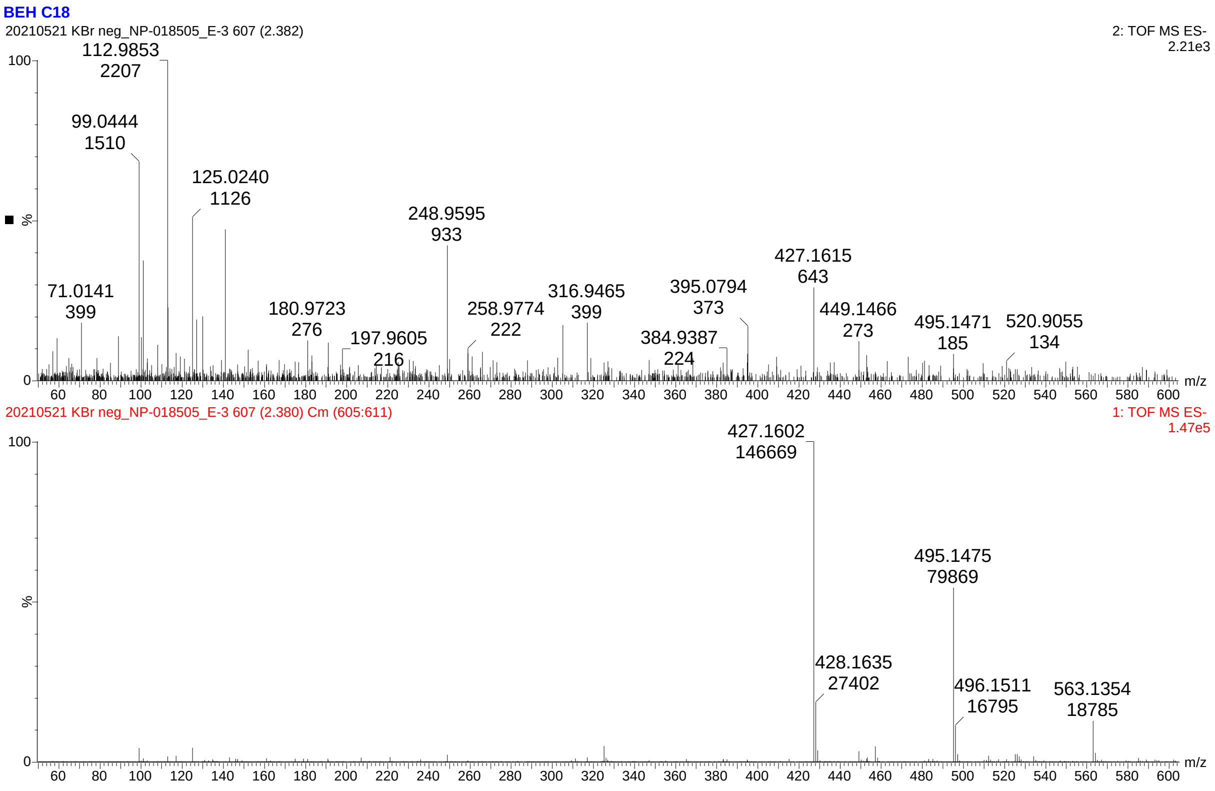

Figure S 98: ToF-MS^E spectra of HMG gluc G standard solution with high CE (top) and low CE (bottom).

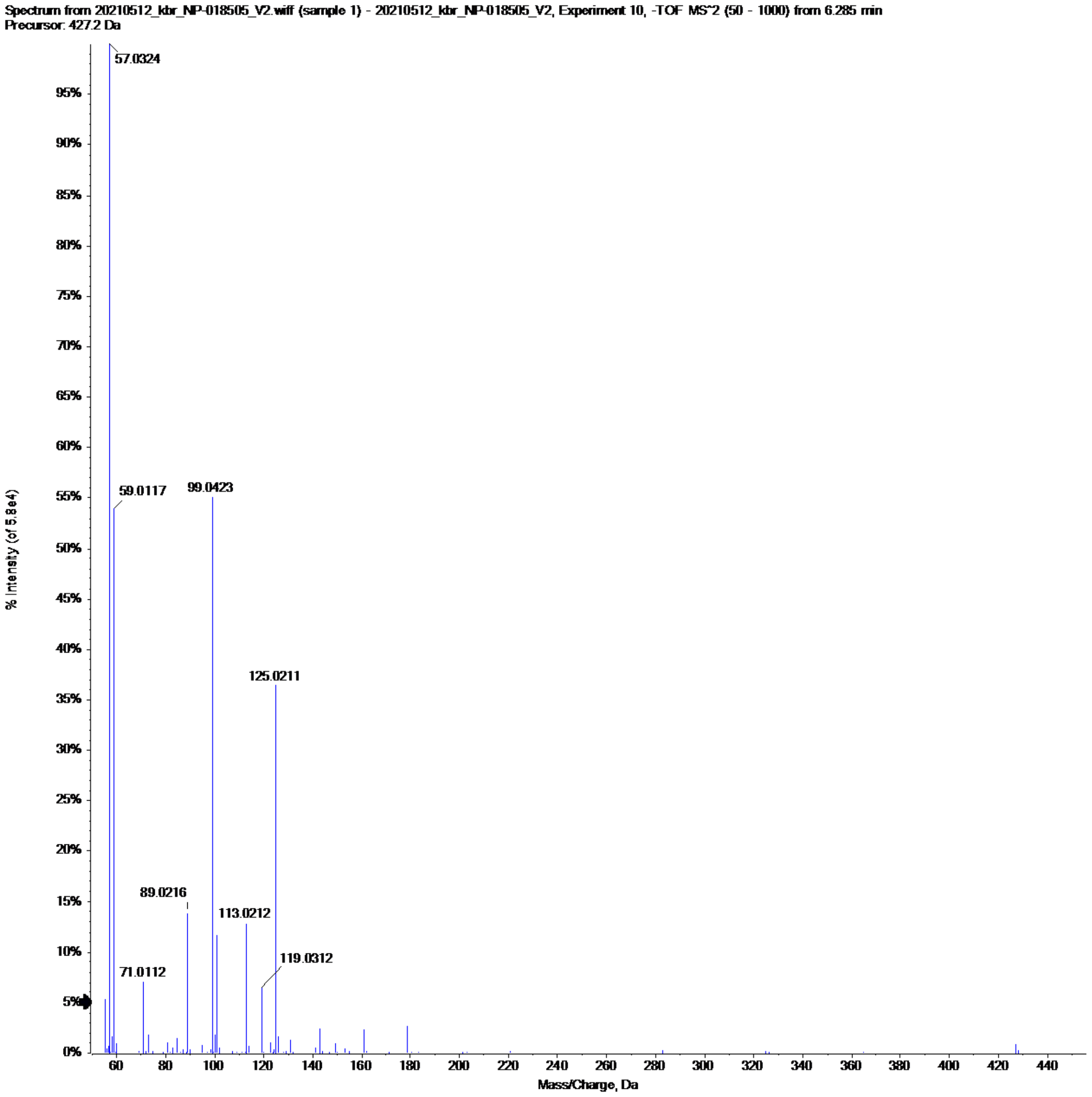

Figure S 99: qToF-MS/MS spectrum of standard solution.

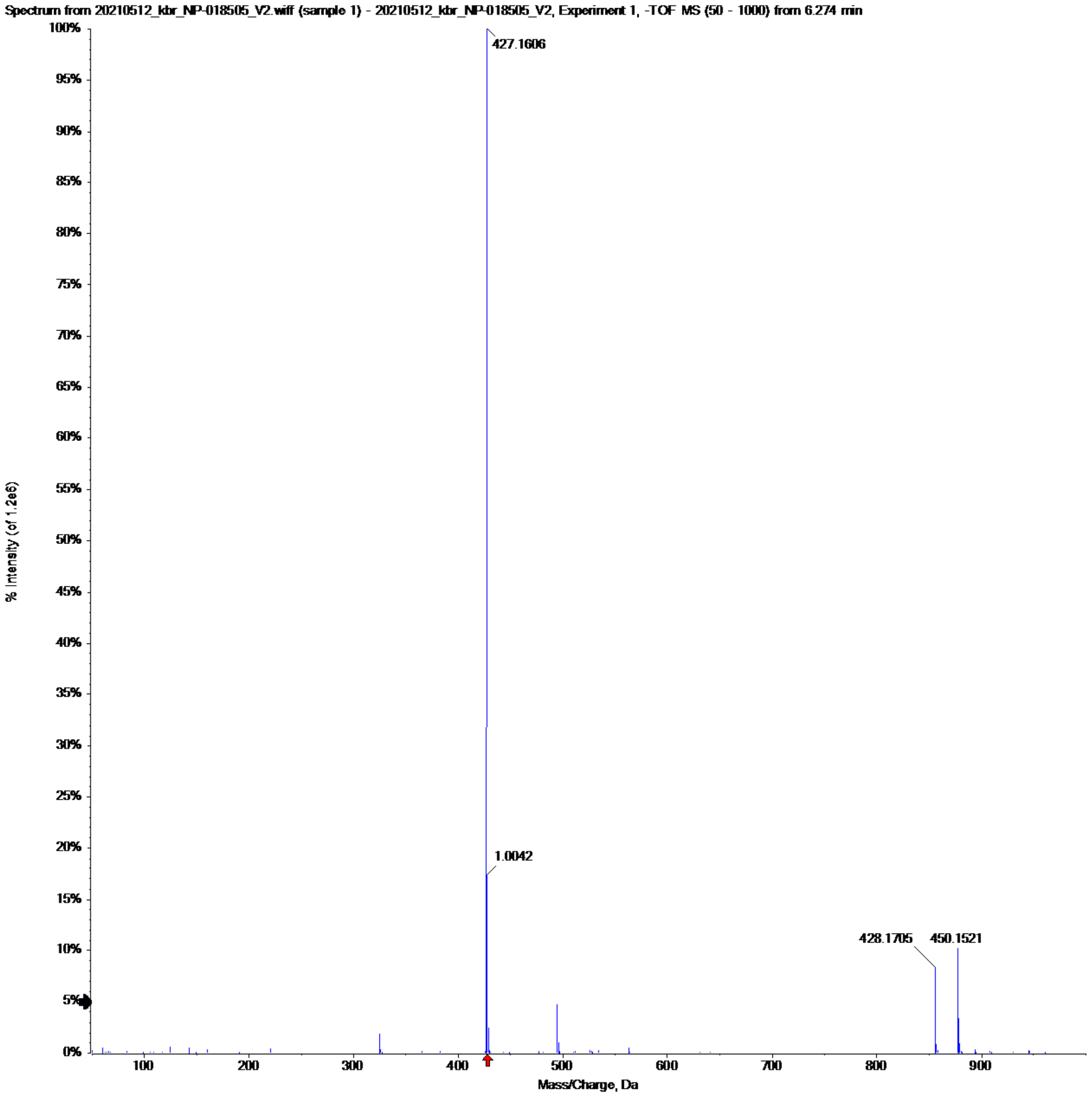

Figure S 100: qToF-MS spectrum (survey scan) of standard solution.

NP021228: HMG gluc H

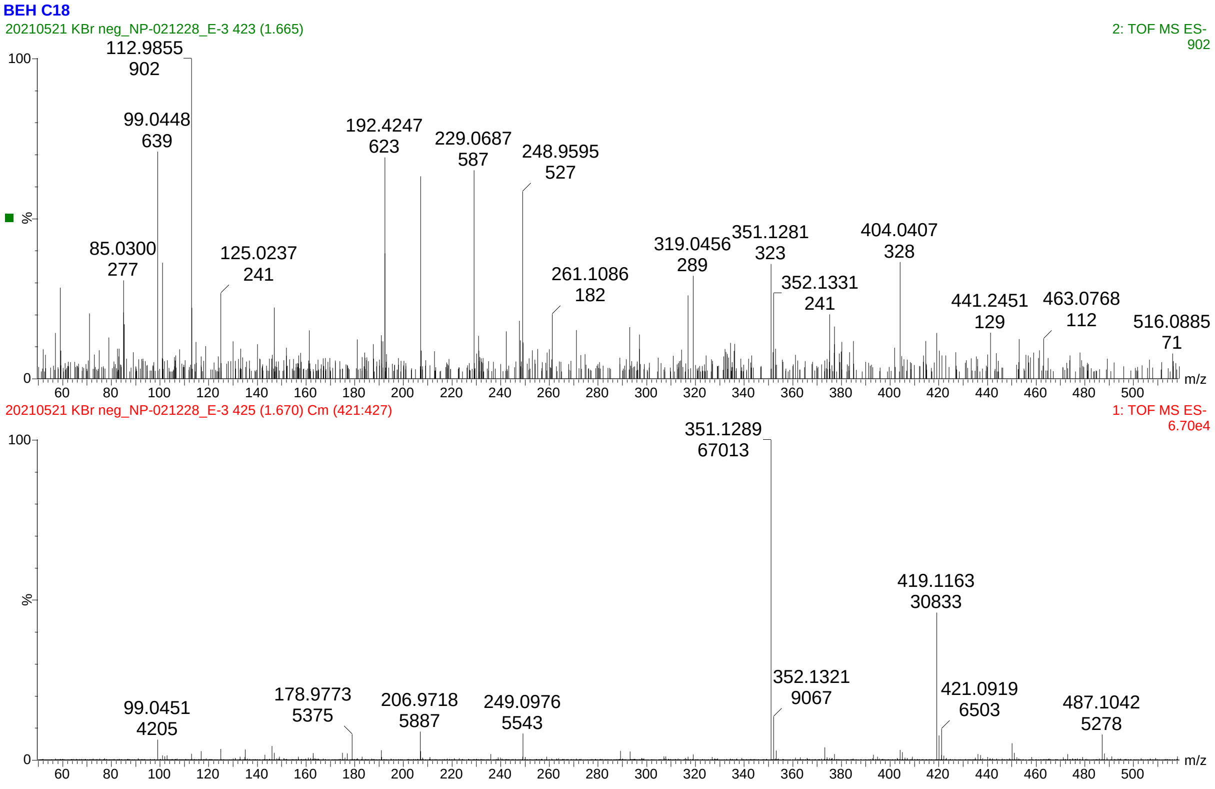

Figure S 101: ToF-MS^E spectra of HMG gluc H standard solution with high CE (top) and low CE (bottom).

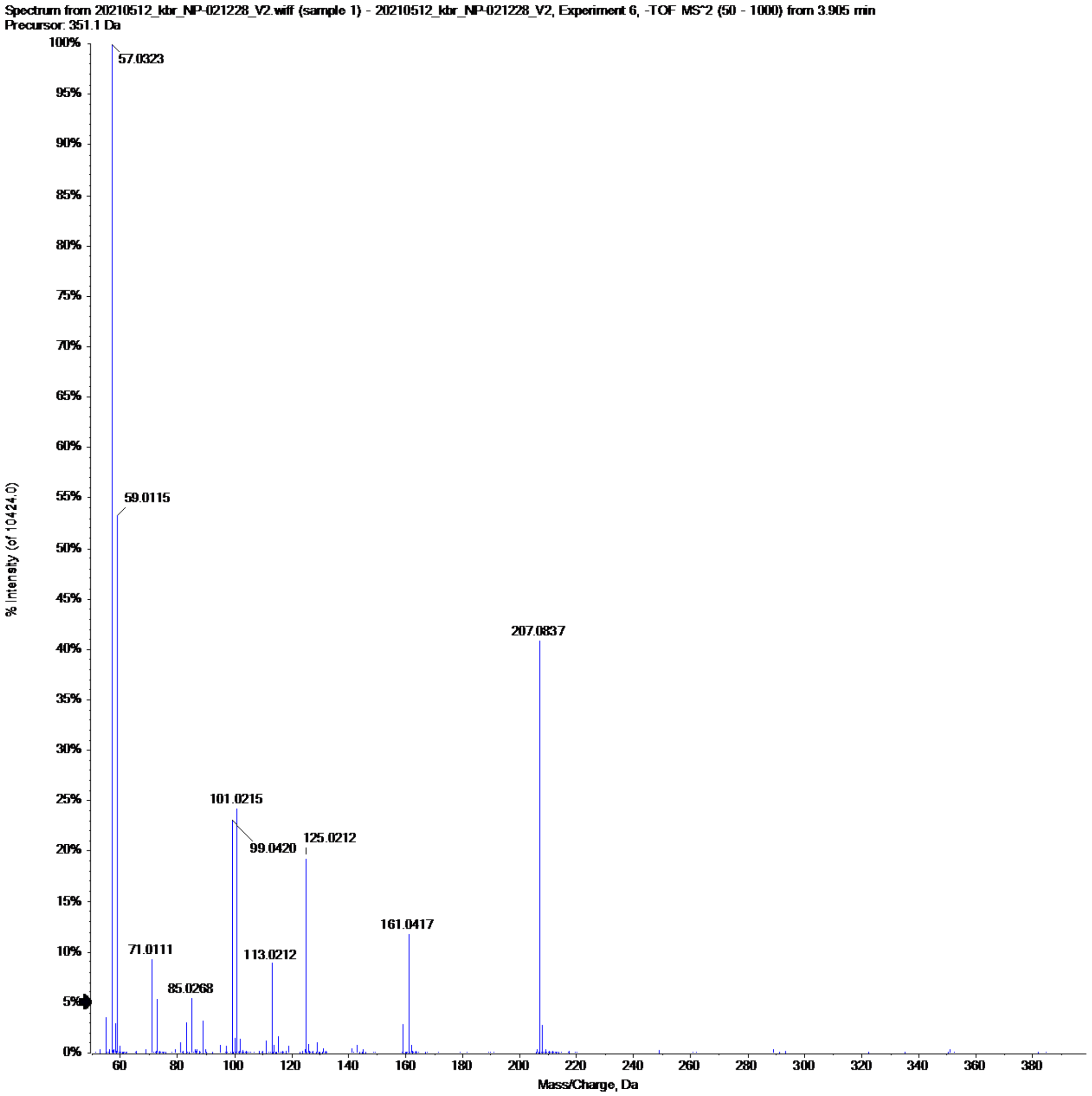

Figure S 102: qToF-MS/MS spectrum of standard solution.

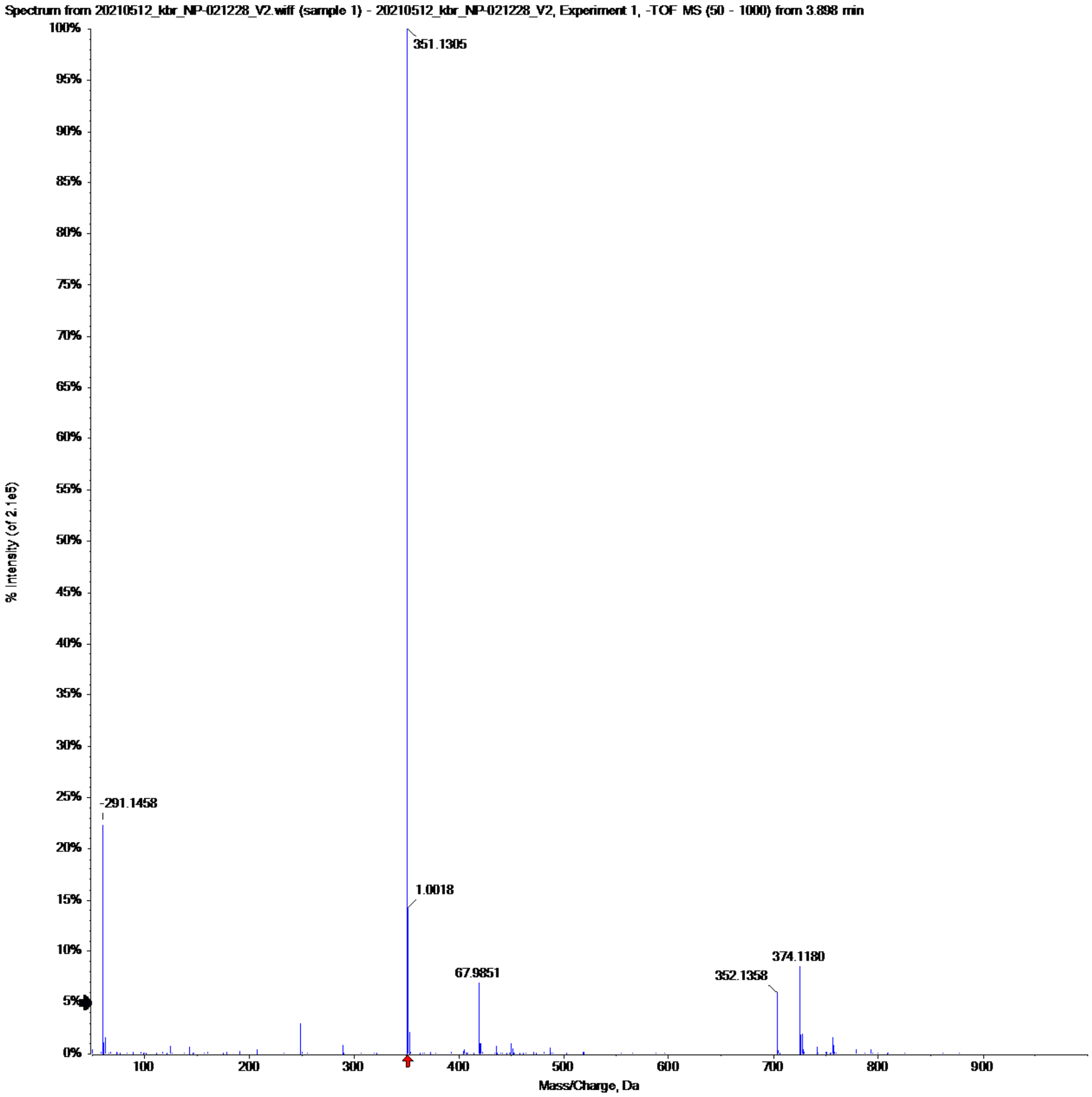

Figure S 103: qToF-MS spectrum (survey scan) of standard solution.

NP022515: HMG gluc I

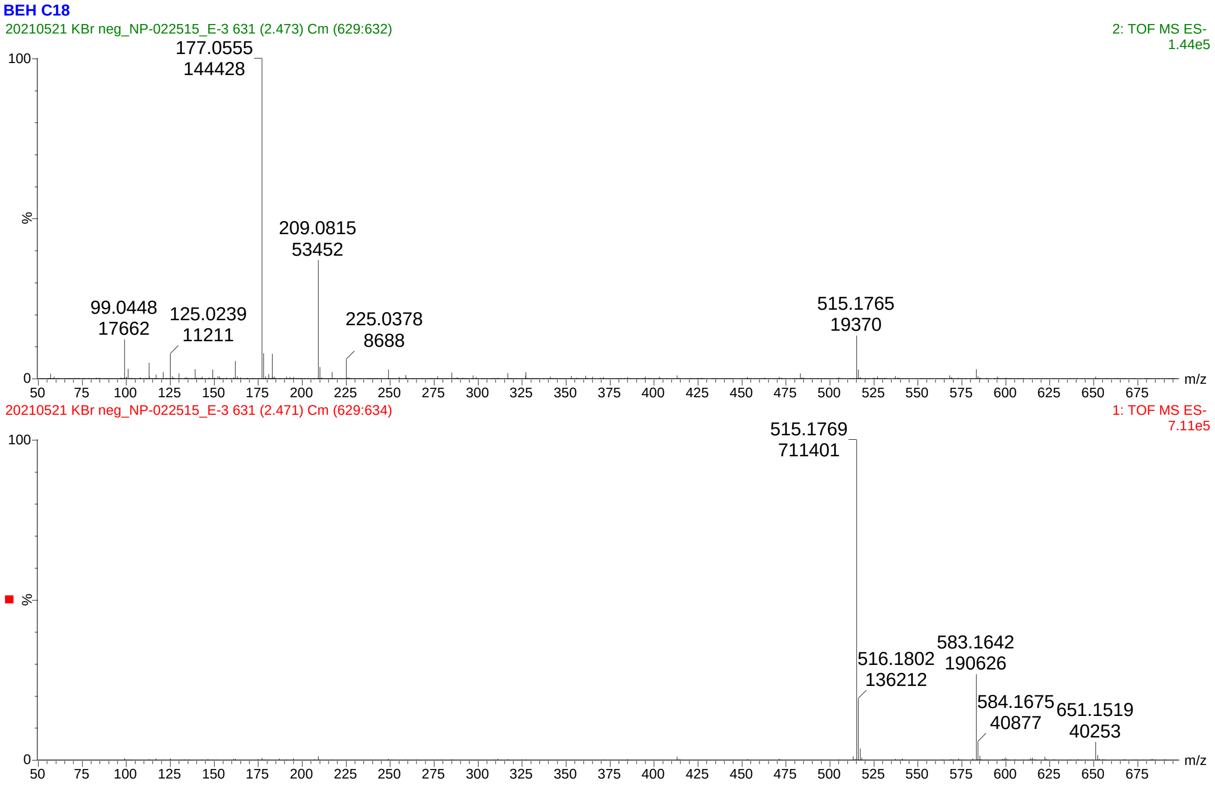

Figure S 104: ToF-MS^E spectra of HMG gluc I standard solution with high CE (top) and low CE (bottom).

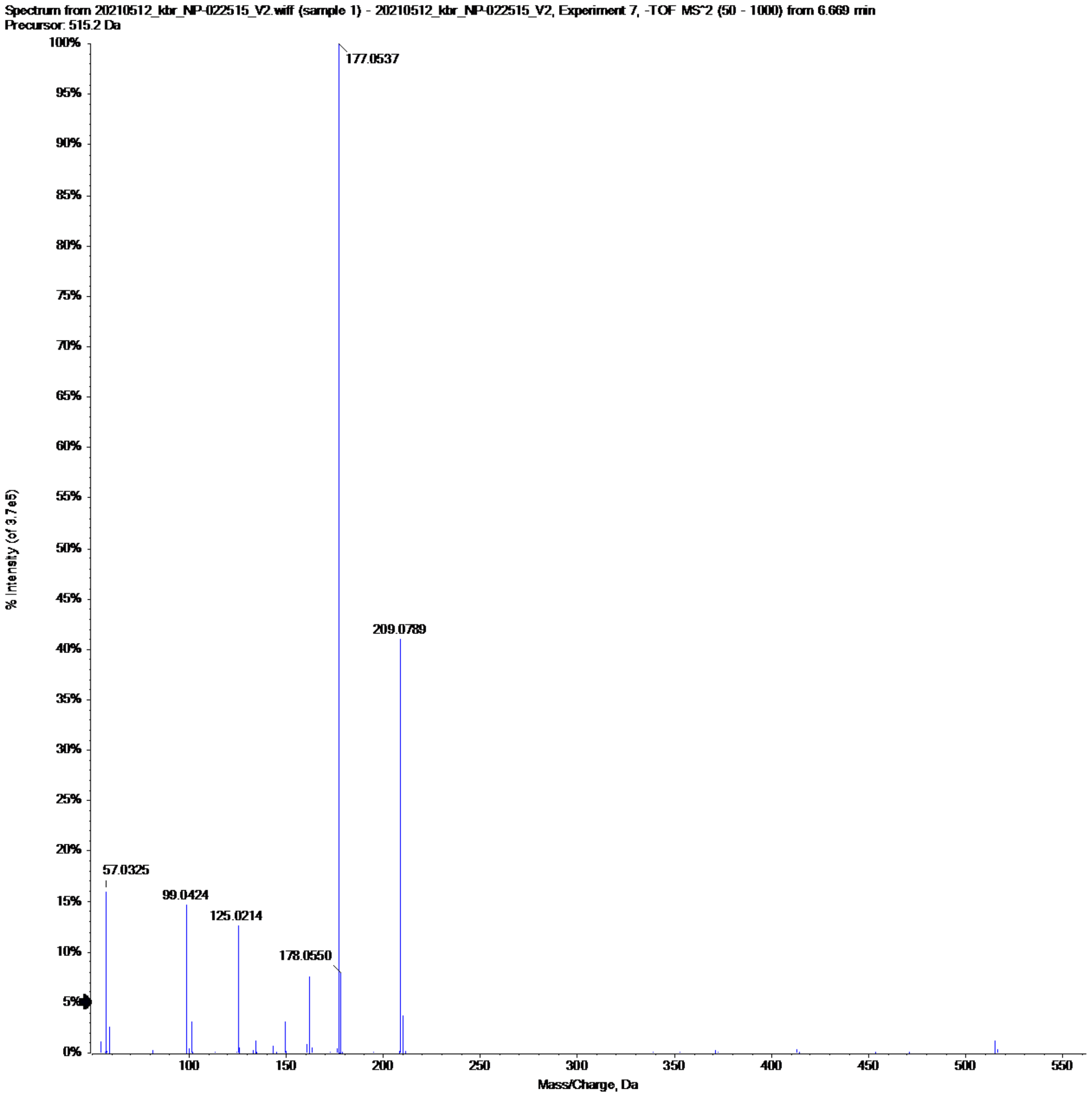

Figure S 105: qToF-MS/MS spectrum of standard solution.

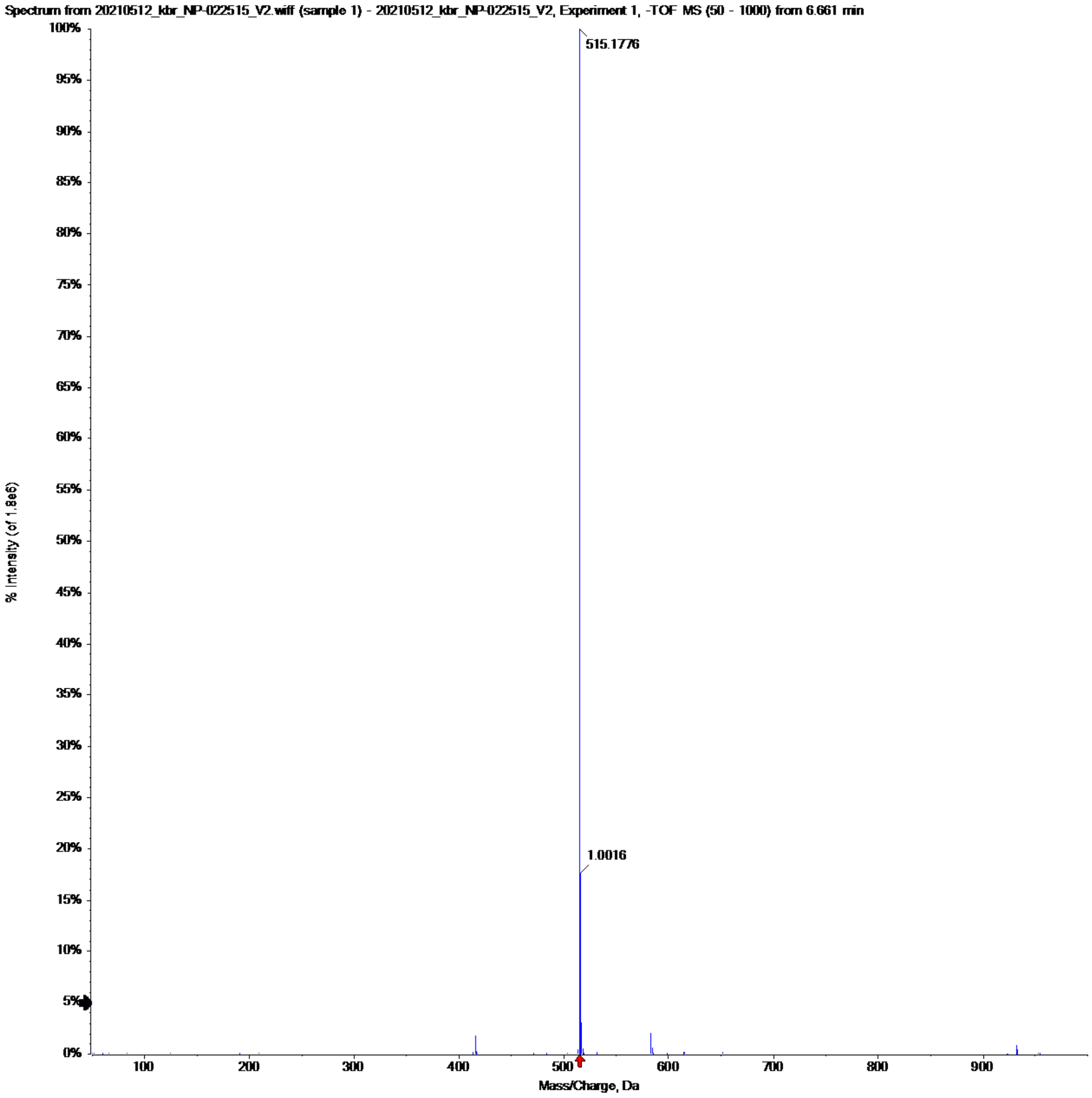

Figure S 106: qToF-MS spectrum (survey scan) of standard solution.

NP022828: HMG gluc J

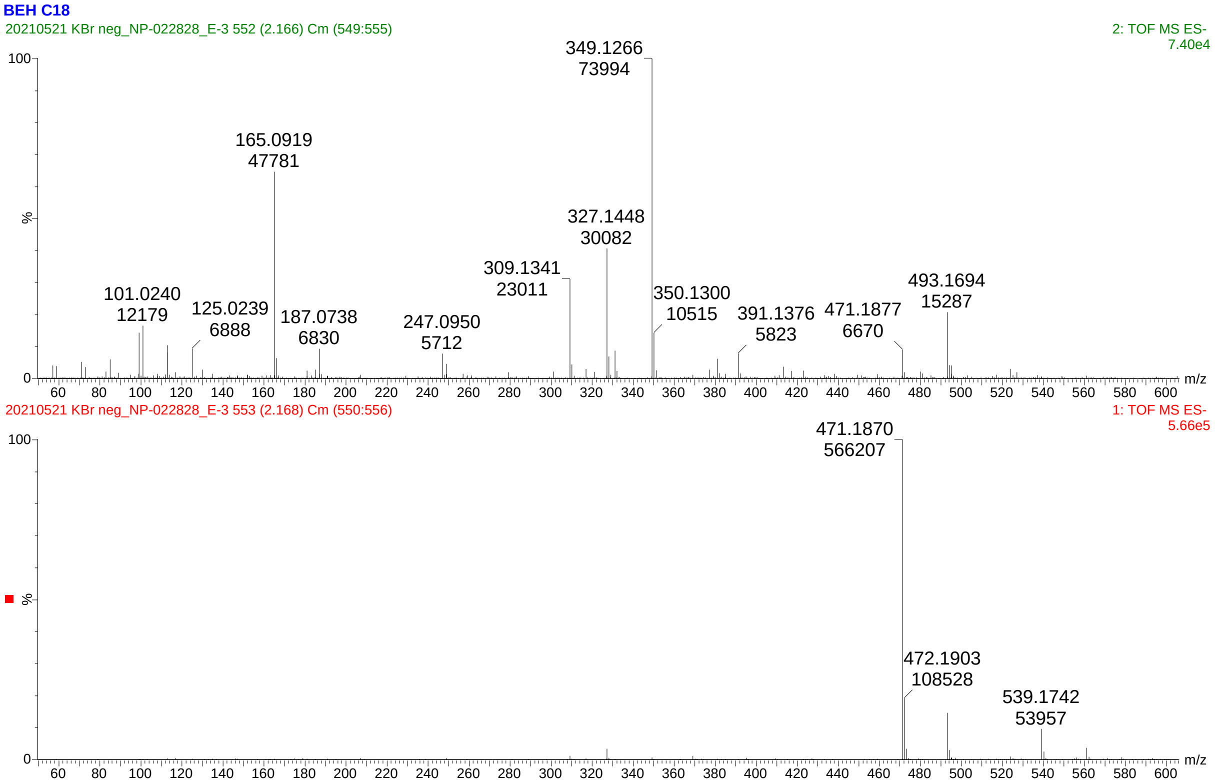

Figure S 107: ToF-MS^E spectra of HMG gluc J standard solution with high CE (top) and low CE (bottom).

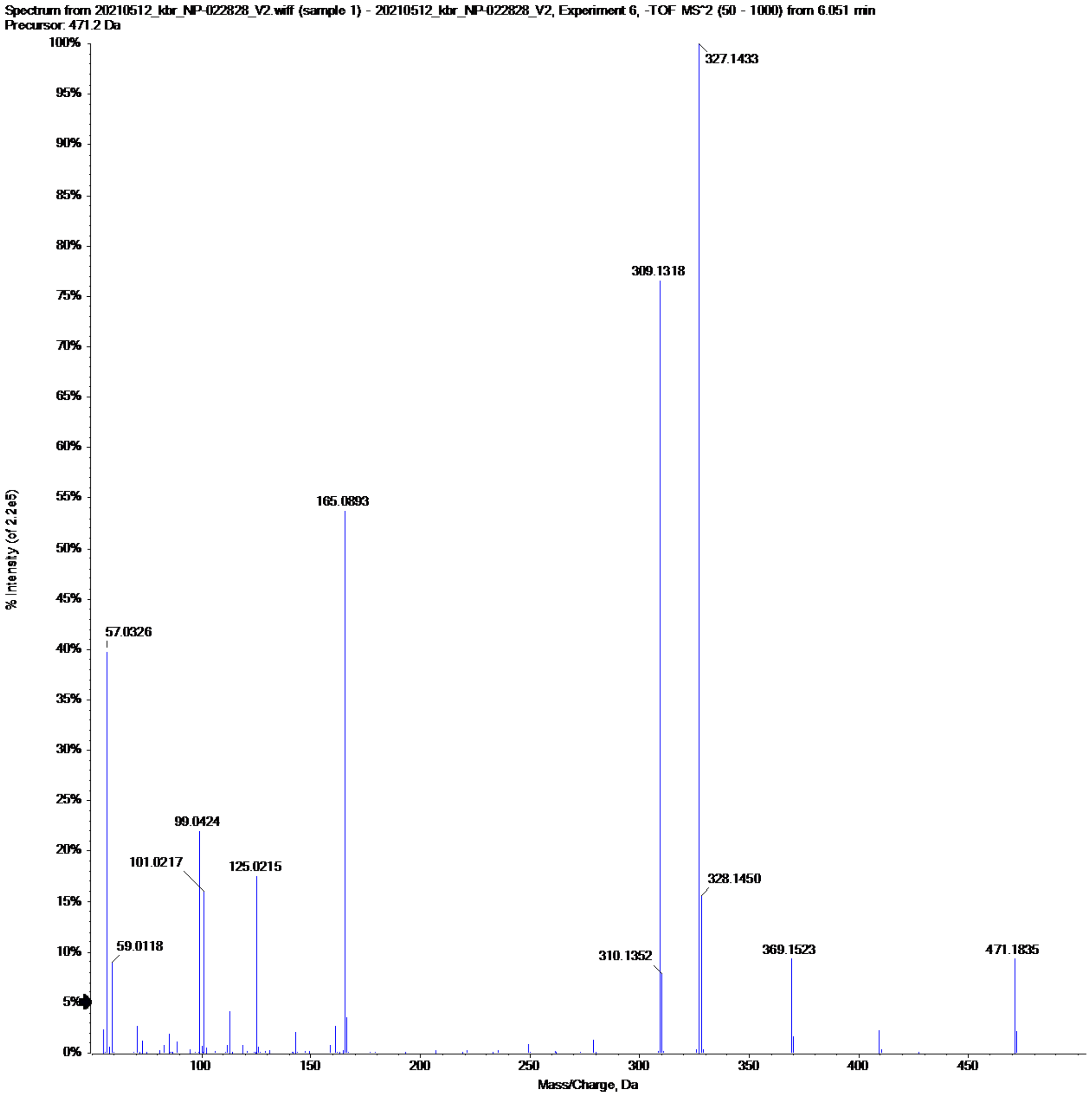

Figure S 108: qToF-MS/MS spectrum of standard solution.

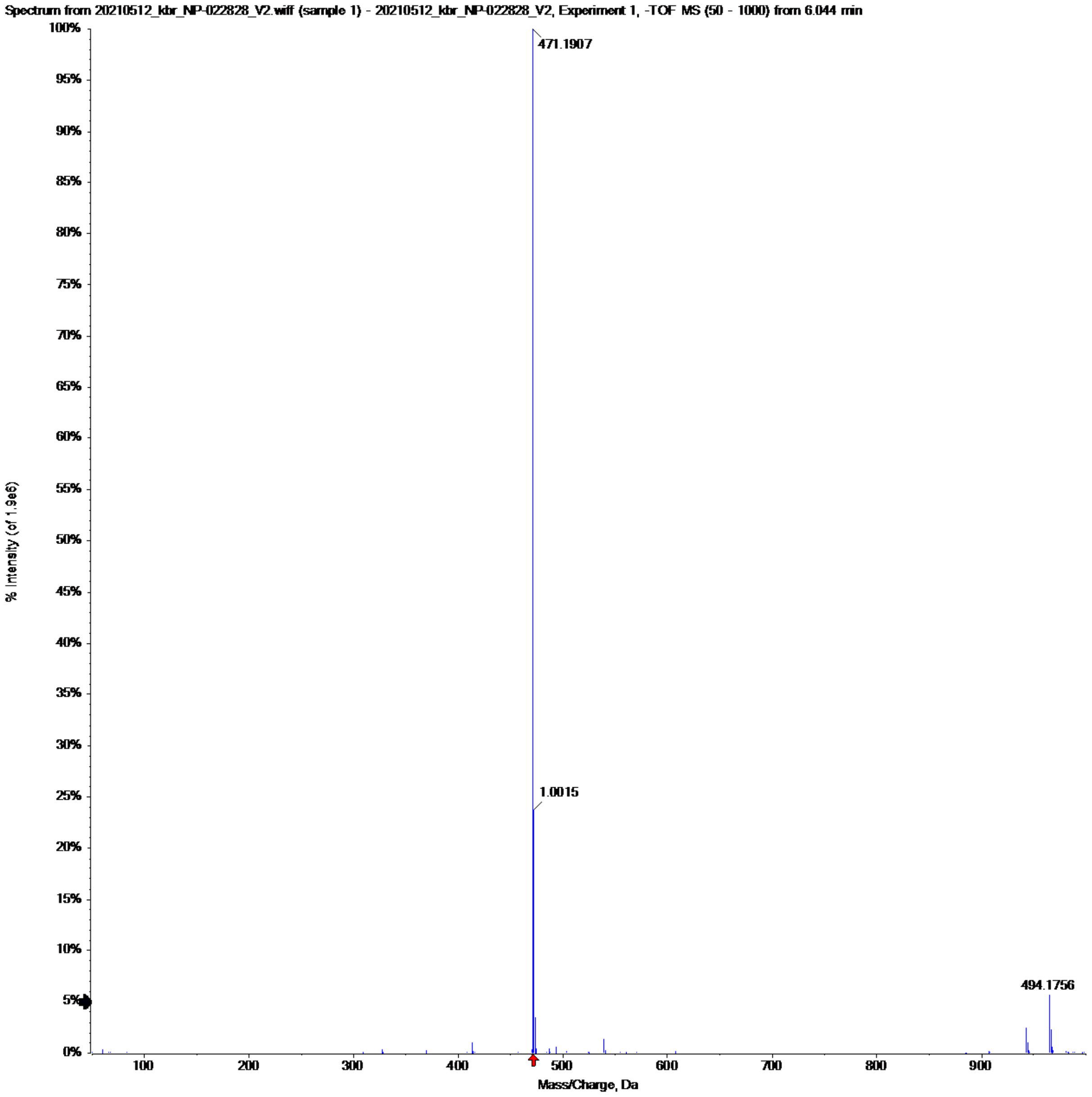

Figure S 109: qToF-MS spectrum (survey scan) of standard solution.

NP023425: HMG gluc K

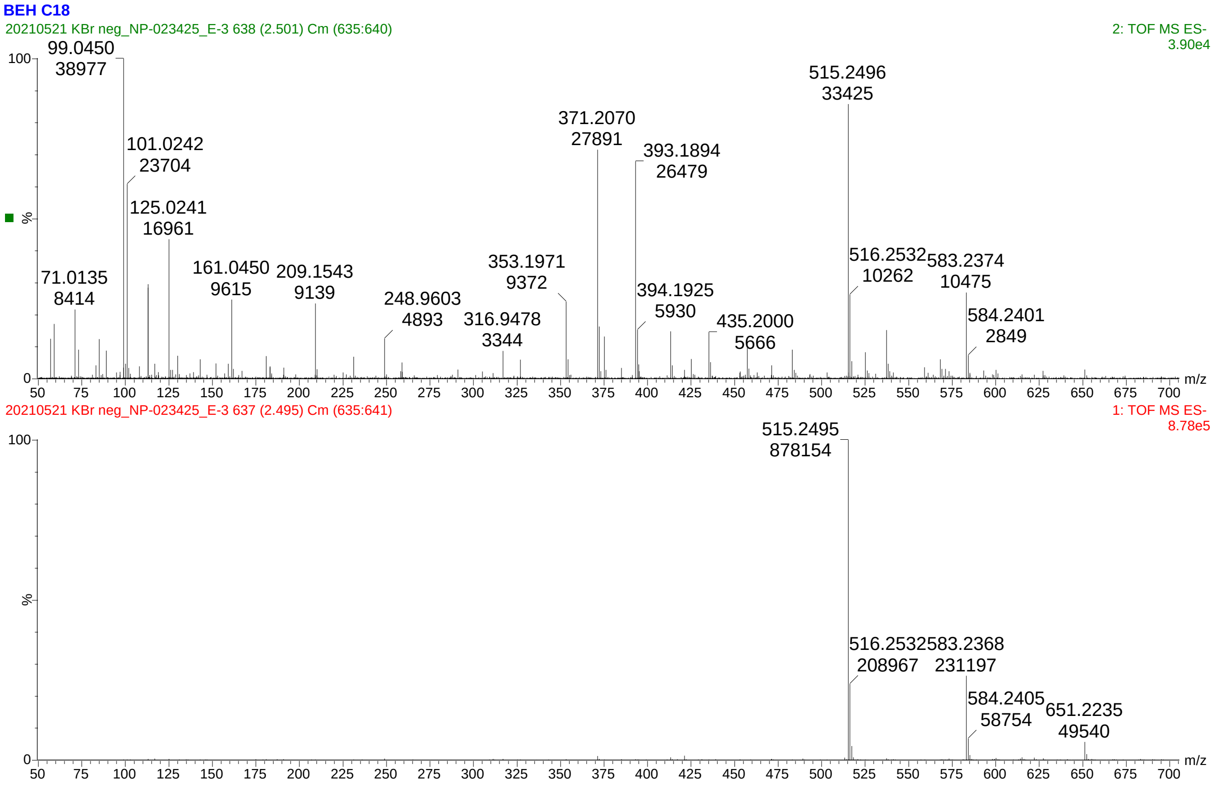

Figure S 110: ToF-MS^E spectra of HMG gluc K standard solution with high CE (top) and low CE (bottom).

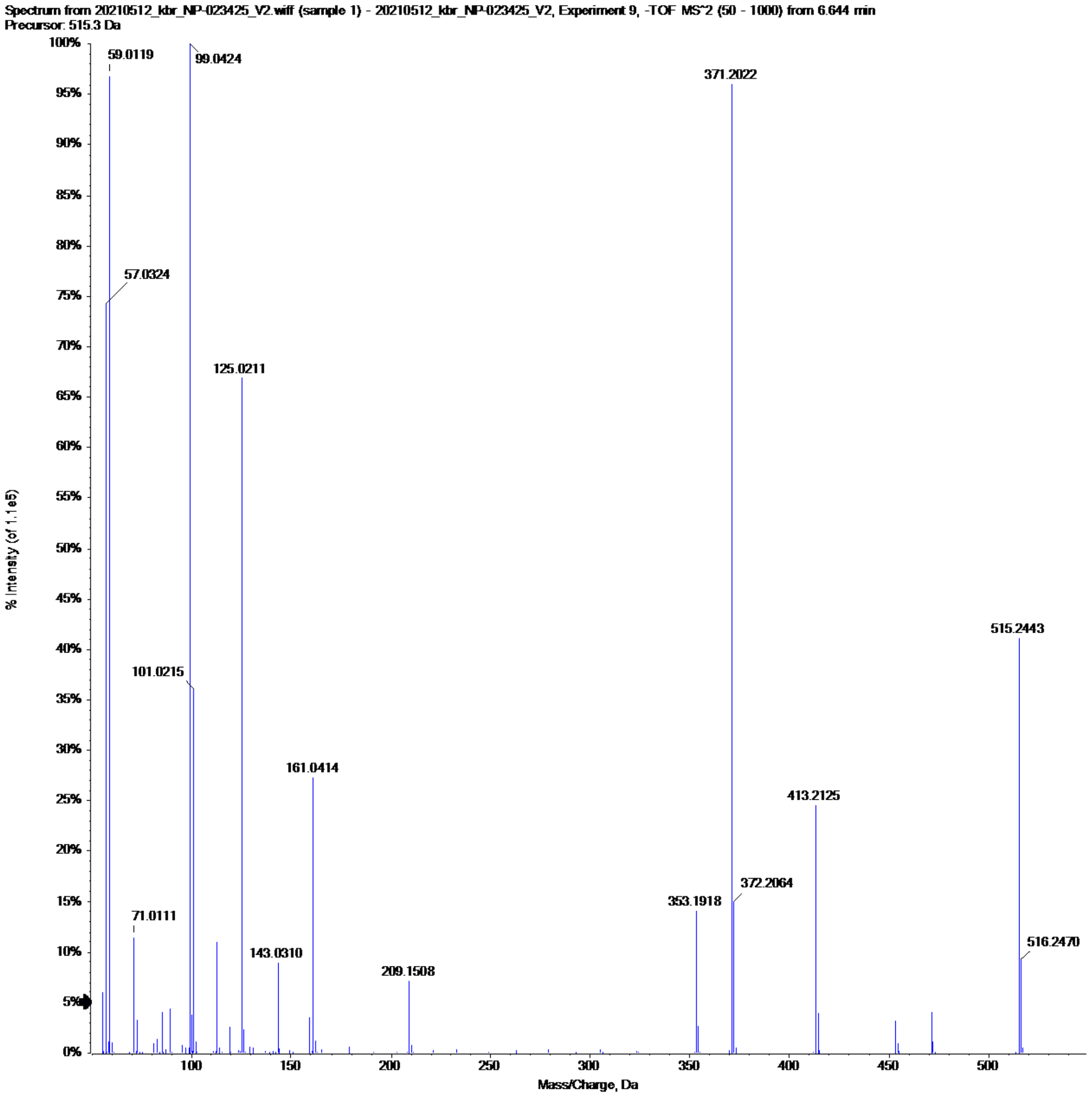

Figure S 111: qToF-MS/MS spectrum of standard solution.

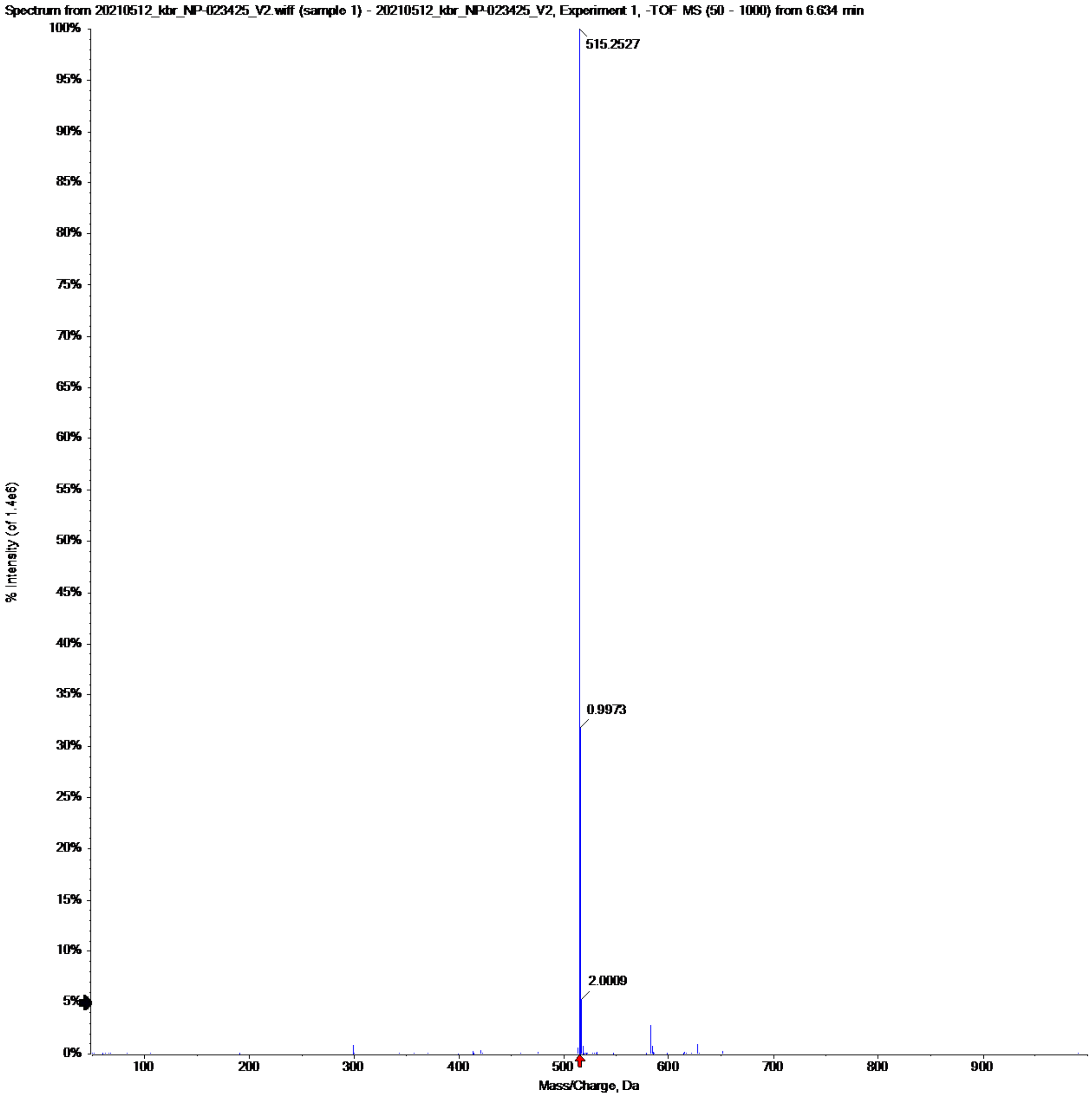

Figure S 112: qToF-MS spectrum (survey scan) of standard solution.

NP023820: HMG gluc L

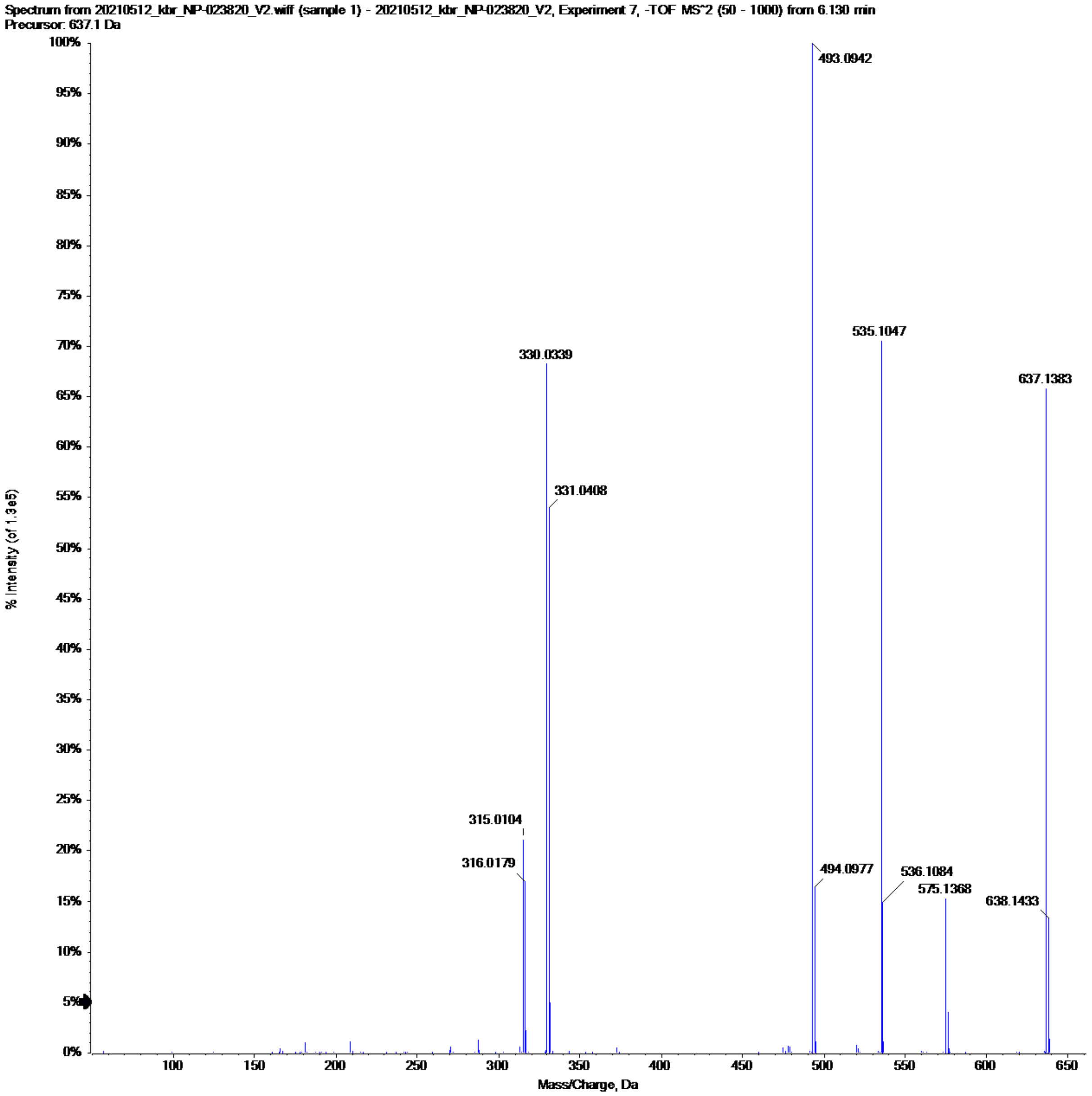

Figure S 113: qToF-MS/MS spectrum of standard solution.

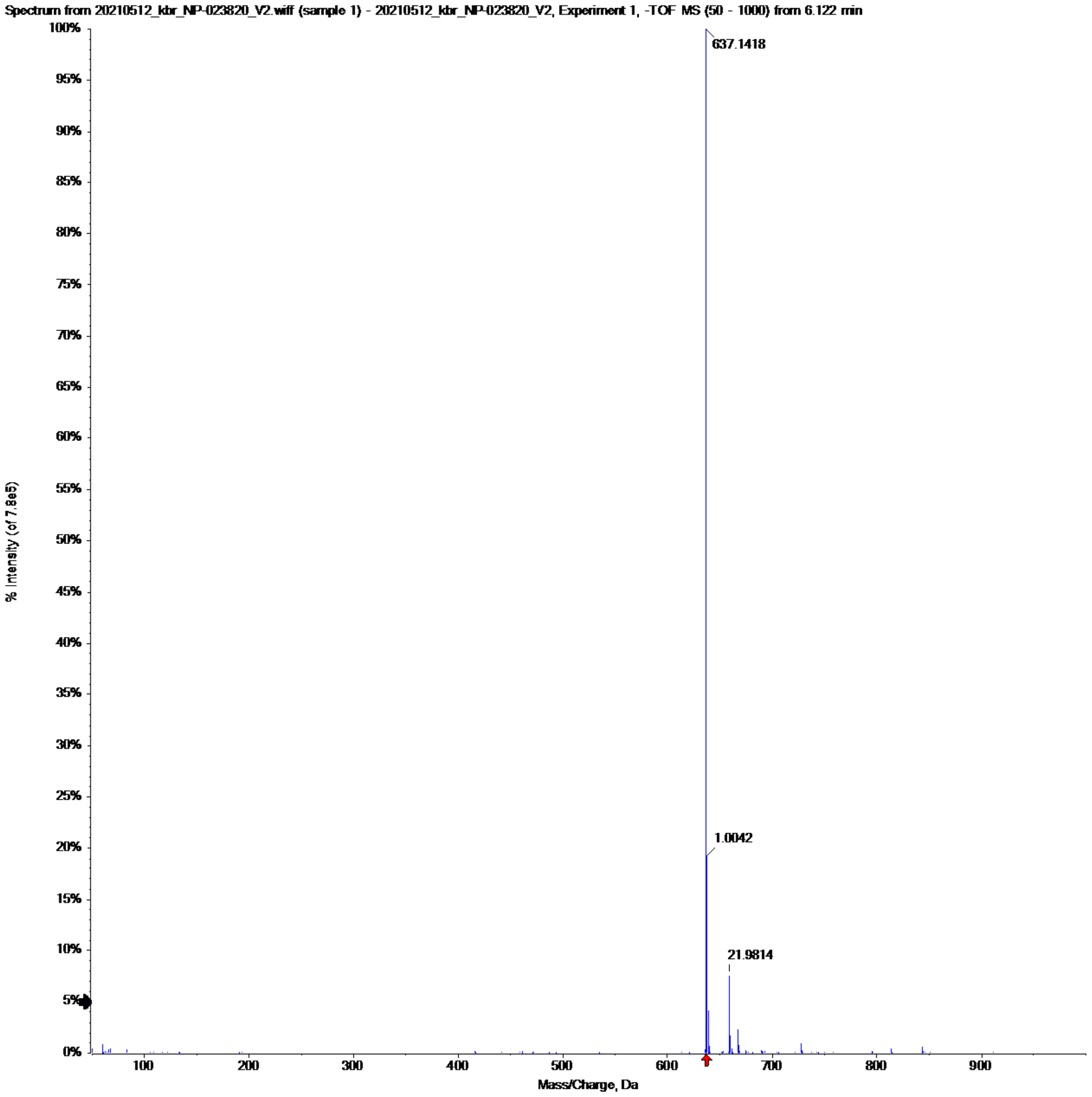

Figure S 114: qToF-MS spectrum (survey scan) of standard solution.

NP023821: HMG gluc M

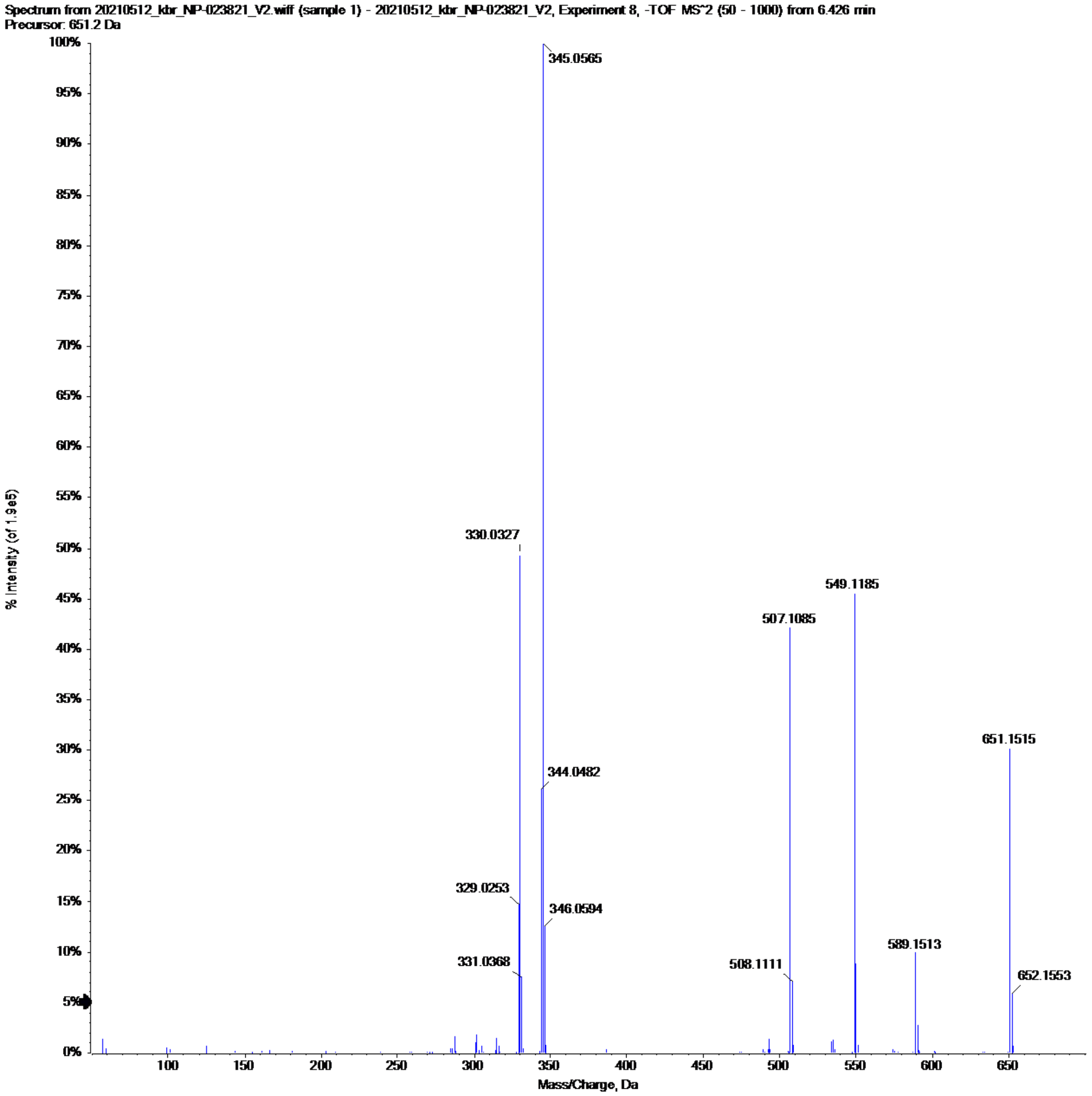

Figure S 115: qToF-MS/MS spectrum of standard solution.

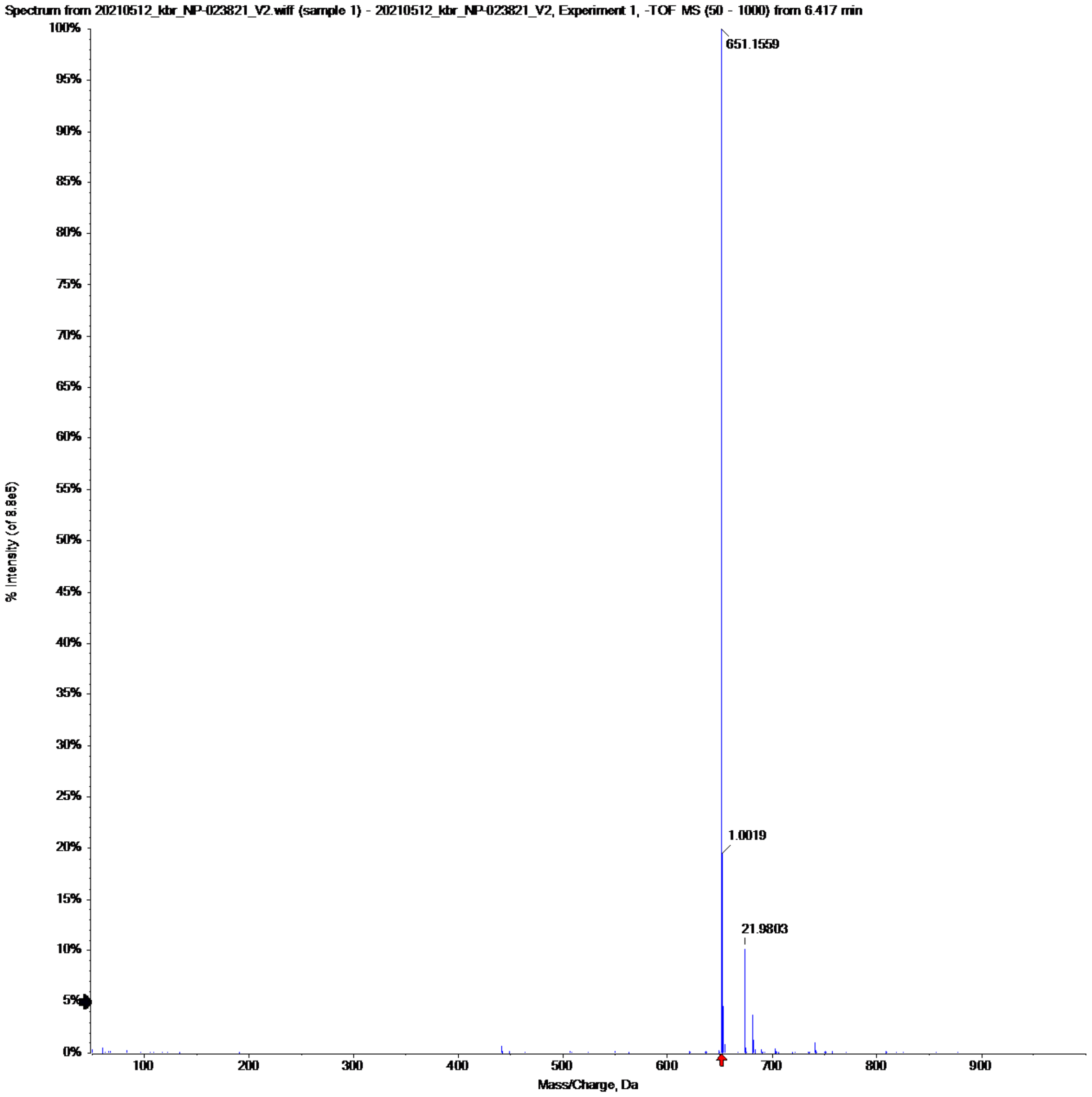

Figure S 116: qToF-MS spectrum (survey scan) of standard solution.

NP024048: HMG gluc N

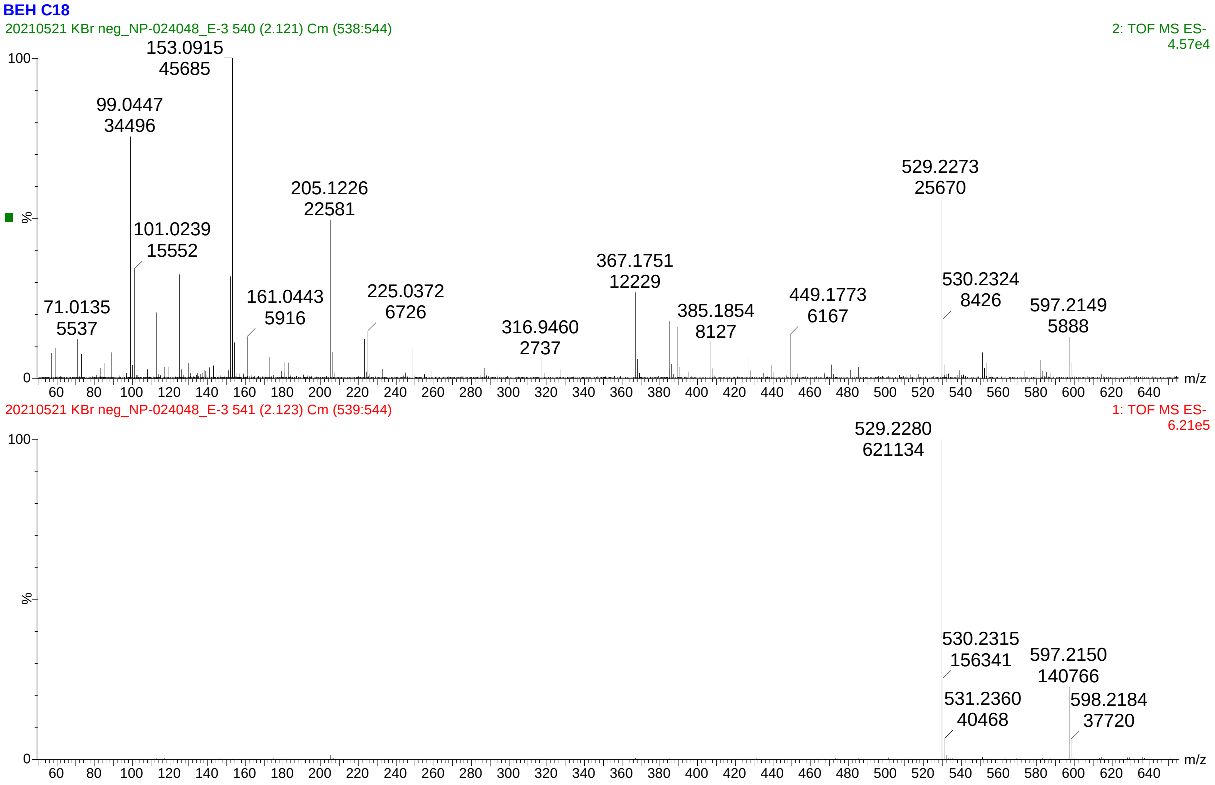

Figure S 117: ToF-MS^E spectra of HMG gluc N standard solution with high CE (top) and low CE (bottom).

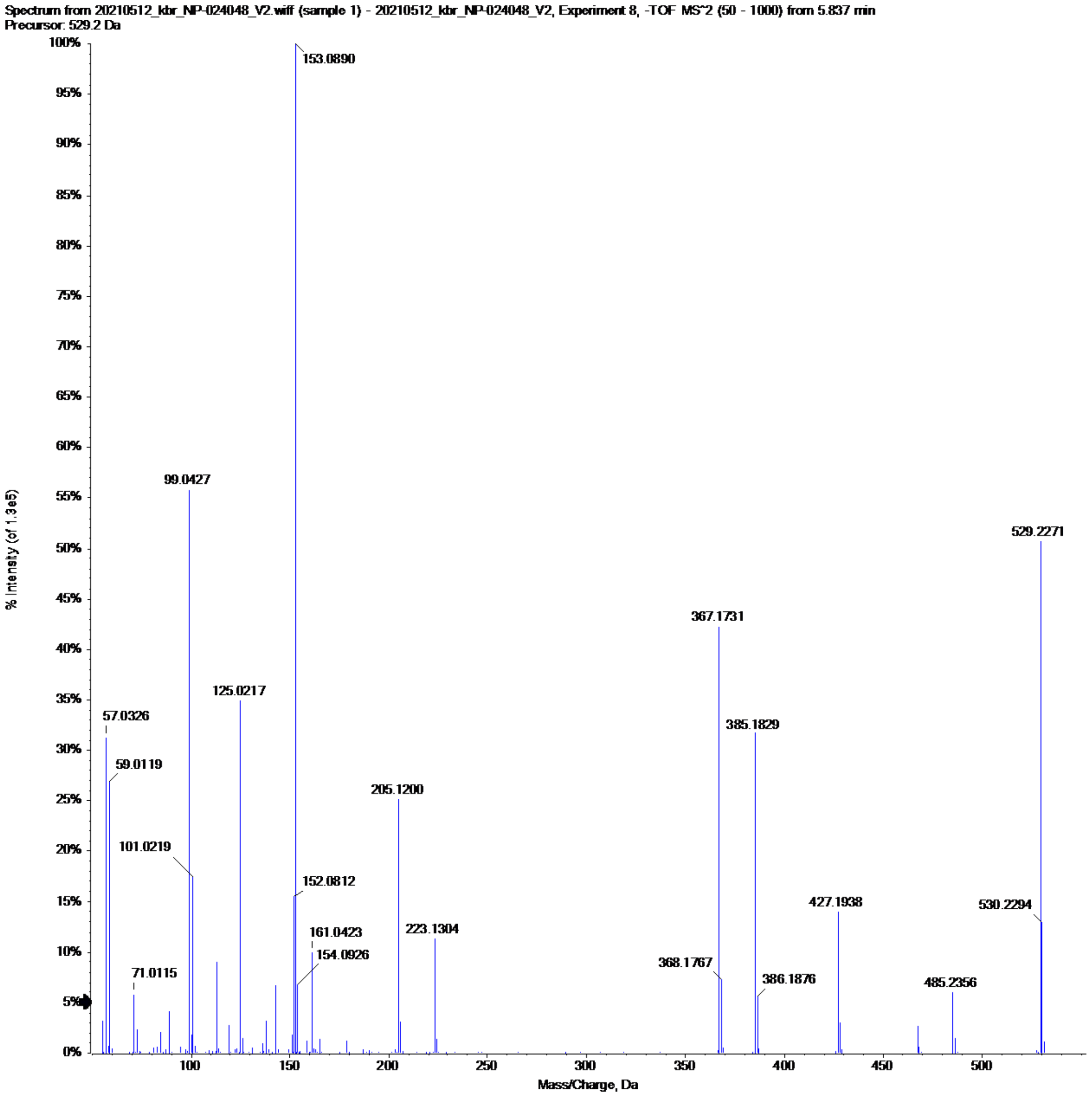

Figure S 118: qToF-MS/MS spectrum of standard solution.

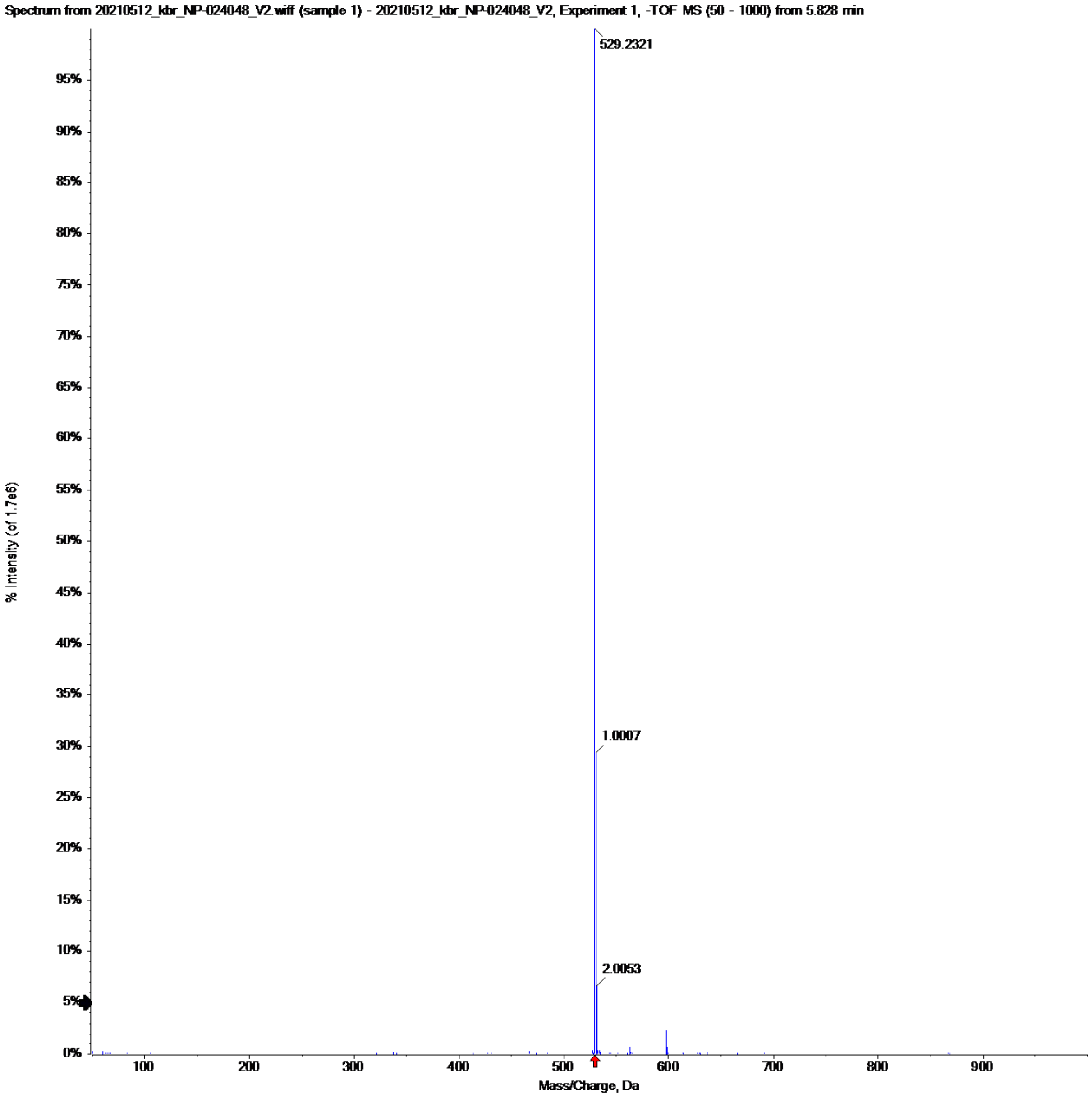

Figure S 119: qToF-MS spectrum (survey scan) of standard solution.

NP023236: HMG gluc O

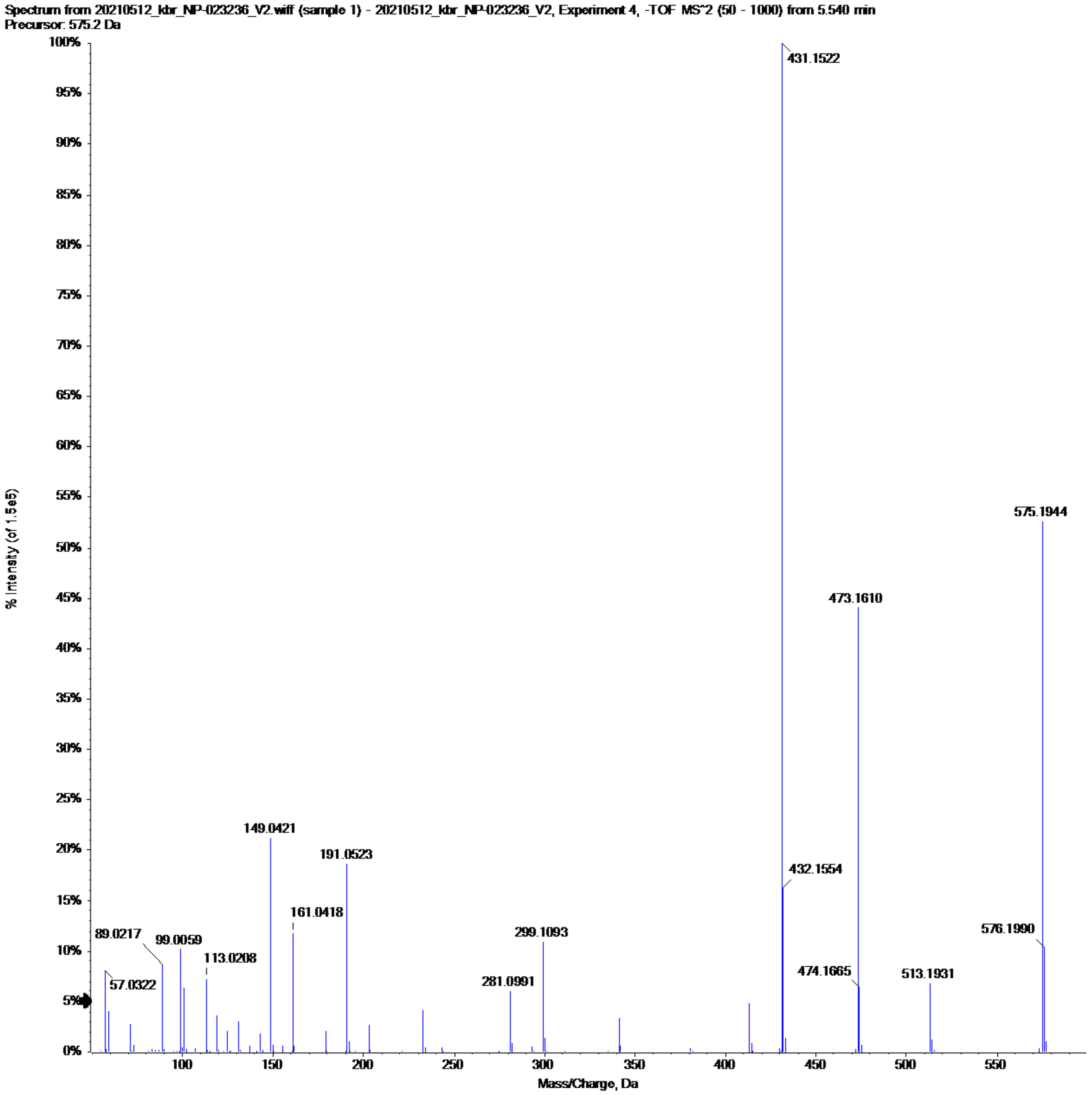

Figure S 120: qToF-MS/MS spectrum of standard solution.

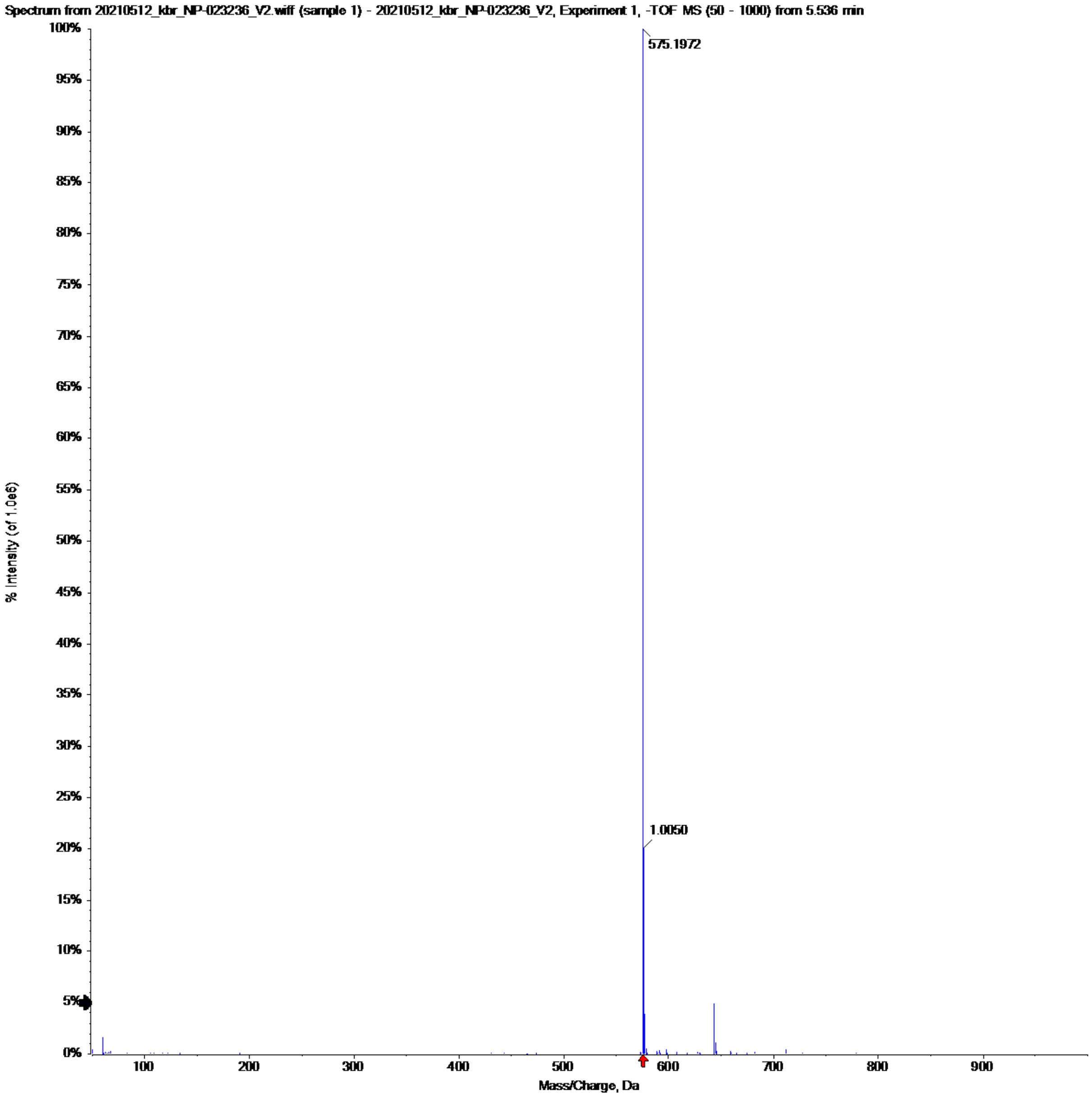

Figure S 121: qToF-MS spectrum (survey scan) of standard solution.

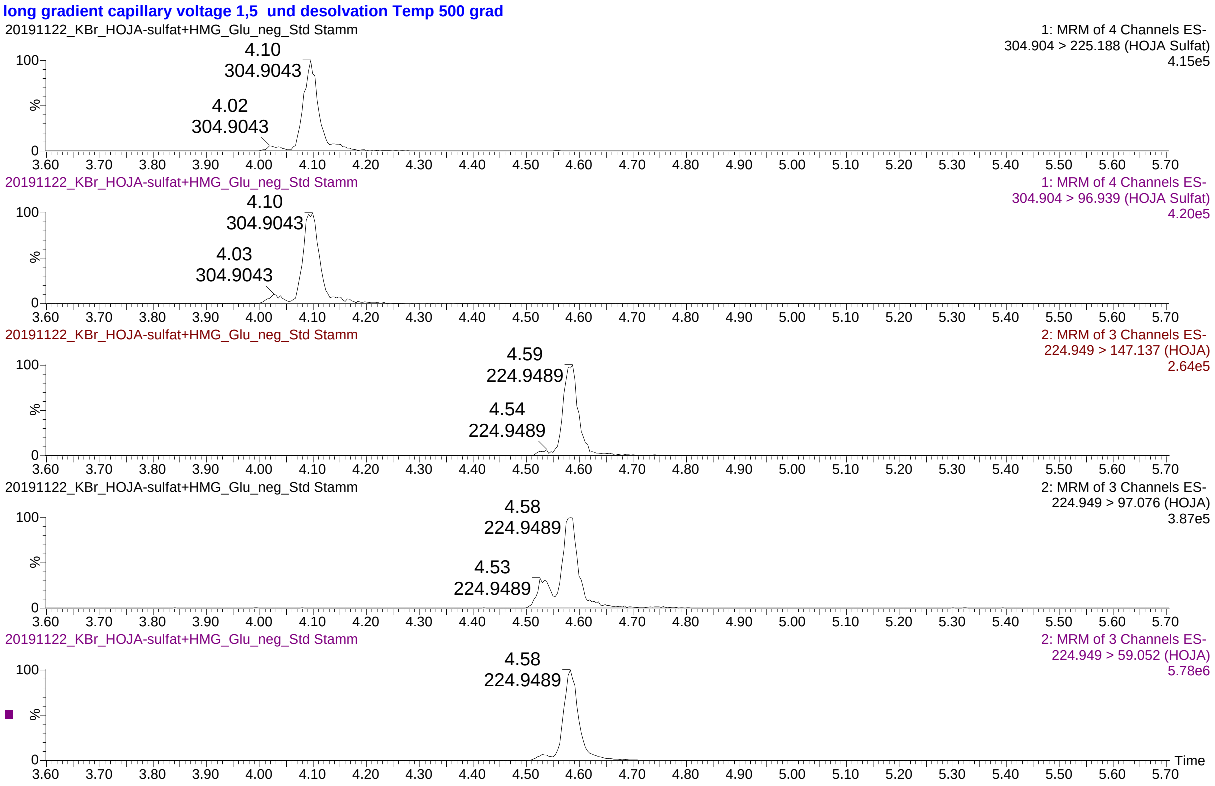

Figure S 122: MRM traces of UPLC-MS/MS of HOJA sulfate (1st and 2nd from top) and of HOJA (3rd, 4th and 5th from top) of standard solution.

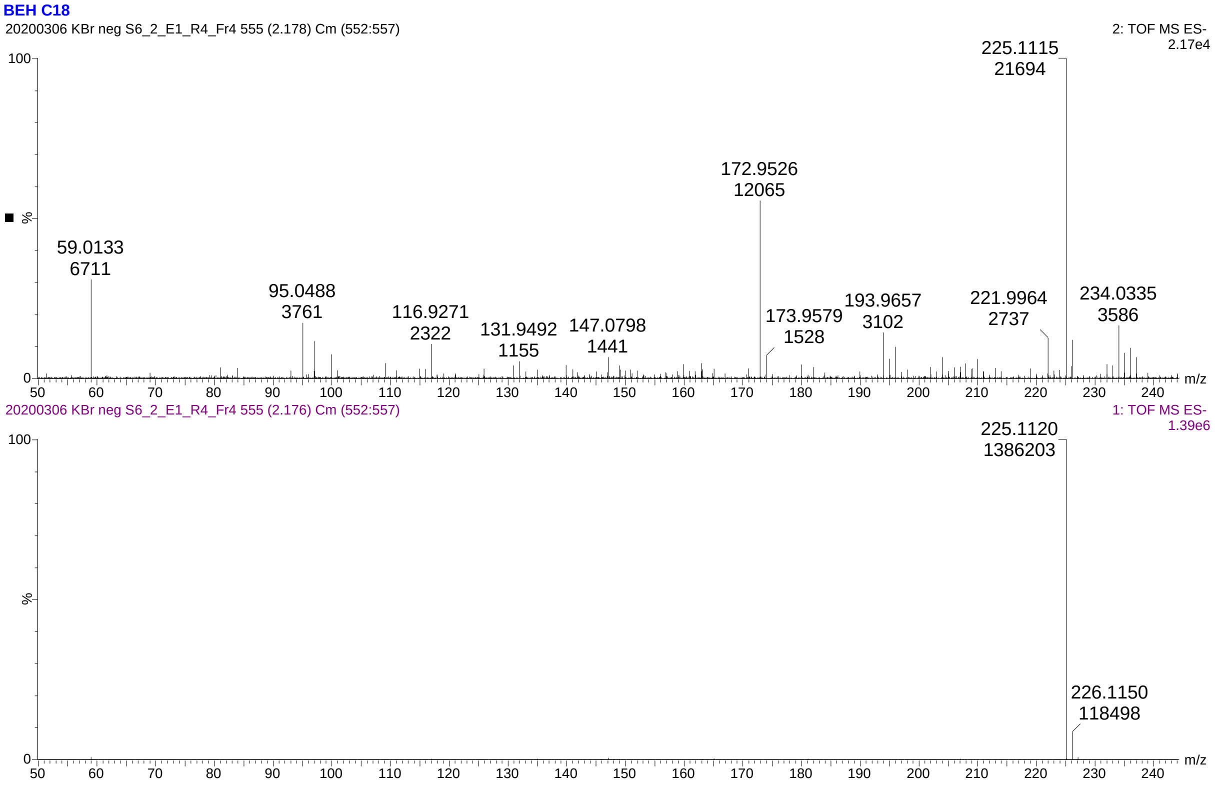

Figure S 123: ToF-MS^E spectra of HOJA standard solution with high CE (top) and low CE (bottom).

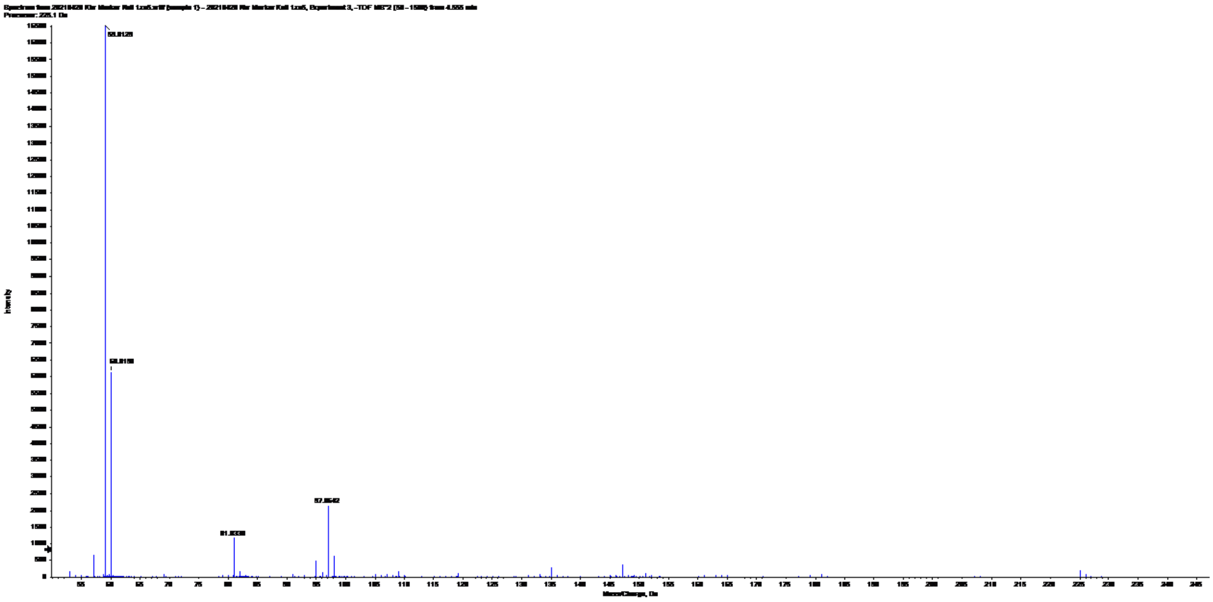

Figure S 124: qToF-MS/MS spectrum of standard solution.

(+)-catechin

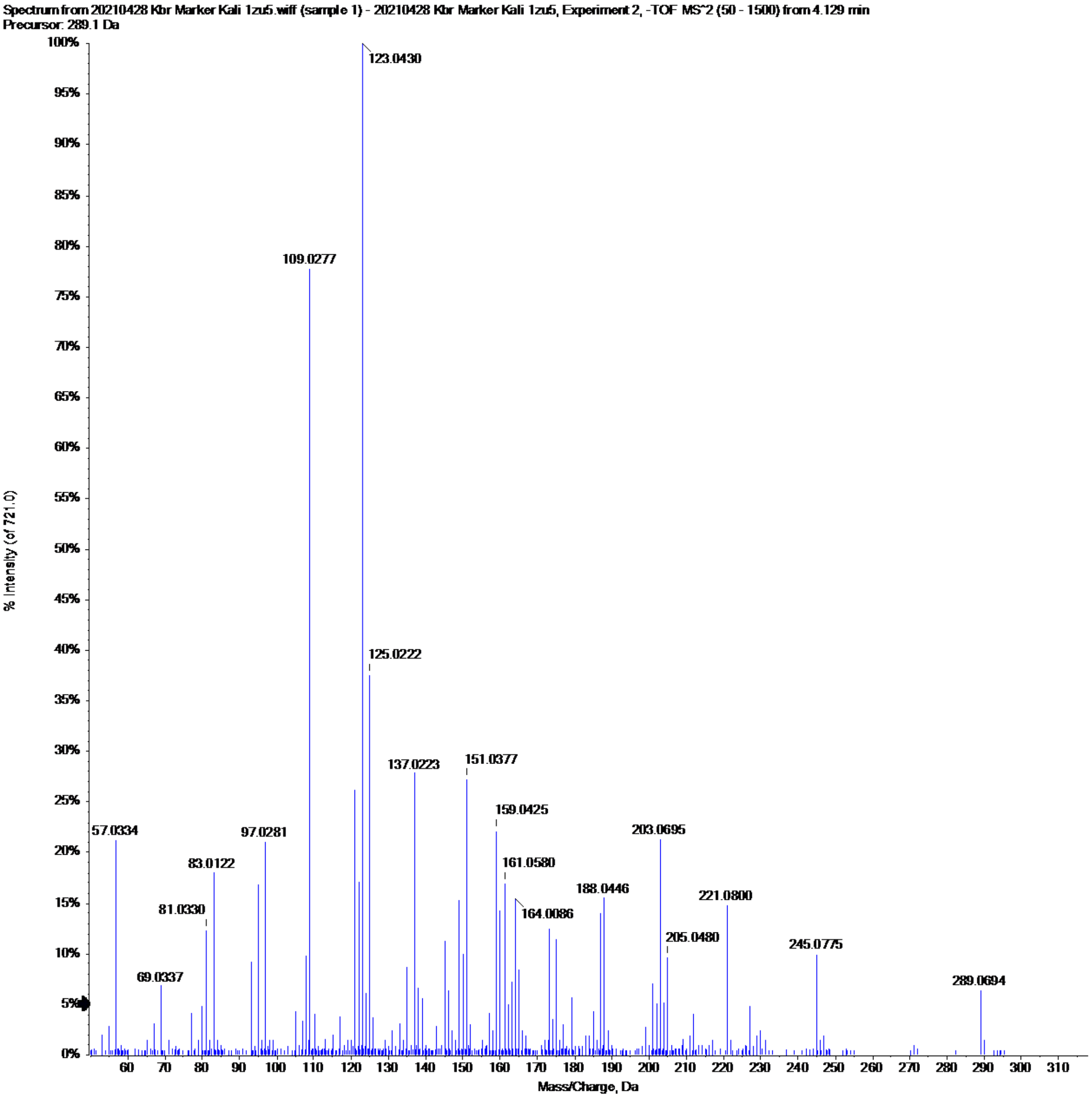

Figure S 125: qToF-MS/MS spectrum of standard solution.

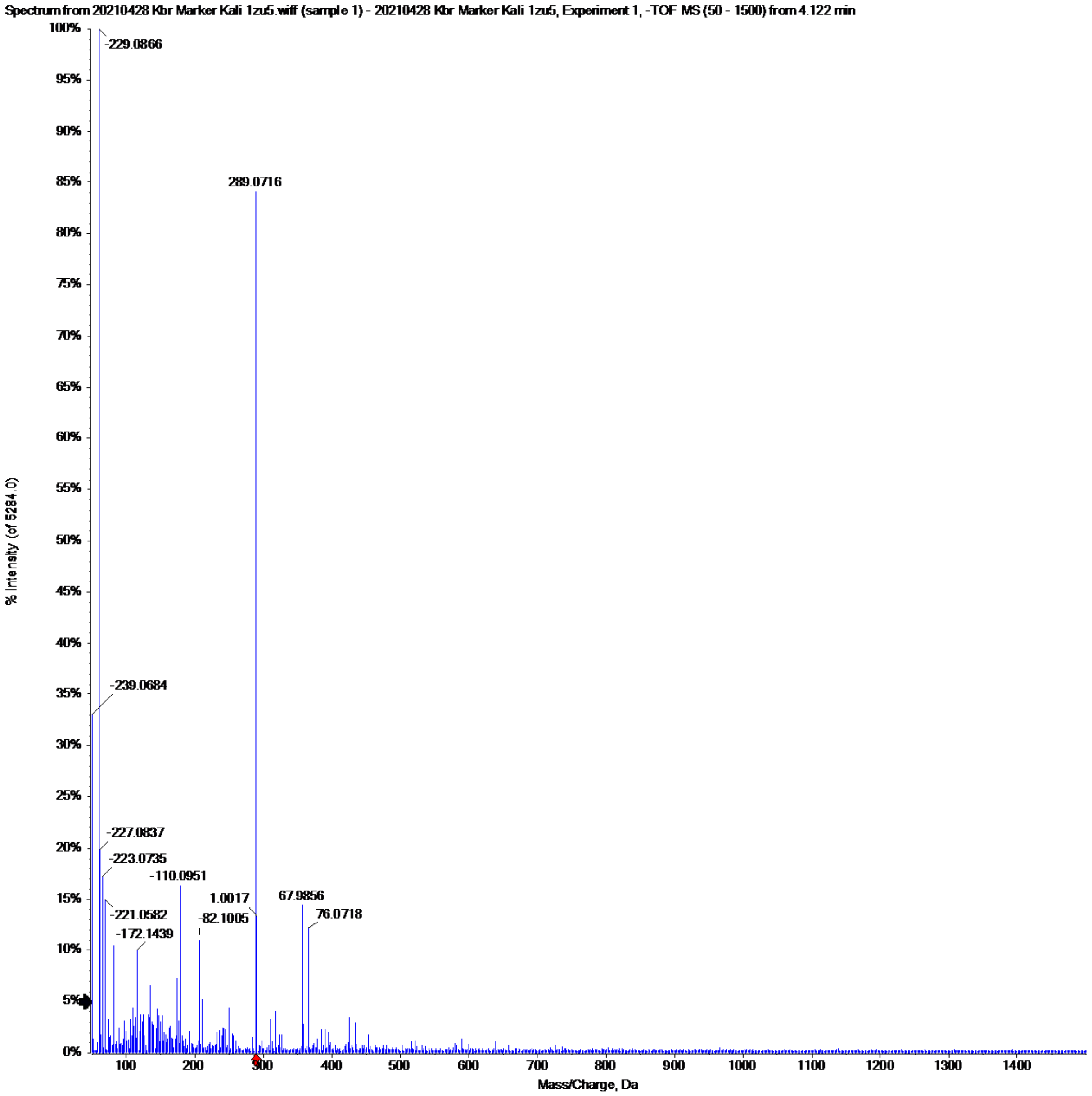

Figure S 126: qToF-MS spectrum (survey scan) of standard solution.

(-)-epicatechin

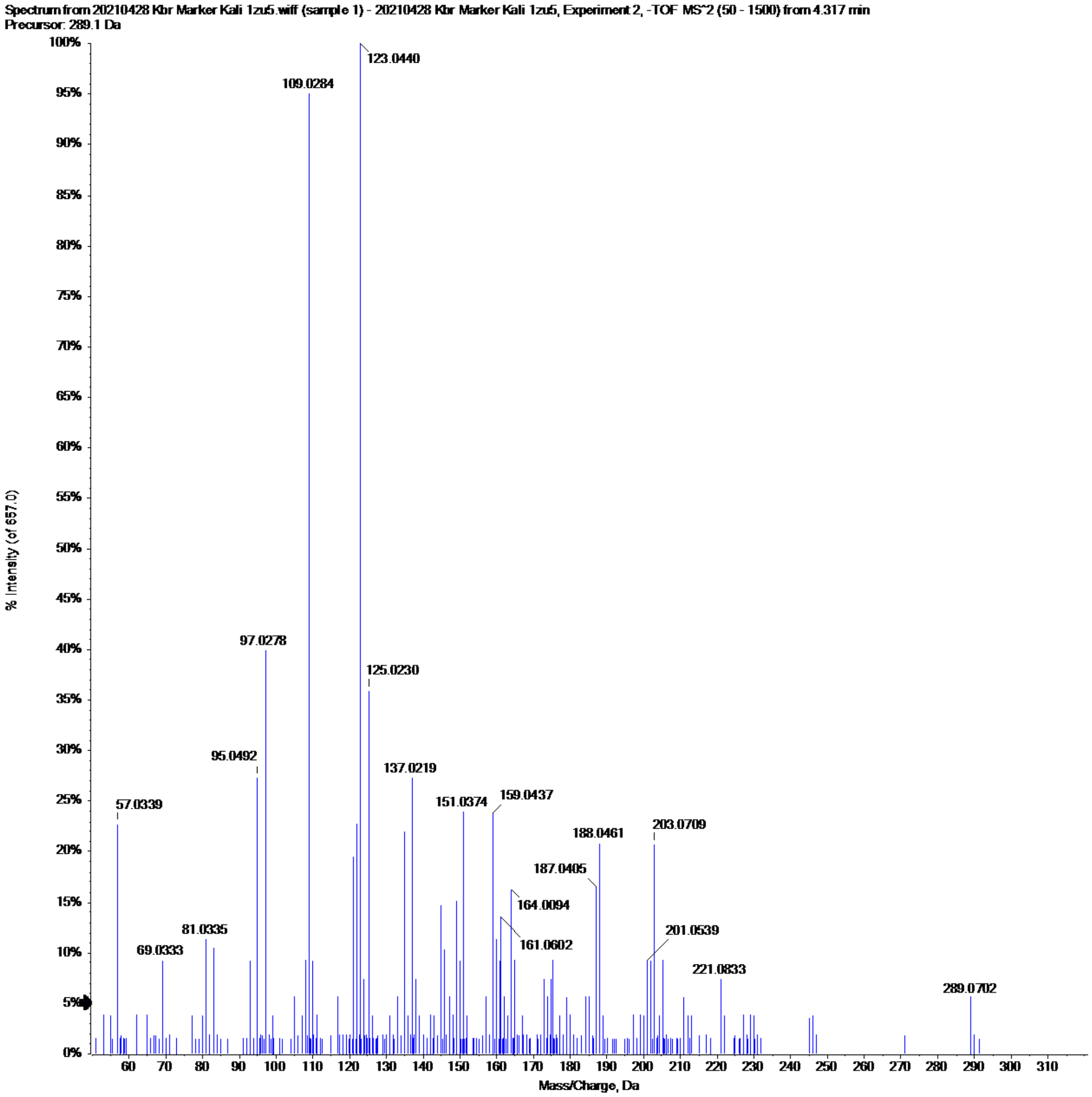

Figure S 127: qToF-MS/MS spectrum of standard solution.

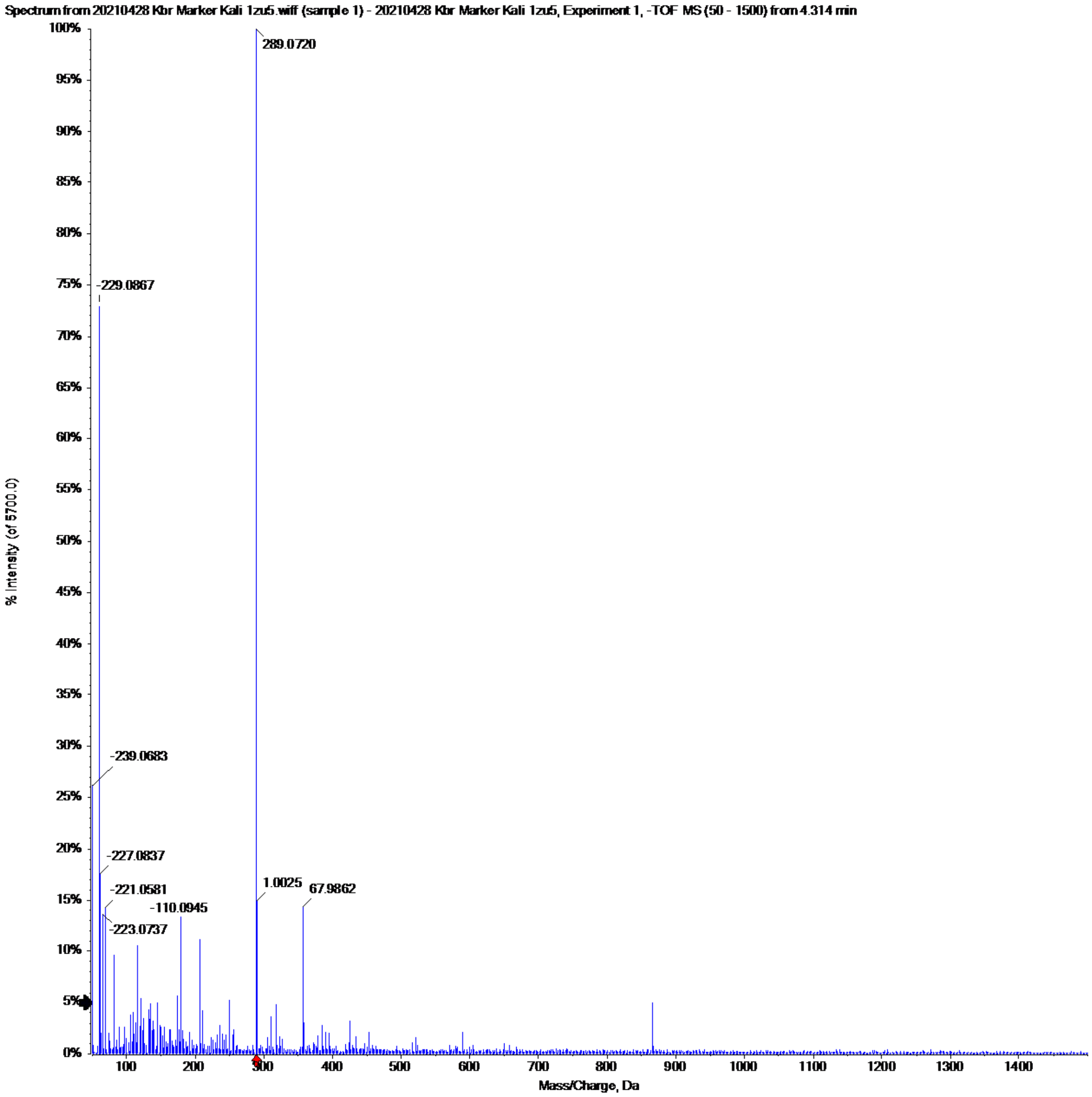

Figure S 128: qToF-MS spectrum (survey scan) of standard solution.
